# Solution-Phase Synthesis of Boranophosphate and Boranophosphate/Phosphorothioate/Phosphate Chimeric Oligonucleotides via the H‑Boranophosphonate Method

**DOI:** 10.1021/acs.joc.5c00583

**Published:** 2025-06-20

**Authors:** Yuhei Takahashi, Itsuki Kato, Kazuki Sato, Takeshi Wada

**Affiliations:** Department of Medicinal and Life Sciences, Faculty of Pharmaceutical Sciences, Tokyo University of Science, 6-3-1 Niijuku, Katsushika-ku, Tokyo 125-8585, Japan

## Abstract

Boranophosphate oligodeoxyribonucleotides (PB-ODNs) are promising candidates as antisense oligonucleotides (ASOs) owing to their high nuclease resistance and low cytotoxicity; however, their synthesis via the conventional phosphoramidite method is difficult. To address this issue, we developed an *H*-boranophosphonate method for the synthesis of PB-ODNs. Since the *H*-boranophosphonate diester intermediate in the *H*-boranophosphonate method is unstable during purification on silica gel, the synthesis of PB-ODNs longer than a 3-mer was conducted exclusively via solid-phase synthesis, which hinders the scalability of the method for the large-scale production of PB-ODNs. Herein, we report the first successful application of the *H*-boranophosphonate method to the solution-phase synthesis of PB-ODNs, overcoming the stability issues by converting an unstable *H*-boranophosphonate diester into a stable boranophosphotriester in a one-pot reaction. For efficient chain elongation, a block condensation strategy using 4-mer building blocks was employed. The block condensation and oxidative esterification proceeded efficiently to yield PB-ODNs with a length of up to 12-mer. Furthermore, the strategy was applicable to the synthesis of PB/phosphorothioate (PS)/phosphate (PO)-ODNs up to 12-mer using *H*-phosphonate and *H*-phosphonothioate derivatives. This advancement enables the scalable production of PB-ODNs and *P*-modified chimeric ODNs, paving the way for the development of more potent ASOs.

## Graphical abstract

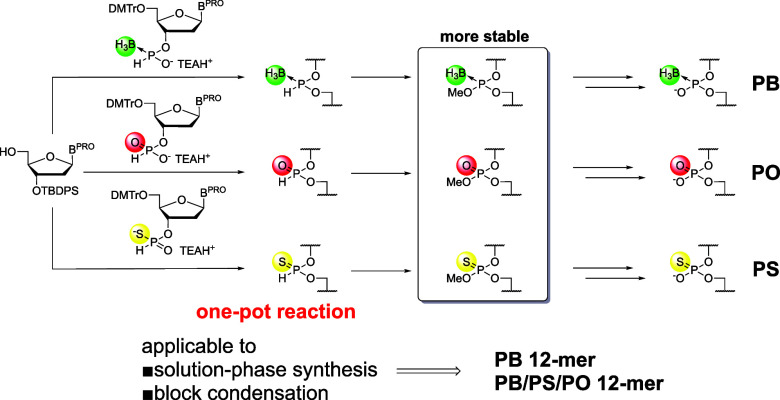

## Introduction

Antisense oligonucleotides (ASOs) control the translation of mRNA into proteins by binding the complementary mRNA with high affinity via Watson–Crick base pairing.^1,2^ ASOs can be roughly classified into two categories: the RNase H-dependent type and the steric blocking type. RNase H^3,4^ is an endonuclease that recognizes DNA/RNA duplexes and selectively cleaves the RNA strand. RNase H-dependent ASOs are designed to be complementary to the target mRNA. After forming DNA/RNA duplexes, RNase H is recruited to cleave the RNA strands, leading to a decrease in the levels of mRNA and proteins. Considering this mechanism, several properties such as high nuclease resistance, duplex stability, and RNase H-inducing ability are required to achieve highly potent RNase H-dependent ASOs. In general, these properties can be regulated by introducing chemical modifications to the phosphate backbone, sugar, and nucleobases of ASOs, which has been the focus of intense research aiming to enhance the efficacy of ASOs.

At present, phosphorothioate (PS), in which one of the nonbridging oxygen atoms of the phosphate (PO) moiety is replaced with a sulfur atom, is widely used for the *P*-modification of ASOs because of its substantial nuclease resistance and RNase H-inducing ability. Notably, PS-ASOs can be readily synthesized using the phosphoramidite method. In addition, PS modifications can contribute to blood retention,^5,6^ intracellular translocation,^7^ and other in vivo kinetics of ASOs that help enhance the efficacy of PS-ASOs.^8^ However, some PS derivatives induce cytotoxicity, which is a major obstacle in clinical trials.^9,10^ To obtain more potent and low-cytotoxic ASOs, many *P*-modified oligonucleotides (ODNs), such as mesyl phosphoramidate,^11–14^ phosphoryl guanidine,^15,16^ and alkylphosphonate,^17^ have emerged as promising ASO candidates. Moreover, replacing parts of the PS linkages with these linkages has been reported to reduce considerably the toxicity without compromising the antisense activity.^14,17^ According to these studies, by taking advantage of each other’s strength, *P*-modified chimeric ODNs can be expected to be the next generation of ASOs.

Boranophosphate (PB), in which one of the nonbridging oxygen atoms of the PO moiety is replaced with a borane group, is another promising *P*-modification for ASOs owing to its higher nuclease resistance^18^ compared with its PS counterpart and low cytotoxicity.^19,20^ Furthermore, *P*-modified chimeric ODNs containing PB linkages, such as PB/PS/PO chimeric ODNs^21^ and PB/PO chimeric ODNs,^21,22^ have been studied for their potential as ASOs. These studies suggested that introducing specific P-modifications to the proper sites of ODNs can help optimize their properties, such as nuclease resistance, duplex stability, and RNase H-inducing ability, which are critical for enhancing the efficacy and safety of ASOs.^21–23^ Given the challenges associated with PS cytotoxicity and the ongoing efforts to improve the properties of ASOs, PB and *P*-modified chimeric ODNs containing PB linkages are considered promising candidates for next-generation ASOs.

Despite the potential of PB-ODNs and P-modified chimeric ODNs containing PB linkages as ASO candidates, the synthesis of PB-ODNs using the standard phosphoramidite method is challenging. Although PB-ODNs can be theoretically synthesized via the phosphoramidite method by boronation of the phosphite triester intermediate, the acyl protecting groups on the nucleobases, which are necessary to prevent side reactions of the activated phosphoramidite monomer with the amino groups of nucleobases, are reduced in the boronation step to alkyl groups that cannot be removed under standard deprotection conditions.^24^ To overcome this problem, Caruthers et al. developed a synthetic method for PB-ODNs based on the phosphoramidite method using a protecting group that is stable under boronation conditions. Using *N*-di-*tert*-butylisobutylsilyl-protected phosphoramidite monomers, they obtained PB-ODNs and PB–PO chimeric ODNs up to 24-mer.^25^

Different from this approach, our group developed alternative strategies such as a boranophosphotriester method^26,27^ and an *H*-boranophosphonate method,^28,29^ which utilize monomers containing a borano group. In these methods, a boranophosphorylation reagent or an *H*-boranophosphonylation reagent containing a borano group is reacted with a 3′-hydroxy group of suitably protected nucleosides to produce the monomer units. The presence of a borano group in these reagents allows circumventing the boronation step in the synthesis of the monomer units and PB-ODNs. Therefore, the nucleobases of the monomer units can be protected by common acyl protecting groups.

The boranophosphotriester method^26,27^ uses deoxyribonucleoside 3′-boranophosphodiester monomer units with either a methyl or a cyanoethyl group as a protecting group at the phosphorus center. The monomer units are synthesized by reacting a boranophosphorylation reagent with a 3′-hydroxy group of a nucleoside derivative, followed by the removal of one of the protecting groups of the phosphorus center. In the next step, the monomer unit is condensed with the 5′-hydroxy group of another nucleoside derivative using a condensing reagent to form boranophosphotriester linkages. It should be noted that the boranophosphotriester linkage is stable during purification by silica gel column chromatography, allowing for the isolation of these compounds without any degradation.^26^ Subsequent deprotection of the boranophosphotriester and removal of the hydroxy and amino protecting groups furnish dinucleoside boranophosphates in good yields in solution. This approach can also be applied to the solid-phase synthesis of PB-ODNs. For chain elongation, repeated condensation of the monomer units with a 5′-hydroxy group of a nucleoside derivative on a solid support and 5′-detritylation are conducted until the desired chain elongation is achieved. Then, the deprotection of all protecting groups and release from the solid support affords PB-ODNs. However, the reactivity of the monomer units is moderate; therefore, the isolated yields of 12-mer PB-ODNs are not satisfactory.^27^

Meanwhile, in the *H*-boranophosphonate method, deoxyribonucleoside 3′-*H*-boranophosphonate monomer units containing characteristic H–P → BH_3_ groups are used. These monomer units are synthesized by condensing a pyridinium *H*-boranophosphonate containing bifunctional groups with suitably protected nucleosides.^28^ Then, repeated condensation of the monomer units and 5′-detritylation are conducted for chain elongation without transformation of the resultant *H*-boranophosphonate diester intermediates. After the designed chain elongation is achieved, all internucleotidic *H*-boranophosphonate diesters are oxidized to boranophosphodiester linkages via treatment with CCl_4_ and water in the presence of bases, followed by deprotection of the nucleobases and release from the solid support to afford PB-ODNs. Compared with the monomer units in the boranophosphotriester method, the *H*-boranophosphonate monomer unit exhibits higher reactivity; therefore, PB-ODNs can be efficiently synthesized using the *H*-boranophosphonate method.^29^ However, because the *H*-boranophosphonate diester intermediate is unstable under the purification conditions of silica gel column chromatography, the isolated yield of the dinucleoside *H*-boranophosphonate diester is moderate in the solution-phase synthesis.^28^ Thus, efficient solution-phase synthesis of PB-ODNs has not yet been achieved.

In addition to the synthesis of PB-ODNs using the *H*-boranophosphonate method, PB/PO-, PB/PS-, and PB/PS/PO-chimeric ODNs have been similarly synthesized utilizing *H*-boranophosphonate, *H*-phosphonothioate, and *H*-phosphonate monomers.^21,22^ This strategy, which enables the design of diverse *P*-modified chimeric ODNs for various applications, involves repeated condensation of *H*-boranophosphonate, *H*-phosphonothioate, and *H*-phosphonate monoesters with a 5′-hydroxy group to form *H*-boranophosphodiester, *H*-phosphonothioate, and *H*-phosphonate diester linkages, respectively, followed by 5′-detritylation without converting the resulting internucleotidic linkages until the desired chain elongation is achieved. Subsequently, simultaneous oxidation of the internucleotidic linkages to form PB, PS, and PO linkages, deprotection of the nucleobases, and release from the solid support are performed. This strategy allowed synthesizing several P-modified chimeric ODNs up to 12-mer on a solid support.^21^ A major challenge of this strategy was the chemoselective condensation of the *H*-phosphonothioate monomer, which contains two different nucleophilic centers, i.e., sulfur and oxygen atoms. To address this issue, phosphonium-type condensing reagents, which are hard electrophiles, were employed; according to the hard and soft acids and bases theory, the hard phosphorus center would preferentially react with the harder oxygen atom, avoiding undesired *S*-activation. In fact, the condensation reaction proceeded in a highly chemoselective manner, and PB/PS- and PB/PS/PO-chimeric ODNs were successfully synthesized.

However, these methods are restricted to solid-phase synthesis, which requires excess monomers and is less cost-effective for large-scale production. Moreover, no examples of the solution-phase synthesis of PB-ODNs longer than the 3-mer have been reported to date. Although the solid-phase synthesis involves simplified purification processes and allows the efficient production of diverse ODNs, developing a method for the solution-phase synthesis of PB-ODNs is essential for realizing a cost-effective large-scale production with predetermined sequences by reducing the required amounts of monomers compared to the solid-phase synthesis.

In this study, we developed a solution-phase synthetic method for PB-ODNs using the *H*-boranophosphonate method, overcoming the limitations of traditional solid-phase synthesis and enabling its application to large-scale production. As mentioned earlier, the biggest hurdle for applying the *H*-boranophosphonate method for solution-phase synthesis is the lability of *H*-boranophosphonate diesters on silica gel. To overcome this issue, we focused on the boranophosphotriester linkage because it is stable under silica gel column chromatography conditions. Therefore, a new strategy was conceived, as shown in Scheme 1. After an *H*-boranophosphonate monomer is condensed with a 5′-hydroxy group of a nucleoside derivative, the *H*-boranophosphonate diester is oxidized to a more stable boranophosphotriester via treatment with CCl_4_ and MeOH in the presence of bases. The proposed mechanism for the formation of the boranophosphotriester linkage consists of deprotonation, oxidative chlorination, and nucleophilic attack of MeOH on the resultant boranophosphorochloridate intermediate (Scheme 2, route A). However, the oxidative esterification is competitive with hydrolysis (Scheme 2, route B); therefore, suppressing the hydrolysis of the boranophosphorochloridate intermediate during the reaction is crucial to ensuring the formation of the desired product.

**Scheme 1:**
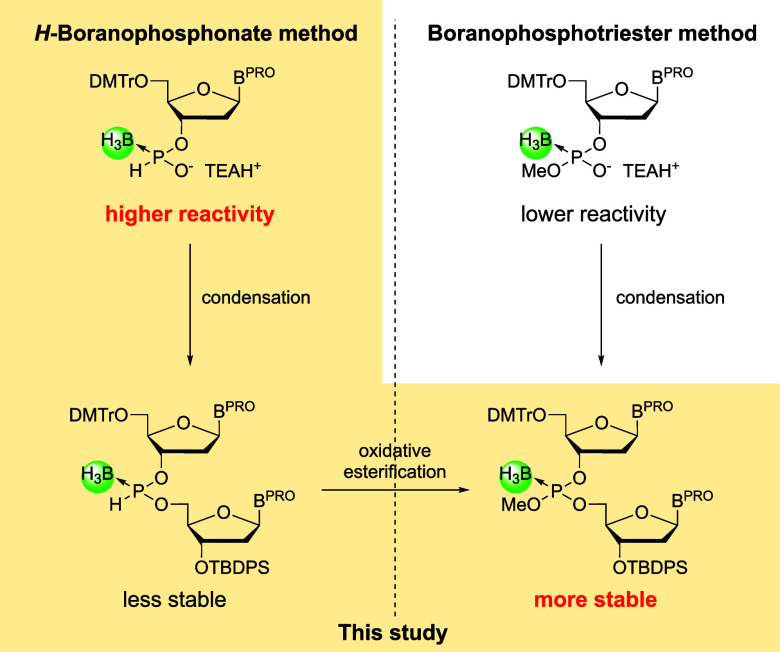
Strategy for Stabilizing *H*-Boranophosphonate Diesters in Solution-Phase Synthesis

**Scheme 2:**
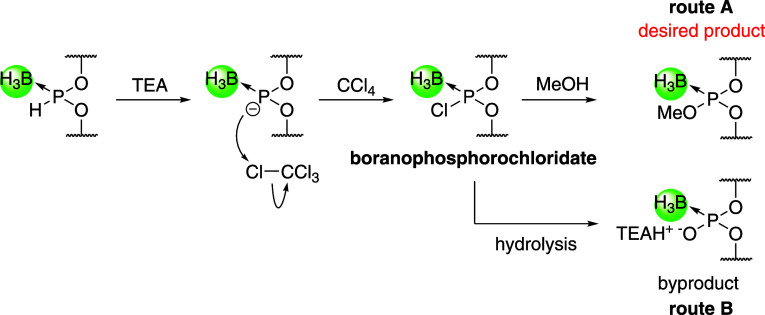
Plausible Mechanisms for the Oxidative Esterification of *H*-Boranophosphonate Diesters and Competitive Hydrolysis

For chain elongation, the block condensation strategy utilizing 4-mer building blocks was adopted because it reduces the total number of reaction steps and avoids the formation of inseparable shortmers such as N-1-mer, thereby simplifying the purification process. The 4-mer building blocks for chain elongation are synthesized according to Scheme 3. In brief, an *H*-boranophosphonate monomer is condensed with a 5′-hydroxy group of a nucleoside derivative whose 3′-hydroxy group is protected with a *tert*-butyldiphenylsilyl (TBDPS) group, followed by oxidative esterification to afford a dinucleoside boranophosphotriester (N_P(B)_N). It should be noted that the abbreviation P­(B) is used to represent the boranophosphotriester internucleotidic linkage, whereas PB denotes the boranophosphodiester linkage. Afterward, a 5′-upstream 2-mer building block is synthesized by removing the TBDPS group on the 3′-hydroxy group, followed by *H*-boranophosphonylation of the resulting 3′-hydroxy group. Meanwhile, a 3′-downstream 2-mer building block is prepared via 5′-detritylation. Next, two types of 4-mer building blocks are synthesized by using the 2-mer building blocks in a similar manner. Using the 4-mer building blocks, the condensation, oxidative esterification, and 5′-detritylation steps are repeated, until the desired chain elongation is achieved. Subsequently, removal of the methyl protecting group at the phosphorus center, the 3′-*O*-TBDPS group, and the acyl protecting groups on the nucleobases is conducted to give PB-ODNs.

**Scheme 3:**
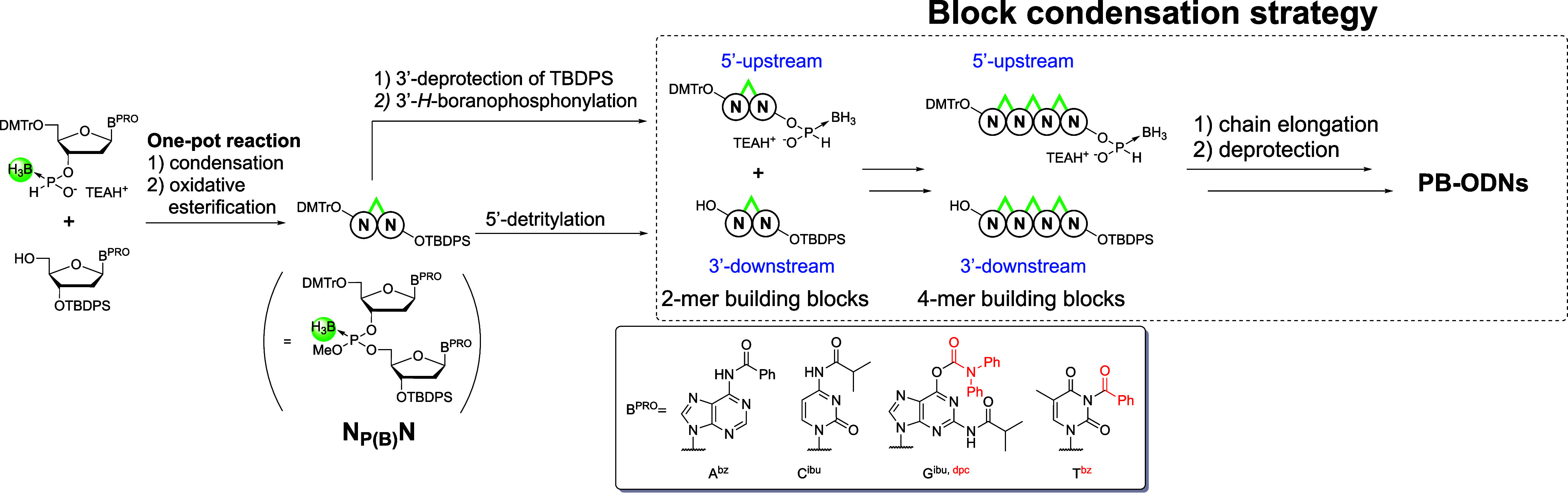
Preparation of 5′-Upstream and 3′-Downstream 4-Mer Building Blocks and Block Condensation

In this study, d­(T_PB_A_PB_G_PB_C_PB_T_PB_A_PB_G_PB_C_PB_T_PB_A_PB_G_PB_C) containing four nucleobases was selected as a synthetic target to demonstrate the potential of the strategy.

## Results and Discussion

### Synthesis of 2-Mer Building Blocks Containing a Boranophosphotriester Internucleotidic Linkage

First, the synthesis of 5′-upstream (T_P(B)_A) and 3′-downstream (G_P(B)_C) 2-mer building blocks was conducted. Each 2-mer building block was synthesized by utilizing deoxyribonucleoside 3′-*H*-boranophosphonate monomers protected with 4,4′-dimethoxytrityl (DMTr) groups at the 5′-hydroxy group as well deoxyribonucleosides with a free 5′-hydroxy group and TBDPS protection at the 3′-hydroxy group. The nucleobase amino groups of these deoxyribonucleosides were protected with acyl protecting groups to prevent unwanted side reactions with the activated monomer unit. Moreover, the *O* ^6^ position of guanine and the *N* ^3^ position of thymine were protected with diphenyl carbamoyl (Dpc) and benzoyl (Bz) groups, respectively,^30^ to prevent side reactions with phosphonium-type condensing reagents.^31^ It should be noted that the Dpc group was utilized for the *O* ^6^ position of guanine because it is more stable and easier to synthesize than the previously used cyanoethyl group,^29^ which is labile and easily eliminated during the preparation of the *H*-boranophosphonate monomer.^22^

For the preparation of T_P(B)_A, thymidine 3′-*H*-boranophosphonate monomer **2t** and deoxyadenosine derivative **1a** were used. *H*-Boranophosphonate monomer **2t** was condensed with the 5′-hydroxy group of compound **1a** using 3-nitro-1,2,4-triazol-1-yl-tris­(pyrrolidin-1-yl) phosphonium hexafluorophosphate (PyNTP)^32^ as a condensing reagent in the presence of 2,6-lutidine. The resulting *H*-boranophosphodiester was then oxidized to the corresponding boranophosphotriester via treatment with MeOH, triethylamine (TEA), and CCl_4_ in a one-pot reaction (Scheme 4). The crude mixture obtained after workup of the oxidative esterification reaction was analyzed by UHPLC and NMR analyses (Figure 1 and Supporting Information). In the ^31^P NMR spectrum (Figure 1), the signals of the boranophosphotriester (110–120 ppm) are predominantly observed, whereas signals corresponding to the *H*-boranophosphonate diester intermediate (130–140 ppm) and a boranophosphodiester (90–100 ppm), which can be formed via hydrolysis of the boranophosphorochloridate intermediate, were not detected. This result indicates that the condensation and oxidative esterification proceeded efficiently without notable hydrolysis of the boranophosphorochloridate intermediate. The success of this reaction can be attributed to a one-pot reaction procedure. The oxidative esterification was conducted in the presence of excess PyNTP, which also acts as a dehydration reagent to provide strict anhydrous conditions. It should be noted that excess *H*-boranophosphonate monoester (**2t**) reacts with MeOH to form the corresponding diester, which is subsequently oxidized to the boranophosphotriester **2′t** by CCl_4_, MeOH, and TEA.

**Scheme 4:**
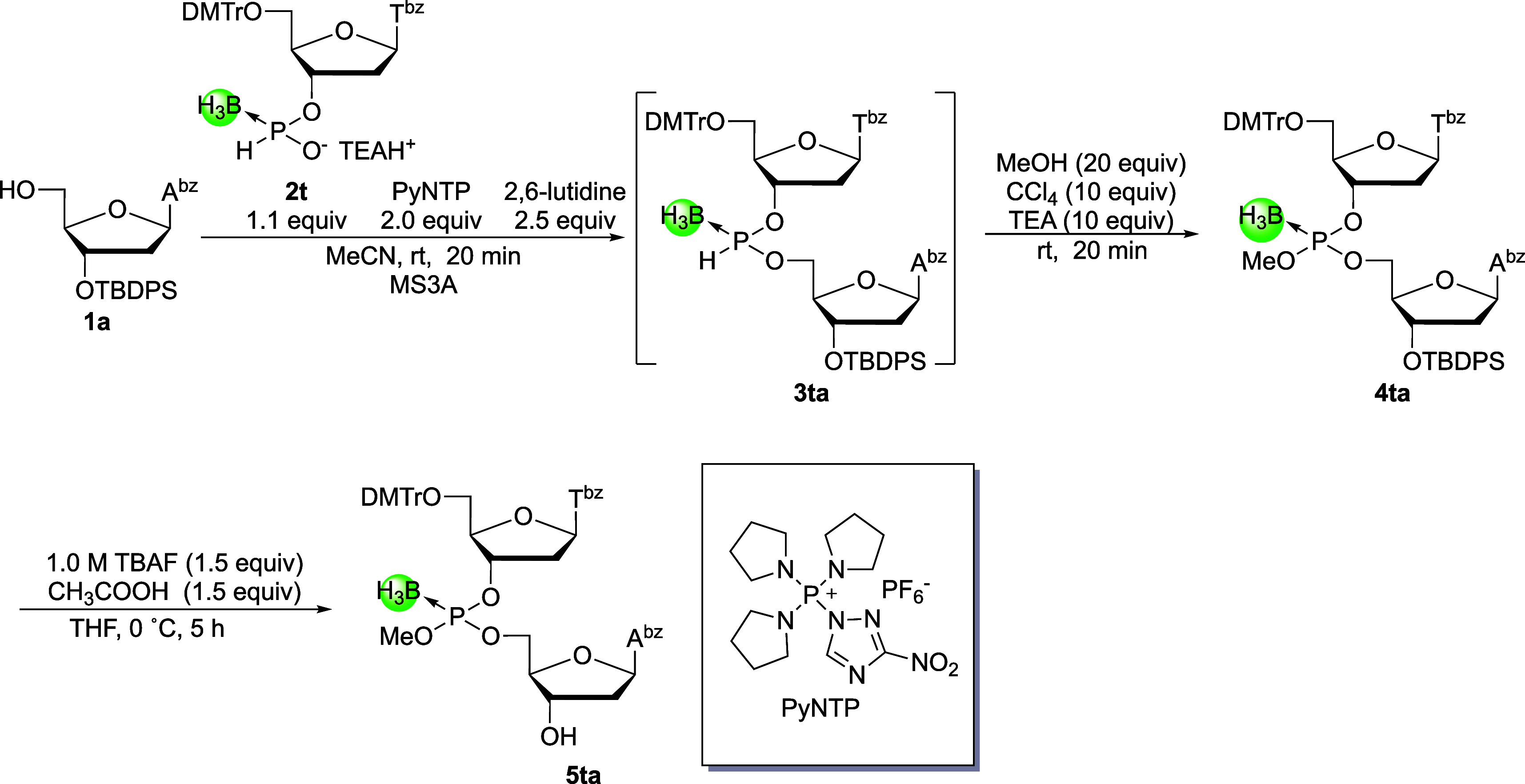
Preparation of the 2-Mer Building Block Having a 3′-Hydroxy Group

**Figure 1:**
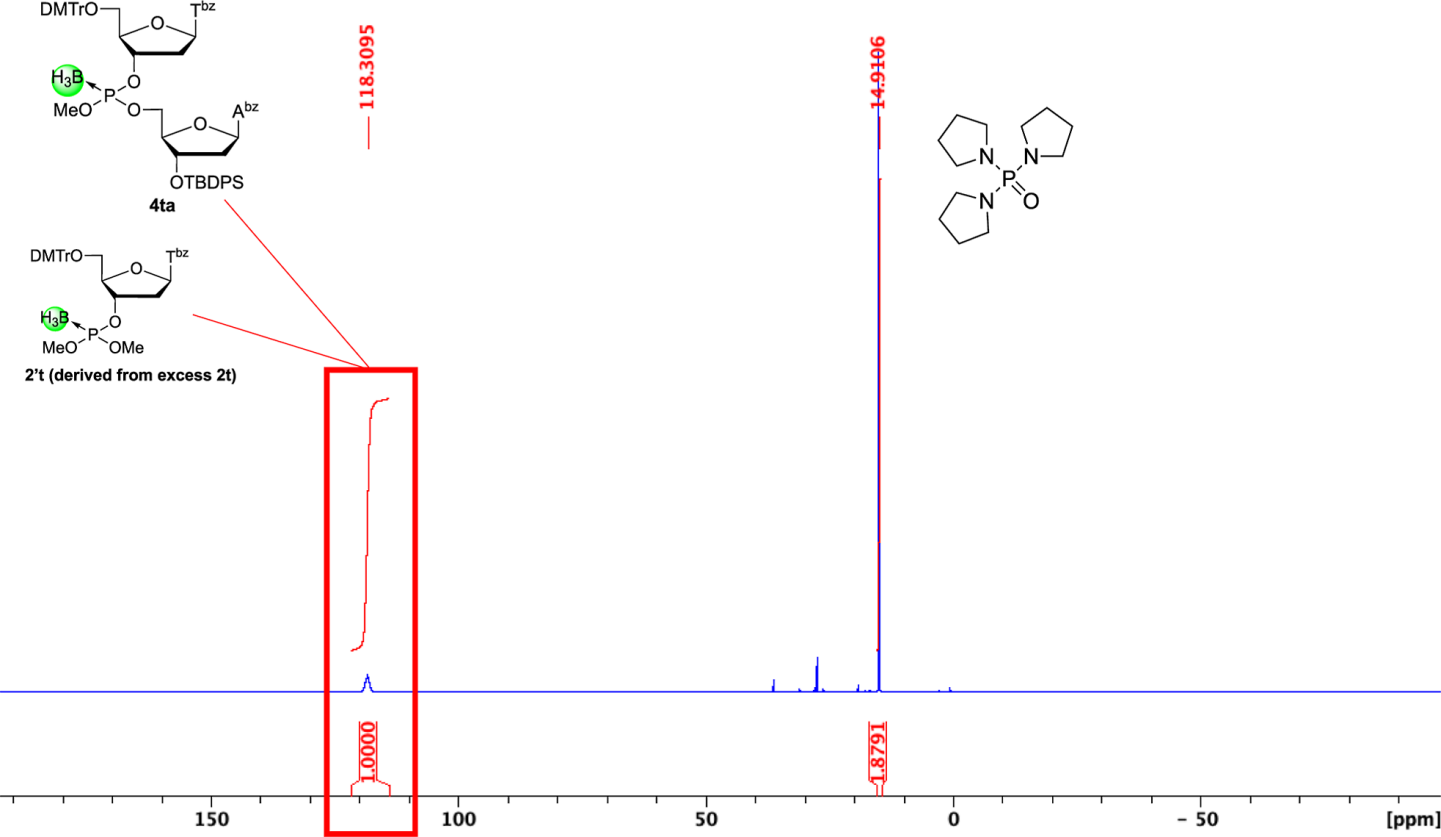
^31^P NMR spectrum of the crude mixture of compound **4ta** after workup of the oxidative esterification reaction.

Subsequently, the removal of the 3′-*O*-TBDPS group of compound **4ta** was performed via treatment with a solution of TBAF–CH_3_COOH (Scheme 4). After the 3′-*O*-TBDPS group was completely removed, the residue of the TBDPS group and the byproducts derived from the excess of monomer **2t** were removed by silica gel column chromatography purification. A signal of a PyNTP residue, a triaminophosphine oxide derivative, was detected in the ^31^P NMR spectrum after purification (Figure S1) and could not be removed completely during purification because the *R*_*f*_ values of compound **5ta** and the triaminophosphine oxide derivative were nearly identical. Nevertheless, compound **5ta** was used in the subsequent reaction.

Next, the *H*-boranophosphonylation of the resultant 3′-hydroxy group of compound **5ta** was performed under similar conditions to those used for the *H*-boranophosphonate monomer synthesis (**2a**, **c**, **g**, and **t**) using the bifunctional reagent **6** (Scheme 5).^28^ Compound **6** was condensed with the 3′-hydroxy group of compound **5ta** using bis­(2-oxo-3-oxazolidinyl)­phosphinic chloride (BopCl) in pyridine. The ^31^P NMR spectrum of the crude mixture obtained after workup (Figure S2) showed signals corresponding to the 5′-upstream 2-mer building block (**7ta**) as the main product (110–120 and 100–110 ppm). However, signals attributable to byproducts, such as a phosphite triester (140 ppm) and a boranophosphodiester (90–100 ppm), were also detected. These byproducts were likely formed during the *H*-boranophosphonylation or workup, possibly due to deboronation or elimination of the methyl group, respectively. Although the mechanism of the elimination of the methyl group remains unclear, phosphite triester **8ta** likely stemmed from the deboronation of boranophosphotriester **7ta** caused by the nucleophilic attack of pyridine. These findings suggested that pyridine is unsuitable as a reaction solvent for *H*-boranophosphonylation of the 2-mer. In addition, the high polarity of *H*-boranophosphonylation reagent **6** restricts its solubility to a narrow range of solvents, limiting its applicability. Furthermore, even if a different solvent could be employed, compound **7ta**, which is formed through the *H*-boranophosphonylation of compound **5ta**, is at the risk of reacting with the condensing reagent and condensing with another compound **5ta**, leading to a decrease in the isolated yield. These solvent restrictions and the risk of overactivation are detrimental to the versatility of the *H*-boranophosphonylation reagent; therefore, a new *H*-boranophosphonylation reagent with improved solubility and a monofunctional design was required to eliminate the challenges posed by bifunctional reactivity.

**Scheme 5:**
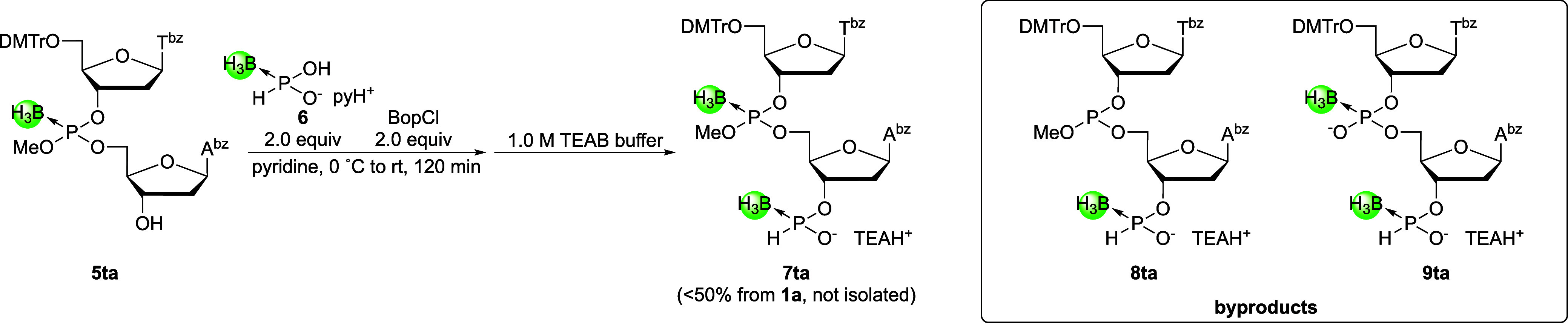
*H*-Boranophosphonylation Using Pyridinium Boranophosphonylation Reagent **6**

Accordingly, considering that a previously reported 9-fluorenylmethyl (Fm)-protected *H*-phosphonylation reagent is crystalline and easy to handle,^33^ a new *H*-boranophosphonylation reagent with one of the oxygen atoms of reagent **6** protected by an Fm group was designed. The *H*-boranophosphonylation reagent was synthesized from phosphinic acid as an *N*,*N*-diisopropylethylammonium salt, followed by condensation of 9-fluorenylmethanol, silylation, and boronation (Scheme S1). *H*-Boranophosphonylation reagent **10** was obtained as a crystalline potassium salt, which contrasts with the oily form of its triethylammonium salt. In addition, reagent **10** exhibited good solubility in a wide range of solvents.

With the new *H*-boranophosphonylation reagent **10** in hand, the 3′-*H*-boranophosphonylation conditions were investigated using 5′-*O*-DMTr-*N* ^3^-benzoyl thymidine (details are shown in the Supporting Information, Scheme S2), and the optimized conditions were applied to the synthesis of 5′-upstream 2-mer building block **7ta**. In brief, *H*-boranophosphonylation reagent **10** was condensed with the 3′-hydroxy group of compound **5ta** using propanephosphonic acid anhydride (T3P)^34^ as a condensing reagent, followed by the removal of the T3P residue via extraction. Then, the Fm group was deprotected using TEA in the presence of trimethylsilyl chloride (TMSCl) as a silylating reagent (Scheme 6). Under these conditions, excess *H*-boranophosphonylation reagent **10** was silylated, and the Fm group was removed to give a highly hydrophilic compound. T3P was used as a condensing reagent for the *H*-boranophosphonate monoester instead of PyNTP because it can be easily removed via extraction. As a result, the 5′-upstream 2-mer building block was isolated in a good yield (80%, five steps from **1a**, as a mixture of TEA and tetrabutylammonium (TBA) salts (TEA/TBA = 1:0.2, determined by ^1^H NMR)), demonstrating the efficiency and practicality of the new *H*-boranophosphonylation reagent.

**Scheme 6:**
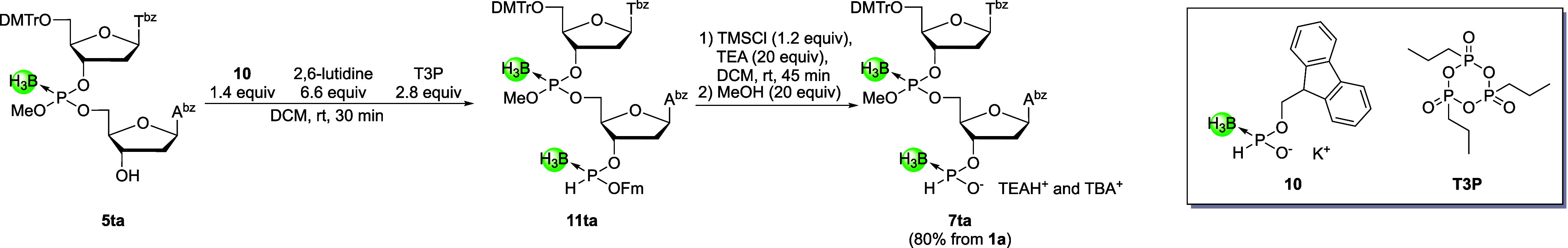
Preparation of 5′-Upstream 2-Mer Building Block

Meanwhile, the 3′-downstream 2-mer building block G_P(B)_C was synthesized as follows (Scheme 7). After the deoxyguanosine 3′-*H*-boranophosphonate monomer unit **2g** was condensed with the 5′-hydroxy group of deoxycytidine derivative **1c**, the resultant *H*-boranophosphonate diester was converted into the corresponding boranophosphotriester under the same conditions to give compound **4gc**. Subsequently, 5′-detritylation was conducted in the presence of 1-dodecanethiol as a cation scavenger^35^ to prevent the decomposition of the boranophosphotriester linkage by the DMTr cation.^36^ Consequently, the 3′-downstream 2-mer building block was successfully synthesized without decomposition of the boranophosphotriester linkage in a good yield (84%, three steps from **1c**).

**Scheme 7:**
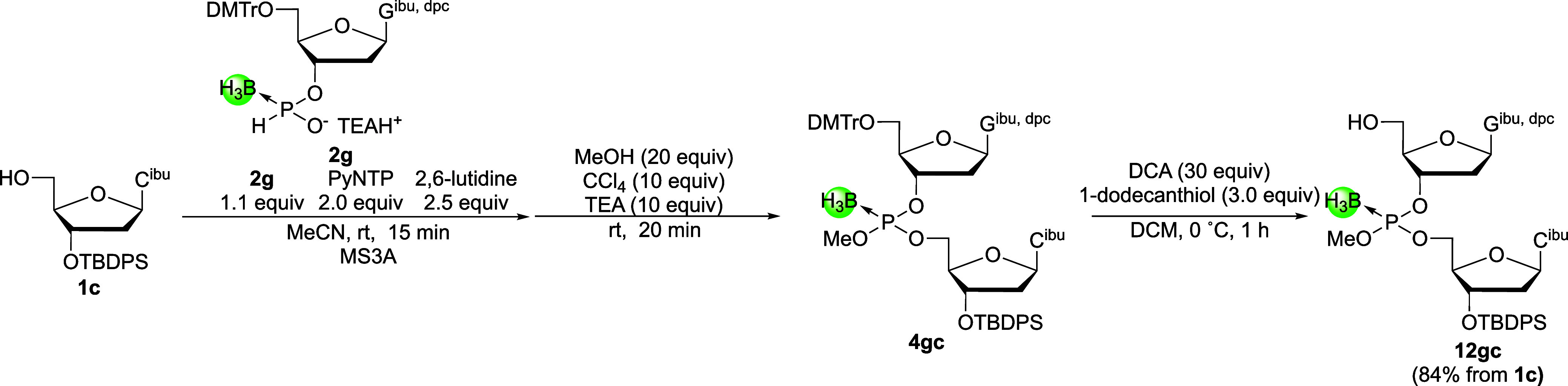
Preparation of the 3′-Downstream 2-Mer Building Block

### Synthesis of PB 4-Mer Building Blocks Containing Boranophosphotriester Internucleotidic Linkages

Next, 4-mer building blocks were synthesized using the obtained 2-mer building blocks **7ta** and **12gc** in a way similar to the synthesis of the 2-mer building blocks (Scheme 8). The 3′-*H*-boranophosphonate monoester of the 5′-upstream 2-mer building block **7ta** was condensed with the 5′-hydroxy group of the 3′-downstream 2-mer building block **12gc**, and the resultant *H*-boranophosphonate diester was oxidized into the corresponding boranophosphotriester. The crude mixture obtained after workup of the oxidative esterification was analyzed by ^31^P NMR (Figure S4). In the spectrum, the signal corresponding to the boranophosphotriester of 4-mer **13tagc** was predominantly observed, whereas no signals for an *H*-boranophosphonate diester (130–140 ppm) or a boranophosphodiester (90–100 ppm) were detected. Accordingly, the condensation and oxidative esterification proceeded efficiently without notable hydrolysis of the boranophosphorochloridate intermediate, even when 2-mer building blocks were used.

**Scheme 8:**
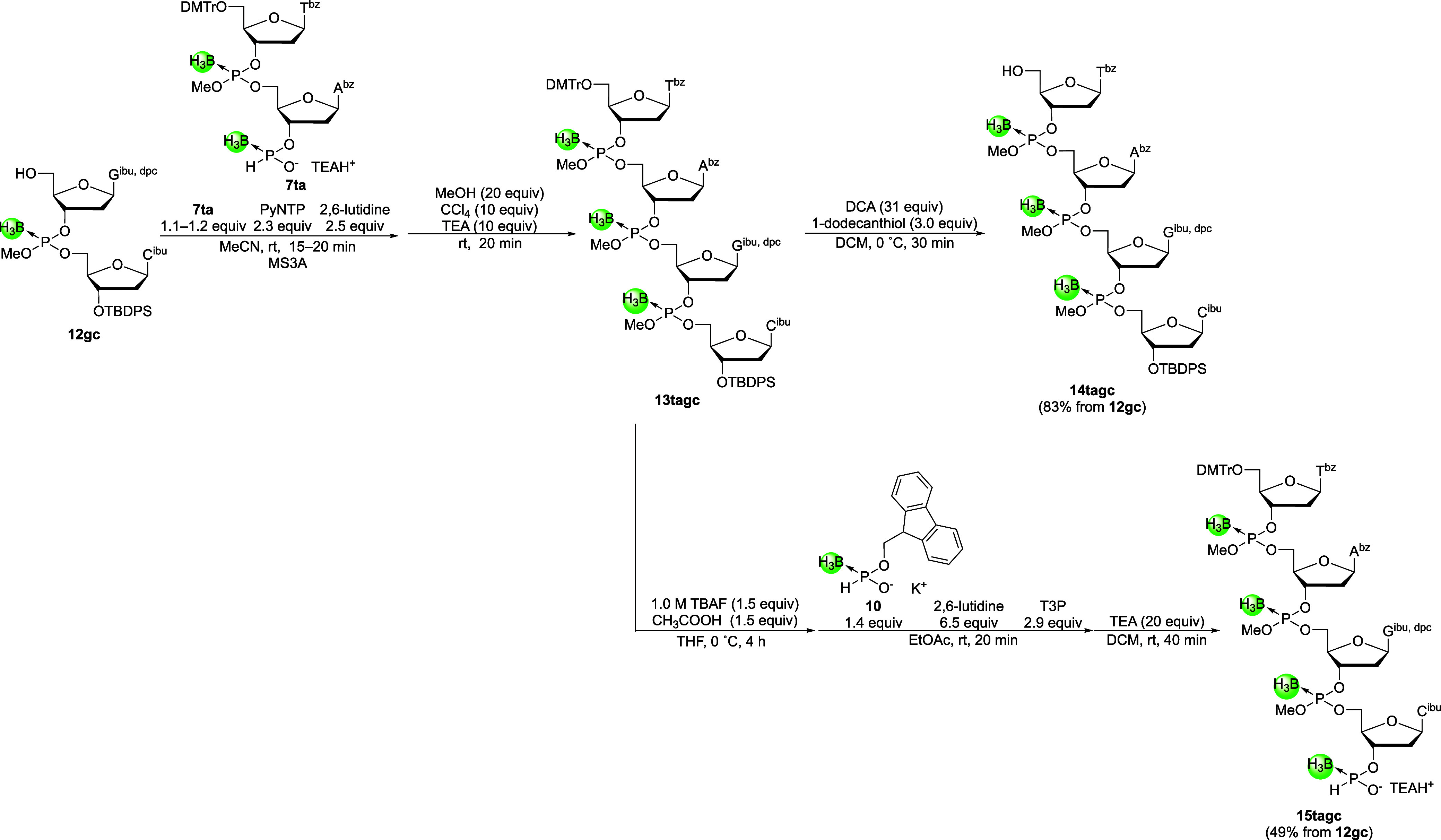
Preparation of 5′-Upstream and 3′-Downstream 4-Mer Building Blocks

Subsequently, 5′-detritylation of compound **13tagc** furnished 3′-downstream 4-mer building block **14tagc** in good yield (83%, three steps from **12gc**).

Meanwhile, deprotection of the 3′-*O*-TBDPS group in **13tagc** and subsequent *H*-boranophosphonylation afforded 5′-upstream 4-mer building block **15tagc** in a moderate yield (49%, five steps from **12gc**). The lower yield obtained in this case was attributed to unintended elimination of the methyl group during the deprotection of the TBDPS and Fm groups. Despite this side reaction, the desired 5′-upstream 4-mer building block **15tagc** was successfully isolated via silica gel chromatography. However, the moderate isolated yield highlights the need for improved protection and deprotection strategies that suppress the elimination of phosphorus protecting groups, which remains a limiting factor in achieving higher yields. To suppress this, one potential strategy could be the use of a more robust protecting group, which may offer greater stability under the current conditions.

### Chain Elongation Using the 4-Mer Building Blocks, Deprotection, and Isolation

Next, chain elongation using the block condensation strategy was conducted with the obtained 5′-upstream and 3′-downstream 4-mer building blocks. First, the 3′-*H*-boranophosphonate monoester of compound **15tagc** was condensed with the 5′-hydroxy group of compound **14tagc** in the presence of PyNTP, and the resultant *H*-boranophosphonate diester linkage was then oxidized to the corresponding boranophosphotriester. When using 1.2 equiv of the 5′-upstream 4-mer building block and 3.0 equiv of PyNTP, the condensation and oxidative esterification did not proceed sufficiently, suggesting that the reactivity of the building block decreased as the chain length increased. Thus, 1.4 equiv of **15tagc** and 5.8 equiv of PyNTP were used for the block condensation (Scheme 9), which proceeded with nearly complete consumption of starting material **14tagc**, as confirmed by UHPLC (Figure S5). Afterward, 5′-detritylation of the resulting 8-mer **16** and purification of the crude mixture by silica gel column chromatography yielded 8-mer **17**, as revealed by ESI–MS and ^31^P NMR analyses. However, complete separation of the byproducts derived from the excess of **15tagc** was difficult, most likely due to the presence of multiple diastereomers of the 8-mer, leading to extended elution tailing during purification by silica gel column chromatography, and the similar *R*_*f*_ values on TLC of the byproduct derived from **15tagc** (**15′tagc**). Although the presence of byproduct **15′tagc** could complicate subsequent reactions, the next step of the process was performed.

**Scheme 9:**
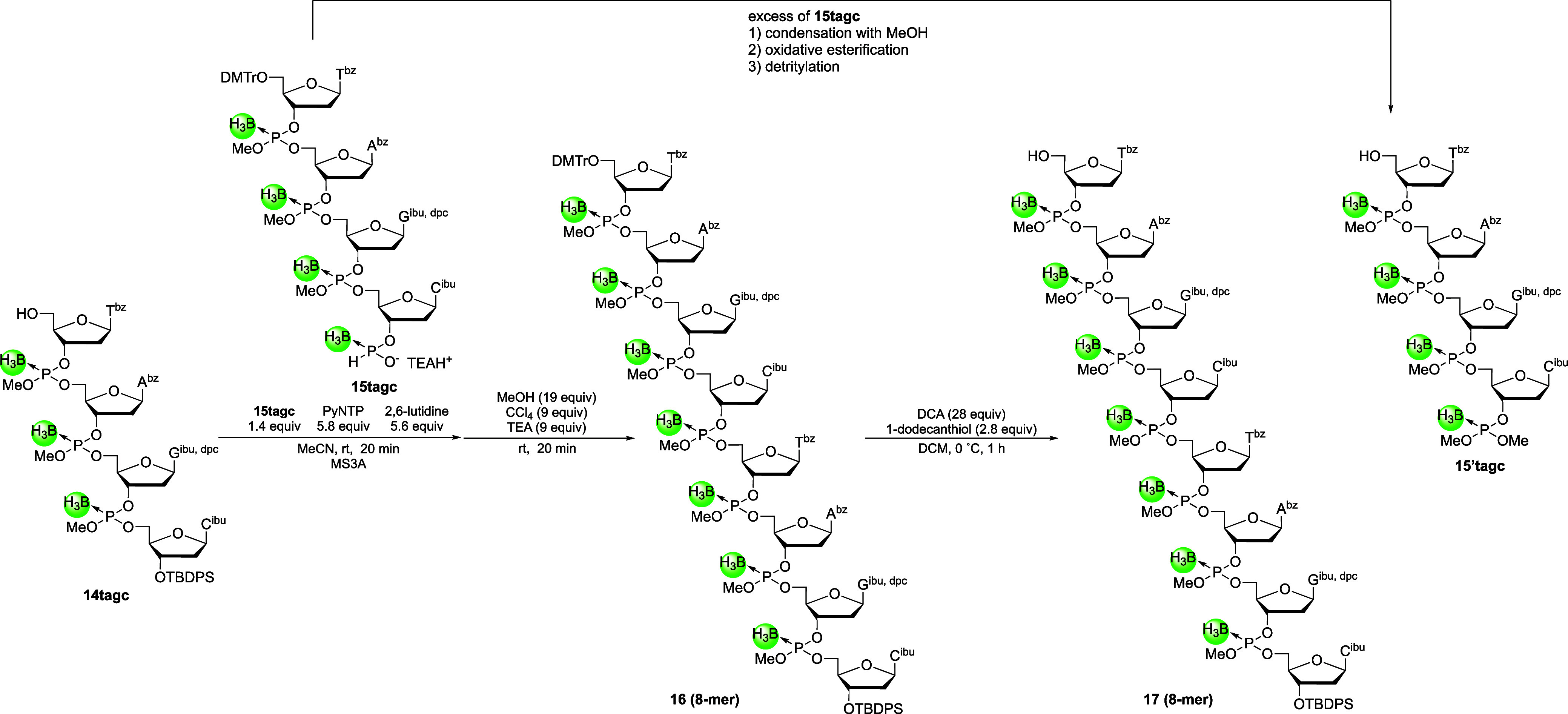
Block Condensation between 4-Mer Building Blocks

Next, the 3′-*H*-boranophosphonate monoester of compound **15tagc** (1.3 equiv) was condensed with the 5′-hydroxy group of 8-mer **17**, and the resulting *H*-boranophosphonate diester was oxidized to the corresponding boranophosphotriester. Since compound **15′tagc** could react with and consume the *H*-boranophosphonate monoester, it was concern that the presence of compound **15′tagc** interfered with the next block condensation reaction. Nonetheless, using 1.3 equiv of **15tagc** allowed the condensation to proceed efficiently, as confirmed by a preliminary UHPLC analysis (Figure S8). Then, deprotection of all protecting groups was performed via detritylation, removal of the methyl group using disodium 2-carbamoyl-2-cyanoethylene-1,1-dithiolate,^37^ removal of the TBDPS group, and deprotection of the nucleobases by treating with concentrated NH_3_ aq–EtOH (Scheme 10). TEA–3HF was used for the deprotection of the TBDPS group instead of TBAF–CH_3_COOH because the former reagent could be easily removed. A UPLC analysis of the crude mixture obtained after NH_3_ treatment revealed that the desired PB 12-mer was the main product. The fully deprotected PB 12-mer was then purified by ODS silica gel column chromatography, and the PB 12-mer **(18**) was isolated in an acceptable yield (6%, nine steps from **14tagc**). The 12-mer was characterized by ESI–MS, ^1^H NMR, and ^31^P NMR analyses. Although the presence of diastereomers and unintended elimination of the methyl group during the deprotection of the TBPDS group and *H*-boranophosphonylation hindered the purification of 8-mer **17**, PB-ODNs were successfully synthesized in modest yield by converting the *H*-boranophosphonate diester into a boranophosphotriester in solution using a block condensation strategy. While the overall yield of the PB 12-mer was modest (6% over nine steps), this solution-phase approach significantly reduced the amount of monomers required compared to solid-phase synthesis. While ∼40 equiv of an *H*-boranophosphonate monomer and ∼100 equiv of a condensing reagent were used per coupling step in solid-phase synthesis,^29^ the present method used only 1.1–1.4 and 2.0–6.0 equiv of an *H*-boranophosphonate monoester and a condensing reagent, respectively, highlighting its potential for cost-effective and scalable production.

**Scheme 10:**
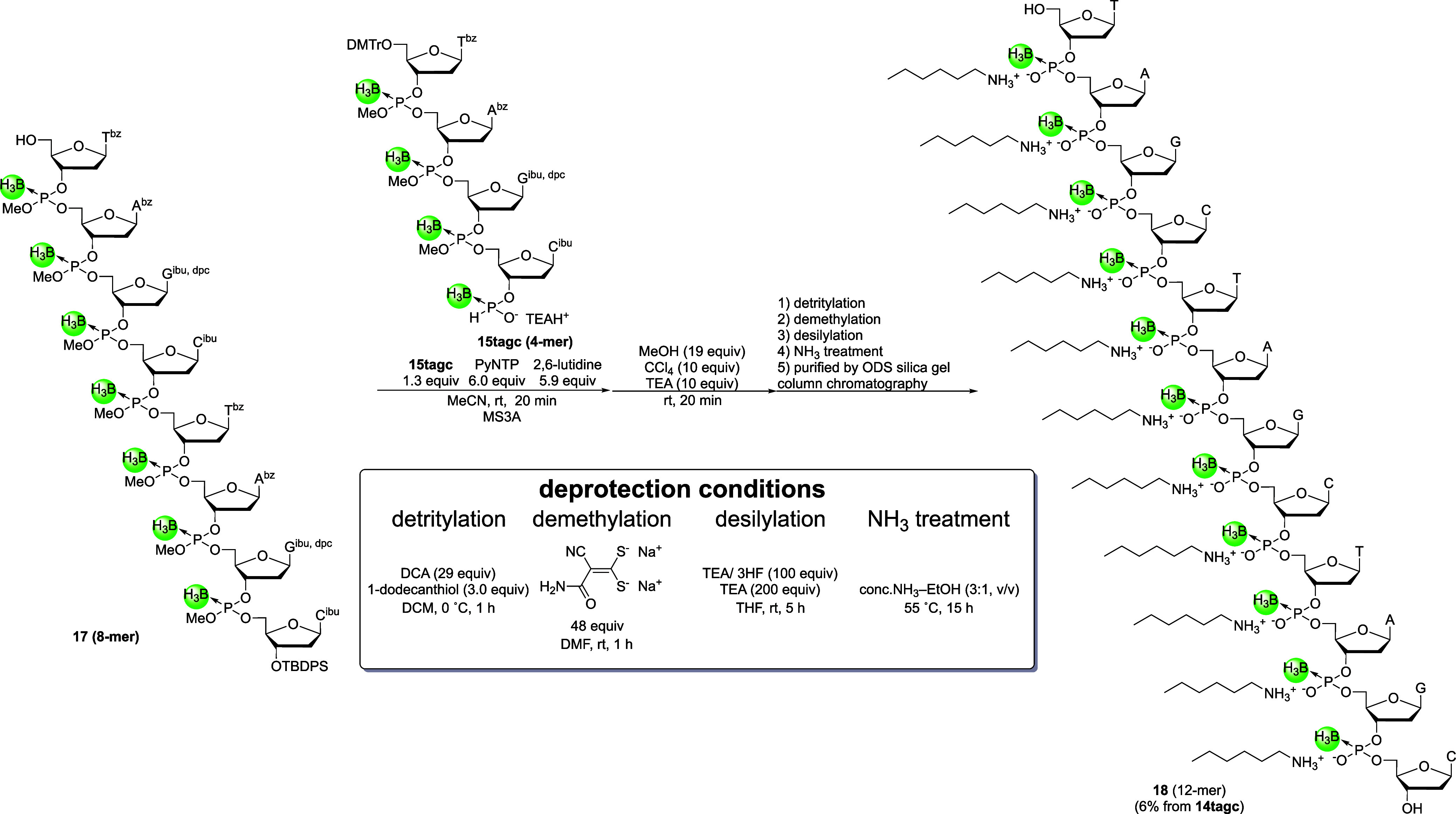
Block Condensation and Complete Removal of Protecting Groups

This result prompted us to synthesize PB/PS/PO chimeric ODNs by subjecting the *H*-phosphonothioate and *H*-phosphonate diester linkages to oxidative esterification.

### Synthesis of 2-Mer Building Blocks Containing Phosphotriester or Phosphorothioate Triester Internucleotidic Linkages

Next, oxidative esterification was applied to the *H*-phosphonothioate and *H*-phosphonate diester linkages to synthesize PB/PS/PO chimeric ODNs. In brief, the *H*-phosphonothioate and *H*-phosphonate diesters were converted to phosphorothiochloridate and phosphorochloridate derivatives in the presence of TEA and CCl_4_, followed by the nucleophilic attack of MeOH to give the phosphorothioate triester N_P(S)_N and phosphotriester N_P(O)_N, respectively. It should be noted that the abbreviations P­(O) and P­(S) indicate phosphotriester and phosphorothioate triester internucleotidic linkages, whereas PO and PS denote phosphodiester and phosphorothioate diester linkages, respectively. In the synthesis of the PB/PS/PO chimeric ODN, d­(A_PO_T_PB_C_PS_G_PB_A_PO_T_PB_C_PS_G_PB_A_PO_T_PB_C_PS_G) was chosen as the synthetic target sequence to demonstrate the effectiveness and applicability of this strategy.

A similar oxidative esterification of *H*-phosphonothioate and *H*-phosphonate diesters was reported, where the introduction of an alcohol into these intermediates was performed in the presence of iodine using phosphoroiodidate derivatives^38^ (Scheme 11). These reactions required strictly anhydrous conditions to avoid the hydrolysis of the phosphoroiodidate intermediates, which are highly sensitive to moisture.^39^ Thus, the oxidative esterification using CCl_4_, TEA, and MeOH also requires strictly anhydrous conditions. Building on our success in suppressing the hydrolysis of a boranophosphorochloridate during oxidative esterification via a one-pot reaction using PyNTP as a dehydration reagent, we hypothesized that this approach could also effectively suppress the hydrolysis of phosphorochloridate derivatives.

**Scheme 11:**
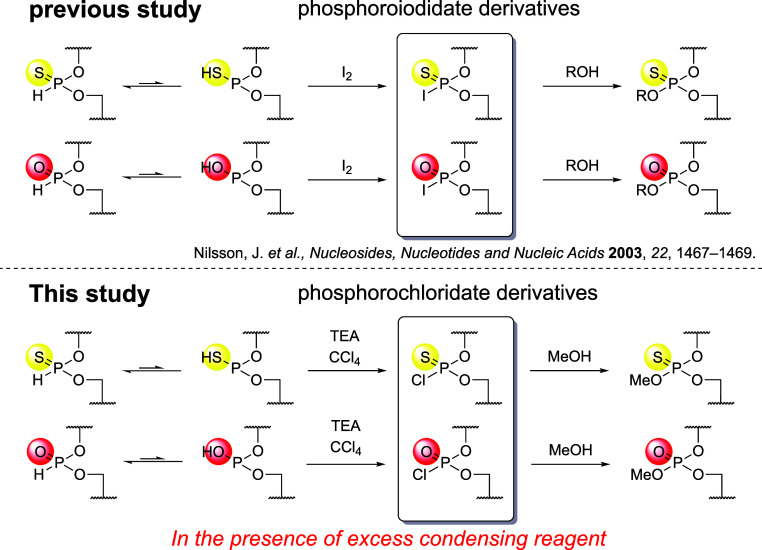
Previous Study and This Study of the Oxidative Esterification of an *H*-Phosphonate Diester and an *H*-Phosphonothioate Diester

First, condensation of the *H*-phosphonate monomer and oxidative esterification of the *H*-phosphonate diester linkage into a phosphotriester were conducted in a way similar to the synthesis of boranophosphotriesters to synthesize 5′-upstream 2-mer building block A_P(O)_T. Deoxyadenosine 3′-*H*-phosphonate monomer **19a** was condensed with the 5′-hydroxy group of thymidine derivative **1t** using PyNTP. Then, the resulting *H*-phosphonate diester linkage was oxidized into a phosphotriester via treatment with CCl_4_, TEA, and MeOH in a one-pot reaction (Scheme 12). Subsequently, deprotection of the 3′-*O*-TBDPS group and *H*-boranophosphonylation of the resultant 3′-hydroxy group afforded the 5′-upstream 2-mer building block **23at** in a moderate yield (59%, five steps from **1t**; Scheme 12).

**Scheme 12:**
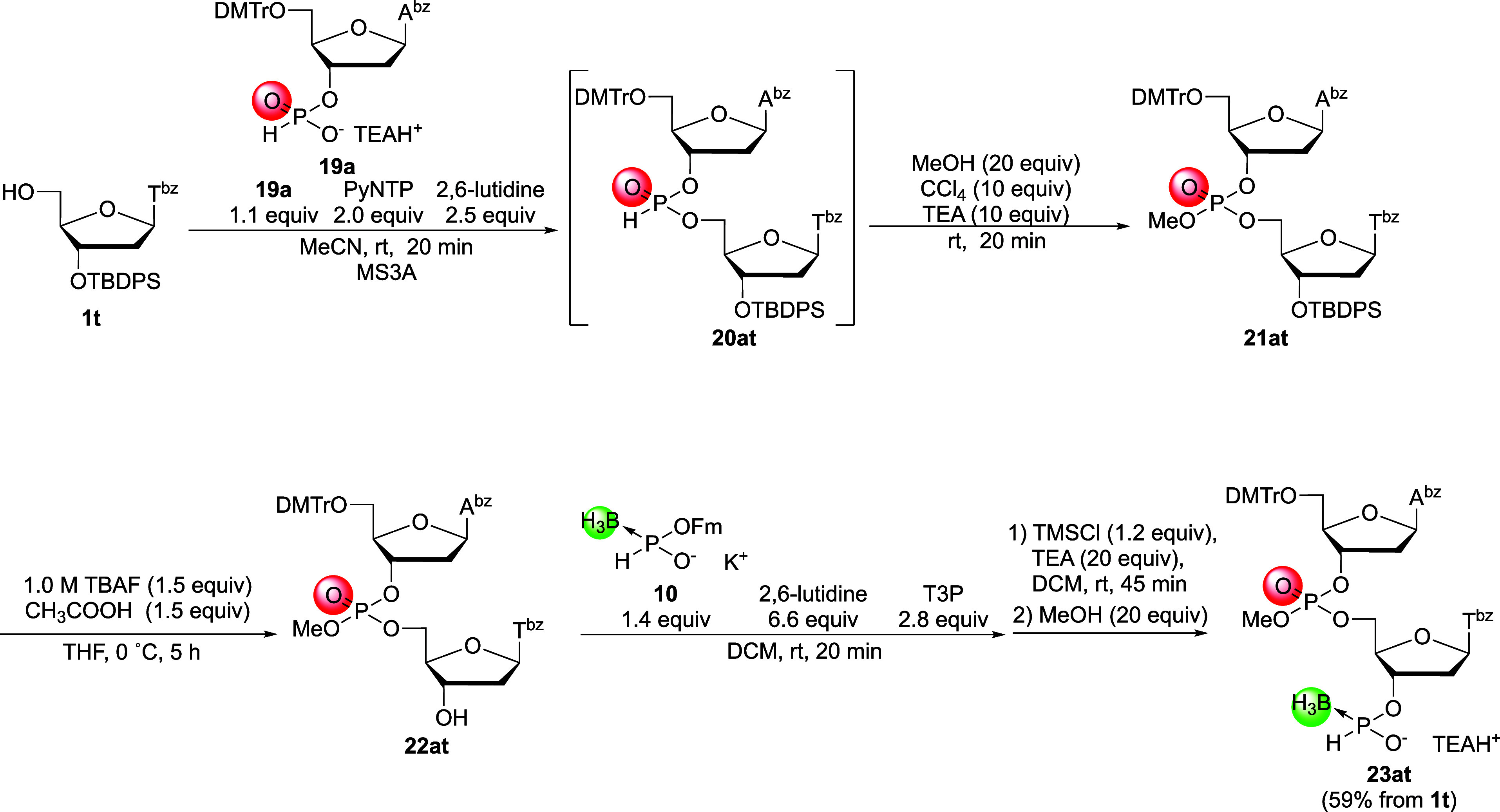
Synthesis of the 5′-Upstream 2-Mer Building Block A_P(O)_T

Next, condensation of the *H*-phosphonothioate monomer and oxidative esterification of the resultant *H*-phosphonothioate diester into a phosphorothioate triester linkage were conducted. The major problem associated with using *H*-phosphonothioate monomers is the chemoselectivity during condensation because, as mentioned earlier, these monomers contain two types of nucleophiles (oxygen and sulfur). Therefore, deoxycytidine 3′-*H*-phosphonothioate monomer **24c** ^21^ was condensed with the 5′-hydroxy group of deoxyguanosine derivative **1g** using PyNTP, which has a hard phosphonium center, enabling the *O*-selective activation of the monomer. Subsequently, oxidative esterification of the resulting *H*-phosphonothioate diester yielded the phosphorothioate triester. Finally, 5′-detritylation of compound **26cg** gave 3′-downstream 2-mer building block **27cg** in a good yield (89%, three steps from **1g**; Scheme 13).

**Scheme 13:**
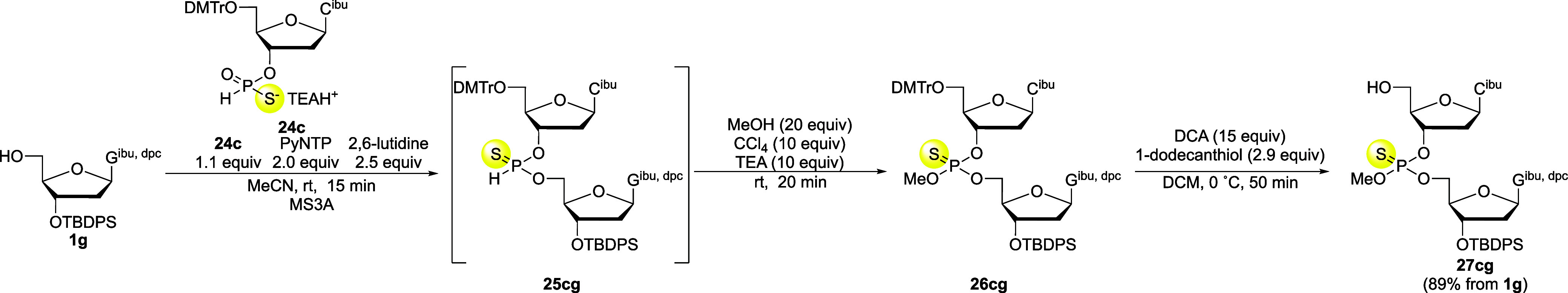
Synthesis of the 3′-Downstream 2-Mer Building Block C_P(S)_G

These results demonstrate that the oxidative esterification of these intermediates also proceeded efficiently without notable hydrolysis of the phosphorochloridate derivatives in a one-pot reaction in the presence of PyNTP.

### Synthesis of 4-Mer Building Blocks Containing Boranophosphotriester, Phosphorothioate Triester, and Phosphotriester Internucleotidic Linkages

Next, 5′-upstream and 3′-downstream 4-mer building blocks were synthesized using 2-mer building blocks **23at** and **27cg** (Scheme 14). The 3′-*H*-boranophosphonate monoester of 5′-upstream 2-mer building block **23at** was condensed with the 5′-hydroxy group of 3′-downstream 2-mer building block **27cg** using PyNTP, and the resulting *H*-boranophosphonate diester linkage was oxidized to give **28atcg** containing a boranophosphotriester linkage. In the ^31^P NMR spectrum of the crude mixture obtained after workup of the oxidative esterification reaction (Figure S13), signals ascribable to the boranophosphotriester, phosphorothioate triester, and phosphotriester of PB/PS/PO chimeric 4-mer **28tagc** were predominantly observed, indicating that the condensation and oxidative esterification proceeded efficiently without notable side reactions. Afterward, 5′-detritylation of 4-mer **28atcg** was performed, furnishing the 3′-downstream 4-mer building block in a relatively good yield after isolation (67%, three steps from **27cg**). Meanwhile, the 5′-upstream 4-mer building block was obtained from compound **28atcg** via deprotection of the 3′-*O*-TBDPS group and H-boranophosphonylation of the resultant 3′-hydroxy group in a moderate yield (40%, five steps from **27cg**). This lower yield was attributed to the unintended elimination of methyl groups during the deprotection of the TBDPS group and subsequent *H*-boranophosphonylation, as observed in the synthesis of PB 5′-upstream 4-mer building blocks. The suppression of the methyl group elimination remains crucial for increasing the yields and optimizing the overall synthetic process.

**Scheme 14:**
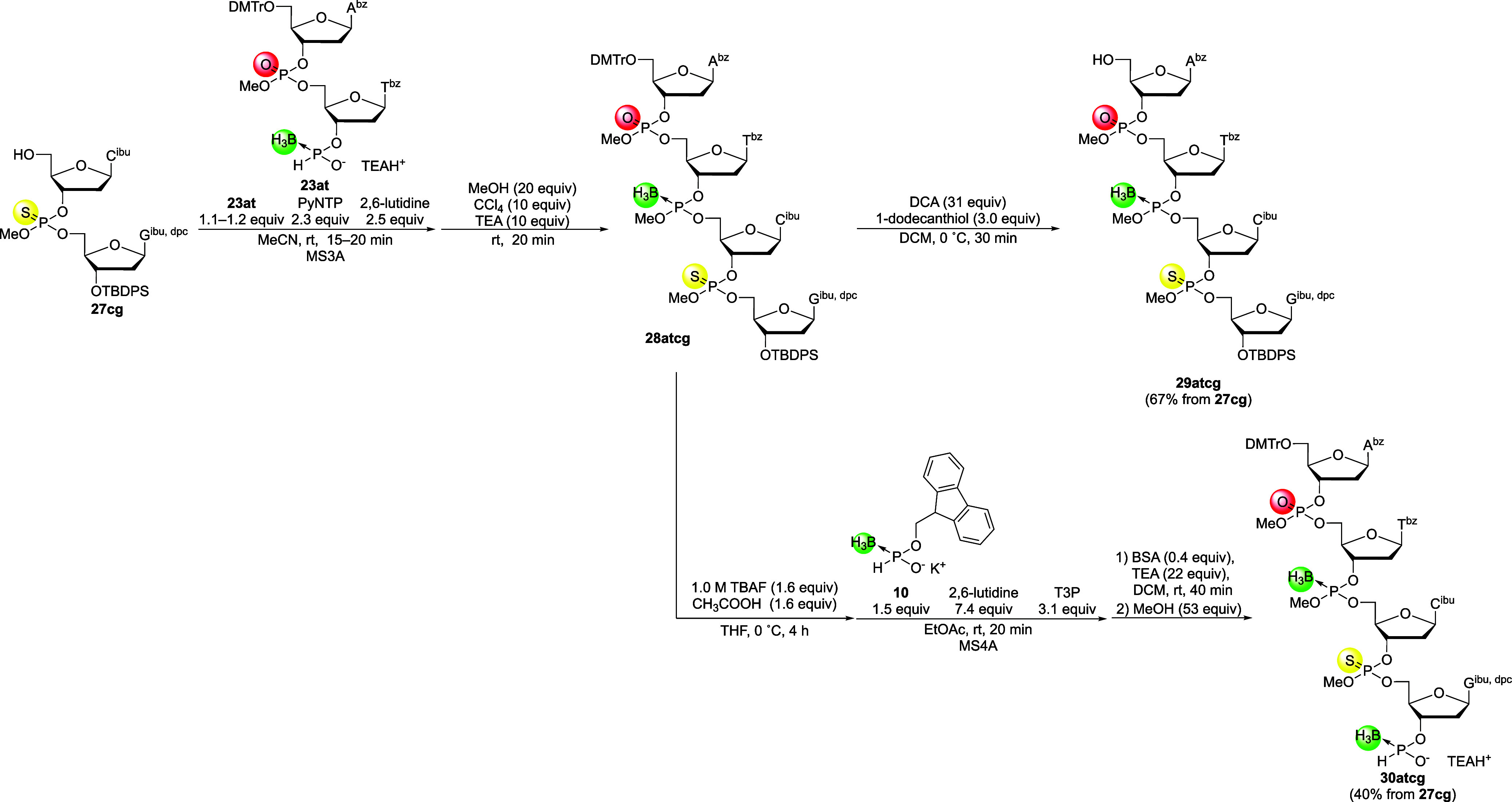
Preparation of 5′-Upstream and 3′-Downstream 4-Mer Building Blocks

### Chain Elongation Using 4-Mer Building Blocks, Deprotection, and Isolation of PB/PS/PO Chimeric ODNs

Next, chain elongation was conducted using the 5′-upstream and 3′-downstream 4-mer building blocks. First, the 5′-hydroxy group of compound **29atcg** and 1.5 equiv of compound **30atcg** bearing a 3′-*H*-boranophosphonate moiety were condensed using PyNTP, followed by oxidation of the resulting *H*-boranophosphonate diester into the corresponding boranophosphotriester and 5′-detritylation (Scheme 15). The progress of the reaction was confirmed by UHPLC, with almost complete consumption of starting material **29atcg** (Figure S14). After detritylation, the 8-mer was purified by silica gel column chromatography, which was also difficult due to extended elution tailing during purification, most likely caused by the presence of multiple diastereomers. As a result, purified product **32** contained minor byproducts, such as a triaminophosphine oxide derivative derived from excess PyNTP (Figure S16). Thus, we estimated the amount of compound **32** taking into contamination of the triaminophosphine oxide derivative (6.3 equiv), and we proceeded to the following block condensation reaction.

**Scheme 15:**
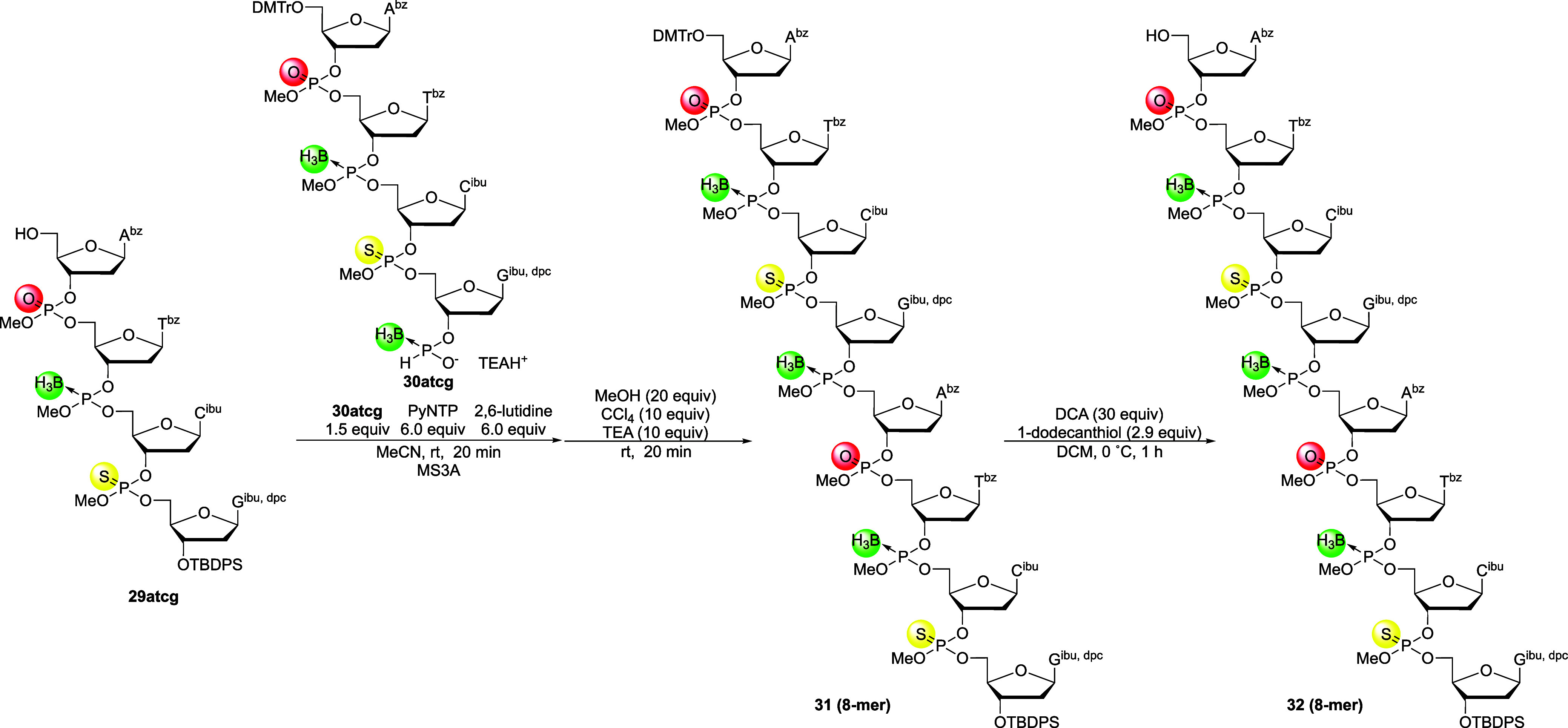
Chain Elongation Using PB/PS/PO 4-Mer Building Blocks

Next, the 3′-*H*-boranophosphonate monoester of compound **30atcg** was condensed with the 5′-hydroxy group of 8-mer **32**, followed by oxidative esterification of the resultant *H*-boranophosphonate diester to afford the corresponding boranophosphotriester (Scheme 16). The condensation between the 4-mer and 8-mer, followed by oxidative esterification, also proceeded efficiently, which was confirmed by UHPLC (Figure S17). Subsequently, all protecting groups were removed following the same procedure, that is, detritylation, removal of methyl and TBDPS groups, and deprotection of the nucleobases (Scheme 16). After these reactions, the crude mixture was analyzed by UPLC, which confirmed that the desired PB/PS/PO chimeric 12-mer was obtained as the main product (Figure S18). The PB/PS/PO chimeric 12-mer was then purified by ODS silica gel column chromatography, isolated in an acceptable yield (13%, nine steps from **29atcg**), and characterized by ESI-MS, ^1^H NMR, and ^31^P NMR analyses. Although isolation of the building block was difficult, the fully deprotected PB/PS/PO chimeric 12-mer (**33**) was efficiently synthesized in solution via conversion to stable boranophosphotriester, phosphorothioate triester, and phosphotriester linkages. Additionally, this solution-phase approach required only minimal excess of each building block (1.1–1.5 equiv per coupling step), highlighting its potential for efficient and scalable production of chimeric ODNs despite challenges in purification.

**Scheme 16:**
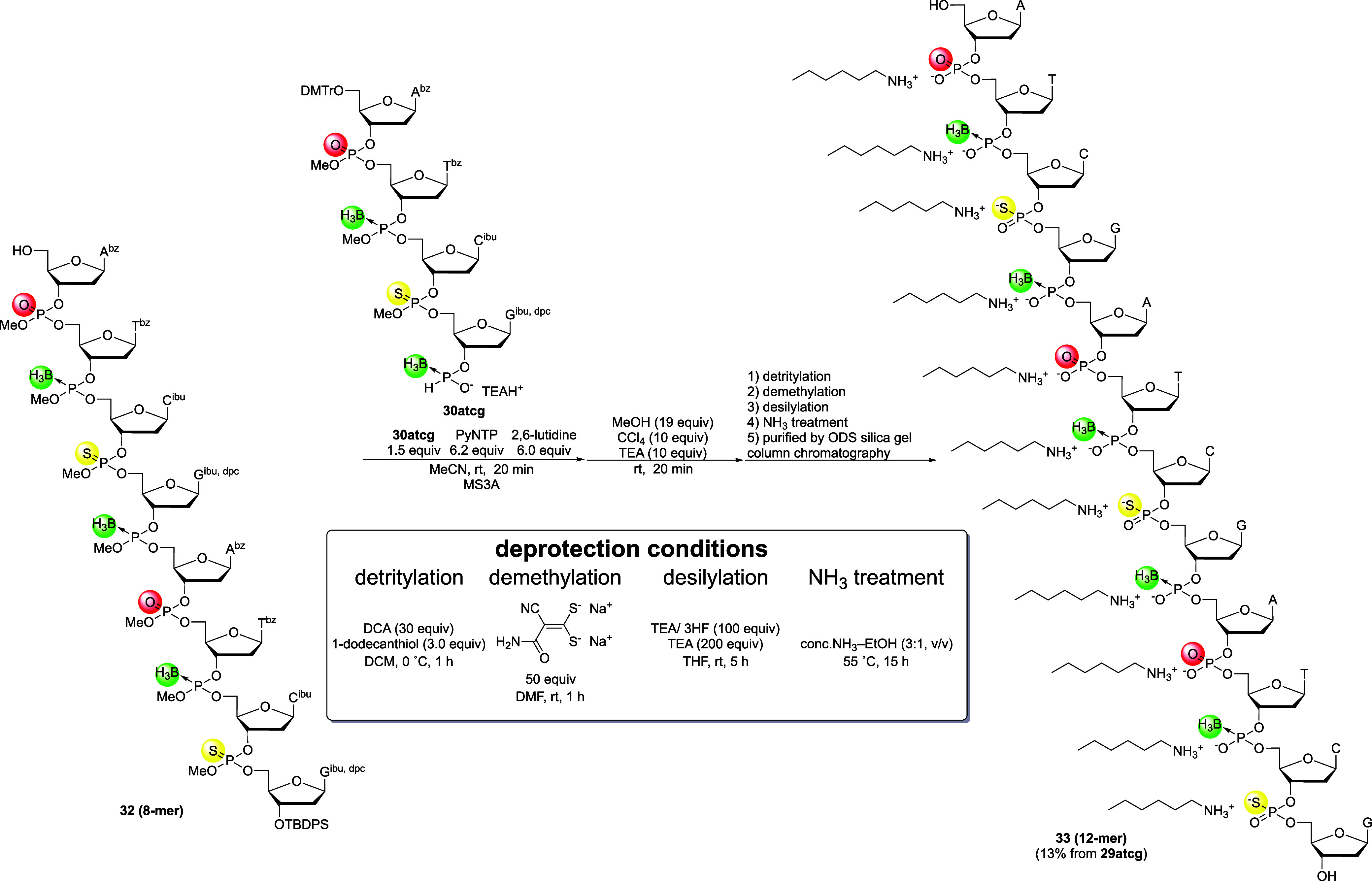
Block Condensation and Complete Removal of Protecting Groups for PB/PS/PO Chimeric 12-Mer

## Conclusion

We successfully synthesized PB-ODNs up to 12-mer in solution using the *H*-boranophosphonate method and a block condensation strategy using 4-mer building blocks. To avoid the decomposition of the internucleotidic linkages, an *H*-boranophosphodiester linkage, which was formed upon condensation of a *H*-boranophosphonate monoester and a hydroxy group, was converted to a stable boranophosphotriester via oxidative esterification. Although the oxidative esterification of the *H*-boranophosphonate diesters was expected to be challenging due to the competitive hydrolysis of the boranophosphorochloridate intermediates, the reaction proceeded efficiently without notable side reactions in a one-pot manner in the presence of excess PyNTP. Furthermore, the use of a newly developed *H*-boranophosphonylation reagent bearing an Fm group as a protecting group enabled the *H*-boranophosphonylation of the 3′-hydroxy groups in the 2-mer and 4-mer building blocks. The block condensation and oxidative esterification of the 2-mer and 4-mer building blocks also proceeded efficiently without hydrolysis of the intermediates, enabling the synthesis of the PB 12-mer. In addition, this strategy proved to be applicable to *H*-phosphonate and *H*-phosphonothioate diesters, enabling the synthesis of the PB/PS/PO chimeric 12-mer in solution. These strategies provide a new platform for the synthesis and scaling-up of PB-ODNs and PB/PS/PO chimeric ODNs as oligonucleotide therapeutics. Further improvement of the overall yield may be possible through optimization of protecting groups and purification. Future work will focus on investigating more robust internucleotidic protecting groups and exploring alternative purification techniques, such as size exclusion chromatography, to mitigate these challenges.

## Experimental Section

### General Information

All reactions were conducted under an Ar atmosphere. Dry organic solvents were prepared by the appropriate procedures. The deoxyadenosine *H*-phosphonate monomer as TEA salt (**19a**) was purchased from Chemgenes (ANP-3410). ^1^H NMR spectra were recorded at 500 MHz with tetramethylsilane (δ 0.00) as an internal standard in CDCl_3_ or with acetonitrile (MeCN) (δ 2.06) as an internal standard in D_2_O. ^13^C NMR spectra were recorded at 126 MHz with CDCl_3_ (δ 77.0) as an internal standard in CDCl_3_. ^31^P NMR spectra were recorded at 202 MHz with 85% H_3_PO_4_ (δ 0.0) as an external standard in CDCl_3_ or D_2_O. ^11^B NMR spectra were recorded at 160 MHz with 15% BF_3_·OEt_2_ in CDCl_3_ as an external standard in CDCl_3_ or D_2_O by using a quartz NMR tube. Structural assignments were made with additional information from gCOSY, gHSQC, and gHMBC experiments. Analytical thin-layer chromatography was performed on commercial glass plates with a 0.25 mm thickness silica gel layer. Automated silica gel column chromatography was performed on silica gel (Yamazen UNIVERSAL Premium column (30 μm 60 Å)) using automated flash chromatography system W-prep 2XY (Yamazen Corporation). The reaction mixture was analyzed using an ACQUITY Premier UPLC system (Waters) at 260 nm at a temperature of 60 °C and a flow rate of 0.5 mL/min using a C18 column (1.7 μm, 2.1 × 50 mm). Synthesized 12-mers were purified by ODS silica gel (Yamazen UNIVERSAL Premium column (30 μm 120 Å)) (Yamazen Corporation) using an automated flash chromatography system W-prep 2XY (Yamazen Corporation) and identified by electrospray ionization (ESI) mass spectroscopy, ^31^P NMR, and ^1^H NMR. Isolated yields were calculated by weighing the purified product.

### T_P(B)_A 2-Mer Building Block Bearing an *H*-Boranophosphonate Monoester at the 3′-End (**7ta**)

Compound **1a** ^40^ (599.2 mg, 1.0 mmol), compound **2t** ^29^ (919.1 mg, 1.1 mmol), and 2,6-lutidine (0.29 mL, 2.5 mmol) were dissolved in dry MeCN (10 mL) and dried over 3 Å molecular sieves. PyNTP (1.01 g, 2.0 mmol) was added to the mixture at rt while stirring. After the mixture was stirred for 20 min, MeOH (0.81 mL, 20 mmol) was added followed by adding a solution containing TEA (1.4 mL, 10 mmol) and CCl_4_ (1.0 mL, 10 mmol) at rt. The mixture was allowed to stir for a further 20 min. Then, the mixture was diluted with CHCl_3_ (100 mL) and washed with 1.0 M citrate buffer (pH 3) (100 mL). The aqueous layer was extracted with CHCl_3_ (50 mL). The organic layers were combined, dried over Na_2_SO_4_, filtered, and concentrated to dryness under reduced pressure. Then, the crude mixture was dissolved in dry THF (10 mL) and cooled to 0 °C. A mixture of a 1.0 M TBA fluoride (TBAF) THF solution (1.5 mL, 1.5 mmol) and acetic acid (86 μL, 1.5 mmol) was added to the solution at 0 °C, and the mixture was allowed to stir for 5 h. The mixture was warmed to rt, diluted with CH_2_Cl_2_ (100 mL), and washed with a saturated NaHCO_3_ solution (100 mL). The aqueous layer was extracted with CH_2_Cl_2_ (100 mL). The organic layers were combined, dried over Na_2_SO_4_, filtered, and concentrated under reduced pressure. The residue was purified by silica gel column chromatography. Column chromatography was carried out on a Yamazen UNIVERSAL Premium column (L size: 40 g silica gel, 30 μm, 3.0 × 16.5 cm) using an automated flash chromatography system W-prep 2XY (Yamazen Corporation), which was performed with an isocratic elution of EtOAc–MeOH (98:2, v/v) over 4 min, followed by a linear gradient of EtOAc–MeOH (98:2–72:28, v/v) over 20 min. Then, the fractions containing **5ta** were collected and concentrated under reduced pressure. Thereafter, the obtained compound **5ta** and boranophosphonylation reagent **10** (420.9 mg, 1.4 mmol) were dissolved in dry CH_2_Cl_2_ (20 mL), and 2,6-lutidine (0.77 mL, 6.6 mmol) was added to the reaction mixture. 50 wt % T3P in EtOAc (1.7 mL, 2.8 mmol) was added to the reaction mixture and allowed to stir for 30 min at rt. The residue was diluted with EtOAc (100 mL) and washed with saturated NaHCO_3_ solution (3 × 50 mL). The organic layers were dried over Na_2_SO_4_, filtered, and concentrated under reduced pressure. The residue was redissolved in dry CH_2_Cl_2_ (20 mL). TMSCl (0.15 mL, 1.2 mmol) was added to the mixture at rt. The mixture was allowed to stir for 10 min. TEA (2.8 mL, 20 mmol) was added to the mixture, and the mixture was stirred for 45 min, followed by adding MeOH (0.81 mL, 20 mmol) at rt. After it was stirred for 20 min, the mixture was concentrated by repeated coevaporation with toluene. The residue was purified by silica gel column chromatography. Column chromatography was carried out on a Yamazen UNIVERSAL Premium column (L size: 40 g silica gel, 30 μm, 3.0 × 16.5 cm) using the automated flash chromatography system W-prep 2XY (Yamazen Corporation), which was performed with an isocratic elution of EtOAc–MeOH–TEA (90:10:1, v/v/v) over 4 min followed by a linear gradient of EtOAc–MeOH–TEA (100:20:1–80:20:1, v/v/v) and with an isocratic elution of CH_2_Cl_2_–MeOH–TEA (80:20:1, v/v/v) over 10 min. Then, the fractions containing **7ta** were collected and concentrated under reduced pressure to afford **7ta** as a colorless foam and obtained as a mixture of a triethylammonium and TBA salt (1.02 g, 0.80 mmol, triethylammonium/TBA = 1:0.2, 80% from **1a**).

^1^H NMR (CDCl_3_, 500 MHz): δ 9.13 (br s, 0.5H, –CONH–), −9.08 (br s, 0.5H, −CONH−), 8.80–8.72 (m, 1H, H-2 of deoxyadenosine), 8.24 (s, 0.25H, H-8 of deoxyadenosine), 8.22 (s, 0.25H, H-8 of deoxyadenosine), 8.20 (s, 0.25H, H-8 of deoxyadenosine), 8.16 (s, 0.25H, H-8 of deoxyadenosine), 8.02–7.96 (m, 1H), 7.94–7.88 (m, 3H), 7.75–7.54 (m, 3.5H, P–H), 7.52–7.42 (m, 4H), 7.42–7.36 (m, 2H), 7.34–7.21 (m, 10H), 6.98–6.89 (br s, 0.5H, P–H), 6.88–6.80 (m, 4H), 6.56–6.48 (m, 1H, H-1′ of deoxyadenosine), 6.43–6.34 (m, 1H, H-1′ of thymidine), 5.27–5.17 (m, 1H, H-3′ of thymidine), 5.12–4.97 (m, 1H, H-3′ of deoxyadenosine), 4.45–4.38 (m, 1H, H-4′ of deoxyadenosine), 4.36–4.22 (m, 2.5H, H-5′ of deoxyadenosine and H-4′ of thymidine), 4.20–4.17 (m, 0.5H, H-4′ of thymidine), 3.79 (s, 3H, -OCH_3_ of DMTr), 3.78 (s, 3H, -OCH_3_ of DMTr), 3.65, 3.63, 3.60, 3.58 (4 × *d*, *J* = 11.2 Hz, 3H, −POCH_3_ of boranophosphotriester), 3.46–3.37 (m, 2H, H-5′ of thymidine), 3.25–3.19 (1.5H, m, −CH_2_– of TBA salt), 3.02 (q, *J* = 7.3 Hz, 6H, −CH_2_– of TEA), 2.96–2.85 (m, 1H, H-2′ of deoxyadenosine), 2.80–2.70 (m, 1H, H-2′ of deoxyadenosine), 2.63–2.54 (m, 1H, H-2′ of thymidine), 2.50–2.41 (m, 1H, H-2′ of thymidine), 1.67–1.58 (1.5H, m, −CH_2_– of TBA salt), 1.47–1.37 (m, 4.5H, 5-CH_3_ of thymidine and −CH_2_– of TBA salt), 1.29 (t, *J* = 7.3 Hz, 9H, −CH_3_ of TEA), 0.98 (t, *J* = 7.3 Hz, 9H, −CH_3_ of TBA salt), 0.9–0.1 (br, 6H, BH_3_); ^13^C­{^1^H} NMR (CDCl_3_, 126 MHz): δ 169.0, 168.9, 164.5, 162.6 (C-4 of thymidine), 158.7, 158.7, 152.5 (C-2 of adenosine), 151.4 (C-4 of deoxyadenosine), 149.5 (C-6 of deoxyadenosine), 149.2 (C-2 of thymidine), 144.0, 141.5, 141.5 (C-8 of deoxyadenosine), 141.4 (C-8 of deoxyadenosine), 135.1 (C-6 of thymidine), 135.1 (C-6 of thymidine), 135.0, 135.0, 134.9, 134.8, 133.5, 133.4, 132.6, 132.6, 131.4, 130.4, 129.9, 129.1, 128.7, 128.7, 128.1, 128.0, 127.9, 127.8, 127.7, 127.2, 123.4 (C-5 of deoxyadenosine), 123.4 (C-5 of deoxyadenosine), 113.3, 111.5 (C-5 of thymidine), 111.5 (C-5 of thymidine), 111.5 (C-5 of thymidine), 87.3, 87.2, 84.9 (C-4′ of thymidine), 84.6 (C-1′ of deoxyadenosine), 84.5 (C-1′ of deoxyadenosine), 84.5 (C-1′ of thymidine), 84.4 (C-1′ of thymidine), 84.2 (C-4′ of deoxyadenosine), 84.2 (C-4′ of deoxyadenosine), 84.2 (C-4′ of deoxyadenosine), 78.0 (C-3′ of thymidine), 74.6 (C-3′ of deoxyadenosine), 74.5 (C-3′ of deoxyadenosine), 74.4 (C-3′ of deoxyadenosine), 74.3 (C-3′ of deoxyadenosine), 65.8 (d, ^2^ *J*_C–P_ = 4.1 Hz, C-5′ of deoxyadenosine), 65.7 (d, ^2^ *J*_C–P_ = 4.4 Hz, C-5′ of deoxyadenosine), 65.5 (d, ^2^ *J*_C–P_ = 4.4 Hz, C-5′ of deoxyadenosine), 63.2 (C-5′ of thymidine), 63.2 (C-5′ of thymidine), 63.1 (C-5′ of thymidine), 55.2 (−OCH_3_ of DMTr), 53.7 (−POCH_3_ of boranophosphotriester), 53.6 (−POCH_3_ of boranophosphotriester), 53.6 (−POCH_3_ of boranophosphotriester), 53.5 (−POCH_3_ of boranophosphotriester), 45.3 (−CH_2_– of TEA), 39.6 (C-2′ of thymidine), 39.3 (C-2′ of thymidine), 39.3 (C-2′ of thymidine), 38.8 (d, ^3^ *J*_C–P_ = 2.7 Hz, C-2′ of deoxyadenosine), 38.6 (d, ^3^ *J*_C–P_ = 3.5 Hz, C-2′ of deoxyadenosine), 23.9 (−CH_2_– of TBA salt), 19.6 (−CH_2_– of TBA salt), 13.6 (−CH_3_ of TBA salt), 11.7 (5-CH_3_ of thymidine), 11.6 (5-CH_3_ of thymidine), 8.5 (−CH_3_ of TEA); ^31^P­{^1^H} NMR (CDCl_3_, 202 MHz): δ 120.2–116.9 (boranophosphotriester), 106.1–102.5 (H-boranophosphonate monoester); ^11^B {^1^H} NMR (CDCl_3_, 160 MHz): δ – 36–40 (H-boranophosphonate monoester), −43–49 (boranophosphotriester); HRMS (ESI–QTOF) *m*/*z*: [M–H]^−^ calcd for C_56_H_60_B_2_N_7_O_14_P_2_^–^ 1138.3865; found, 1138.3823.

### G_P(B)_C 2-Mer Building Block Bearing a 5′-OH Group (**12gc**)

Compound **1c** ^41^ (1.07 g, 2.0 mmol), compound **2g** (2.14 g, 2.1 mmol), and 2,6-lutidine (0.58 mL, 5.0 mmol) were dissolved in dry MeCN (20 mL) and dried over 3 Å molecular sieves. PyNTP (2.03 g, 4.1 mmol) was added to the mixture at room temperature while stirring. After the mixture was stirred for 15 min, MeOH (1.6 mL, 40 mmol) was added followed by adding a solution containing TEA (2.8 mL, 20 mmol) and CCl_4_ (1.9 mL, 20 mmol) at rt. The mixture was allowed to stir for a further 20 min. Then, the mixture was diluted with CHCl_3_ (150 mL) and washed with 1.0 M citrate buffer (pH 3) (3 × 100 mL). The combined aqueous layers were extracted with CHCl_3_ (3 × 50 mL). The organic layers were combined, dried over Na_2_SO_4_, filtered, and concentrated to dryness under reduced pressure. Then, the crude mixture was dissolved in dry CH_2_Cl_2_ (180 mL). After 1-dodecanethiol (1.4 mL, 5.9 mmol) was added to the mixture, the reaction mixture was cooled to 0 °C. Dichloroacetic acid (DCA) (4.9 mL, 30 mmol) in CH_2_Cl_2_ (20 mL) was added to the mixture at 0 °C while stirring. After the mixture was stirred for 1 h, 1-methylimidazole (6.3 mL, 80 mmol) was added to the mixture, and the mixture was warmed to room temperature while stirring for a further 10 min. Then, the mixture was diluted with CH_2_Cl_2_ (10 mL) and washed with saturated NaHCO_3_ aqueous solution (3 × 100 mL). The combined aqueous layers were extracted with CH_2_Cl_2_ (3 × 50 mL). The organic layers were combined, dried over Na_2_SO_4_, filtered, and concentrated to dryness under reduced pressure. The residue was purified by silica gel column chromatography. Column chromatography was carried out on a Yamazen UNIVERSAL Premium column (2L size: 54 g silica gel, 30 μm, 3.0 × 20.0 cm) using an automated flash chromatography system W-prep 2XY (Yamazen Corporation), which was performed with a linear gradient of EtOAc–hexane (50:50–100:0, v/v) over 10 min, followed by an isocratic elution of EtOAc for 5 min and a linear gradient of EtOAc–MeOH (100:0–80:20, v/v) over 19 min. Then, the fractions containing **12gc** were collected and concentrated under reduced pressure to afford **12gc** as a colorless foam (1.91 g, 1.7 mmol, 84% from **1c**).

^1^H NMR (CDCl_3_, 500 MHz): δ 9.07 (br s, 1H, −CONH-), 8.66 (br s, 0.5H, −CONH−), 8.48 (br s, 0.5H, −CONH−), 8.16 (s, 0.5H, H-8 of deoxyguanosine), 8.11 (s, 0.5H, H-8 of deoxyguanosine), 7.82 (d, *J* = 7.6 Hz, 0.5H, H-6 of deoxycytidine), 7.71 (d, *J* = 7.4 Hz, 0.5H, H-6 of deoxycytidine), 7.67–7.61 (m, 4H), 7.49–7.33 (m, 15H), 7.28–7.22 (m, 2H), 6.39 (t, *J* = 6.6 Hz, 0.5H, H-1′ of deoxycytidine), 6.32 (t, *J* = 6.4 Hz, 0.5H, H-1′ of deoxycytidine), 6.23 (dd, *J* = 8.2, 5.5 Hz, 0.5H, H-1′ of deoxyguanosine), 6.20 (dd, *J* = 8.6, 5.6 Hz, 0.5H, H-1′ of deoxyguanosine), 5.28–5.23 (m, 0.5H, H-3′ of deoxyguanosine), 5.21–5.15 (m, 0.5H, H-3′ of deoxyguanosine), 4.41–4.30 (m, 2H, H-3′ of deoxycytidine and 5′-OH), 4.18–4.15 (m, 1H, H-4′ of deoxyguanosine), 4.13–4.10 (m, 0.5H, H-4′ of deoxycytidine), 4.09–4.06 (m, 0.5H, H-4′ of deoxycytidine), 4.03–3.97 (m, 0.5H, H-5′ of deoxycytidine), 3.97–3.91 (m, 0.5H, H-5′ of deoxycytidine), 3.86–3.76 (m, 1H, H-5′ of deoxyguanosine), 3.75–3.58 (m, 5H, H-5′ of deoxyguanosine, H-5′ of deoxycytidine, and −POCH_3_ of boranophosphotriester), 3.15 (dd, *J* = 8.7, 5.7 Hz, 0.5H, H-2′ of deoxyguanosine), 3.12 (dd, *J* = 8.6, 5.7 Hz, 0.5H, H-2′ of deoxyguanosine), 3.1–3.0 (br, 0.5H, −CH­(CH_3_)_2_), 2.9–2.8 (br, 0.5H, −CH­(CH_3_)_2_), 2.69–2.52 (m, 2H, −CH­(CH_3_)_2_ and H-2′ of deoxycytidine), 2.42–2.34 (m, 1H, H-2′ of deoxyguanosine), 1.99–1.89 (m, 1H, H-2′ of deoxycytidine), 1.32–1.15 (m, 12H, –CH­(CH_3_)_2_), 1.08 (s, 4.5H, –C­(CH_3_)_3_ of TBDPS), 1.08 (s, 4.5H, –C­(CH_3_)_3_ of TBDPS), 0.80–0.10 (br s, 3H, BH_3_); ^13^C­{^1^H} NMR (CDCl_3_, 126 MHz): δ 177.4 (−CONH−), 177.2 (−CONH−), 175.8 (−CONH−), 162.9 (C-4 of deoxycytidine), 162.4 (C-4 of deoxycytidine), 156.4, 155.2 (C-2 of deoxycytidine), 155.1 (C-2 of deoxycytidine), 154.1 (C-4 of deoxyguanosine), 154.1 (C-4 of deoxyguanosine), 151.5, 150.2, 150.1, 144.0, 143.9, 143.8, 141.6, 135.6, 135.6, 132.9, 132.8, 132.6, 132.5, 130.3, 130.3, 130.2, 130.2, 129.2, 128.0, 128.0, 122.3 (C-5 of deoxyguanosine), 122.2 (C-5 of deoxyguanosine), 97.1 (C-5 of deoxycytidine), 96.5 (C-5 of deoxycytidine), 87.4 (d, ^3^ *J*_C–P_ = 3.0 Hz, C-4′ of deoxyguanosine), 87.3 (C-1′ of deoxycytidine), 87.2 (d, ^3^ *J*_C–P_ = 3.6 Hz, C-4′ of deoxyguanosine), 86.2 (C-1′ of deoxycytidine), 86.1 (C-1′ of deoxyguanosine), 85.5 (d, ^3^ *J*_C–P_ = 6.4 Hz, C-4′ of deoxycytidine), 85.1 (d, ^3^ *J*_C–P_ = 5.0 Hz, C-4′ of deoxycytidine), 78.5 (C-3′ of deoxyguanosine), 78.2 (d, ^2^ *J*_C–P_ = 2.8 Hz, C-3′ of deoxyguanosine), 72.4 (C-3′ of deoxycytidine), 72.1 (C-3′ of deoxycytidine), 65.3 (C-5′ of deoxycytidine), 65.2 (d, ^2^ *J*_C–P_ = 3.8 Hz, C-5′ of deoxycytidine), 62.3 (C-5′ of deoxyguanosine), 62.2 (C-5′ of deoxyguanosine), 53.8 (d, ^2^ *J*_C–P_ = 2.1 Hz, −POCH_3_ of boranophosphotriester), 53.8 (d, ^2^ *J*_C–P_ = 2.5 Hz, −POCH_3_ of boranophosphotriester), 41.4 (C-2′ of deoxycytidine), 40.9 (C-2′ of deoxycytidine), 38.7 (C-2′ of deoxyguanosine), 36.6 (−CH­(CH_3_)_2_), 36.6 (−CH­(CH_3_)_2_), 36.0 (−CH­(CH_3_)_2_), 35.2 (−CH­(CH_3_)_2_), 26.8 (−C­(CH_3_)_3_ of TBDPS), 19.4, 19.3, 19.2, 19.1, 19.0, 19.0, 18.8 (−CH­(CH_3_)_2_); ^31^P­{^1^H} NMR (CDCl_3_, 202 MHz): δ 121.0–118.3; ^11^B {^1^H} NMR (CDCl_3_, 160 MHz): δ – 43–49 (boranophosphotriester); HRMS (ESI–QTOF) *m*/*z*: [M + H]^+^ calcd for C_57_H_70_BN_9_O_12_PSi^+^ 1142.4738; found, 1142.4702.

### T_P(B)_A_P(B)_G_P(B)_C 4-Mer Building Block Bearing a 5′-OH Group (**14tagc**)

Compound **12gc** (225.0 mg, 0.20 mmol), compound **7ta** (286.7 mg, 0.23 mmol), and 2,6-lutidine (58 μL, 0.50 mmol) were dissolved in dry MeCN (2 mL) and dried over 3 Å molecular sieves. PyNTP (221.3 mg, 0.44 mmol) was added to the mixture at rt while stirring. After the mixture was stirred for 20 min, MeOH (0.16 mL, 3.9 mmol) was added, followed by adding a solution containing TEA (0.28 mL, 2.0 mmol) and CCl_4_ (0.19 mL, 2.0 mmol) at rt. The mixture was allowed to stir for a further 20 min. Then, the mixture was diluted with CHCl_3_ (20 mL) and washed with 1.0 M citrate buffers (pH 3) (3 × 10 mL). The combined aqueous layers were extracted with CHCl_3_ (3 × 10 mL). The organic layers were combined, dried over Na_2_SO_4_, filtered, and concentrated to dryness under reduced pressure. Then, the crude mixture was dissolved in dry CH_2_Cl_2_ (18 mL). After the addition of 1-dodecanethiol (0.14 mL, 0.59 mmol) to the mixture, the reaction mixture was cooled to 0 °C. DCA (0.50 mL, 6.1 mmol) in dry CH_2_Cl_2_ (2.0 mL) was added to the mixture at 0 °C while stirring. After the mixture was stirred for 30 min, EtOH (2 mL) was added to the mixture, and the mixture was warmed to room temperature stirring for a further 3 min. Then, the mixture was diluted with CH_2_Cl_2_ (2 mL) and washed with saturated NaHCO_3_ aqueous solutions (3 × 10 mL). The combined aqueous layers were extracted with CH_2_Cl_2_ (2 × 10 mL). The organic layers were combined, dried over Na_2_SO_4_, filtered, and concentrated to dryness under reduced pressure. The residue was purified by silica gel column chromatography. Column chromatography was carried out on a Yamazen UNIVERSAL Premium column (M size: 16 g silica gel, 30 μm, 2.3 × 12.3 cm) using an automated flash chromatography system W-prep 2XY (Yamazen Corporation), which was performed with an isocratic elution of EtOAc over 3 min followed by a linear gradient of EtOAc–MeOH (100:0–81:19, v/v) over 10 min and an isocratic elution of EtOAc–MeOH (81:19,/v/v) for 7 min. Then, the fractions containing **14tagc** were collected and concentrated under reduced pressure to afford **14tagc** as a colorless foam (326.7 mg, 0.16 mmol, 83% from **12gc**). ^1^H NMR (CDCl_3_, 500 MHz), ^13^C­{^1^H} NMR (CDCl_3_, 126 MHz), and ^31^P­{^1^H} NMR (CDCl_3_, 202 MHz) spectra are provided in the Supporting Information. HRMS (ESI–QTOF) *m*/*z*: [M + H]^+^ calcd for C_93_H_113_B_3_N_16_O_24_P_3_Si^+^ 1991.7369; found, 1991.7419.

### T_P(B)_A_P(B)_G_P(B)_C 4-Mer Building Block Bearing an *H*-Boranophosphonate Monoester at the 3′-End (**15tagc**)

Compound **12gc** (226.9 mg, 0.20 mmol), compound **7ta** (275.4 mg, 0.22 mmol), and 2,6-lutidine (58 μL, 0.50 mmol) were dissolved in dry MeCN (2.0 mL) and dried over 3 Å molecular sieves. PyNTP (225.8 mg, 0.45 mmol) was added to the mixture at rt while stirring. After the mixture was stirred for 15 min, MeOH (0.16 mL, 3.9 mmol) was added, followed by addition of a solution containing TEA (0.28 mL, 2.0 mmol) and CCl_4_ (0.19 mL, 2.0 mmol) at rt. The mixture was allowed to stir for a further 20 min. Then, the mixture was diluted with CHCl_3_ (50 mL) and washed with 1.0 M citrate buffer (pH 3) (3 × 30 mL). The combined aqueous layers were extracted with CHCl_3_ (1 × 50 mL). The organic layers were combined, dried over Na_2_SO_4_, filtered, and concentrated to dryness under reduced pressure. Then, the crude mixture was dissolved in dry THF (2.0 mL) and dried over 3 Å molecular sieves and cooled to 0 °C. A mixture of 1.0 M TBAF in THF solution (0.30 mL, 0.30 mmol) and acetic acid (17 μL, 0.30 mmol) was added to the solution at 0 °C and allowed to stir for 4 h at 0 °C. The mixture was diluted with CHCl_3_ (50 mL) and washed with saturated NaHCO_3_ solution (3 × 50 mL). The combined aqueous layers were extracted with CHCl_3_ (2 × 50 mL). The organic layers were combined, dried over Na_2_SO_4_, filtered, and concentrated under reduced pressure. The residue was purified by silica gel column chromatography. Column chromatography was carried out on a Yamazen UNIVERSAL Premium column (M size: 16 g silica gel, 30 μm, 2.3 × 12.3 cm) using an automated flash chromatography system W-prep 2XY (Yamazen Corporation), which was performed with an isocratic elution of EtOAc over 3 min followed by a linear gradient of EtOAc–MeOH (100:0–78:22, v/v) over 20 min. Then, the fractions were collected and concentrated under reduced pressure. Thereafter, boranophosphonylation reagent **10** (84.0 mg, 0.28 mmol) was added to the residue and dried by repeated coevaporation with CHCl_3_ and dissolved in dry EtOAc (4.0 mL). Then, 2,6-lutidine (0.15 mL, 1.3 mmol) was added to the reaction mixture. 50 wt % T3P in EtOAc (0.34 mL, 0.57 mmol) was added to the reaction mixture and allowed to stir for 20 min at rt. The mixture was diluted with EtOAc (20 mL) and washed with saturated NaHCO_3_ solution (3 × 10 mL). The organic layer was dried over Na_2_SO_4_, filtered, and concentrated under reduced pressure. The residue was dissolved in CH_2_Cl_2_ (4.0 mL). TEA (0.55 mL, 4.0 mmol) was added to the mixture at room temperature, and the mixture was stirred for 40 min. Then, the mixture was concentrated by repeated coevaporation with toluene. The residue was purified by silica gel column chromatography. Column chromatography was carried out on a Yamazen UNIVERSAL Premium column (M size: 16 g silica gel, 30 μm, 2.3 × 12.3 cm) using the automated flash chromatography system W-prep 2XY (Yamazen Corporation), which was performed with an isocratic elution of EtOAc–MeOH–TEA (95:5:1, v/v/v) over 3 min followed by a linear gradient of EtOAc–MeOH–TEA (95:5:1–90:10:1, v/v/v) over 14 min, and with an isocratic elution of EtOAc–MeOH–TEA (90:10:1, v/v/v) over 3 min followed by a linear gradient of CH_2_Cl_2_–MeOH–TEA (90:10:1–80:20:1, v/v/v) for 19 min and an isocratic elution of CH_2_Cl_2_–MeOH–TEA (80:20:1, v/v/v) for 10 min. Then, the fractions containing **15tagc** were collected and concentrated under reduced pressure to afford **15tagc** as a colorless foam (216.2 mg, 97 μmol, 49% from **12gc**). ^1^H NMR (CDCl_3_, 500 MHz), ^13^C­{^1^H} NMR (CDCl_3_, 126 MHz), ^31^P­{^1^H} NMR (CDCl_3_, 202 MHz) and ^11^B {^1^H} NMR (CDCl_3_, 160 MHz) spectra are provided in the Supporting Information. HRMS (ESI–QTOF) *m*/*z*: [M–H]^−^ calcd for C_98_H_115_B_4_N_16_O_27_P_4_^–^ 2115.7446; found, 2115.7290.

### T_P(B)_A_P(B)_G_P(B)_C_P(B)_T_P(B)_A_P(B)_G_P(B)_C 8-Mer Bearing a 5′-OH Group (**17**)

Compound **14tagc** (42.5 mg, 21 μmol), compound **15tagc** (67.7 mg, 30 μmol), and 2,6-lutidine (14 μL, 0.12 mmol) were dissolved in dry MeCN (0.40 mL) and dried over 3 Å molecular sieves. PyNTP (61.7 mg, 0.12 mmol) was added to the mixture at rt while stirring. After the mixture was stirred for 20 min, MeOH (16 μL, 0.40 mmol) was added, followed by addition of a solution containing TEA (28 μL, 0.20 mmol) and CCl_4_ (19 μL, 0.20 mmol). The mixture was allowed to stir for further 20 min. Then, the mixture was diluted with CHCl_3_ (10 mL) and washed with a 1.0 M citrate buffer (pH 3) (10 mL). The aqueous layer was extracted with CHCl_3_ (10 mL). The organic layers were combined, dried over Na_2_SO_4_, filtered, and concentrated to dryness under reduced pressure. Then, the crude mixture was dissolved in dry CH_2_Cl_2_ (2 mL). After the addition of 1-dodecanethiol (14 μL, 60 μmol) to the mixture, the reaction mixture was cooled to 0 °C. DCA (49 μL, 0.60 mmol) was added to the mixture at 0 °C while the mixture was stirring. After the mixture was stirred for 1 h at 0 °C, EtOH (10 μL) was added to the mixture, and the mixture was warmed to room temperature and stirred for a further 5 min. Then, the mixture was diluted with CH_2_Cl_2_ (5 mL) and washed with a saturated NaHCO_3_ aqueous solution (10 mL). The aqueous layer was extracted with CH_2_Cl_2_ (10 mL). The organic layers were combined, dried over Na_2_SO_4_, filtered, and concentrated to dryness under reduced pressure. The residue was purified by silica gel column chromatography. Column chromatography was carried out on a Yamazen UNIVERSAL Premium column (S size: 7 g silica gel, 30 μm, 1.8 × 11.4 cm) using an automated flash chromatography system W-prep 2XY (Yamazen Corporation), which was performed with an isocratic elution of EtOAc over 3 min, followed by a linear gradient of EtOAc–MeOH (100:0–70:30, v/v) over 20 min. Then, the fractions containing compound **17** were collected and concentrated under reduced pressure to afford compound **17** as a colorless foam (54.4 mg, 14 μmol). The entirety of obtained compound **17** (54.4 mg, 14 μmol) was utilized in the subsequent reaction without further purification.

### PB 12-Mer (Sequence: d­(T_PB_A_PB_G_PB_C_PB_T_PB_A_PB_G_PB_C_PB_T_PB_A_PB_G_PB_C)) (**18**)

Compound **17** (54.4 mg, 14 μmol), compound **15tagc** (43.0 mg, 19 μmol), and 2,6-lutidine (9.8 μL, 84 μmol) were dissolved in dry MeCN (0.28 mL) and dried over 3 Å molecular sieves. PyNTP (42.9 mg, 86 μmol) was added to the mixture at rt while stirring. After the mixture was stirred for 20 min, MeOH (11 μL, 0.27 mmol) was added followed by adding a solution containing TEA (19 μL, 0.14 mmol) and CCl_4_ (14 μL, 0.14 mmol) at rt. The mixture was allowed to stir for a further 20 min. Then, the mixture was diluted with CHCl_3_ (10 mL) and washed with a 1.0 M citrate buffer (pH 3) (10 mL). The aqueous layer was extracted with CHCl_3_ (10 mL). The organic layers were combined, dried over Na_2_SO_4_, filtered, and concentrated to dryness under reduced pressure. Then, the crude mixture was dissolved in dry CH_2_Cl_2_ (1.4 mL). After addition of 1-dodecanethiol (10 μL, 42 μmol) to the mixture, the reaction mixture was cooled to 0 °C. DCA (34 μL, 0.42 mmol) was added to the mixture at 0 °C while the mixture was stirring. After the mixture was stirred for 1 h, EtOH (20 μL) was added to the mixture, and the mixture was warmed to room temperature and stirred for a further 5 min. Then, the mixture was diluted with CH_2_Cl_2_ (5 mL) and washed with a saturated NaHCO_3_ aqueous solution (10 mL). The aqueous layer was extracted with CH_2_Cl_2_ (5 mL). The organic layers were combined, dried over Na_2_SO_4_, filtered, and concentrated to dryness under reduced pressure. Thereafter, the mixture was dissolved in dry DMF (1.4 mL). 2-Carbamoyl-2-cyanoethylene-1,1-dithiolate^42^ (140.9 mg, 0.69 mmol) was added to the reaction mixture and stirred for 1 h at rt. The reaction mixture was concentrated to dryness under reduced pressure, diluted with EtOAc (10 mL), and washed with 1.0 M TEAB buffer (pH 7, 10 mL). The aqueous layer was extracted with CHCl_3_ (3 × 10 mL). The organic layers were combined and concentrated to dryness under reduced pressure. The residue was dissolved in dry THF (1.4 mL). A solution containing TEA/3HF (0.23 mL, 1.4 mmol) and TEA (0.39 mL, 2.8 mmol) was added to the reaction mixture, and the mixture was allowed to stir for a further 5 h at rt. Then, the reaction mixture was concentrated under reduced pressure, followed by addition of concentrated NH_3_aq–EtOH (3:1, v/v) (5 mL). The reaction mixture was warmed to 55 °C using a magnetic stirrer with a hot plate (EYELA, Model: RCH-1000) and stirred for 15 h. Then, the reaction mixture was cooled to rt, diluted with H_2_O (20 mL), and washed with Et_2_O (3 × 10 mL). The aqueous layer was concentrated to dryness under reduced pressure to obtain a crude mixture (173.4 mg). A part of the residue (57.1 mg) was purified by ODS silica gel column chromatography. Column chromatography was carried out on ODS silica gel (Yamazen UNIVERSAL Premium column (30 μm 120 Å) (S size: 7 g silica gel, 30 μm, 1.8 × 11.4 cm)) using the automated flash chromatography system W-prep 2XY (Yamazen Corporation), which was performed with an isocratic elution of solution A (50 mM hexafluoroisopropanol (HFIP) and 5 mM hexylamine in H_2_O)–solution B (50 mM HFIP and 5 mM hexylamine in H_2_O–MeCN, 1:1, v/v) (90:10, v/v) over 2 min followed by a linear gradient of solution A–solution B (90:10–15:85, v/v) for 40 min. Then, the fractions containing **18** were collected and concentrated under reduced pressure to afford **18** as a colorless solid (2.0 mg, 0.46 μmol, 6% from **14tagc**). ^1^H NMR (D_2_O, 500 MHz), ^13^C­{^1^H} NMR (D_2_O, 126 MHz), ^31^P­{^1^H} NMR (D_2_O, 202 MHz) and ^11^B {^1^H} NMR (D_2_O, 160 MHz) spectra are provided in the Supporting Information. HRMS (ESI–QTOF) *m*/*z*: [M–4H]^4–^ calcd for C_117_H_177_B_11_N_45_O_59_P_11_^4–^904.2625; found, 904.2532.

### A_P(O)_T 2-Mer Building Block Bearing *H*-Boranophosphonate Monoester at the 3′-End (**23at**)

Compound **1t** (587.2 mg, 1.0 mmol), compound **19a** (974.8 mg, 1.1 mmol), and 2,6-lutidine (290 μL, 2.5 mmol) were dissolved in dry MeCN (10 mL) and dried over 3 Å molecular sieves. PyNTP (0.99 g, 2.0 mmol) was added to the mixture at rt while stirring. After the mixture was stirred for 20 min, MeOH (0.81 mL, 20 mmol) was added, followed by adding a solution containing TEA (1.40 mL, 10 mmol) and CCl_4_ (0.96 mL, 10 mmol). The mixture was allowed to stir for a further 20 min. Then, the mixture was diluted with CHCl_3_ (100 mL) and washed with 1.0 M citrate buffer (pH 3) (100 mL). The aqueous layer was extracted with CHCl_3_ (100 mL). The organic layers were combined, dried over Na_2_SO_4_, filtered, and concentrated to dryness under reduced pressure. Then, the crude mixture was dissolved in dry THF (10 mL) and cooled to 0 °C. A mixture of a 1.0 M TBAF THF solution (1.5 mL, 1.5 mmol) and acetic acid (86 μL, 1.5 mmol) was added to the solution at 0 °C and allowed to stir for 5 h. The mixture was warmed to rt, diluted with CH_2_Cl_2_ (100 mL), and washed with saturated NaHCO_3_ solution (100 mL). The aqueous layer was extracted with CH_2_Cl_2_ (100 mL). The organic layers were combined, dried over Na_2_SO_4_, filtered, and concentrated under reduced pressure. The residue was purified by silica gel column chromatography. Column chromatography was carried out on a Yamazen UNIVERSAL Premium column (L size: 40 g silica gel, 30 μm, 3.0 × 16.5 cm) using an automated flash chromatography system W-prep 2XY (Yamazen Corporation), which was performed with an isocratic elution of EtOAc–MeOH (98:2, v/v) over 4 min, followed by a linear gradient of EtOAc–MeOH (98:2–72:28, v/v) over 20 min. Then, the fractions containing **22at** were collected and concentrated under reduced pressure. Thereafter, the obtained compound **22at** and boranophosphonylation reagent **10** (420.9 mg, 1.4 mmol) were dissolved in dry CH_2_Cl_2_ (20 mL), and 2,6-lutidine (0.77 mL, 6.6 mmol) was added to the reaction mixture. 50 wt % T3P in EtOAc (1.7 mL, 2.9 mmol) was added to the reaction mixture and allowed to stir for 20 min at rt. The residue was diluted with EtOAc (100 mL) and washed with saturated NaHCO_3_ solution (3 × 50 mL). The organic layers were combined, dried over Na_2_SO_4_, filtered, and concentrated under reduced pressure. The residue was redissolved in CH_2_Cl_2_ (20 mL). TMSCl (0.15 mL, 1.2 mmol) was added to the mixture at rt. The mixture was allowed to stir for a further 10 min. TEA (2.8 mL, 20 mmol) was added to the mixture, and the mixture was stirred for 45 min, followed by addition of MeOH (0.81 mL, 20 mmol). After being stirred for 15 min, the mixture was concentrated by repeated coevaporation with toluene. The residue was purified by silica gel column chromatography. Column chromatography was carried out on a Yamazen UNIVERSAL Premium column (L size: 40 g silica gel, 30 μm, 3.0 × 16.5 cm) using the automated flash chromatography system W-prep 2XY (Yamazen Corporation), which was performed with an isocratic elution of EtOAc–MeOH–TEA (90:10:1, v/v/v) over 4 min followed by a linear gradient of EtOAc–MeOH–TEA (90:10:1–80:20:1, v/v/v) over 20 min, with an isocratic elution of CH_2_Cl_2_–MeOH–TEA (80:20:1, v/v/v) over 10 min. Then, the fractions containing **23at** were collected and concentrated under reduced pressure to afford **23at** as a colorless foam (766.0 mg, 0.59 mmol, 59% yield from **1t**).

^1^H NMR (CDCl_3_, 500 MHz): δ 9.22 (br s, 1H, −CONH-), 8.70–8.62 (m, 1H, H-2 of deoxyadenosine), 8.19 (s, H-8 of deoxyadenosine), 8.05–7.98 (m, 2H), 7.94–7.87 (m, 2H), 7.70–7.43 (m, 7.5H), 7.42–7.35 (m, 2H), 7.31–7.18 (m, 7H), 6.93–6.84 (m, 0.5H, P–H), 6.84–6.75 (m, 4H), 6.58–6.48 (m, 1H, H-1′ of deoxyadenosine), 6.40–6.26 (m, 1H, H-1′ of thymidine), 5.35–5.26 (m, 1H, H-3′ of deoxyadenosine), 4.97–4.86 (m, 0.5H, H-3′ of thymidine), 4.86–4.76 (m, 0.5H, H-3′ of thymidine), 4.52–4.26 (m, 4H, H-4′ of thymidine, H-4′ of deoxyadenosine, and H-5′ of thymidine), 3.86–3.74 (m, 9H, −OCH_3_ of DMTr and −OCH_3_ of phosphotriester), 3.52–3.38 (m, 2H, H-5′ of deoxyadenosine), 3.26–3.14 (m, 1H, H-2′ of deoxyadenosine), 3.02 (q, *J* = 7.3 Hz, 6H, −CH_2_– of TEA), 2.87–2.76 (m, 1H, H-2′ of deoxyadenosine), 2.66–2.54 (m, 1H, H-2′ of thymidine), 2.32–2.20 (m, 1H, H-2′ of thymidine), 2.01–1.93 (m, 3H, CH_3_ of thymidine), 1.30 (t, *J* = 7.3 Hz, 9H, −CH_3_ of TEA), 1.0–0.1 (br, 3H, BH_3_); ^13^C­{^1^H} NMR (CDCl_3_, 126 MHz): δ 168.9, 168.9, 164.5, 162.6 (C-4 of thymidine), 158.4, 152.3 (C-2 of deoxyadenosine), 151.3 (C-4 of deoxyadenosine), 149.4 (C-6 of deoxyadenosine), 149.1 (C-2 of thymidine), 149.1 (C-2 of thymidine), 149.0 (C-2 of thymidine), 144.1, 141.8 (C-8 of deoxyadenosine), 141.7 (C-8 of deoxyadenosine), 135.4, 135.3, 135.3, 135.2, 135.2, 135.1, 134.9, 133.4, 132.6, 131.3, 130.3, 129.8, 129.8, 129.0, 128.7, 127.9, 127.7, 127.7, 126.9, 123.5 (C-5 of deoxyadenosine), 123.4 (C-5 of deoxyadenosine), 113.0, 111.2 (C-5 of thymidine), 111.2 (C-5 of thymidine), 86.6, 86.6, 85.4 (C-1′ of thymidine), 85.1 (d, ^3^ *J*_C–P_ = 4.6 Hz, C-4′ of deoxyadenosine), 84.9 (d, ^3^ *J*_C–P_ = 5.7 Hz, C-4′ of deoxyadenosine), 84.9 (d, ^3^ *J*_C–P_ = 6.2 Hz, C-4′ of deoxyadenosine), 84.6 (C-1′ of deoxyadenosine), 84.6 (C-1′ of deoxyadenosine), 84.0 (C-4′ of thymidine), 84.0 (C-4′ of thymidine), 83.9 (C-4′ of thymidine), 83.9 (C-4′ of thymidine), 79.1 (d, ^2^ *J*_C–P_ = 5.2 Hz, C-3′ of deoxyadenosine), 78.9 (d, ^2^ *J*_C–P_ = 4.8 Hz, C-3′ of deoxyadenosine), 75.0 (d, ^2^ *J*_C–P_ = 6.9 Hz, C-3′ of thymidine), 73.9 (C-3′ of thymidine), 67.2 (d, ^2^ *J*_C–P_ = 4.6 Hz, C-5′ of thymidine), 67.0 (d, ^2^ *J*_C–P_ = 6.5 Hz, C-5′ of thymidine), 66.9 (d, ^2^ *J*_C–P_ = 5.6 Hz, C-5′ of thymidine), 63.0 (C-5′ of deoxyadenosine), 62.9 (C-5′ of deoxyadenosine), 55.1 (−OCH_3_ of DMTr), 54.7 (−OCH_3_ of phosphotriester), 54.7 (-OCH_3_ of phosphotriester), 45.4 (−CH_2_– of TEA), 39.6 (C-2′ of thymidine), 39.4 (C-2′ of thymidine), 39.3 (C-2′ of thymidine), 39.1 (C-2′ of thymidine), 38.0 (C-2′ of deoxyadenosine), 37.8 (C-2′ of deoxyadenosine), 12.3 (5-CH_3_ of thymidine), 8.4 (−CH_3_- of TEA); ^31^P­{^1^H} NMR (CDCl_3_, 202 MHz): δ 109.0–101.8 (H-boranophosphonate monoester), 0.1, −0.4 (phosphotriester); HRMS (ESI–QTOF) *m*/*z*: [M–H]^−^ calcd for C_56_H_57_BN_7_O_15_P_2_^–^ 1140.3486; found, 1140.3446.

### C_P(S)_G 2-Mer Building Block Bearing a 5′-OH Group (**27cg**)

Compound **1g** (1.54 g, 2.0 mmol), compound **24c** (1.72 g, 2.2 mmol), and 2,6-lutidine (0.58 mL, 5.0 mmol) were dissolved in dry MeCN (20 mL) and dried over 3 Å molecular sieves. PyNTP (1.97 g, 3.9 mmol) was added to the mixture while stirring. After the mixture was stirred for 15 min, MeOH (1.6 mL, 40 mmol) was added, followed by adding a solution containing TEA (2.8 mL, 20 mmol) and CCl_4_ (1.9 mL, 20 mmol) at rt. The mixture was allowed to stir for a further 20 min. Then, the mixture was diluted with CHCl_3_ (150 mL) and washed with 1.0 M citrate buffers (pH 3) (3 × 100 mL). The combined aqueous layers were extracted with CHCl_3_ (3 × 50 mL). The organic layers were combined, dried over Na_2_SO_4_, filtered, and concentrated to dryness under reduced pressure. Then, the crude mixture was dissolved in dry CH_2_Cl_2_ (180 mL). After adding 1-dodecanethiol (1.4 mL, 5.9 mmol) to the mixture, the reaction mixture was cooled to 0 °C. DCA (2.5 mL, 30 mmol) in CH_2_Cl_2_ (20 mL) was added to the mixture at 0 °C while stirring. After the mixture was stirred for 50 min, 1-methylimidazole (6.3 mL, 80 mmol) was added to the mixture, and the mixture was warmed to rt and stirred for a further 20 min. Then, the mixture was diluted with CH_2_Cl_2_ (10 mL) and washed with saturated NaHCO_3_ aqueous solutions (3 × 100 mL). The combined aqueous layers were extracted with CH_2_Cl_2_ (2 × 50 mL). The organic layers were combined, dried over Na_2_SO_4_, filtered, and concentrated to dryness under reduced pressure. The residue was purified by silica gel column chromatography. Column chromatography was carried out on a Yamazen UNIVERSAL Premium column (2L size: 54 g silica gel, 30 μm, 3.0 × 20.0 cm) using an automated flash chromatography system W-prep 2XY (Yamazen Corporation), which was performed with an isocratic elution of EtOAc–hexane (67:33, v/v) over 4 min followed by a linear gradient of EtOAc–hexane (67:33–100:0, v/v) over 25 min, an isocratic elution of EtOAc for 5 min, and a linear gradient of EtOAc–MeOH (100:0–90:10, v/v) over 20 min. Then, the fractions containing **27cg** were collected and concentrated under reduced pressure to afford **27cg** as a pale-yellow foam (2.05 g, 1.8 mmol, 89% from **1g**).

^1^H NMR (CDCl_3_, 500 MHz): δ 8.30–8.03 (m, 4H, −CONH- of deoxycytidine and deoxyguanosine, H-8 of deoxyguanosine, and H-6 of deoxycytidine), 7.69–7.63 (m, 4H), 7.51–7.30 (m, 15H), 7.28–7.23 (m, 2H), 6.49–6.44 (m, 1H, H-1′ of deoxyguanosine), 6.15 (dd, *J* = 7.9, 5.8 Hz, 0.5H, H-1′ of deoxycytidine), 6.10 (t, *J* = 6.4 Hz, 0.5H, H-1′ of deoxycytidine), 5.05–5.00 (m, 0.5H, H-3′ of deoxycytidine), 4.72–4.66 (m, 0.5H, H-3′ of deoxycytidine), 4.62–4.58 (m, 1H, H-3′ of deoxyguanosine), 4.23–4.19 (m, 0.5H, H-4′ of deoxyguanosine), 4.18–4.14 (m, 1H, H-4′ of deoxyguanosine and H-4′ of deoxycytidine), 4.13–4.08 (m, 0.5H, H-5′ of deoxyguanosine), 4.01–3.98 (m, 0.5H, H-4′ of deoxycytidine), 3.91–3.84 (m, 1H, H-5′ of deoxyguanosine), 3.83–3.74 (m, 0.5H, H-5′ of deoxyguanosine), 3.66–3.50 (m, 5H, H-5′ of deoxycytidine and -POCH_3_ of phosphorothioate triester), 3.02–2.89 (m, 1H, −CH­(CH_3_)_2_), 2.80–2.72 (m, 0.5H), 2.67–2.54 (m, 2H), 2.53–2.41 (m, 1.5H), 2.33–2.26 (m, 0.5H), 1.94–1.86 (m, 0.5H), 1.27–1.20 (m, 12H, –CH­(CH_3_)_2,_ of deoxyguanosine and deoxycytidine), 1.12 (s, 4.5H, –C­(CH_3_)_3_ of TBDPS), 1.11 (s, 4.5H, –C­(CH_3_)_3_ of TBDPS); ^13^C­{^1^H} NMR (CDCl_3_, 126 MHz): δ 176.6 (−CONH−), 176.6 (−CONH−), 175.7 (−CONH−), 162.2 (C-4 of deoxycytidine), 162.1 (C-4 of deoxycytidine), 155.9 155.2 (C-2 of deoxycytidine), 155.1 (C-2 of deoxycytidine), 154.5 (C-4 of deoxyguanosine), 154.3 (C-4 of deoxyguanosine), 152.0, 151.9, 150.9, 150.5, 145.5 (C-6 of deoxycytidine), 145.2 (C-6 of deoxycytidine), 143.0 (C-8 of deoxyguanosine), 142.7 (C-8 of deoxyguanosine), 141.6, 135.7, 135.6, 132.8, 132.8, 132.7, 130.3, 130.2, 129.2, 128.1, 128.0, 128.0, 128.0, 121.5 (C-5 of deoxyguanosine), 121.3 (C-5 of deoxyguanosine), 96.4 (C-5 of deoxycytidine), 96.3 (C-5 of deoxycytidine), 87.8 (C-1′ of deoxycytidine), 87.7 (C-1′ of deoxycytidine), 86.4 (d, ^3^ *J*_C–P_ = 3.8 Hz), 86.0 (d, ^3^ *J*_C–P_ = 5.1 Hz), 85.9 (d, ^3^ *J*_C–P_ = 6.7 Hz), 85.8 (d, ^3^ *J*_C–P_ = 8.9 Hz), 85.1 (C-1′ of deoxyguanosine), 84.9 (C-1′ of deoxyguanosine), 78.9 (d, ^3^ *J*_C–P_ = 4.6 Hz, C-3′ of deoxycytidine), 77.5 (d, ^3^ *J*_C–P_ = 4.4 Hz, C-3′ of deoxycytidine), 73.3 (C-3′ of deoxyguanosine), 73.3 (C-3′ of deoxyguanosine), 67.4 (d, ^2^ *J*_C–P_ = 6.4 Hz, C-5′ of deoxyguanosine), 66.9 (d, ^2^ *J*_C–P_ = 4.7 Hz, C-5′ of deoxyguanosine), 61.6 (C-5′ of deoxycytidine), 61.1 (C-5′ of deoxycytidine), 54.7 (d, ^2^ *J*_C–P_ = 4.9 Hz, −OCH_3_ of phosphorothioate triester), 54.6 (d, ^2^ *J*_C–P_ = 5.3 Hz, −OCH_3_ of phosphorothioate triester), 40.2 (C-2′ of deoxyguanosine), 39.6 (C-2′ of deoxyguanosine), 39.6 (d, ^3^ *J*_C–P_ = 5.6 Hz, C-2′ of deoxycytidine), 39.4 (d, ^3^ *J*_C–P_ = 3.8 Hz, C-2′ of deoxycytidine), 36.8 (−CH­(CH_3_)_2_), 35.8 (−CH­(CH_3_)_2_), 26.9 (−C­(CH_3_)_3_ of TBDPS), 19.2 (−CH­(CH_3_)_2_), 19.0 (−C­(CH_3_)_3_ of TBDPS); ^31^P­{^1^H} NMR (CDCl_3_, 202 MHz): δ 69.8, 68.6; HRMS (ESI–QTOF) *m*/*z*: [M + H]^+^ calcd for C_57_H_67_N_9_O_12_PSSi^+^ 1160.4131; found, 1160.4133.

### A_P(O)_T_P(B)_C_P(S)_G 4-Mer Building Block Bearing a 5′-OH Group (**29atcg**)

Compound **27cg** (229.6 mg, 0.20 mmol), compound **23at** (293.6 mg, 0.23 mmol), and 2,6-lutidine (58 μL, 0.50 mmol) were dissolved in dry MeCN (2.0 mL) and dried over 3 Å molecular sieves. PyNTP (224.3 mg, 0.45 mmol) was added to the mixture at rt while stirring. After the mixture was stirred for 20 min, MeOH (0.16 mL, 3.9 mmol) was added, followed by adding a solution containing TEA (0.28 mL, 2.0 mmol) and CCl_4_ (0.19 mL, 2.0 mmol) at rt. The mixture was allowed to stir for a further 20 min. Then, the mixture was diluted with CHCl_3_ (20 mL) and washed with 1.0 M citrate buffers (pH 3) (3 × 10 mL). The combined aqueous layers were extracted with CHCl_3_ (3 × 10 mL). The organic layers were combined, dried over Na_2_SO_4_, filtered, and concentrated to dryness under reduced pressure. Then, the crude mixture was dissolved in dry CH_2_Cl_2_ (18 mL). After addition of 1-dodecanethiol (0.14 mL, 0.60 mmol) to the mixture, the reaction mixture was cooled to 0 °C. DCA (0.50 mL, 6.1 mmol) in CH_2_Cl_2_ (2.0 mL) was added to the mixture at 0 °C while stirring. After the mixture was stirred for 30 min, EtOH (2 mL) was added to the mixture, and the mixture was warmed to room temperature and was stirred for a further 2 min. Then, the mixture was diluted with CH_2_Cl_2_ (2 mL) and washed with saturated NaHCO_3_ aqueous solutions (3 × 10 mL). The combined aqueous layers were extracted with CH_2_Cl_2_ (2 × 10 mL). The organic layers were combined, dried over Na_2_SO_4_, filtered, and concentrated to dryness under reduced pressure. The residue was purified by silica gel column chromatography. Column chromatography was carried out on a Yamazen UNIVERSAL Premium column (M size: 16 g silica gel, 30 μm, 2.3 × 12.3 cm) using an automated flash chromatography system W-prep 2XY (Yamazen Corporation), which was performed with an isocratic elution of EtOAc over 3 min followed by a linear gradient of EtOAc–MeOH (100:0–81:19, v/v) over 10 min and with an isocratic elution of EtOAc–MeOH (81:19, v/v) for 7 min. Then, the fractions containing **29atcg** were collected and concentrated under reduced pressure to afford **29atcg** as a colorless foam (268.0 mg, 0.13 mmol, 67% from **27cg**). ^1^H NMR (CDCl_3_, 500 MHz), ^13^C­{^1^H} NMR (CDCl_3_, 126 MHz), and ^31^P­{^1^H} NMR (CDCl_3_, 202 MHz) are provided in the Supporting Information. HRMS (ESI–QTOF) *m*/*z*: [M + H]^+^ Calcd for C_93_H_107_BN_16_O_25_P_3_SSi^+^ 2011.6384; Found 2011.6437.

### A_P(O)_T_P(B)_C_P(S)_G 4-Mer Building Block Bearing *H*-Boranophosphonate Monoester on 3′-OH (**30atcg**)

Compound **27cg** (455.2 mg, 0.39 mmol), compound **23at** (569.5 mg, 0.44 mmol), and 2,6-lutidine (116 μL, 1.0 mmol) were dissolved in dry MeCN (4.0 mL) and dried over 3 Å molecular sieves. PyNTP (446.2 g, 0.89 mmol) was added to the mixture at room temperature while stirring. After the mixture was stirred for 15 min, MeOH (0.33 mL, 8.1 mmol) was added, followed by adding a solution containing TEA (0.55 mL, 4.0 mmol) and CCl_4_ (0.39 mL, 4.0 mmol) at rt. The mixture was allowed to stir for further 20 min. Then, the mixture was diluted with CHCl_3_ (50 mL) and washed with 1.0 M citrate buffers (pH 3) (3 × 30 mL). The combined aqueous layers were extracted with CHCl_3_ (1 × 50 mL). The organic layers were combined, dried over Na_2_SO_4_, filtered, and concentrated to dryness under reduced pressure to give a crude mixture (1.22 g). Then, a part of the crude mixture (0.579 g) was dissolved in dry THF (10 mL), dried over 3 Å molecular sieves, and cooled to 0 °C. A mixture of 1.0 M TBAF in THF solution (0.30 mL, 0.30 mmol) and acetic acid (17 μL, 0.30 mmol) was added to the solution at 0 °C and allowed to stir for 4 h. The residue was diluted with CH_2_Cl_2_ (50 mL) and washed with saturated NaHCO_3_ solutions (3 × 50 mL). The combined aqueous layers were extracted with CH_2_Cl_2_ (2 × 50 mL). The organic layers were combined, dried over Na_2_SO_4_, filtered, and concentrated under reduced pressure. Column chromatography was carried out on a Yamazen UNIVERSAL Premium column (M size: 16 g silica gel, 30 μm, 2.3 × 12.3 cm) using an automated flash chromatography system W-prep 2XY (Yamazen Corporation), which was performed with an isocratic elution of EtOAc over 3 min, followed by a linear gradient of EtOAc–MeOH (100:0–80:20, v/v) over 28 min. Then, the fractions were collected and concentrated under reduced pressure. Thereafter, H-boranophosphonylation reagent **10** (83.2 mg, 0.28 mmol) was added to the residue, and the mixture was dried by repeated coevaporation with CHCl_3_ and dissolved in dry EtOAc (4.0 mL). 2,6-Lutidine (0.16 mL, 1.4 mmol) was added to the reaction mixture, and the mixture was dried over 4 Å molecular sieves for 20 min. Then, 50 wt % T3P in EtOAc (0.34 mL, 0.57 mmol) was added to the reaction mixture and allowed to stir at rt for 20 min. The residue was diluted with EtOAc (20 mL) and washed with saturated NaHCO_3_ solutions (3 × 10 mL). The organic layer was dried over Na_2_SO_4_, filtered, and concentrated under reduced pressure. The residue was dissolved in dry CH_2_Cl_2_ (4 mL). Bis­(trimethylsilyl)­acetamide (BSA) (20 μL, 82 μmol) was added to the mixture at rt. The mixture was allowed to stir for a further 10 min. TEA (0.56 mL, 4.0 mmol) was added to the mixture, and the mixture was stirred for 40 min, followed by addition of MeOH (0.40 mL, 9.9 mmol). After stirring for 20 min, the mixture was concentrated by repeated coevaporation with toluene. The residue was purified by silica gel column chromatography. Column chromatography was carried out on a Yamazen UNIVERSAL Premium column (M size: 16 g silica gel, 30 μm, 2.3 × 12.3 cm) using the automated flash chromatography system W-prep 2XY (Yamazen Corporation), which was performed with an isocratic elution of EtOAc–MeOH–TEA (95:5:1, v/v/v) over 3 min followed by a linear gradient of EtOAc–MeOH–TEA (95:5:1–90:10:1, v/v/v) over 19 min, an isocratic elution of EtOAc–MeOH–TEA (90:10:1, v/v/v) for 9 min, a linear gradient of CH_2_Cl_2_–MeOH–TEA (90:10:1–80:20:1, v/v/v) over 19 min, and an isocratic elution of CH_2_Cl_2_–MeOH–TEA (80:20:1, v/v/v) for 20 min. Then, the fractions containing **30atcg** were collected and concentrated under reduced pressure to afford **30atcg** as a colorless foam (166.8 mg, 73 μmol, 40% from **27cg**).

^1^H NMR (CDCl_3_, 500 MHz), ^13^C­{^1^H} NMR (CDCl_3_, 126 MHz), and ^31^P­{^1^H} NMR (CDCl_3_, 202 MHz) spectra are provided in the Supporting Information. HRMS (ESI–QTOF) *m*/*z*: [M–H]^−^ calcd for C_98_H_109_B_2_N_16_O_28_P_4_S^–^ 2135.6460; found, 2135.6401.

### A_P(O)_T_P(B)_C_P(S)_G_P(B)_A_P(O)_T_P(B)_C_P(S)_G 8-Mer Bearing a 5′-OH Group (**32**)

Compound **29atcg** (40.3 mg, 20 μmol), compound **30atcg** (68.6 mg, 30 μmol), and 2,6-lutidine (14 μL, 0.12 mmol) were dissolved in dry MeCN (0.40 mL) and dried over 3 Å molecular sieves. PyNTP (60.2 mg, 0.12 mmol) was added to the mixture at room temperature while stirring. After the mixture was stirred for 20 min, MeOH (16 μL, 0.40 mmol) was added, followed by adding a solution containing TEA (28 μL, 0.20 mmol) and CCl_4_ (19 μL, 0.20 mmol) at rt. The mixture was allowed to stir for a further 20 min. Then, the mixture was diluted with CHCl_3_ (10 mL) and washed with a 1.0 M citrate buffer (pH 3) (10 mL). The aqueous layer was extracted with CHCl_3_ (10 mL). The organic layers were combined, dried over Na_2_SO_4_, filtered, and concentrated to dryness under reduced pressure. Then, the crude mixture was dissolved in dry CH_2_Cl_2_ (2.0 mL). After the addition of 1-dodecanethiol (14 μL, 59 μmol) to the mixture, the reaction mixture was cooled to 0 °C. DCA (49 μL, 0.60 mmol) was added to the mixture at 0 °C while stirring. After the mixture was stirred for 1 h, EtOH (10 μL) was added to the mixture, and the mixture was warmed to rt and stirred for a further 5 min. Then, the mixture was diluted with CH_2_Cl_2_ (5 mL) and washed with a saturated NaHCO_3_ aqueous solution (10 mL). The aqueous layer was extracted with CH_2_Cl_2_ (10 mL). The organic layers were combined, dried over Na_2_SO_4_, filtered, and concentrated to dryness under reduced pressure. The residue was purified by silica gel column chromatography. Column chromatography was carried out on a Yamazen UNIVERSAL Premium column (S size: 7 g silica gel, 30 μm, 1.8 × 11.4 cm) using the automated flash chromatography system W-prep 2XY (Yamazen Corporation), which was performed with an isocratic elution of EtOAc over 3 min, followed by a linear gradient of EtOAc–MeOH (100:0–70:30, v/v) over 20 min. Then, the fractions containing **32** were collected and concentrated under reduced pressure to afford **32** as a colorless foam (76.5 mg). Analysis of compound **32** by ^31^P NMR confirmed the presence of 6.3 equiv of a triaminophosphine oxide derivative relative to compound **32**. Based on ^31^P NMR analysis and considering impurities, the actual amount of compound **32** was estimated to be 14 μmol and utilized in the subsequent reaction without further purification.

### PB/PS/PO Chimeric 12-Mer (Sequence: d­(A_PO_T_PB_C_PS_G_PB_A_PO_T_PB_C_PS_G_PB_A_PO_T_PB_C_PS_G)) (**33**)

Compound **32** (76.5 mg, 14 μmol, including 6.3 equiv of triaminophosphine oxide as impurities), compound **30atcg** (46.9 mg, 21 μmol), and 2,6-lutidine (9.8 μL, 84 μmol) were dissolved in dry MeCN (0.28 mL) and dried over 3 Å molecular sieves. PyNTP (43.1 mg, 86 μmol) was added to the mixture at rt while stirring. After the mixture was stirred for 20 min, MeOH (11 μL, 0.27 mmol) was added, followed by adding a solution containing TEA (19 μL, 0.14 mmol) and CCl_4_ (14 μL, 0.14 mmol) at rt. The mixture was allowed to stir for further 20 min. Then, the mixture was diluted with CHCl_3_ (10 mL) and washed with a 1.0 M citrate buffer (pH 3) (10 mL). The aqueous layer was extracted with CHCl_3_ (10 mL). The organic layers were combined, dried over Na_2_SO_4_, filtered, and concentrated to dryness under reduced pressure. Then, the crude mixture was dissolved in dry CH_2_Cl_2_ (1.4 mL). After adding 1-dodecanethiol (10 μL, 42 μmol) to the mixture, the reaction mixture was cooled to 0 °C. DCA (34 μL, 0.41 mmol) was added to the mixture at 0 °C while stirring. After the mixture was stirred for 1 h, EtOH (20 μL) was added to the mixture, and the mixture was warmed to rt and stirred for a further 5 min. Then, the mixture was diluted with CH_2_Cl_2_ (5 mL) and washed with a saturated NaHCO_3_ aqueous solution (10 mL). The aqueous layer was extracted with CH_2_Cl_2_ (5 mL). The organic layers were combined, dried over Na_2_SO_4_, filtered, and concentrated to dryness under reduced pressure. Thereafter, the mixture was dissolved in dry DMF (1.4 mL). 2-Carbamoyl-2-cyanoethylene-1,1-dithiolate (142.9 mg, 0.70 mmol) was added to the reaction mixture, and the mixture was stirred for 1 h. The reaction mixture was concentrated to dryness under reduced pressure, diluted with EtOAc (10 mL), and washed with a 1.0 M TEAB buffer (pH 7, 3 ×10 mL). The aqueous layer was extracted with CHCl_3_ (3 × 10 mL). The organic layers were combined and concentrated to dryness under reduced pressure. The residue was dissolved in THF (1.4 mL), and a solution containing TEA/3HF (0.23 mL, 1.4 mmol) and TEA (0.39 mL, 2.8 mmol) was added to the reaction mixture and allowed to stir for a further 5 h at rt. Then, the reaction mixture was concentrated under reduced pressure, followed by addition of concentrated NH_3_aq–EtOH (3:1, v/v) (5 mL). The reaction mixture was warmed to 55 °C using a magnetic stirrer with a hot plate (EYELA, Model: RCH-1000) and allowed to stir for 15 h. Then, the reaction mixture was cooled to rt. The reaction mixture was diluted with H_2_O (20 mL) and washed with Et_2_O (3 × 10 mL). The aqueous layer was concentrated to dryness under reduced pressure to give a crude mixture (212.1 mg). A portion of the residue (77.2 mg) was purified by ODS silica gel column chromatography. Column chromatography was carried out on ODS silica gel (Yamazen UNIVERSAL Premium column (30 μm 120 Å) (S size: 7 g silica gel, 30 μm, 1.8 × 11.4 cm) using the automated flash chromatography system W-prep 2XY (Yamazen Corporation), which was performed with an isocratic elution of solution A (50 mM HFIP, 5 mM Hexylamine in H_2_O)–solution B (50 mM HFIP, 5 mM Hexylamine in H_2_O–MeCN, 1:1, v/v) (90:10, v/v) over 2 min, followed by a linear gradient of solution A–solution B (90:10–15:85, v/v) for 40 min. Then, the fractions containing **33** were collected and concentrated under reduced pressure to afford **33** as a colorless solid (4.1 mg, 0.94 μmol, 13% from **29atcg**). ^1^H NMR (D_2_O, 500 MHz), ^13^C­{^1^H} NMR (D_2_O, 126 MHz), ^31^P­{^1^H} NMR (D_2_O, 202 MHz), and ^11^B {^1^H} NMR (D_2_O, 160 MHz)­are provided in the Supporting Information. HRMS (ESI–QTOF) *m*/*z*: [M–4H]^4–^ calcd for C_117_H_159_B_5_N_45_O_62_P_11_S_3_^4–^919.4376; found, 919.4289.

## Supplementary Material



## Data Availability

The data underlying this study are available in the published article and its online Supporting Information.

## Notes

Investigation of an H-boranophosphonylation, LC chart, experimental procedures, and copies of NMR spectra for new compounds (PDF)
