# Supplementary material for: Solution-Phase Synthesis of Boranophosphate and Boranophosphate/Phosphorothioate/Phosphate Chimeric Oligonucleotides via the H‑Boranophosphonate Method
Source: J Org Chem. 2025 Jun 20;90(26):8966–85. doi: 10.1021/acs.joc.5c00583 (PMC12235651; doi:10.1021/acs.joc.5c00583)

Solution-phase synthesis of boranophosphate and boranophosphate/phosphorothioate/phosphate chimeric oligonucleotides via the *H*-boranophosphonate method

Yuhei Takahashi [†], *Itsuki Kato* [†], *Kazuki Sato* [*, †], *Takeshi Wada* [*, †]

[†]Department of Medicinal and Life Sciences, Faculty of Pharmaceutical Sciences, Tokyo University of Science, 6-3-1 Niijuku, Katsushika-ku, Tokyo 125-8585, Japan

Supporting Information

Table of contents

Experimental Section *S5*

General Information S5

3'-*O*-TBDPS-*N*[3]-benzoyl-thymidine (1t) S5

5'-*O*-DMTr-*N*[2]-isobutyryl-*O*[6]-diphenyl carbamoyl-deoxyguanosine (S1g) S6

3'-*O*-TBDPS-*N*[2]-isobutyryl-*O*[6]-diphenyl carbamoyl-deoxyguanosine (1g) S7

5'-*O*-DMTr-*N*[2]-isobutyryl-*O*[6]-diphenyl carbamoyl-deoxyguanosine 3'-*H*-boranophosphonate monomer (2g) S8

Potassium fluorenylmethyl *H*-boranophosphonate monoester (10) S9

Scheme S1 Synthesis of potassium fluorenylmethyl *H*-boranophosphonate monoester (10) S10

Reference for Supporting Information *S10*

UHPLC, UPLC, and ^{31}P NMR analyses *S11*

^{31}P NMR analysis of the purified compound 5ta S11

^{31}P NMR analysis of *H*-boranophosphonylation of a 3'-hydroxy group of compound 5ta ... S11

Investigation of *H*-boranophosphonylation of a 3'-hydroxy group of 5'-*O*-DMTr-*N*[3]-benzoyl-thymidine with the *H*-boranophosphonylation reagent (10) S12

H-boranophosphonylation of the 3'-hydroxy group of compound 5ta using the fluorenylmethyl protected *H*-boranophosphonylation reagent (10) S14

Synthesis of 4-mer building blocks containing boranophosphotriester linkages S15

Synthesis of PB 8-mer containing boranophosphotriester linkages by block condensation using the 4-mer building blocks S16

Synthesis of 12-mer containing boranophosphotriester linkages by block condensation between the 4-mer (15tagc) and the 8-mer (17).. S18

Synthesis of 2-mer building block containing a phosphorothioate triester linkage............ S20

Synthesis of 4-mer building blocks containing boranophosphotriester, phosphorothioate triester, and phosphotriester linkages.. S21

Synthesis of 8-mer containing boranophosphotriester, phosphorothioate triester, and phosphotriester linkages by block condensation using the 4-mer building blocks S22

Synthesis of 12-mer containing boranophosphotriester, phosphorothioate triester, and phosphotriester linkages by block condensation between 4-mer (30atcg) and 8-mer (32) S24

NMR spectra of new compounds.. *S26*

3'-*O*-TBDPS-*N*^{6}-benzoyl-adenosine (1a) S26

3'-*O*-TBDPS-*N*^{4}-isobutyryl-cytosine (1c).. S27

5'-O-DMTr- *N*^{3}-benzoyl-thymidine 3'-*H*-boranophosphonate monomer (2t) S28

5'-*O*-DMTr- *N*^{3}-benzoyl-thymidine 3'-*H*-boranophosphonate monomer (2t) S29

3'-*O*-TBDPS-*N*^{3}-benzoyl-thymidine (1t) S30

5'-*O*-DMTr-*N*^{2}-isobutyryl-*O*^{6}-diphenyl carbamoyl-deoxyguanosine (S1g) S35

3'-*O*-TBDPS-*N*^{2}-isobutyryl-*O*^{6}-diphenyl carbamoyl-deoxyguanosine (1g) S40

5'-*O*-DMTr-*N*^{2}-isobutyryl-*O*^{6}-diphenyl carbamoyl-deoxyguanosine 3'-*H*-boranophosphonate monomer (2g) S45

T-A 2-mer building block bearing *H*-boranophosphonate monoester on 3'-OH group (7ta) S51

Potassium Fluorenylmethyl H-boranophosphonate monoester (10) S58

T-A-G-C 4-mer building block bearing 5′-OH group (14tagc) S71

T-A-G-C 4-mer building block bearing *H*-boranophosphonate monoester on 3'-OH (15tagc) S77

T-A-G-C-T-A-G-C-T-A-G-C 12-mer (18) S84

A-T 2-mer building block bearing H-boranophosphonate monoester on 3'-OH group (23at) S88

C-G 2-mer building block bearing 5'-OH group (27cg) S94

A-T-C-G 4-mer building block bearing 5'-OH group (29atcg) S102

A-T-C-G 4-mer building block bearing *H*-boranophosphonate monoester on 3'-OH (30atcg) S108

A-T-C-G-A-T-C-G-A-T-C-G 12-mer (33) S114

Experimental Section

General Information

All reactions were conducted under an Ar atmosphere. Dry organic solvents were prepared by appropriate procedures. ^{1}H NMR spectra were recorded at 500 MHz with tetramethylsilane (δ 0.00) as an internal standard in $CDCl_3$ or with acetonitrile (MeCN) (δ 2.06) as an internal standard in D_2O. ^{13}C NMR spectra were recorded at 126 MHz with $CDCl_3$ (δ 77.0) as an internal standard in $CDCl_3$. ^{31}P NMR spectra were recorded at 202 MHz in $CDCl_3$ or D_2O with 85% H_3PO_4 (δ 0.0) as an external standard. ^{11}B NMR spectra were recorded at 160 MHz with 15% $BF_3{\cdot}OEt_2$ in $CDCl_3$ as an external standard in $CDCl_3$, or D_2O using a quartz NMR tube. Structural assignments were made with additional information from gCOSY, gHSQC, and gHMBC experiments. Analytical thin-layer chromatography was performed on commercial glass plates with a 0.25 mm thickness silica gel layer. Phosphinic acid as the DIPEA salt was prepared by neutralizing 50% aqueous phosphinic acid with DIPEA, followed by successive coevaporation with pyridine and toluene. Automated silica gel column chromatography was performed on silica gel (Yamazen UNIVERSAL Premium column (30 μm 60 Å)) using automated flash chromatography system W-prep 2XY (Yamazen Corporation). The reaction mixture was analyzed using an ACQUITY Premier UPLC system (Waters) at 260 nm at a temperature of 60°C and a flow rate of 0.5 mL/min using a C18 column (1.7 μm, 2.1 × 50 mm). Synthesized 12-mers were purified by ODS silica gel (Yamazen UNIVERSAL Premium column (30 μm 120 Å)) (Yamazen Corporation) using automated flash chromatography system W-prep 2XY (Yamazen Corporation) and identified by electrospray ionization (ESI) mass spectroscopy, ^{31}P NMR, and ^{1}H NMR. Isolated yields of new compounds including 12-mers were determined by weighing the purified product.

3'-*O*-TBDPS-N^3-benzoyl-thymidine (1t)

5'-*O*-(4, 4'-Dimethoxytrityl)-N^3-benzoyl-thymidine (**S1t**)[1] (3.24 g, 5.0 mmol) and imidazole (1.71 g, 25 mmol) were dissolved in dry MeCN (20 mL) and dried over 3Å molecular sieves. TBDPSCl (1.67 mL, 6.5 mmol) was added to the mixture at rt while stirring. After stirring for 1 h, MeOH (0.81 mL, 20 mmol) was added and the mixture was stirred for a further 5 min. The reaction mixture was then cooled to 0 ˚C, followed by the addition of 1-dodecanethiol (1.55 mL, 6.5 mmol). After 5 min of stirring, trifluoroacetic acid (TFA) (3.1 mL, 41 mmol) was added dropwise over 10 min and the mixture was allowed to stir for 2.5 h. Subsequently, 1-methylimidazole (4.74 mL, 60 mmol) was added at 0 ˚C and the mixture was stirred for 10 min and warmed to room temperature. The reaction mixture was then concentrated under reduced pressure to remove MeCN. The residue was diluted with CH_2Cl_2 (100 mL) and washed with saturated $NaHCO_3$ solutions (3 × 100 mL). The combined aqueous layers were extracted with CH_2Cl_2 (3 × 50 mL). The organic layers were combined, dried over Na_2SO_4, filtered, and concentrated under reduced

pressure. The residue was purified by silica gel column chromatography. Column chromatography was carried out on Yamazen UNIVERSAL Premium column (2L size: 54 g silica gel, 30 μm, 3.0 × 20.0 cm) using automated flash chromatography system W-prep 2XY (Yamazen Corporation), which was performed with an isocratic elution of EtOAc–hexane (53:47, v/v) over 4 min followed by a linear gradient of EtOAc–hexane (53:47–74:26, v/v) over 13 min. Then, the fractions containing **1t** were collected and concentrated under reduced pressure to afford **1t** as a colorless foam (2.42 g, 4.1 mmol, 83% R_f = 0.40 (EtOAc–Hexane=6:4, v/v, neutral silica)).

^{1}H NMR ($CDCl_3$, 500 MHz): δ 7.92–7.89 (m, 2H, Ar-H), 7.67–7.61 (m, 5H, Ar-H), 7.50–7.44 (m, 5H, Ar-H, H-6), 7.42–7.38 (m, 4H, Ar-H), 6.31 (dd, *J* = 7.5, 6.2 Hz, 1H, H-1'), 4.44 (dt, *J* = 6.0 , 2.9 Hz, 1H, H-3'), 3.98 (dt, *J* = 2.7, 4.0 Hz, 1H, H-4'), 3.66–3.61 (m, 1H, H-5'), 3.29–3.22 (m, 1H, H-5''), 2.32 (ddd, *J* = 13.4, 5.8, 2.9 Hz, 1H, H-2'), 2.13 (dt, *J* = 14.3, 6.8 Hz, 1H, H-2''), 1.89 (d, *J* = 1.1 Hz, 3H, 5-CH_3), 1.07 (s, 9H, -C(CH_3)$_3$); ^{13}C{^{1}H} NMR (126 MHz, $CDCl_3$) δ 168.9 (-Ph<u>C</u>ON-), 162.8 (C-4), 149.4 (C-2), 136.3 (C-6), 135.7, 135.7, 135.0, 133.2, 132.9, 131.5, 130.4, 130.1, 130.1, 129.1, 127.9, 127.9, 111.0 (C-5), 87.6 (C-4'), 86.5 (C-1'), 72.9 (C-3'), 62.0 (C-5'), 40.6 (C-2'), 26.8 (-C(<u>C</u>H$_3$)$_3$), 19.0 (-<u>C</u>(CH$_3$)$_3$), 12.6 (5-CH_3); HRMS (ESI–QTOF) m/z: [M+H]$^+$ Calcd for $C_{33}H_{37}N_2O_6Si^+$ 585.2415; Found 585.2390.

5'-*O*-DMTr-*N*^{2}-isobutyryl-*O*^{6}-diphenyl carbamoyl-deoxyguanosine (S1g)

5'-*O*-(4, 4'-Dimethoxytrityl)-*N*^{2}-isobutyryldeoxyguanosine (12.8 g, 20 mmol) was dissolved in dry CH_2Cl_2 (80 mL). Hexamethyldisilazane (HMDS) (6.7 mL, 32 mmol) and iodine (59.7 mg, 0.24 mmol) were successively added to the reaction mixture and stirred at rt for 1 h. After that, EtOH (1.1 mL) was added to the reaction mixture and stirred for further 5 min. Then, the reaction mixture was concentrated under reduced pressure and dried by repeated coevaporation with $CHCl_3$. The residue was diluted with dry pyridine (100 mL). DIPEA (17.1 mL, 100 mmol) and diphenyl carbamoyl chloride (11.56 g, 50 mmol) were added to the mixture at rt and the mixture was stirred for 1.5 h. Subsequently, MeOH (150 mL) and H_2O (75 mL) were added to the mixture, and the reaction mixture was allowed to stir at rt for further 16.5 h. Next, the reaction mixture was concentrated under reduced pressure. The residue was diluted with EtOAc (150 mL) and washed with saturated $NaHCO_3$ solutions (2 × 100 mL). The combined aqueous layers were extracted with EtOAc (2 × 50 mL). The organic layers were combined, dried over Na_2SO_4, filtered, and concentrated under reduced pressure. The residue was purified by silica gel column chromatography. Column chromatography was carried out on Yamazen UNIVERSAL Premium column (3L size: 132 g silica gel, 30 μm, 5.0 × 19.0 cm) using automated flash chromatography system W-prep 2XY (Yamazen Corporation), which was performed with an isocratic elution of EtOAc–hexane (90:10, v/v) over 3 min followed by a linear gradient of EtOAc–hexane (90:10–100:0, v/v) over 10 min and isocratic elution of EtOAc for 14 min. Then, the fractions

containing compound **S1g** were collected and concentrated under reduced pressure to afford compound **S1g** as a pink foam (16.1 g, 19 mmol, 96%, R_f = 0.40 (EtOAc–Hexane=9:1, v/v, neutral silica)).

^{1}H NMR ($CDCl_3$, 500 MHz): δ 8.14 (s, 1H, H-8), 8.09 (brs, 1H, -CONH-), 7.46–7.32 (m, 10H, Ar-H), 7.30–7.14 (m, 9H, Ar-H), 6.79–6.74 (m, 4H, Ar-H), 6.64 (t, J = 6.5 Hz, 1H, H-1'), 4.76–4.71 (m, 1H, H-3'), 4.22 (td, J = 3.5, 3.3 Hz, 1H, H-4'), 3.73 (s, 6H, -OCH_3 of DMTr), 3.38 (dq, J = 9.6, 4.2 Hz, 2H, H-5', H-5''), 2.74–2.58 (m, 3H, H-2', H-2'' and -$CH(CH_3)_2$), 1.18 (d, J = 1.9 Hz, 3H, -$CH(CH_3)_2$), 1.16 (d, J = 1.9 Hz, 3H, -$CH(CH_3)_2$); ^{13}C{^{1}H} NMR ($CDCl_3$, 126 MHz): δ 175.2 (-CONH-), 158.4 (C-6), 155.9 (C-2), 154.6 (C-4), 151.6, 150.4, 144.5, 142.7 (C-8), 141.7, 135.7, 135.6, 130.0, 130.0, 129.1, 128.0, 127.4, 126.5 (C-5), 125.9, 125.7, 121.6, 113.1, 86.7, 86.4 (C-4'), 84.5 (C-1'), 72.3 (C-3'), 64.0 (C-5'), 55.1 (-OCH_3 of DMTr), 40.8 (C-2'), 36.2 (-$CH(CH_3)_2$), 19.2 (-$C(CH_3)_3$); HRMS (ESI–QTOF) m/z: $[M+H]^+$ Calcd for $C_{48}H_{47}N_6O_8^+$ 835.3450; Found 835.3448.

3'-*O*-TBDPS-*N*^{2}-isobutyryl-*O*^{6}-diphenyl carbamoyl-deoxyguanosine (1g)

5'-*O*-(4, 4'-Dimethoxytrityl)-*N*^{2}-isobutyryl-*O*^{6}-diphenyl carbamoyl-deoxyguanosine (**S1g**) (8.39 g, 10 mmol) and imidazole (3.42 g, 50 mmol) were dissolved in dry MeCN (40 mL) and dried over 3Å molecular sieves. TBDPSCl (3.4 mL, 13 mmol) was added to the mixture at rt while stirring. After stirring for 45 min, MeOH (1.6 mL, 40 mmol) was added, and the mixture was stirred for 5 min. The reaction mixture was then cooled to 0 ˚C, followed by the addition of 1-dodecanethiol (3.1 mL, 13 mmol). After 10 min of stirring, TFA (6.1 mL, 80 mmol) was added dropwise over 20 min and the mixture was allowed to stir at rt for 2 h. Subsequently, 1-methylimidazole (9.4 mL, 120 mmol) was added at 0 ˚C and the mixture was warmed rt and stirred for 10 min. The reaction mixture was then concentrated under reduced pressure to remove MeCN. The residue was diluted with CH_2Cl_2 (100 mL) and washed with saturated $NaHCO_3$ solutions (3 × 100 mL). The combined aqueous layers were extracted with CH_2Cl_2 (3 × 50 mL). The organic layers were combined, dried over Na_2SO_4, filtered, and concentrated under reduced pressure. The residue was purified by silica gel column chromatography. Column chromatography was carried out on Yamazen UNIVERSAL Premium column (3L size: 132 g silica gel, 30 µm, 5.0 × 19.0 cm) using automated flash chromatography system W-prep 2XY (Yamazen Corporation), which was performed with an isocratic elution of EtOAc–hexane (64:36, v/v) over 3 min followed by a linear gradient of EtOAc–hexane (64:36–85:15, v/v) over 10 min. Then, the fractions containing **1g** were collected and concentrated under reduced pressure to afford **1g** as a pink foam (6.48 g, 8.4 mmol, 84%, R_f = 0.34 (EtOAc–Hexane=6:4, v/v, neutral silica)).

^{1}H NMR ($CDCl_3$, 500 MHz): δ 8.01 (s, 1H, H-8), 7.98 (brs, 1H, -CONH-), 7.67–7.63 (m, 4H, Ar-H), 7.47–7.31 (m, 14H, Ar-H), 7.27–7.19 (m, 2H, Ar-H), 6.32 (dd, J = 9.3, 5.3 Hz, 1H, H-1'), 4.77–4.73 (m, 1H, H-3'), 4.13–4.07 (m, 2H, H-4'. -OH), 3.67 (ddd, J = 12.9, 3.6, 2.1 Hz, 1H, H-5'), 3.25 (ddd, J = 10.4, 9.8, 2.6 Hz, 1H, H-5''), 2.86 (ddd.

J = 13.1, 8.8, 5.3 Hz, 1H, H-2'), 2.81–2.70 (m, 1H, -C<u>H</u>(CH_3)$_2$), 2.25 (ddd. J = 12.7, 5.4, 1.3 Hz, 1H, H-2"), 1.21 (s, 3H, -CH(C<u>H</u>$_3$)$_2$), 1.20 (s, 3H, -CH(C<u>H</u>$_3$)$_2$), 1.12 (s, 9H, -C(CH_3)$_3$); $^{13}C\{^1H\}$ NMR ($CDCl_3$, 126 MHz): δ 175.1 (-<u>C</u>ONH-), 156.3, 154.1 (C-4), 151.4, 151.4, 150.2, 143.7 (C-8), 141.6, 135.7, 135.6, 133.2, 133.1, 130.1, 130.1, 129.1, 127.9, 127.7, 127.6, 127.6, 126.6, 125.8, 121.6 (C-5), 113.1, 89.4 (C-4'), 87.0 (C-1'), 74.2 (C-3'), 62.5 (C-5'), 40.5 (C-2'), 36.2 (-<u>C</u>H(CH_3)$_2$), 26.9 (-C(<u>C</u>H$_3$)$_3$), 19.1 (-CH(<u>C</u>H$_3$)$_2$), 19.0 (-<u>C</u>(CH_3)$_3$). HRMS (ESI–QTOF) m/z: $[M+H]^+$ Calcd for $C_{43}H_{47}N_6O_6Si^+$ 771.3321; Found 771.3317.

5'-*O*-DMTr-*N*^{2}-isobutyryl-*O*^{6}-diphenyl carbamoyl-deoxyguanosine 3'-*H*-boranophosphonate monomer (2g)

5'-*O*-(4, 4'-Dimethoxytrityl)-*N*^{2}-isobutyryl-*O*^{6}-diphenyl carbamoyl-deoxyguanosine (**S1g**) (4.17 g, 5.0 mmol) and pyridinium *H*-boranophosphonate **6** (2.73 g, 9.8 mmol) were dried by repeated coevaporation with dry pyridine and dissolved together in dry pyridine (100 mL) at 0 °C and dried over 4Å molecular sieves. Bis(2-oxo-3-oxazolidinyl) phosphinic chloride (Bop-Cl) (2.54 g, 10 mmol) was added at 0 °C, and the mixture was stirred for 5 min at 0 °C. Then, the mixture was warmed to rt and stirred for 1.5 h. The reaction mixture was then concentrated under reduced pressure to remove pyridine and diluted with $CHCl_3$ (150 mL) and washed with 1.0 M triethylammonium bicarbonate (TEAB) buffers (pH 7) (3 × 100 mL). The organic layer was combined, dried over Na_2SO_4, filtered, and concentrated under reduced pressure. The residue was purified by silica gel column chromatography. Column chromatography was carried out on Yamazen UNIVERSAL Premium column (2L size: 54 g silica gel, 30 µm, 3.0 × 20.0 cm) using automated flash chromatography system W-prep 2XY (Yamazen Corporation), which was performed with an isocratic elution of EtOAc–triethylamine (TEA) (100:1, v/v) over 4 min followed by a linear gradient of EtOAc–MeOH–TEA (100:0:1–80:20:1, v/v/v) over 25 min, an isocratic elution of EtOAc–MeOH–TEA (80:20:1, v/v/v) over 6 min, and an isocratic elution CH_2Cl_2–TEA (100:1, v/v) over 6 min. Then, the fractions containing **2g** were collected and concentrated under reduced pressure to afford **2g** as a pale red foam (2.97 g, 3.0 mmol, 60%).

^{1}H NMR ($CDCl_3$, 500 MHz): δ 8.14 (s, 0.5H, H-8), 8.10 (s, 0.5H, H-8), 7.91 (s, 0.5H, -CON<u>H</u>-), 7.91 (s, 0.5H, -CON<u>H</u>-), 7.70–7.62 (brs, 0.5H, P-H), 7.49–7.32 (m, 10H, Ar-H), 7.32–7.13 (m, 9H, Ar-H), 6.92–6.84 (brs, 0.5H, P-H), 6.80–6.71 (m, 4H, Ar-H), 6.45 (dd, J = 7.4, 6.0 Hz, 1H, H-1'), 5.11 (ddd. J = 10.0, 5.8, 2.8 Hz, 0.5H, H-3'), 5.01 (ddd. J = 10.1, 6.2, 3.1 Hz, 0.5H, H-3'), 4.40 (dt. J = 4.5, 2.9 Hz, 0.5H, H-4'), 4.37 (dt. J = 3.5, 3.2 Hz, 0.5H, H-4'), 3.75–3.72 (m, 6H, -OC<u>H</u>$_3$ of DMTr), 3.45–3.43 (m, 2H, H-5', H-5"), 3.00 (q, J = 7.3 Hz, 6H, -CH_2- of TEA), 2.93–2.68 (m, 3H, H-2', H-2", -C<u>H</u>(CH_3)$_2$), 1.27 (t, J = 7.3 Hz, 9H, -C<u>H</u>$_3$- of TEA), 1.22–1.17 (m, 6H, -CH(C<u>H</u>$_3$)$_2$), 1.0–0.1 (br, 3H, BH_3); $^{13}C\{^1H\}$ NMR ($CDCl_3$, 126 MHz): δ 175.6 (-<u>C</u>ONH-), 158.5, 158.4, 156.0, 156.0, 154.5 (C-4), 154.5 (C-4), 151.9, 151.8, 150.5, 150.5, 144.6, 144.5, 142.7 (C-8), 142.6 (C-8), 141.8, 135.8, 135.7, 135.6, 130.1, 130.0, 130.0, 130.0, 129.1, 128.1, 128.1, 127.8, 127.8, 126.8, 126.8, 121.5, 113.2, 113.1, 86.4, 86.3, 86.0 (d, $^3J_{C–P}$ = 4.3 Hz, C-4') , 85.7 (d, $^3J_{C–P}$ = 4.6 Hz, C-4'), 84.7 (C-1'), 84.6 (C-1'), 76.6 (d, $^2J_{C–P}$ = 8.8 Hz, C-3'), 75.4 (d, $^3J_{C–P}$ =

6.6 Hz, C-3'), 63.7 (C-5'), 63.4 (C-5'), 55.2 (-O$\underline{C}H_3$ of DMTr), 45.3 (-$\underline{C}H_2$- of TEA), 40.1 (C-2'), 39.2 (d, $^3J_{C-P}$ = 2.6 Hz, C-2'), 35.6 (-$\underline{C}H(CH_3)_2$), 19.2 (-CH($\underline{C}H_3)_2$), 19.2 (-CH($\underline{C}H_3)_2$), 19.1 (-CH($\underline{C}H_3)_2$), 8.5 (-$\underline{C}H_3$- of TEA); $^{31}P\{^1H\}$ NMR ($CDCl_3$, 202 MHz): δ 106.5–102.0; HRMS (ESI–QTOF) m/z: $[M–H]^-$ Calcd for $C_{48}H_{49}BN_6O_9P^-$, 895.3397; Found 895.3381.

Potassium fluorenylmethyl *H*-boranophosphonate monoester (10)

A phosphinic acid diisopropylethylammnonium salt (9.8 g, 50 mmol) and 9-fluorenylmethanol (10.9 g, 55 mmol) were dissolved in deoxygenated CH_2Cl_2 (150 mL). To this mixture, 2,6-lutidine (17.8 mL, 150 mmol) and pyridine (4.0 mL, 50 mmol) were added. The reaction mixture was cooled to 0 ˚C on an ice bath and PivCl (7.4 mL, 61 mmol) was then added at 0 ˚C to the reaction mixture while stirring. The mixture was warmed to rt 5 min after addition of PivCl and allowed to stir for a further 2 h. Thereafter, the mixture was cooled to 0 ˚C on an ice bath and stir for 10 min. A mixture of TMSCl (19.0 mL, 150 mmol) and $BH_3 \cdot SMe_2$ (14.5 mL, 150 mmol) was added dropwise to the reaction mixture over 4 min. The reaction mixture was then warmed to rt and stir for further 20 min. Then, the reaction mixture was cooled to 0 ˚C on an ice bath for 5 min. To the reaction mixture, H_2O (100 mL) was added and stir for further 10 min. The mixture was warmed to rt, diluted with CH_2Cl_2 (150 mL), and wash with a 1.0 M HCl aqueous solution (48 mL). The organic layer was neutralized by the addition of TEA (17 mL, 120 mmol) and concentrated under reduced pressure. The resulting residue was dissolved in EtOAc (300 mL) and washed with a saturated $NaHCO_3$ aqueous solution (150 mL). The organic layer was further washed with an aqueous solution of pyridinium hydrochloride (1.5 M) and a saturated NaCl solution (1:1, v/v) (100 mL) and concentrated under reduced pressure. The residue was dissolved in toluene (150 mL) and washed with a saturated $KHCO_3$ aqueous solution (200 mL). The aqueous layer was extracted with toluene (100 mL). The aqueous layer was back-extracted with EtOAc (3 × 100 mL). The combined EtOAc layers were dried over Na_2SO_4, filtered, and concentrated under reduced pressure to afford compound **10** as a colorless foam (9.56 g, 32 mmol, 64%). This reagent was used for *H*-boranophosphonylation reactions without further purification.

^1H NMR ($CDCl_3$, 500 MHz): δ 7.72 (s, 1H, Ar-H), 7.70 (s, 1H, Ar-H), 7.61 (s, 1H, Ar-H), 7.60 (s, 1H, Ar-H), 7.55 (s, 1H, Ar-H), 7.54 (s, 1H, Ar-H), 7.50 (s, 1H, Ar-H), 7.49 (s, 1H, Ar-H), 7.42–7.32 (brs, 0.5H, P-H), 7.28–7.22 (m, 2H, Ar-H), 7.20–7.13 (m, 2H, Ar-H), 6.58 (brs, 0.5H, P-H), 4.34–4.19 (m, 1H), 4.12–3.99 (m, 2H), 0.61– -0.26 (br, 3H, BH_3); $^{13}C\{^1H\}$ NMR ($CDCl_3$, 126 MHz): δ 143.9, 143.7, 141.1, 141.0, 127.7, 127.7, 127.2, 127.2, 125.1, 125.0, 120.0, 120.0, 77.0, 68.1, 48.4, 48.4; $^{31}P\{^1H\}$ NMR ($CDCl_3$, 202 MHz): δ 108.5–104.1; HRMS (ESI–QTOF) m/z: $[M–H]^-$ Calcd for $C_{14}H_{15}BO_2P^-$ 257.0908; Found 257.0908.

Scheme S1 Synthesis of potassium fluorenylmethyl *H*-boranophosphonate monoester (**10**)

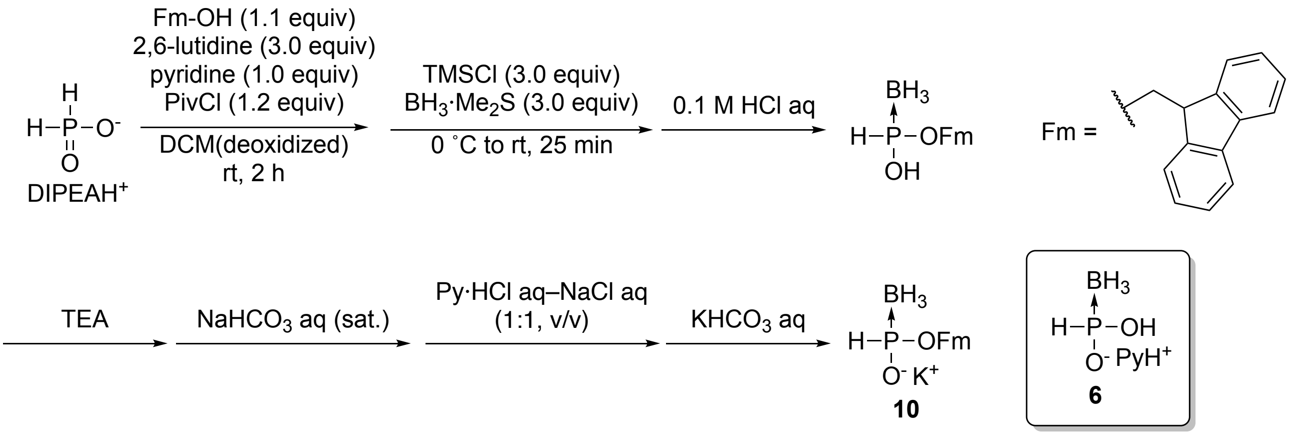

Reference for Supporting Information

(1) Peyrat, S.; Xie, J. Synthesis of Thymidine Dimers from 5'-O-Aminothymidine. *Synth.* **2012**, *44* (11), 1718–1724. https://doi.org/10.1055/s-0031-1289759.

UHPLC, UPLC, and ^{31}P NMR analyses

^{31}P NMR analysis of the purified compound 5ta

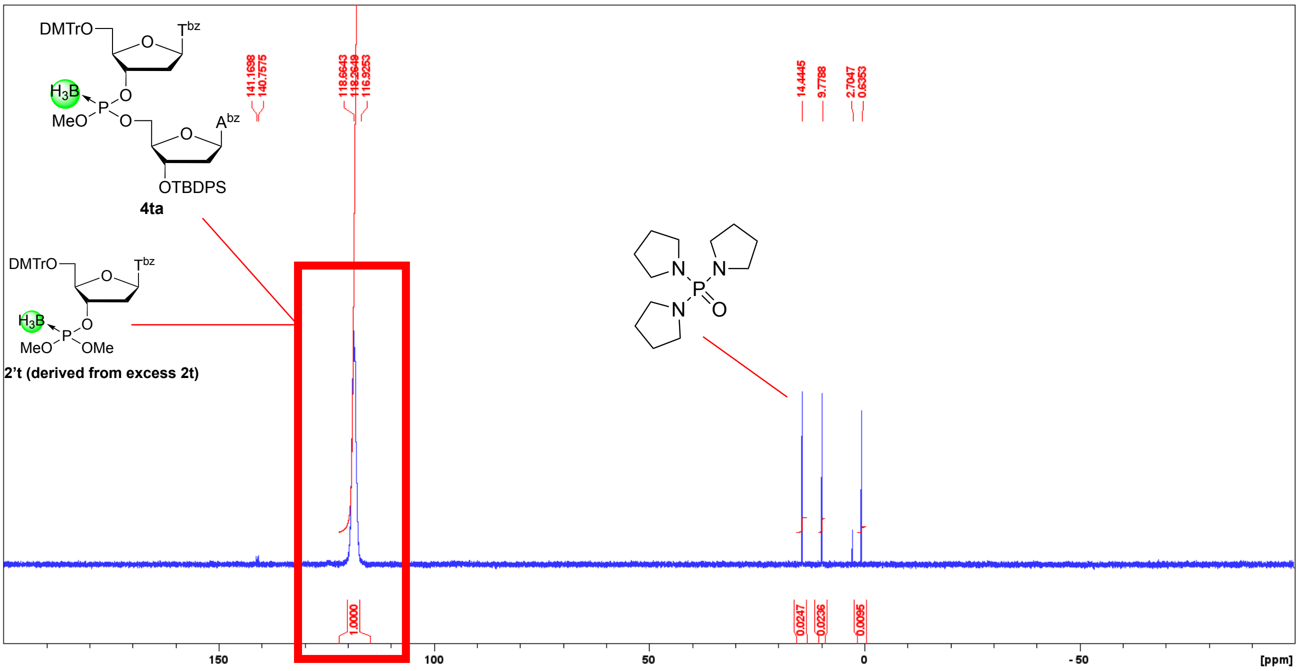

Figure S1 ^{31}P NMR spectrum of the purified compound **5ta** through silica gel column chromatography

^{31}P NMR analysis of *H*-boranophosphonylation of a 3'-hydroxy group of compound 5ta

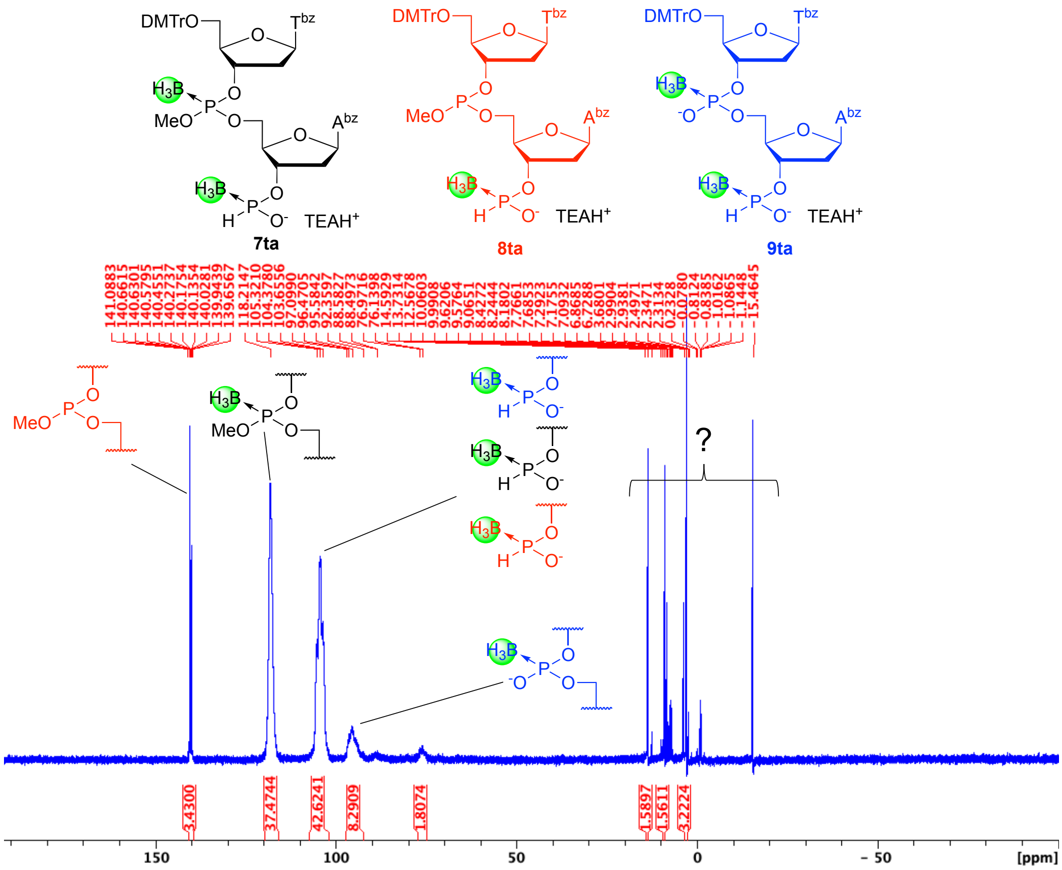

Figure S2 ^{31}P NMR spectrum of the crude mixture after work-up of the *H*-boranophosphonylation using *H*-boranophosphonylation reagent (**6**)

Investigation of *H*-boranophosphonylation of a 3'-hydroxy group of 5'-*O*-DMTr-*N*^{3}-benzoyl-thymidine with the *H*-boranophosphonylation reagent (10)

The reaction conditions for *H*-boranophosphonylation were investigated using 5'-O-DMTr-*N*^{3}-benzyl thymidine (**S1**) and the *H*-boranophosphonylating reagent **10**. Compound **10** was condensed with the 3'-hydroxy group of the thymidine derivative in the presence of a condensing reagent and 2,6-lutidine in MeCN. Excess condensing reagent was removed by extraction using EtOAc/saturated $NaHCO_3$ aqueous solution. The resulting crude mixture of **S2** was analyzed by ^{1}H NMR to evaluate the efficiency of the condensation and the residual amount of the condensing agent.

First, condensing reagents were examined. BopCl, DPCP, and T3P, which can be easily removed by extraction, were selected as condensing reagents. After the *H*-boranophosphonylation reaction, the crude product after work-up was analyzed by ^{1}H NMR. It was confirmed that the reaction proceeded over 95% for all condensing reagents. Furthermore, analysis by ^{31}P NMR revealed the presence of a residual condensing reagent when BopCl and DPCP were used. In contrast, no residues of T3P were detected when it was employed as a condensing agent. Based on these results, T3P, which allows for the quantitative removal of residual condensing agents, was chosen as the optimal condensing reagent.

Next, the solvent used during the condensation with the *H*-boranophosphonylating reagent was investigated. In addition to MeCN, CH_2Cl_2 and EtOAc were employed as solvents. The crude mixtures obtained after the reactions were analyzed by ^{1}H NMR, and it was confirmed that the reaction proceeded over 95% in all solvents. While detailed reaction times for MeCN and EtOAc were not recorded, CH_2Cl_2 allowed for the reaction to complete in under 3 minutes, ensuring efficient and rapid reaction completion.

Scheme S2 Investigation of *H*-boranophosphonylation using thymidine derivative

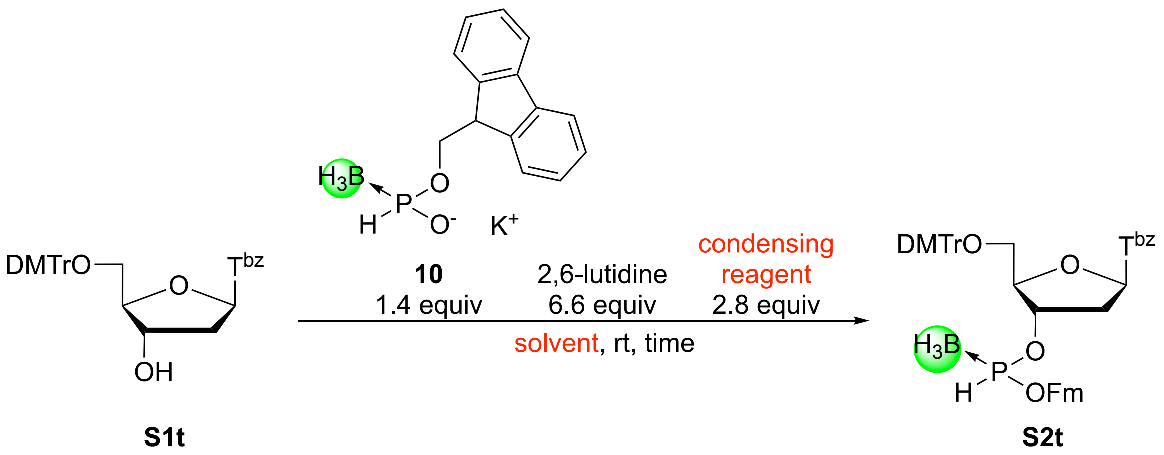

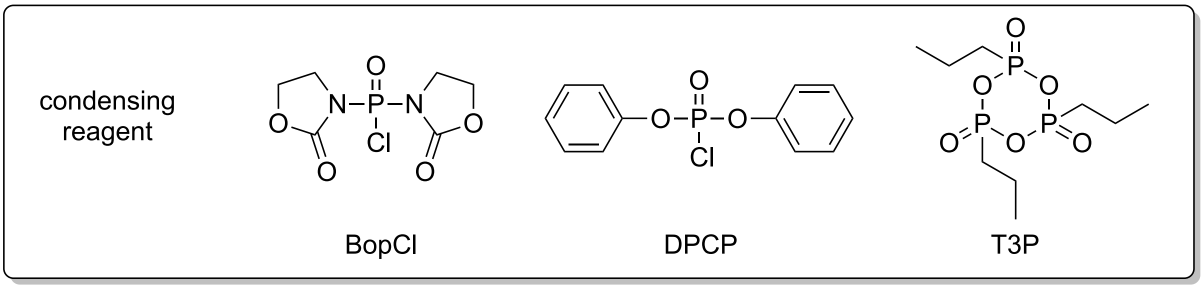

entry	condensing reagent	solvent	Reaction duration[a]
1	BopCl	MeCN	20 min
2	DPCP	MeCN	<5 min
3	T3P	MeCN	<5 min
4	T3P	EtOAc	<5 min
5	T3P	CH_2Cl_2	<3 min

[a] The reaction time was determined based on the point at which the reaction reached completion, as analyzed by UHPLC.

Using the condensation conditions in entry 5, compound **10** was condensed with the 3'-hydroxy group of thymidine derivative (**S1**), followed by the removal of excess T3P through extraction. Subsequently, the fluorenylmethyl (Fm) group was removed using TEA (Scheme S3). The crude product obtained was purified by silica gel column chromatography, affording the desired compound in 91% yield.

Scheme S3 *H*-Boranophosphonylation of the thymidine derivative (**S1t**) using the *H*-boranophosphonylation reagent (**10**)

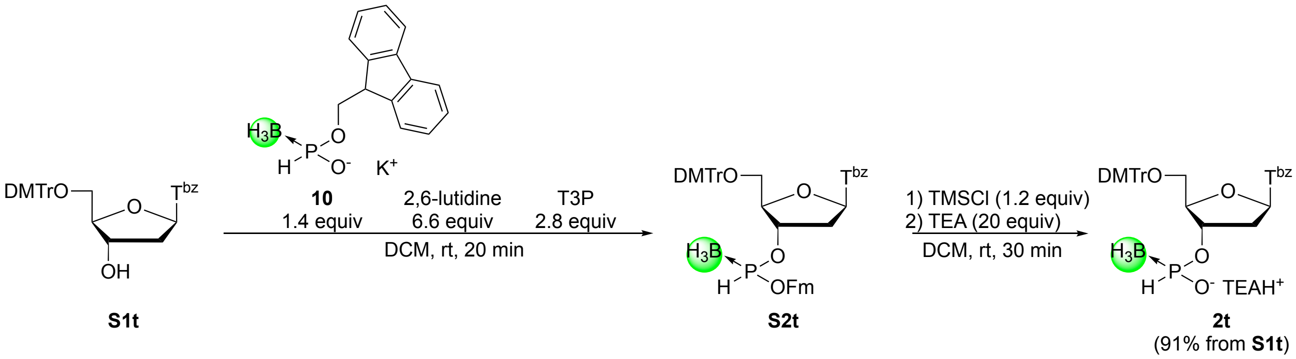

***H*-boranophosphonylation of the 3'-hydroxy group of compound 5ta using the fluorenylmethyl protected *H*-boranophosphonylation reagent (10)**

Figure S3 ^{31}P NMR spectrum of the crude mixture of **7ta** after work-up of the *H*-boranophosphonylation

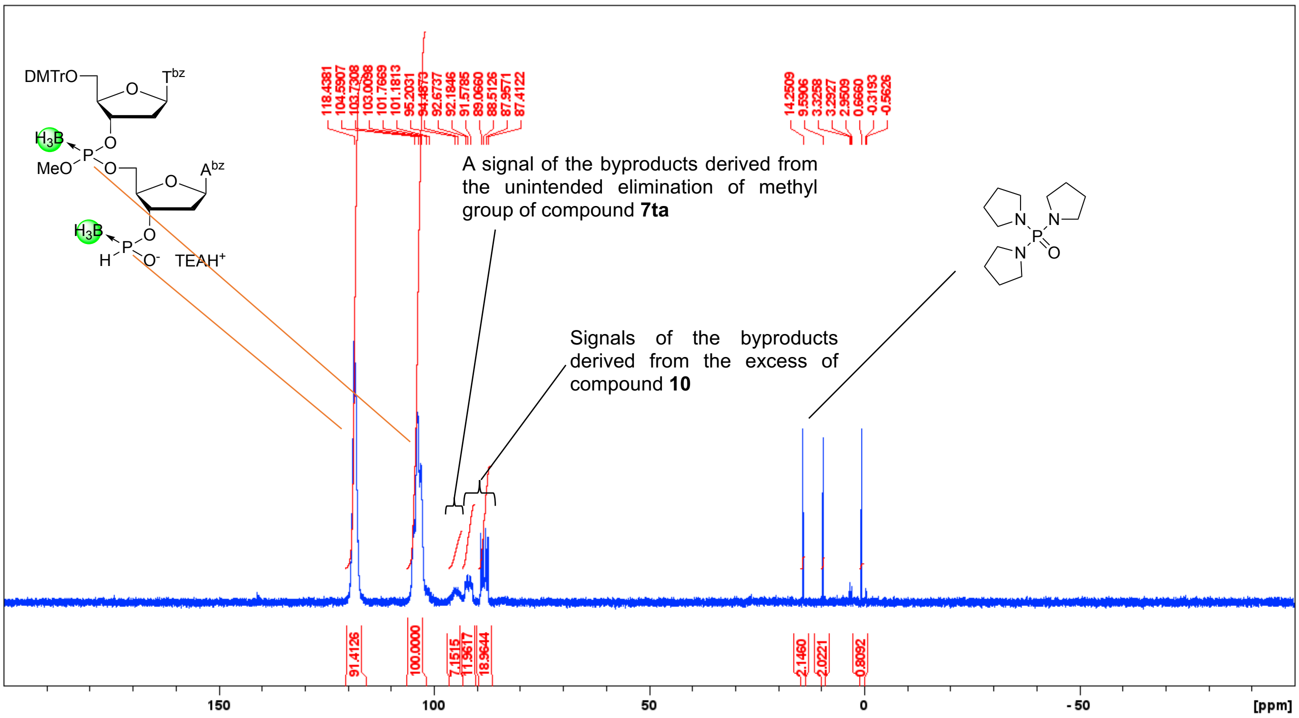

Synthesis of 4-mer building blocks containing boranophosphotriester linkages

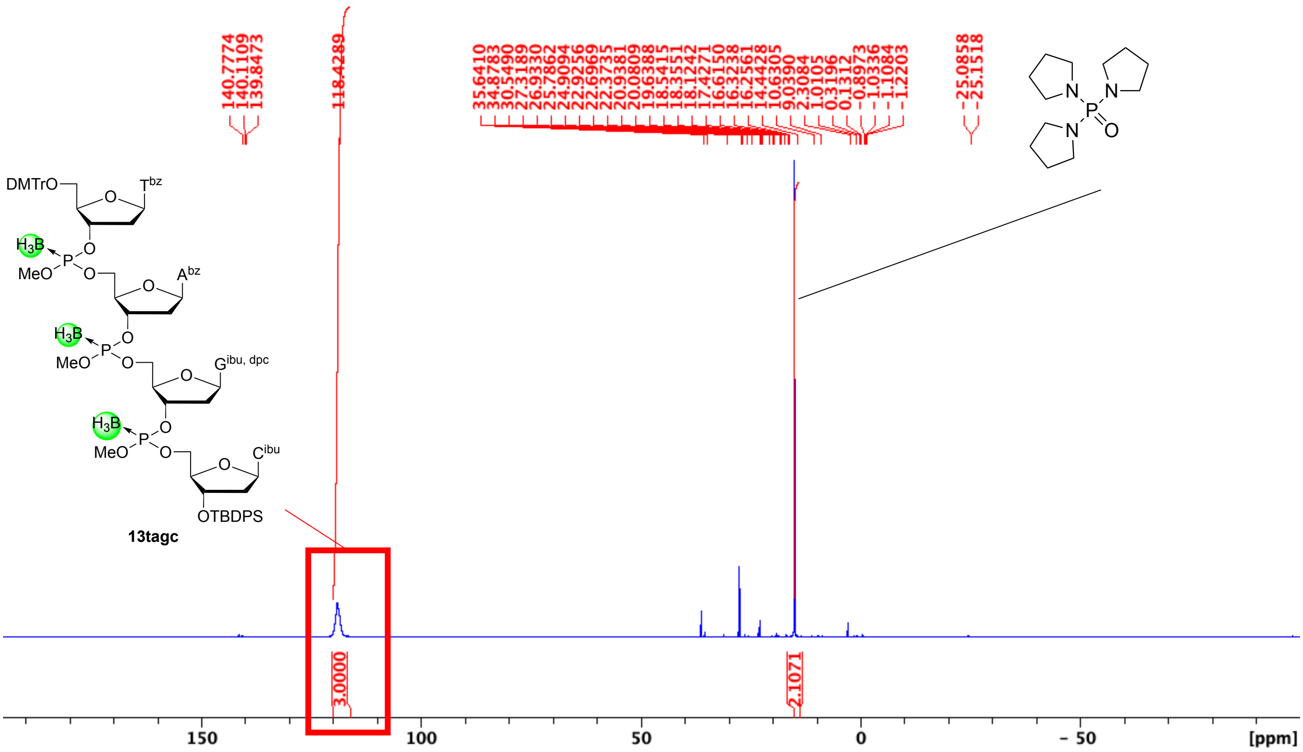

Figure S4 ^{31}P NMR spectrum of crude mixture after work-up of oxidative esterification using 2-mer building blocks

Synthesis of PB 8-mer containing boranophosphotriester linkages by block condensation using the 4-mer building blocks

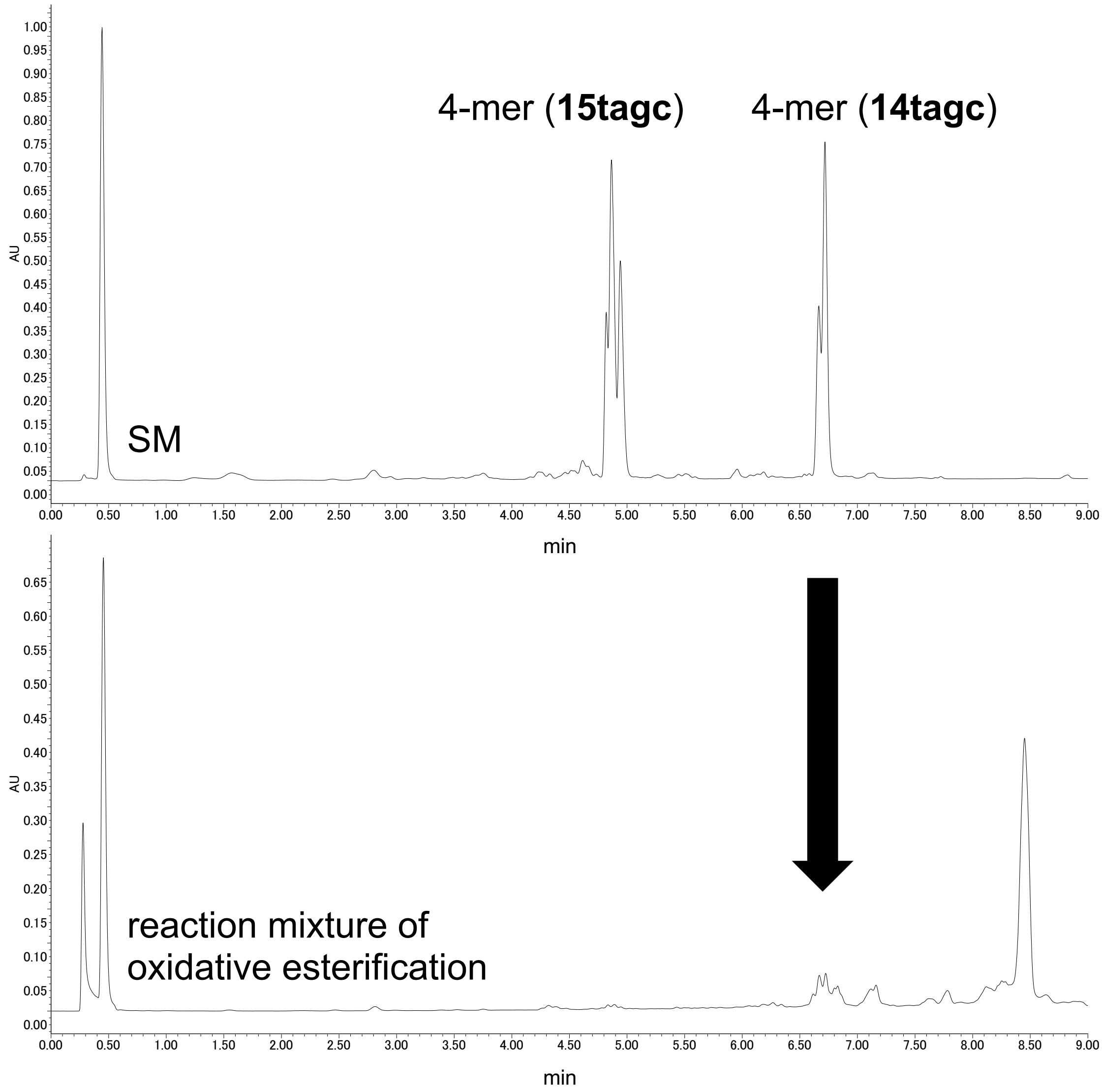

Figure S5 UHPLC profile of crude mixture after work-up of the oxidative esterification (1.4 equivalents of 5'-upstream 4-mer building block and 6.0 equivalents of PyNTP were used). UHPLC (ODS, H_2O–MeCN (9:1, v/v, containing 50 mM HFIP, 5 mM TEA) / MeCN = 60:40–0:100 over 8 min, 60 °C, flow rate = 0.5 mL/min, λ = 260 nm).

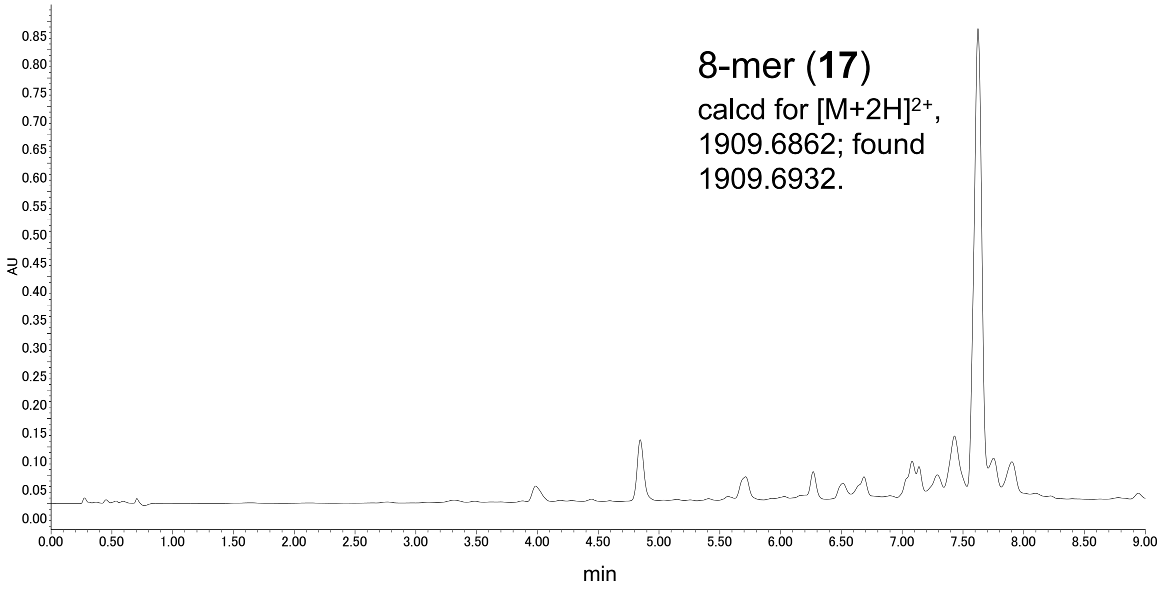

Figure S6 UHPLC profile of the purified 8-mer (**17**) through silica gel column chromatography. UHPLC (ODS, H_2O–MeCN (9:1, v/v, containing 50 mM HFIP, 5 mM TEA) / MeCN = 60:40–0:100 over 8 min, 60 °C, flow rate = 0.5 mL/min, λ = 260 nm).tR = 7.5 min

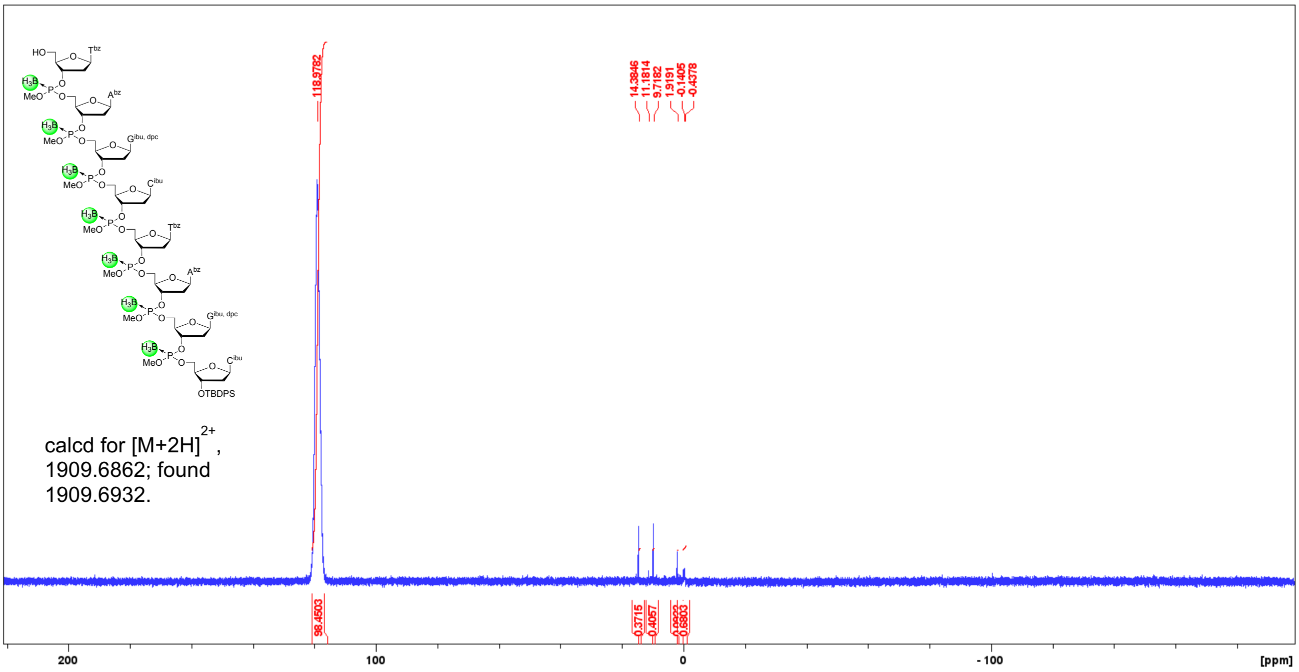

Figure S7 ^{31}P NMR spectrum of the purified 8-mer (**17**) through silica gel column chromatography

Synthesis of 12-mer containing boranophosphotriester linkages by block condensation between the 4-mer (15tagc) and the 8-mer (17)

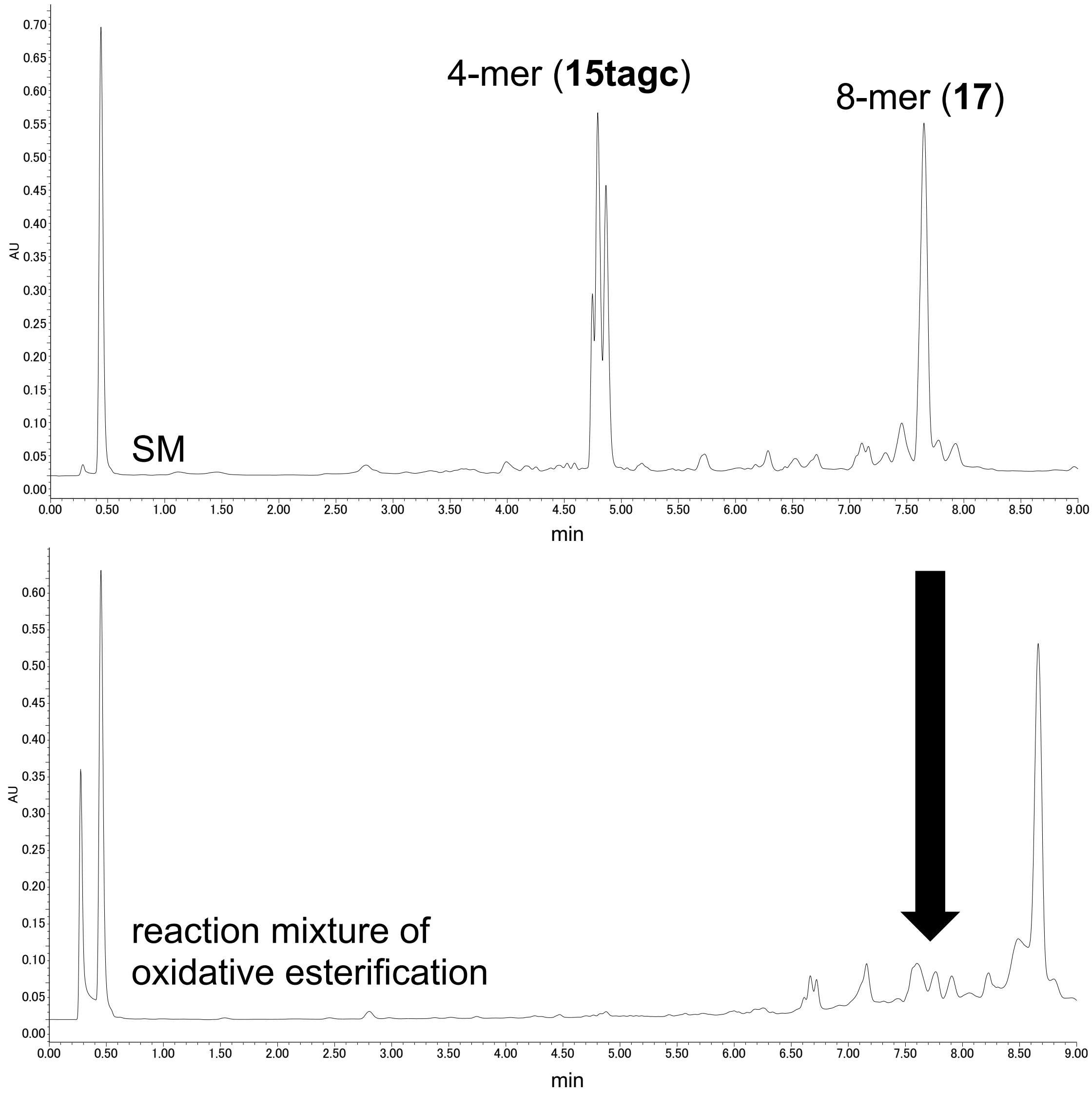

Figure S8 UHPLC profile of block condensation and oxidative esterification between 4-mer and 8-mer. UHPLC (ODS, H_2O–MeCN (9:1, v/v, containing 50 mM HFIP, 5 mM TEA) / MeCN = 60:40–0:100 over 8 min, 60 °C, flow rate = 0.5 mL/min, λ = 260 nm).

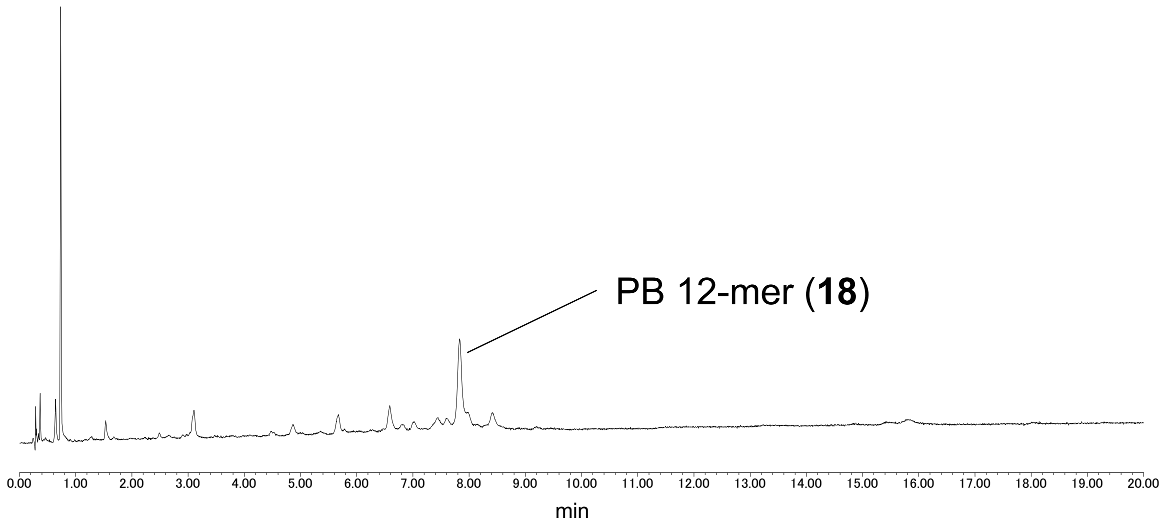

Figure S9 UPLC profile of the crude mixture of PB 12-mer (**18**) after removal of all protecting groups. UPLC (ODS, H_2O (containing 50 mM HFIP, 5 mM TEA) / H_2O–MeCN (1:1, v/v, containing 50 mM HFIP, 5 mM TEA) = 90:10–15:85 over 20 min, 60 °C, flow rate = 0.5 mL/min, λ = 260 nm). tR = 7.8 min (main peak).

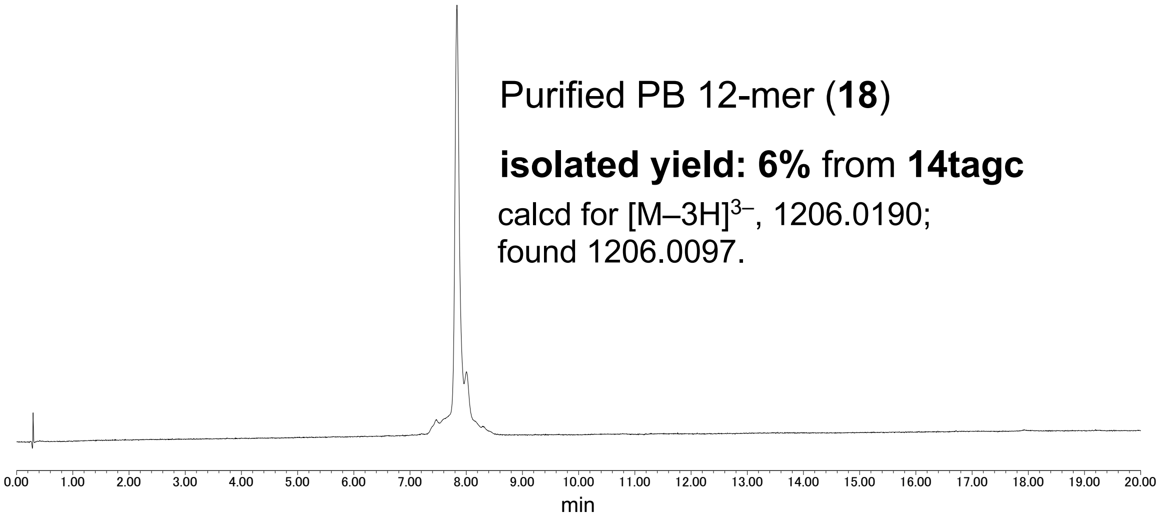

Figure S10 UPLC profile of the purified PB 12-mer (**18**) after removal of all protecting groups. UPLC (ODS, H_2O (containing 50 mM HFIP, 5 mM TEA) / H_2O–MeCN (1:1, v/v, containing 50 mM HFIP, 5 mM TEA) = 90:10–15:85 over 20 min, 60 °C, flow rate = 0.5 mL/min, λ = 260 nm). tR = 7.8 min.

Synthesis of 2-mer building block containing a phosphotriester linkage

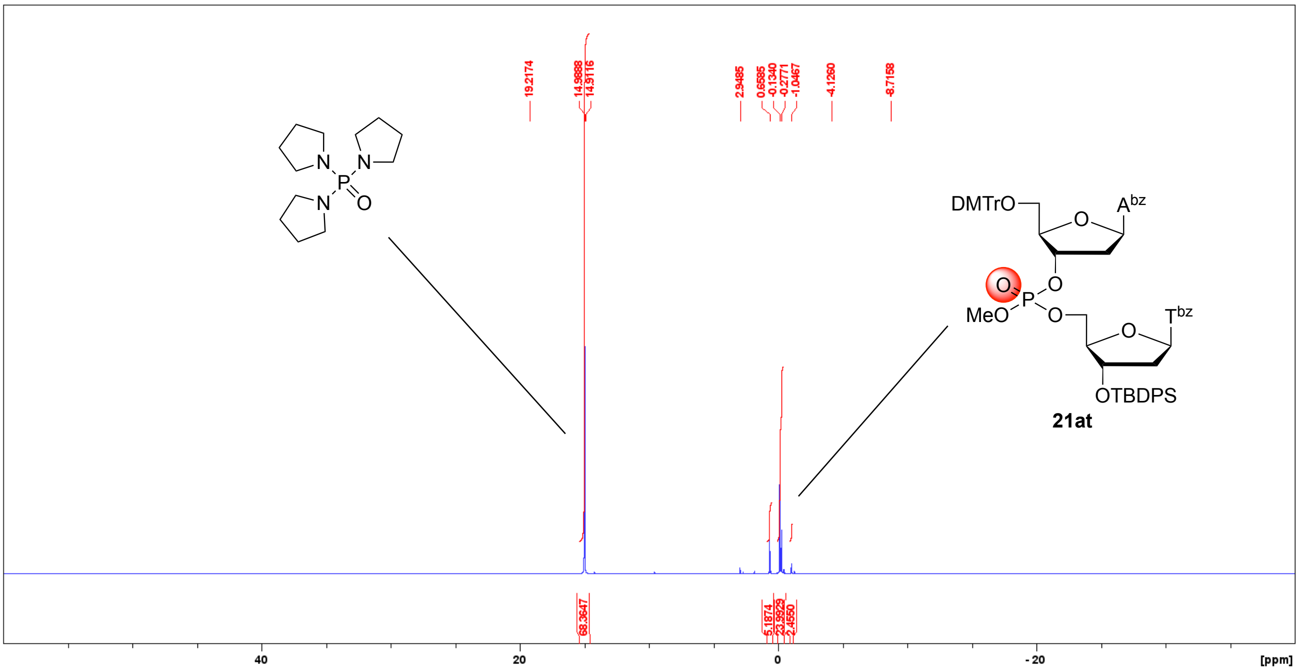

Figure S11 ^{31}P NMR spectrum of the crude mixture of **21at** after work-up of the oxidative esterification

Synthesis of 2-mer building block containing a phosphorothioate triester linkage

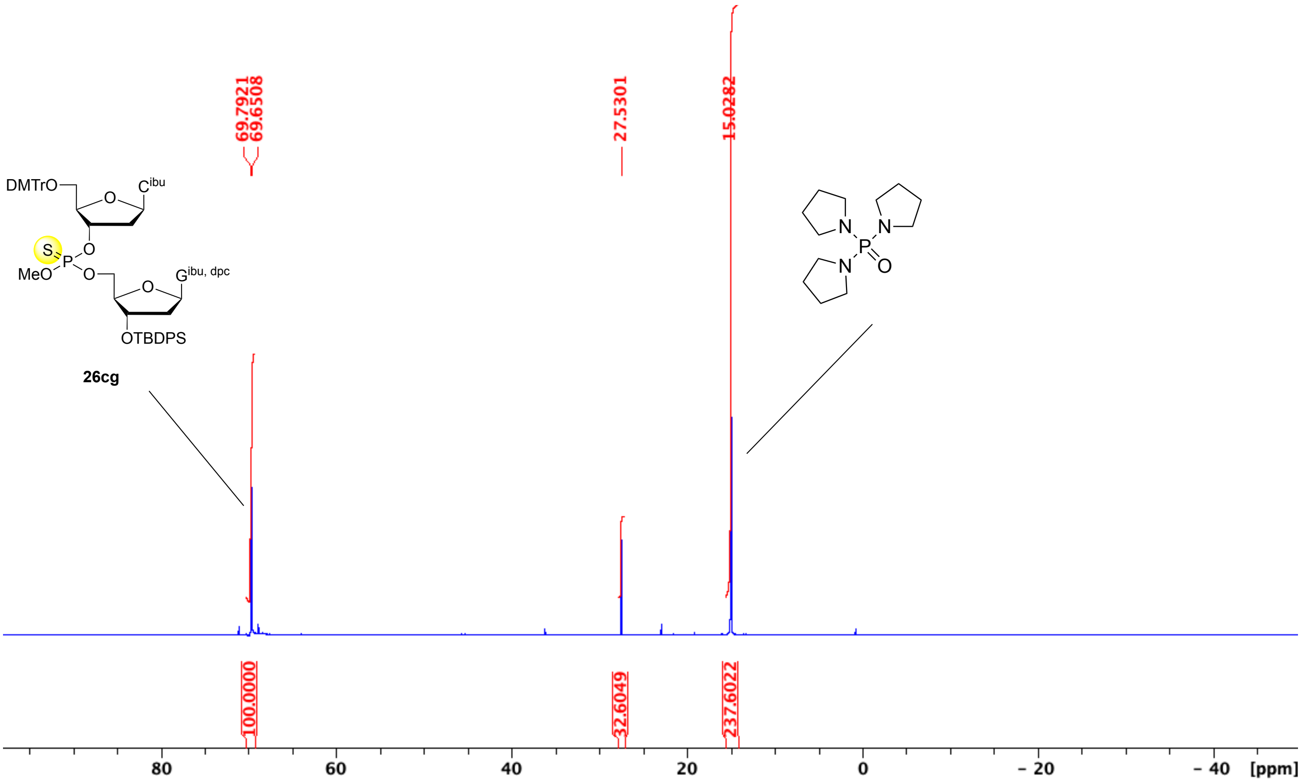

Figure S12 ^{31}P NMR analysis of the crude mixture of 26cg after work-up of the oxidative esterification

Synthesis of 4-mer building blocks containing boranophosphotriester, phosphorothioate triester, and phosphotriester linkages

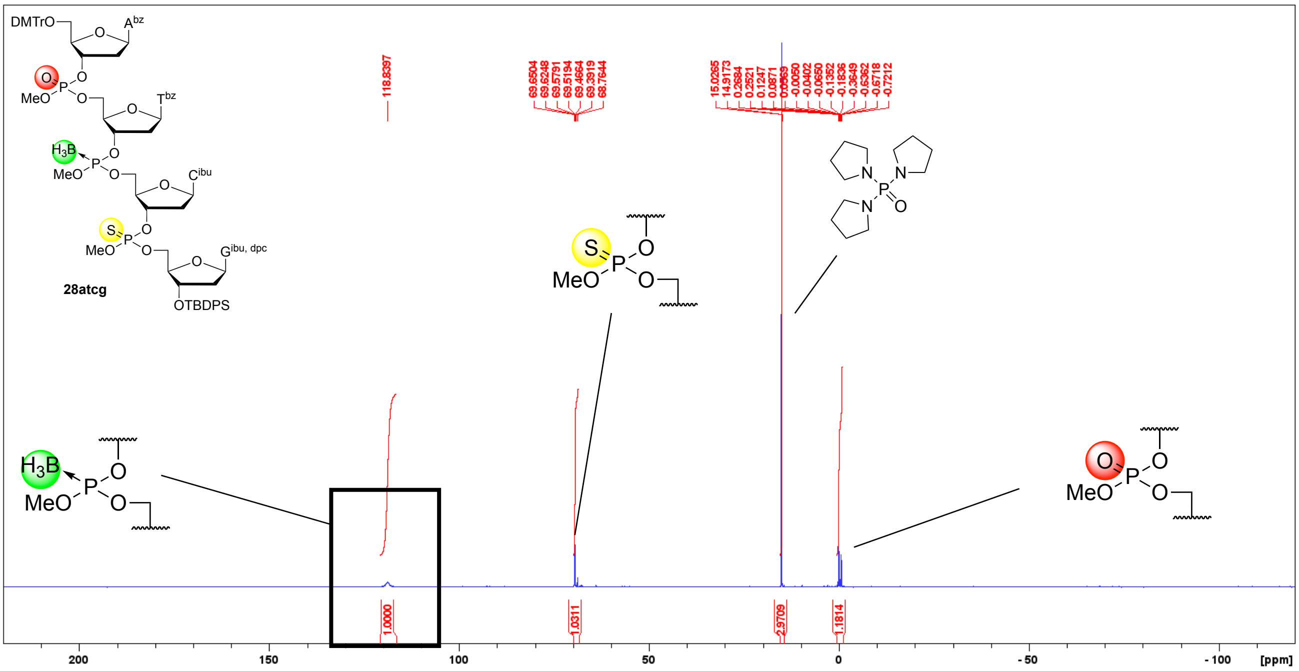

Figure S13 ^{31}P NMR analysis of the crude mixture of **28atcg** after work-up of the oxidative esterification

Synthesis of 8-mer containing boranophosphotriester, phosphorothioate triester, and phosphotriester linkages by block condensation using the 4-mer building blocks

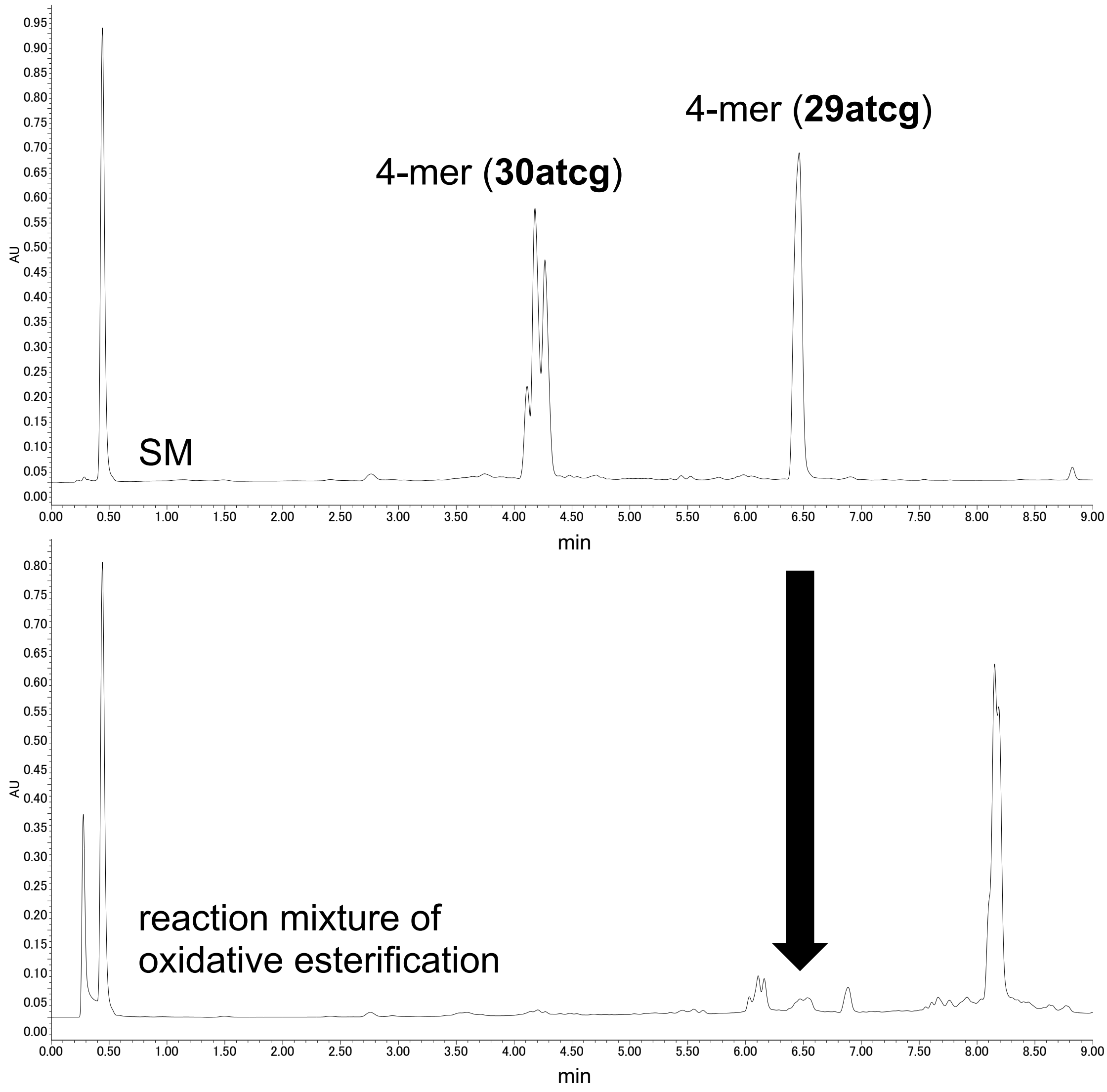

Figure S14 UHPLC profile of crude mixture after work-up of the oxidative esterification (1.5 equivalents of 5'-upstream 4-mer building block and 6.0 equivalents of PyNTP were used). UHPLC (ODS, H_2O–MeCN (9:1, v/v, containing 50 mM HFIP, 5 mM TEA) / MeCN = 60:40–0:100 over 8 min, 60 °C, flow rate = 0.5 mL/min, λ = 260 nm).

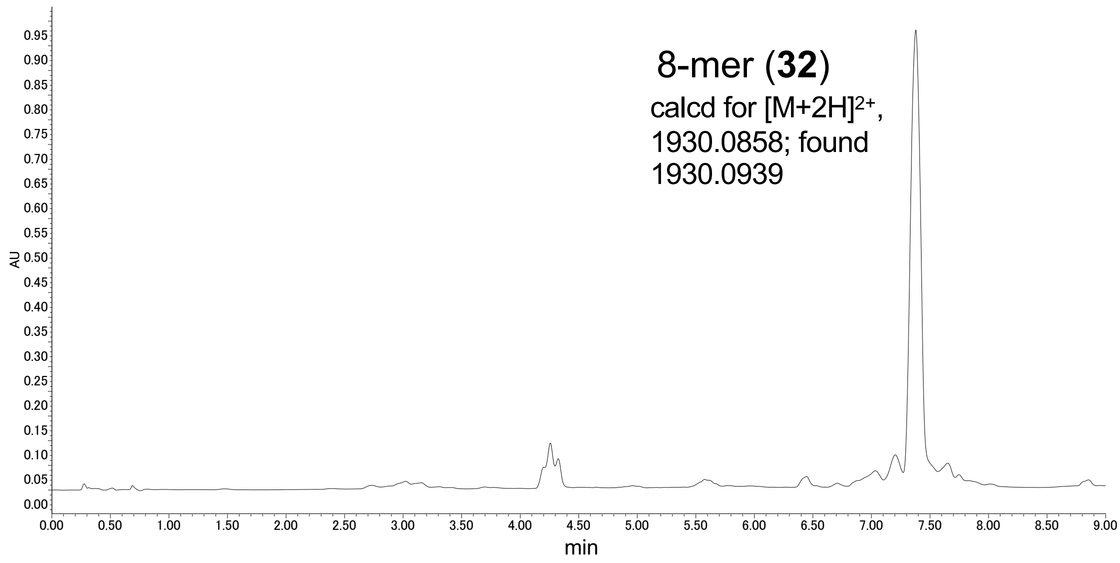

Figure S15 UHPLC profile of the purified 8-mer (**32**) through silica gel column chromatography. UHPLC (ODS, H_2O–MeCN (9:1, v/v, containing 50 mM HFIP, 5 mM TEA) / MeCN = 60:40–0:100 over 8 min, 60 °C, flow rate = 0.5 mL/min, λ = 260 nm). tR = 7.3 min (compound **32**).

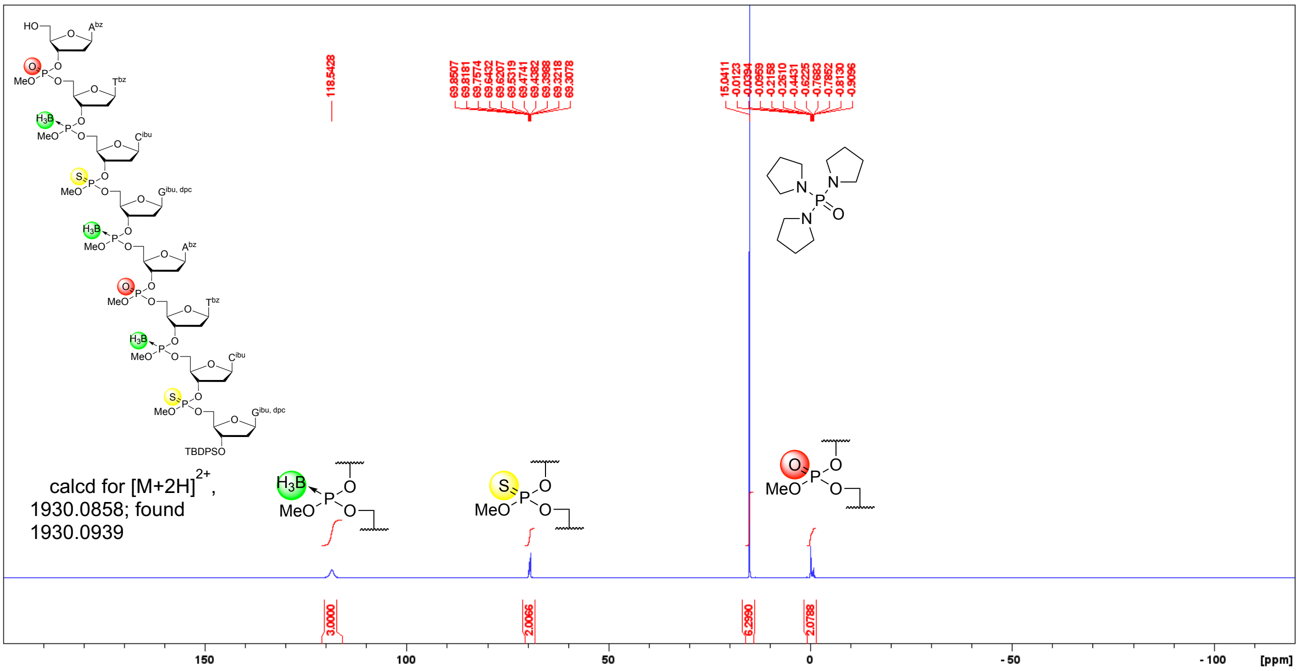

Figure S16 ^{31}P NMR spectrum of the purified compound **32** through silica gel column chromatography

Synthesis of 12-mer containing boranophosphotriester, phosphorothioate triester, and phosphotriester linkages by block condensation between 4-mer (30atcg) and 8-mer (32)

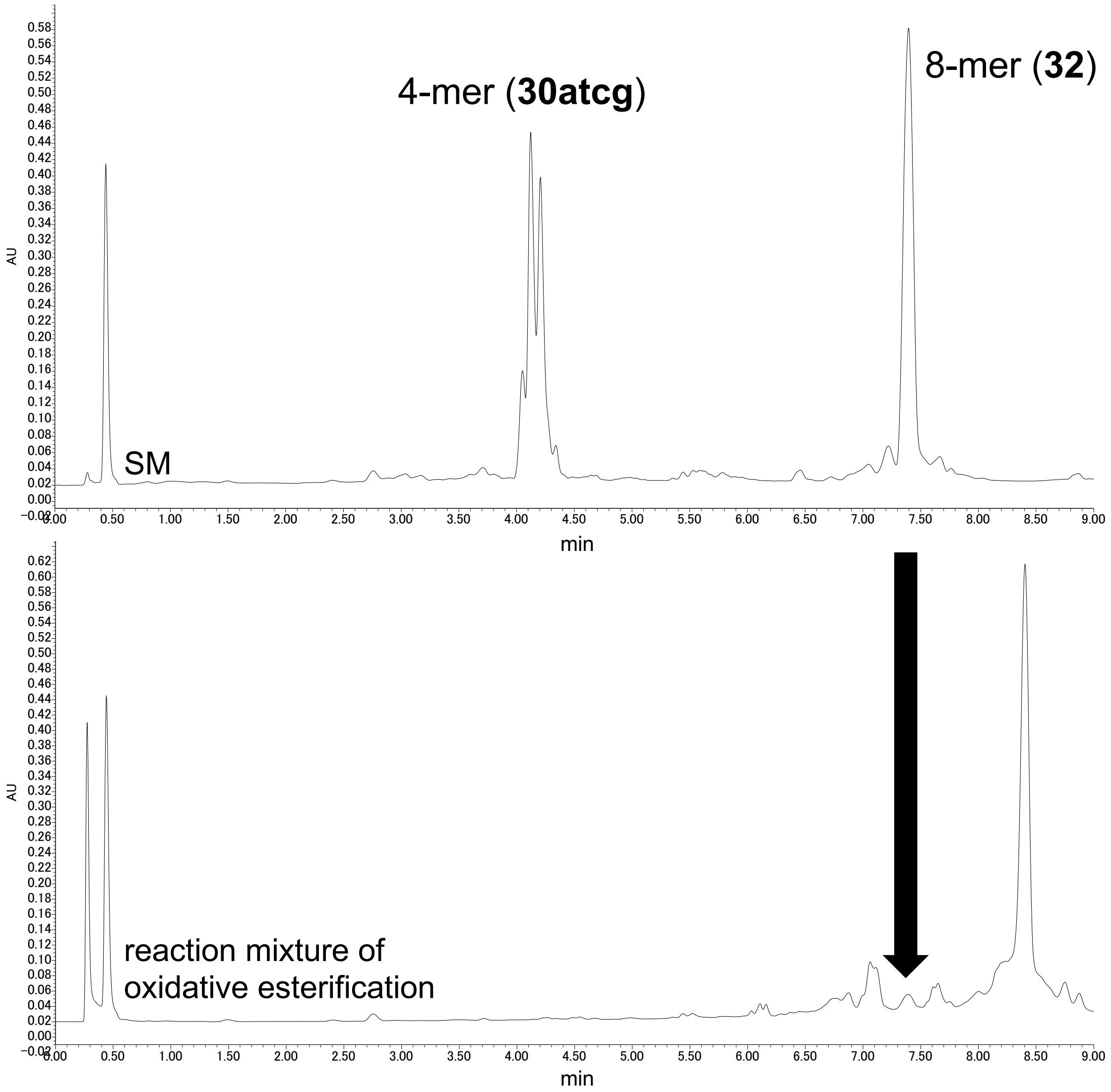

Figure S17 UHPLC profile of the reaction mixture after block condensation and oxidative esterification between 4-mer and 8-mer.UHPLC (ODS, H_2O–MeCN (9:1, v/v, containing 50 mM HFIP, 5 mM TEA) / MeCN = 60:40–0:100 over 8 min, 60 °C, flow rate = 0.5 mL/min, λ = 260 nm).

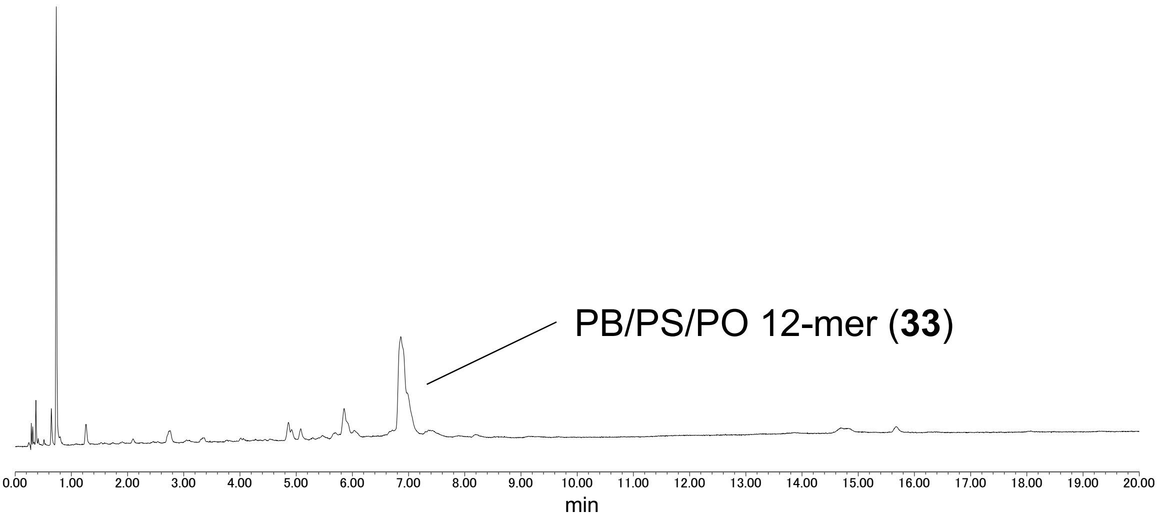

Figure S18 UPLC profile of the crude mixture of PB/PS/PO 12-mer (**33**) after removal of all protecting groups. UPLC (ODS, H_2O (containing 50 mM HFIP, 5 mM TEA) / H_2O–MeCN (1:1, v/v, containing 50 mM HFIP, 5 mM TEA) = 90:10–15:85 over 20 min, 60 °C, flow rate = 0.5 mL/min, λ = 260 nm). tR = 6.9 min (main peak).

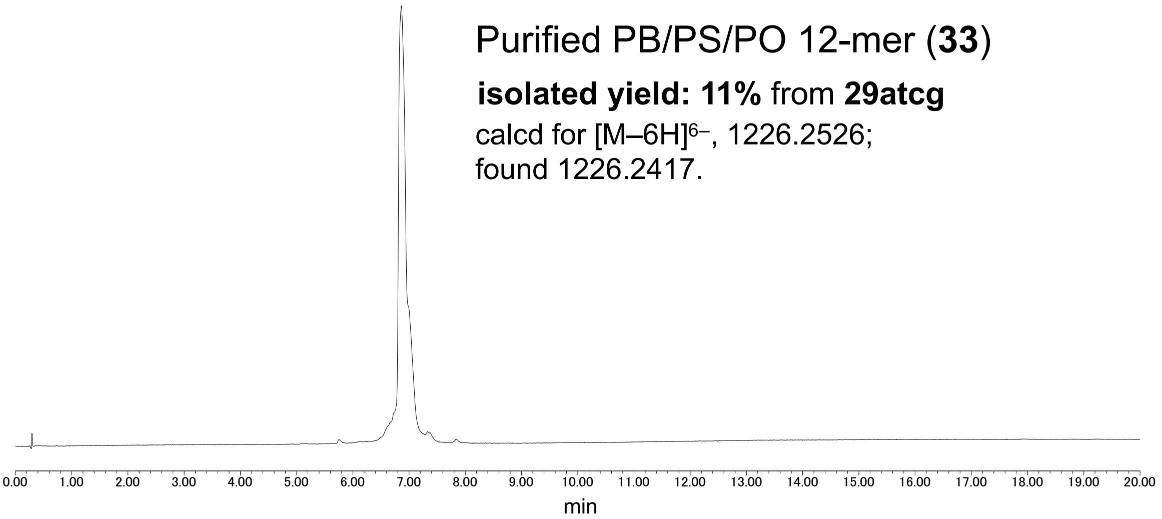

Figure S19 UPLC profile of the purified PB/PS/PO 12-mer (**33**) after removal of all protecting groups. UPLC (ODS, H_2O (containing 50 mM HFIP, 5 mM TEA) / H_2O–MeCN (1:1, v/v, containing 50 mM HFIP, 5 mM TEA) = 90:10–15:85 over 20 min, 60 °C, flow rate = 0.5 mL/min, λ = 260 nm). tR = 6.9 min (compound **33**).

NMR spectra of new compounds

3'-*O*-TBDPS-*N*[6]-benzoyl-adenosine (1a)

^{1}H NMR ($CDCl_3$, 500 MHz)

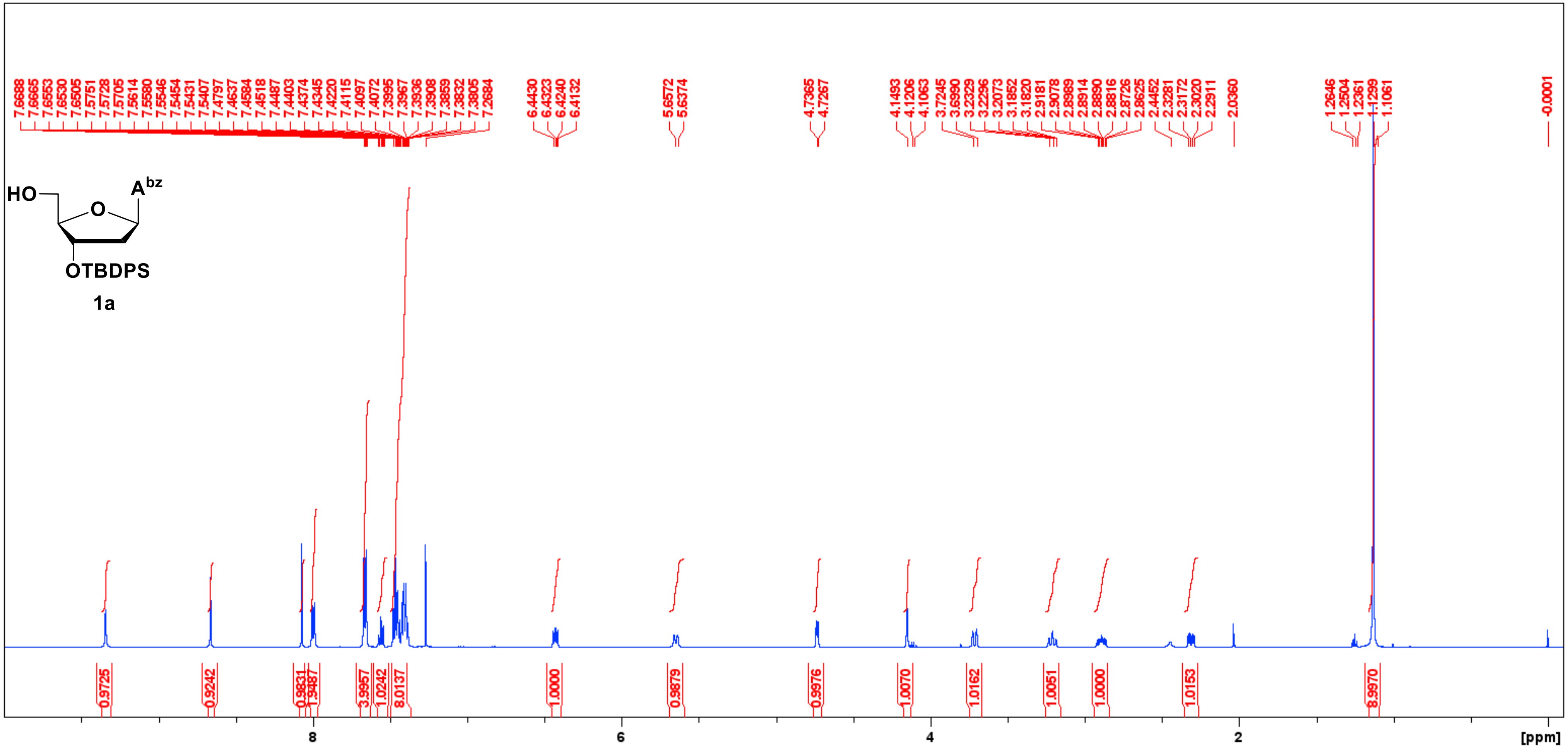

3'-*O*-TBDPS-*N*[4]-isobutyryl-cytosine (1c)

^{1}H NMR ($CDCl_3$, 500 MHz)

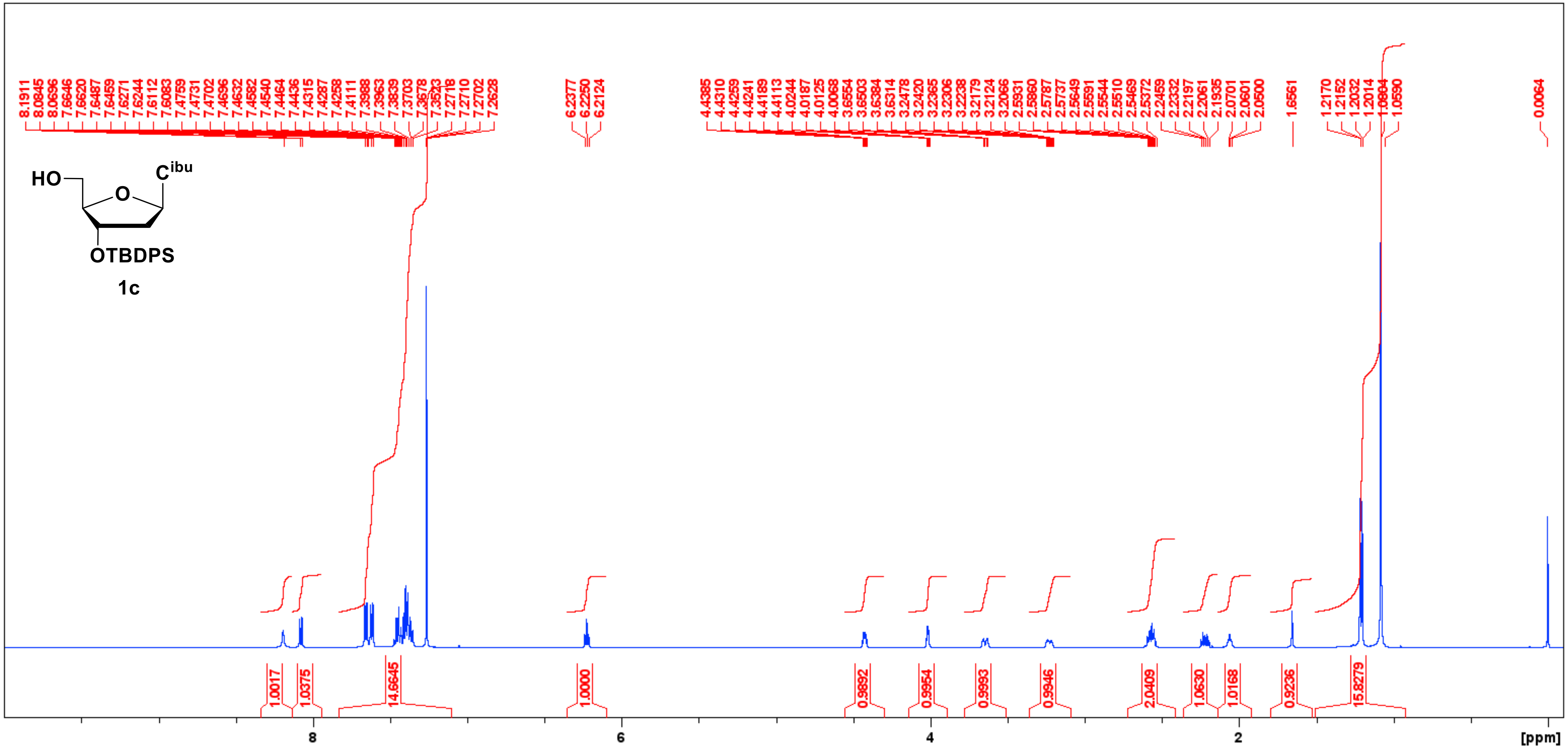

5'-O-DMTr- N^3-benzoyl-thymidine 3'-H-boranophosphonate monomer (2t)

^{1}H NMR ($CDCl_3$, 500 MHz)

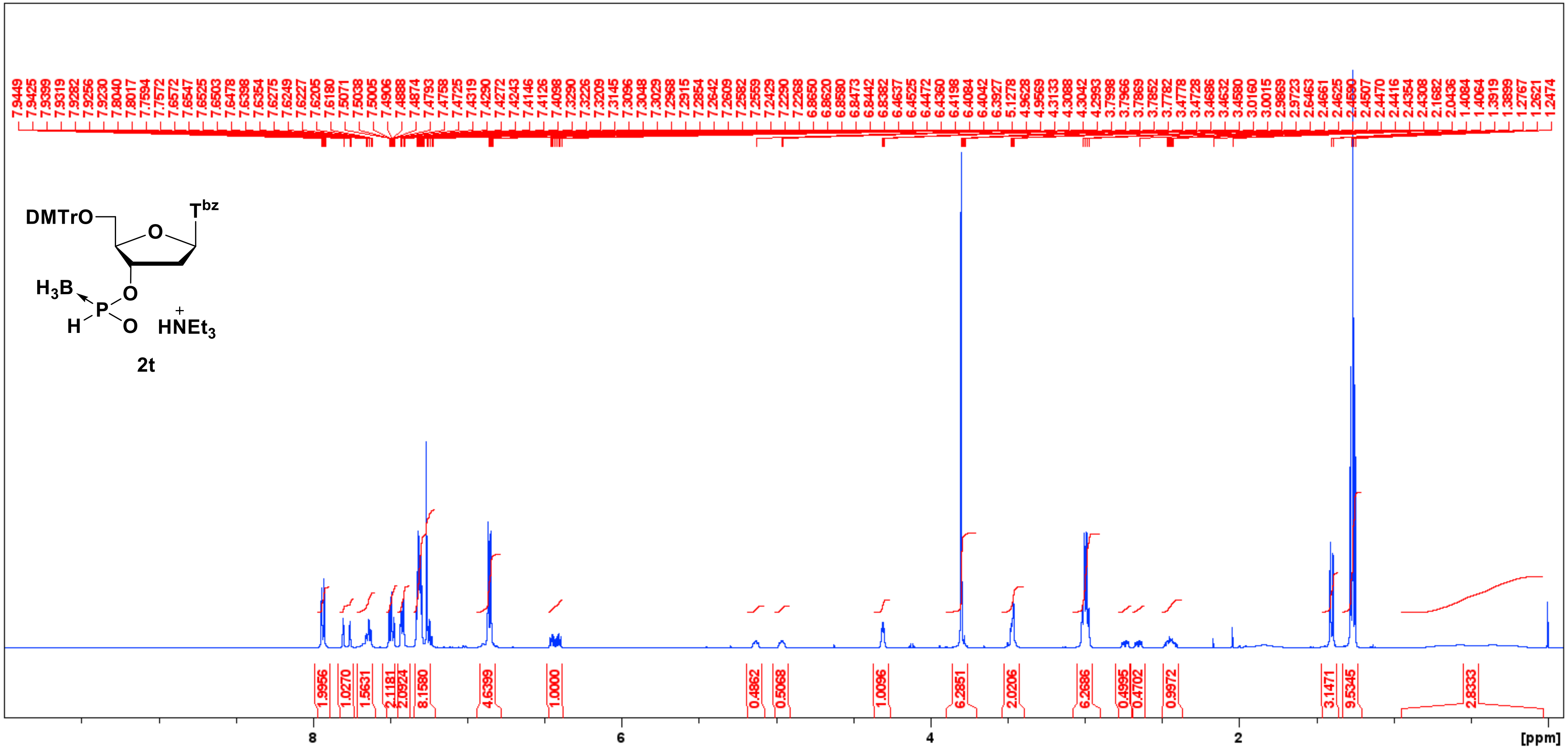

5'-*O*-DMTr- *N*^{3}-benzoyl-thymidine 3'-*H*-boranophosphonate monomer (2t)

$^{31}P\{^1H\}$ NMR ($CDCl_3$, 202 MHz)

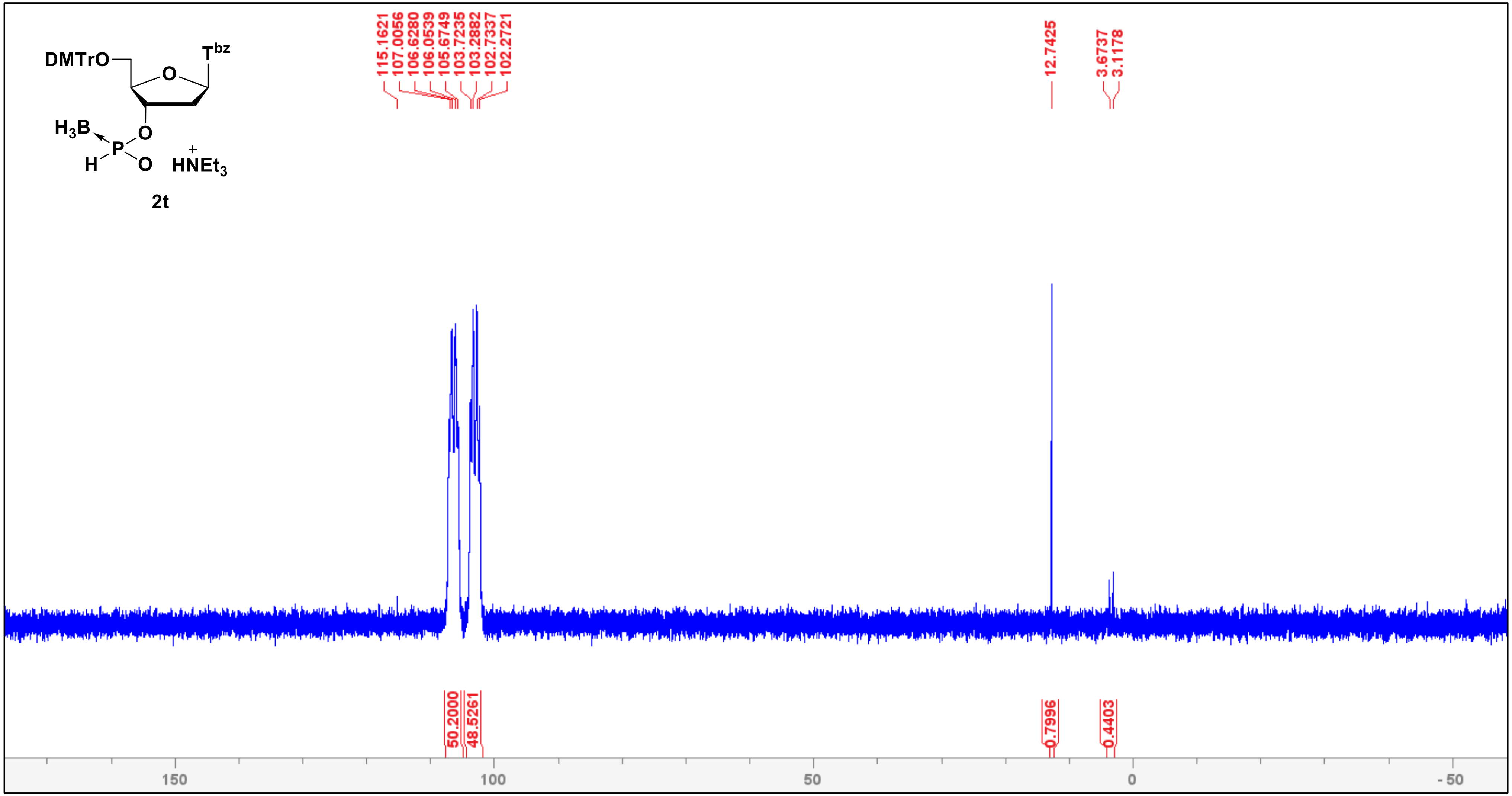

3'-*O*-TBDPS-*N*[3]-benzoyl-thymidine (1t)

^{1}H NMR ($CDCl_3$, 500 MHz)

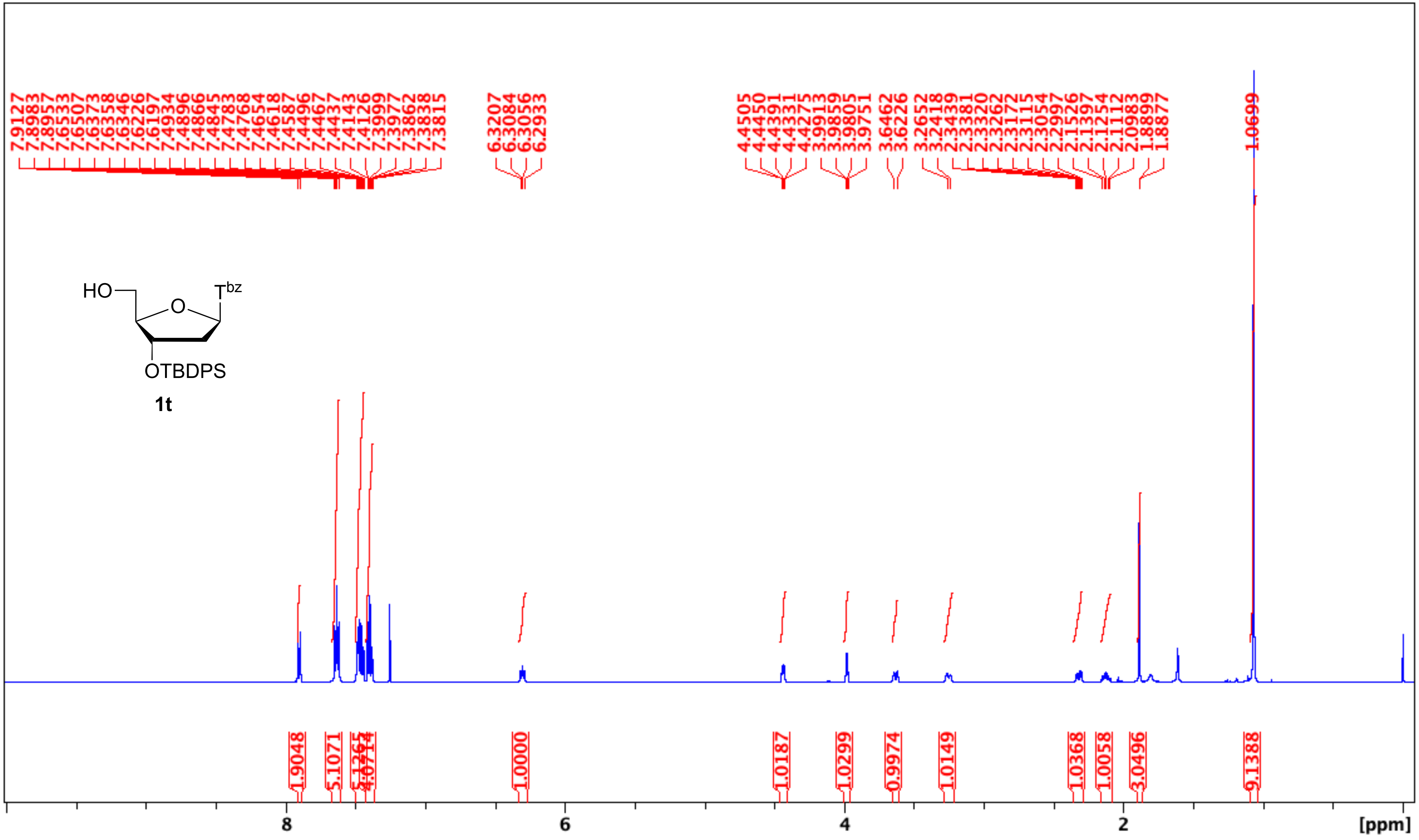

$^{13}C\{^{1}H\}$ NMR ($CDCl_3$, 126 MHz)

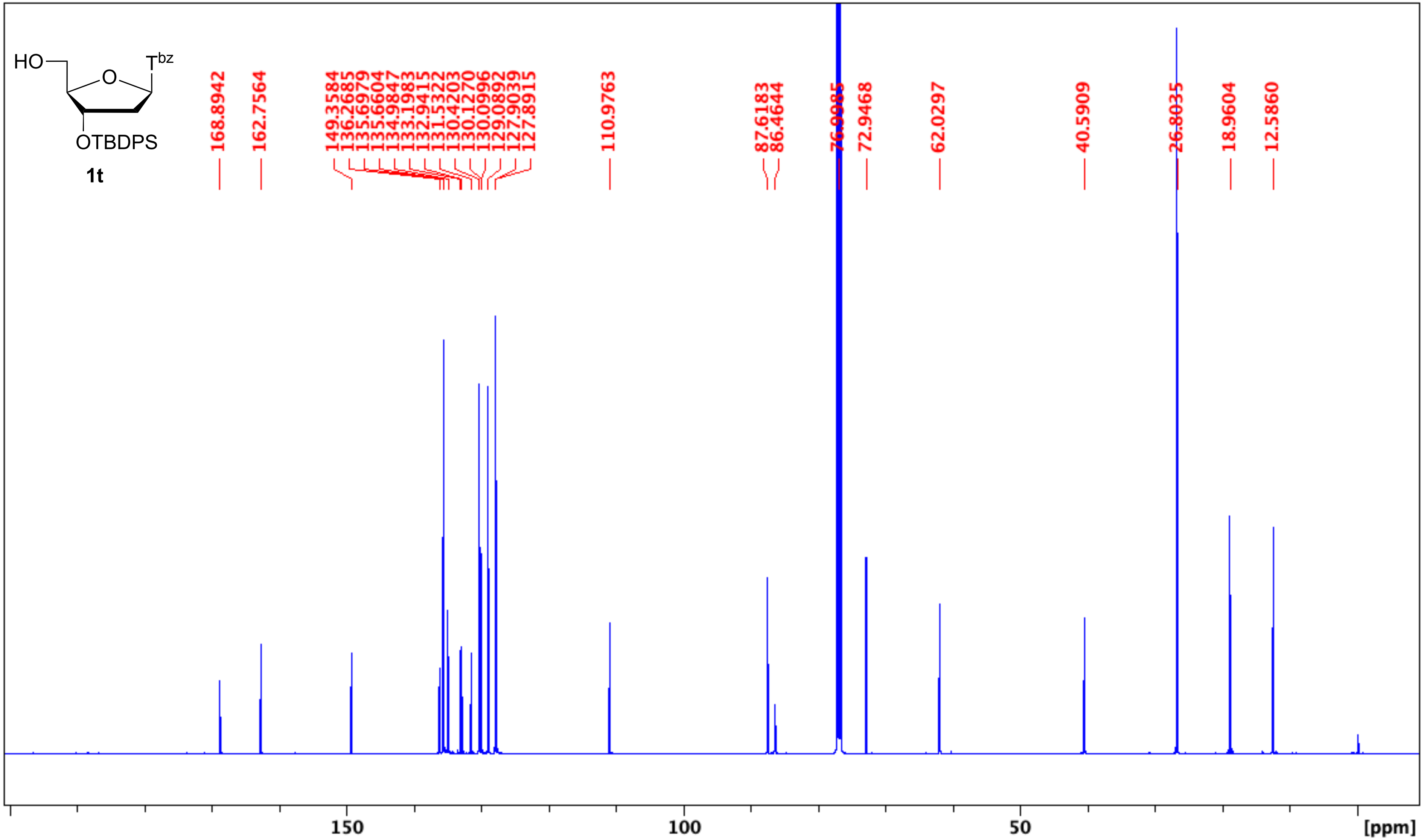

COSY ($CDCl_3$)

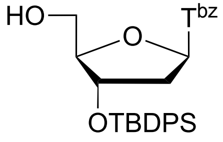

1t

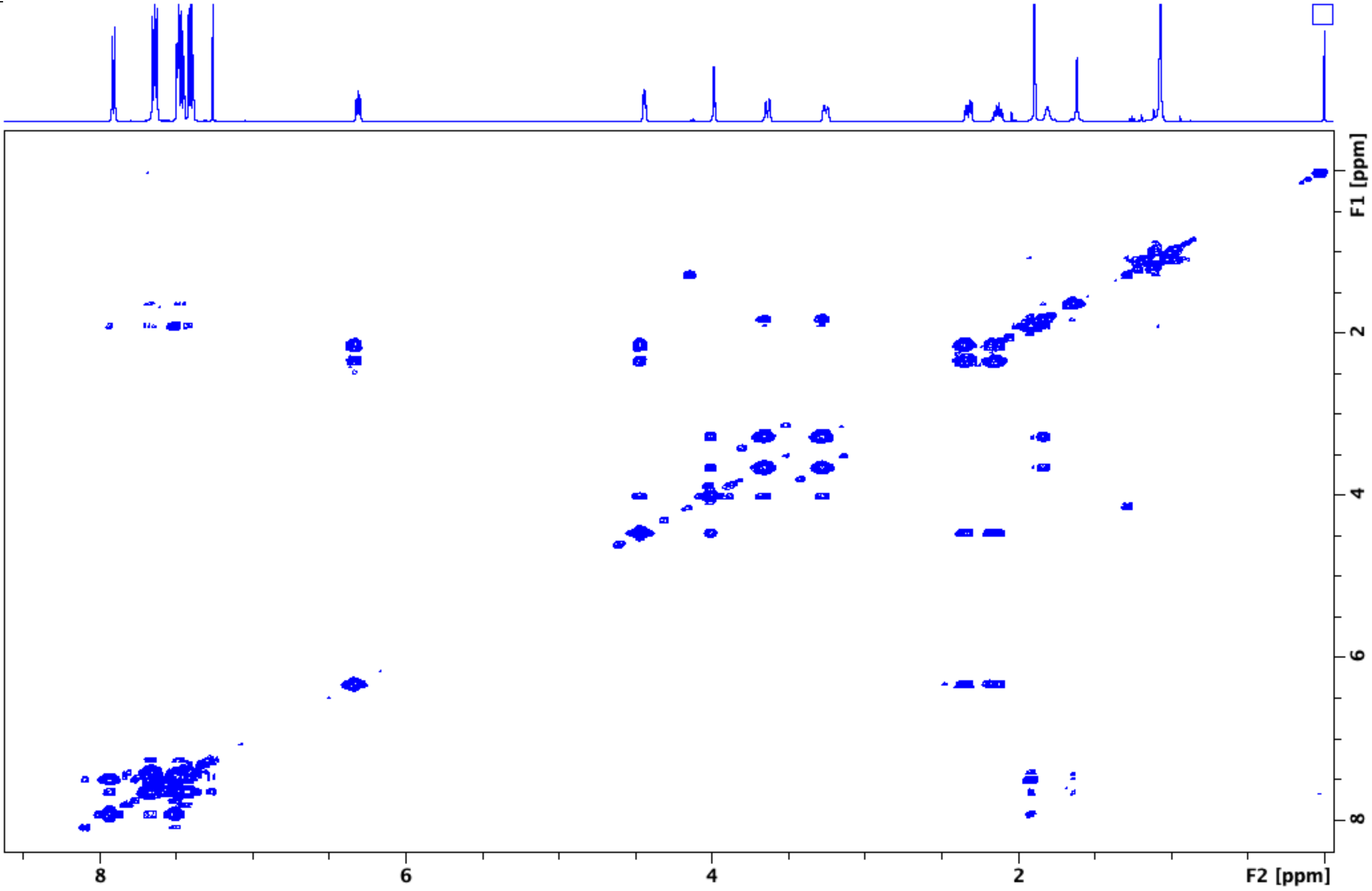

HSQC (CDCl₃)

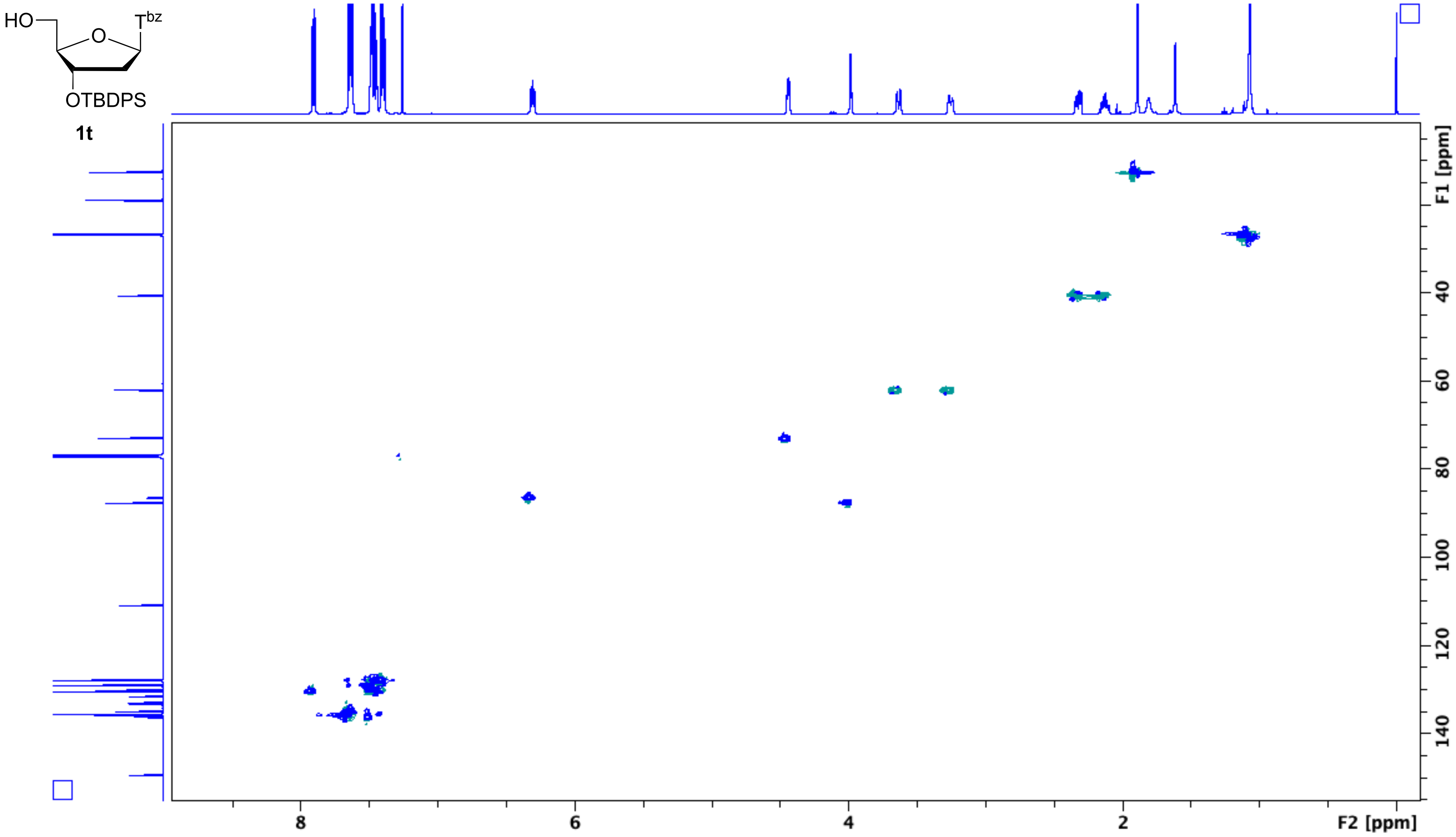

HMBC ($CDCl_3$)

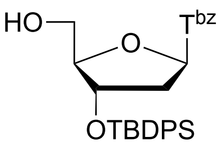

1t

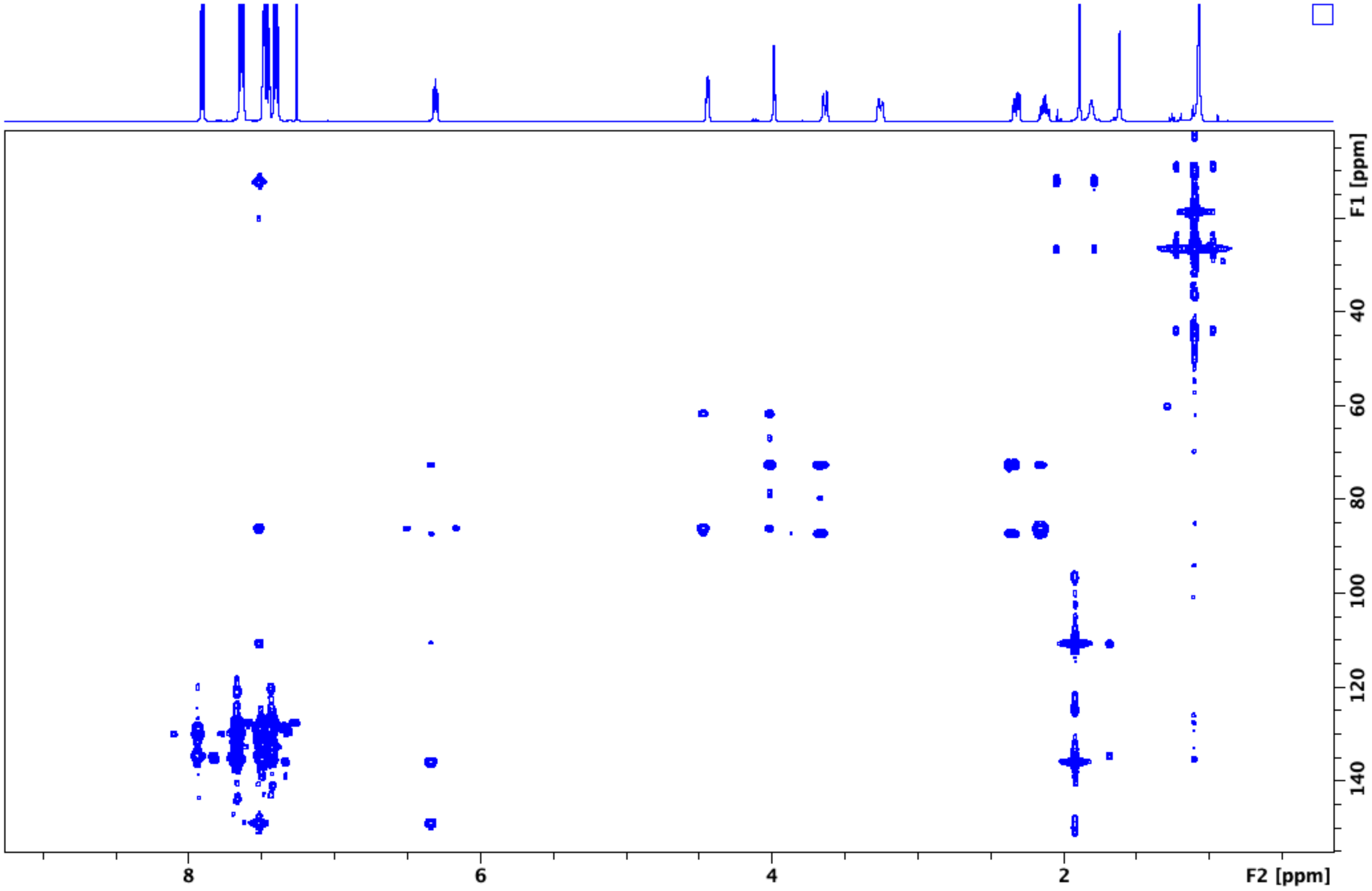

5'-*O*-DMTr-*N*²-isobutyryl-*O*⁶-diphenyl carbamoyl-deoxyguanosine (S1g)

^{1}H NMR ($CDCl_3$, 500 MHz)

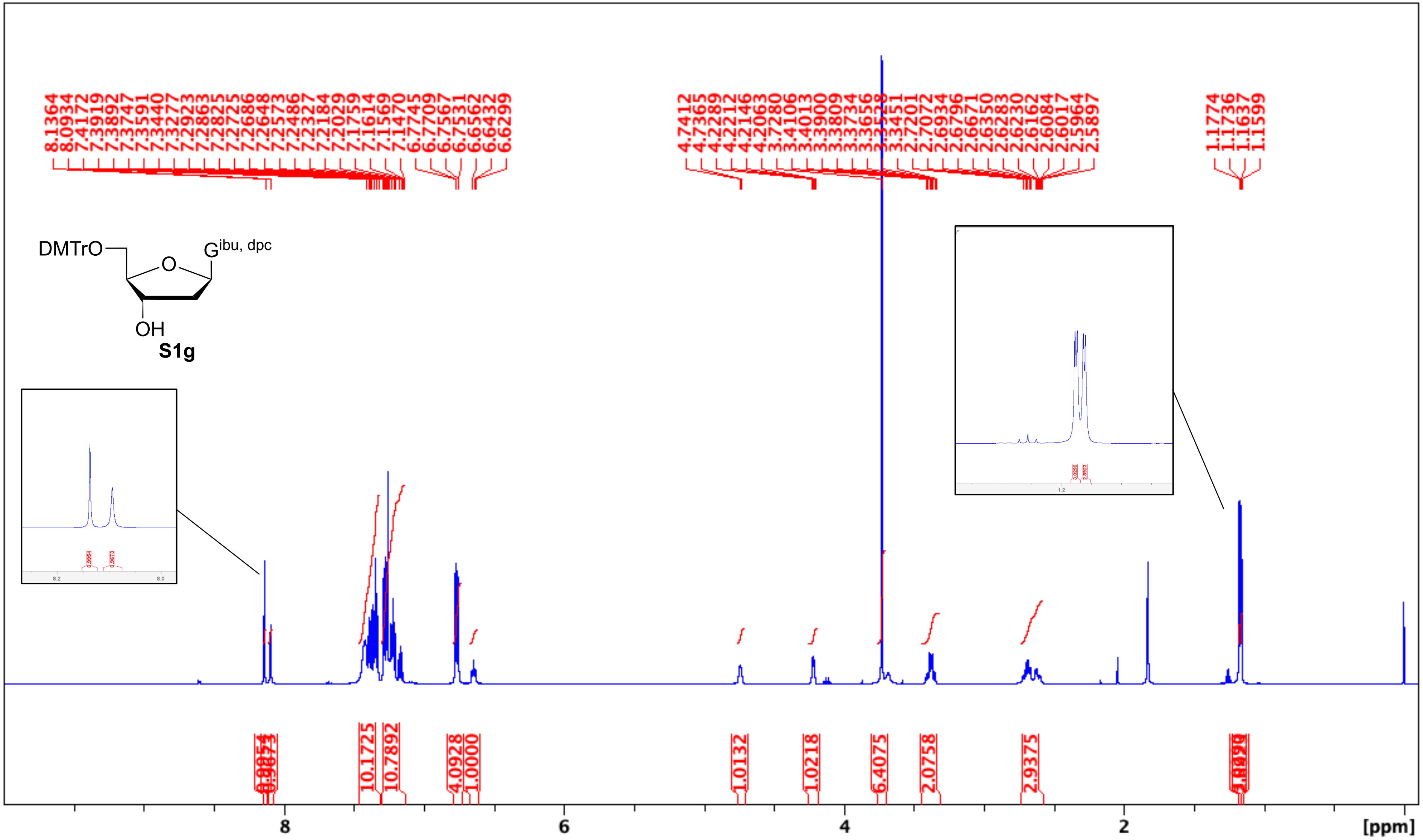

$^{13}C\{^1H\}$ NMR ($CDCl_3$, 126 MHz)

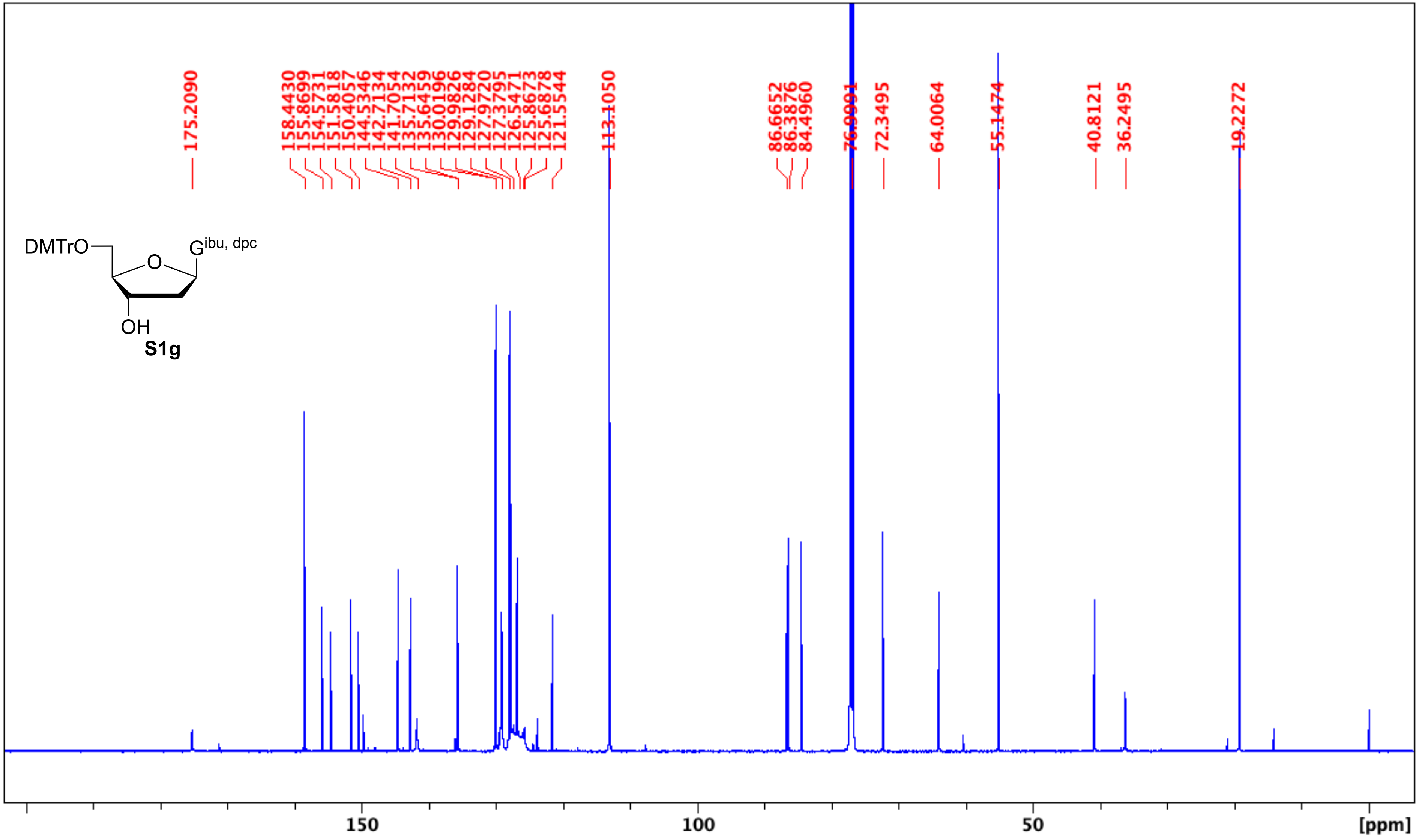

COSY (CDCl3)

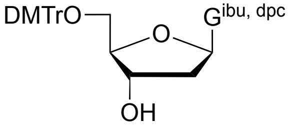

S1g

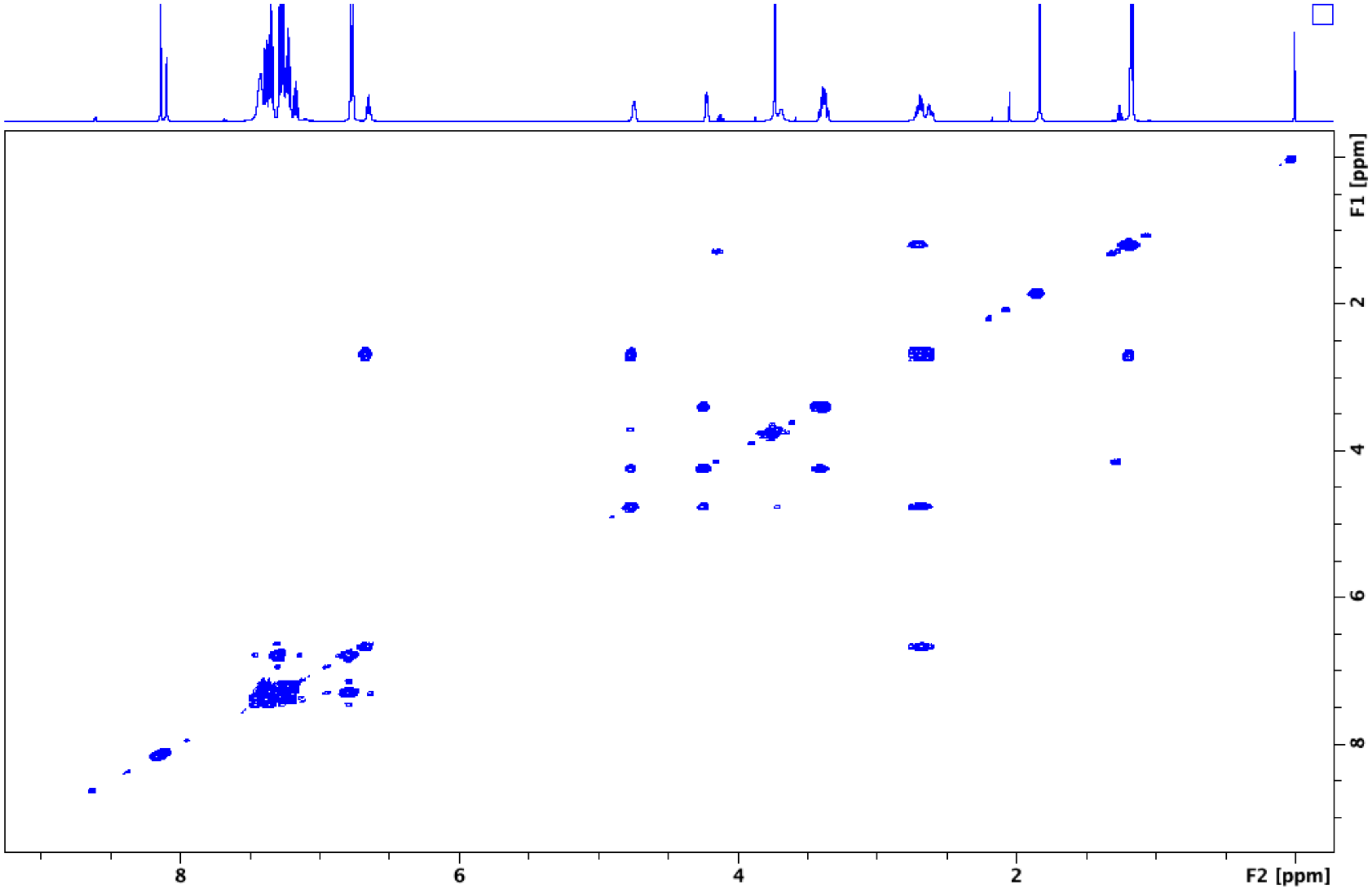

HSQC ($CDCl_3$)

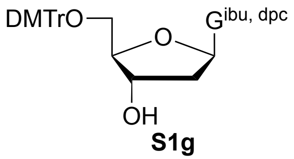

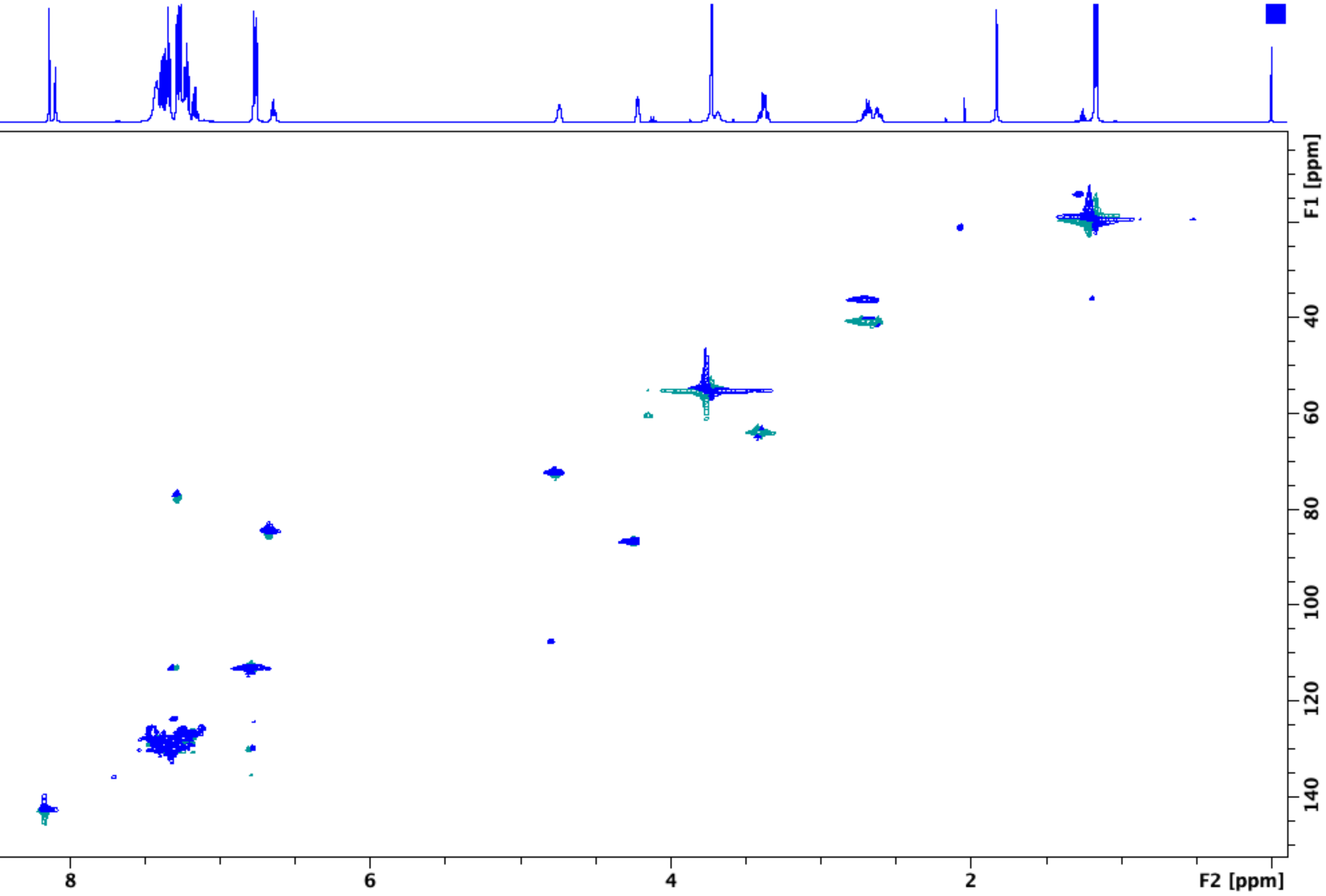

HMBC ($CDCl_3$)

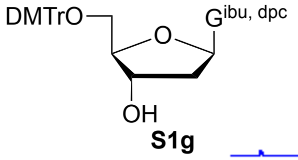

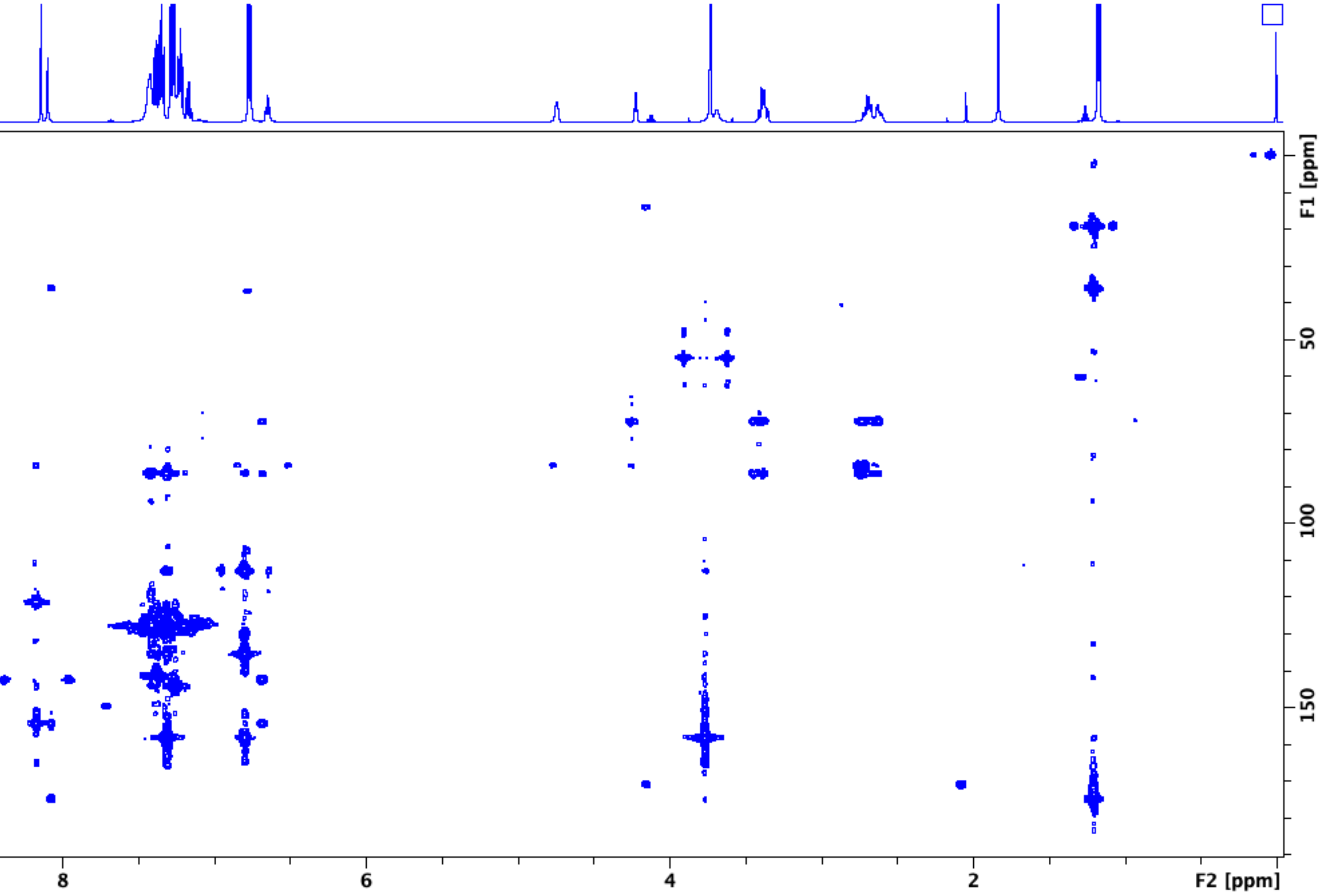

3'-*O*-TBDPS-*N*[2]-isobutyryl-*O*[6]-diphenyl carbamoyl-deoxyguanosine (1g)

^{1}H NMR ($CDCl_3$, 500 MHz)

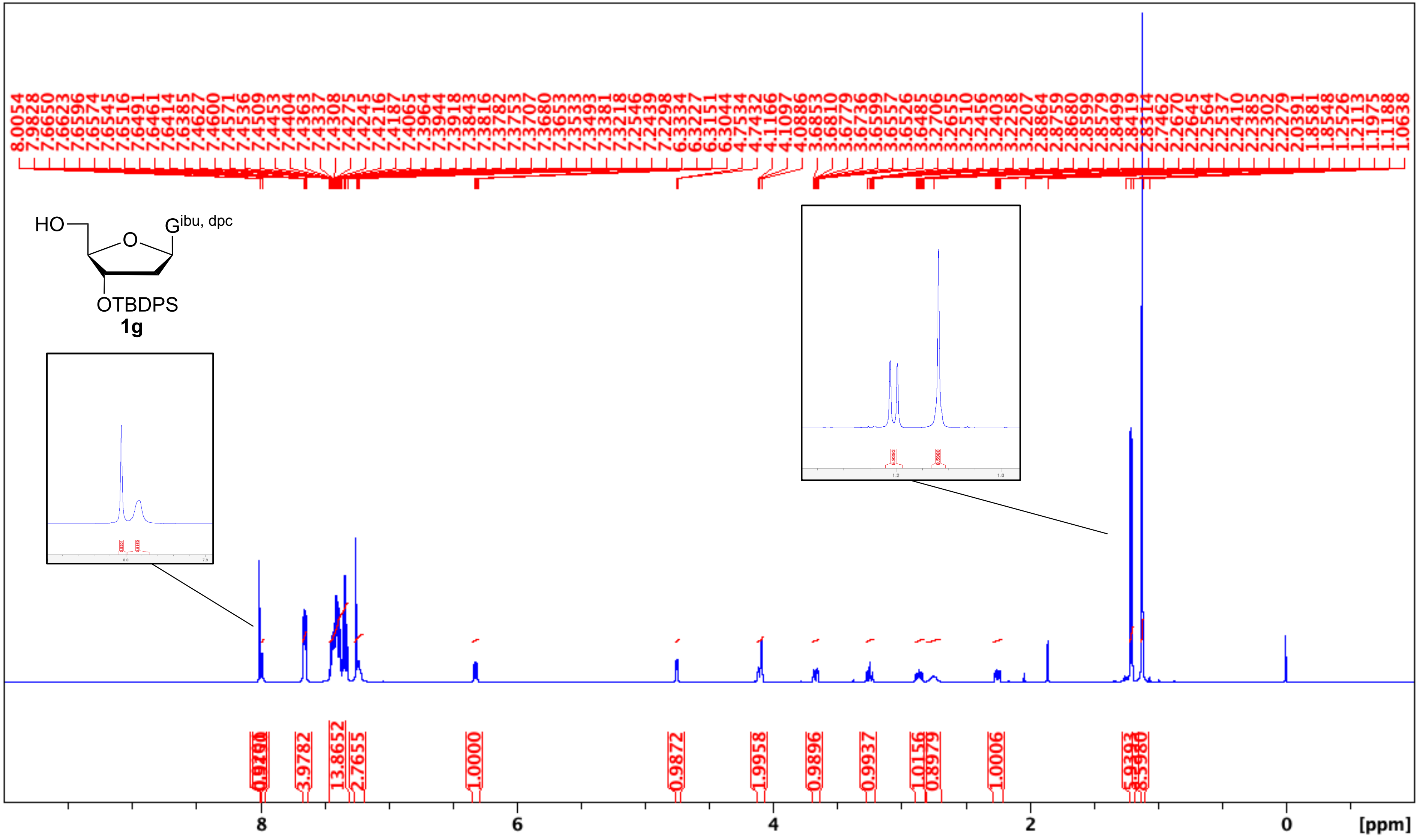

$^{13}C\{^{1}H\}$ NMR ($CDCl_3$, 126 MHz)

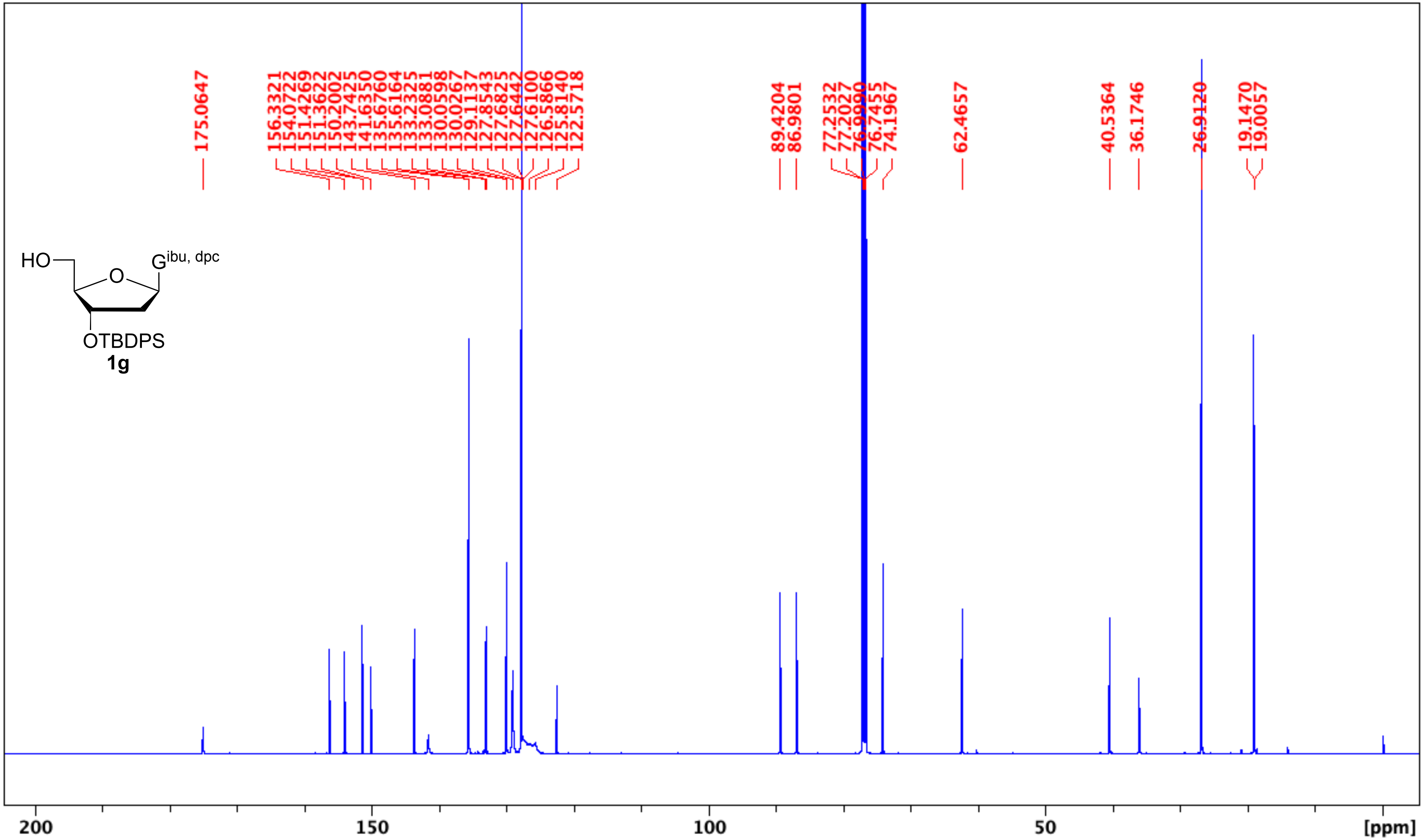

COSY ($CDCl_3$)

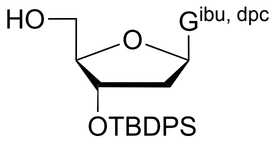

1g

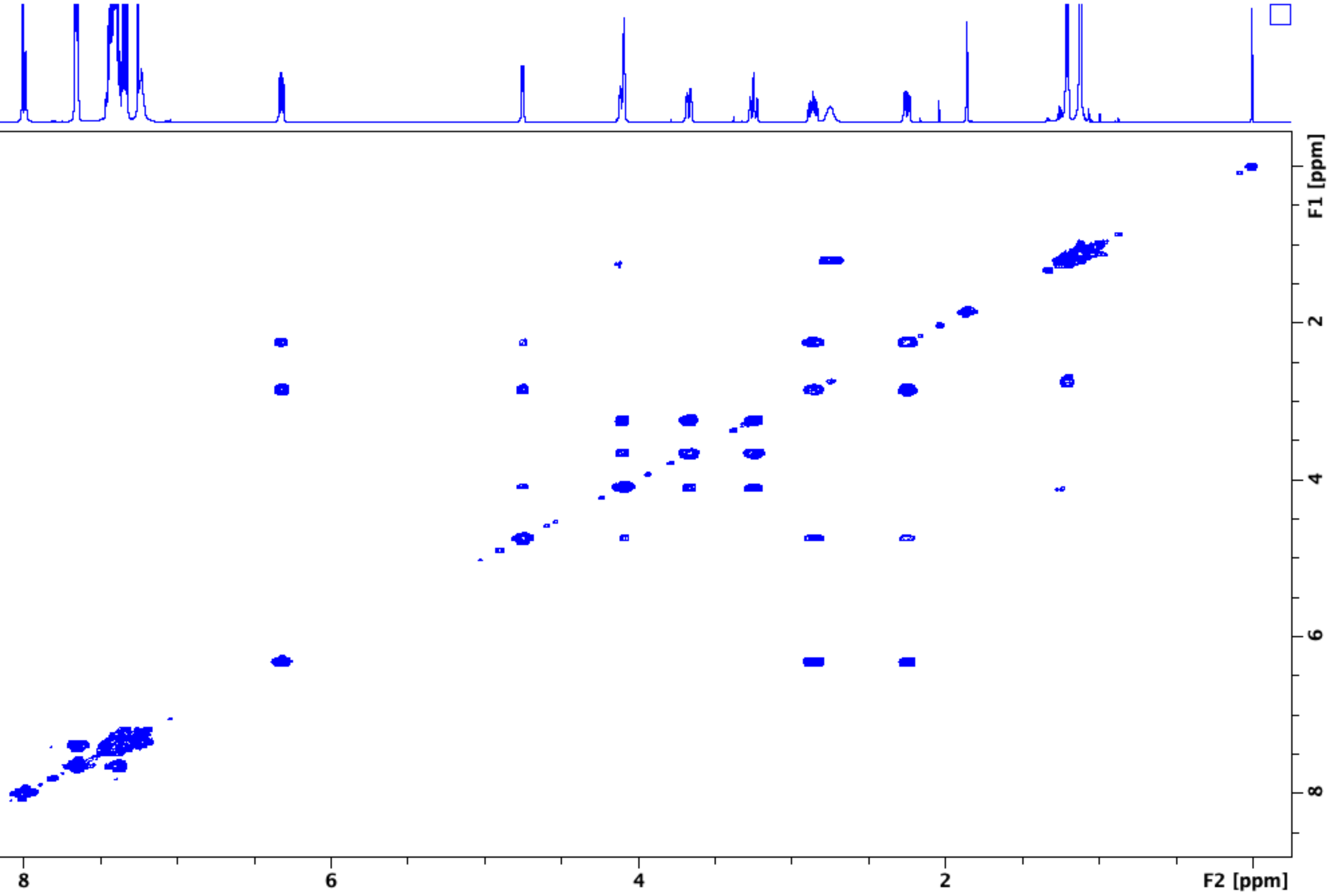

HSQC ($CDCl_3$)

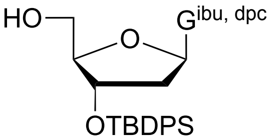

1g

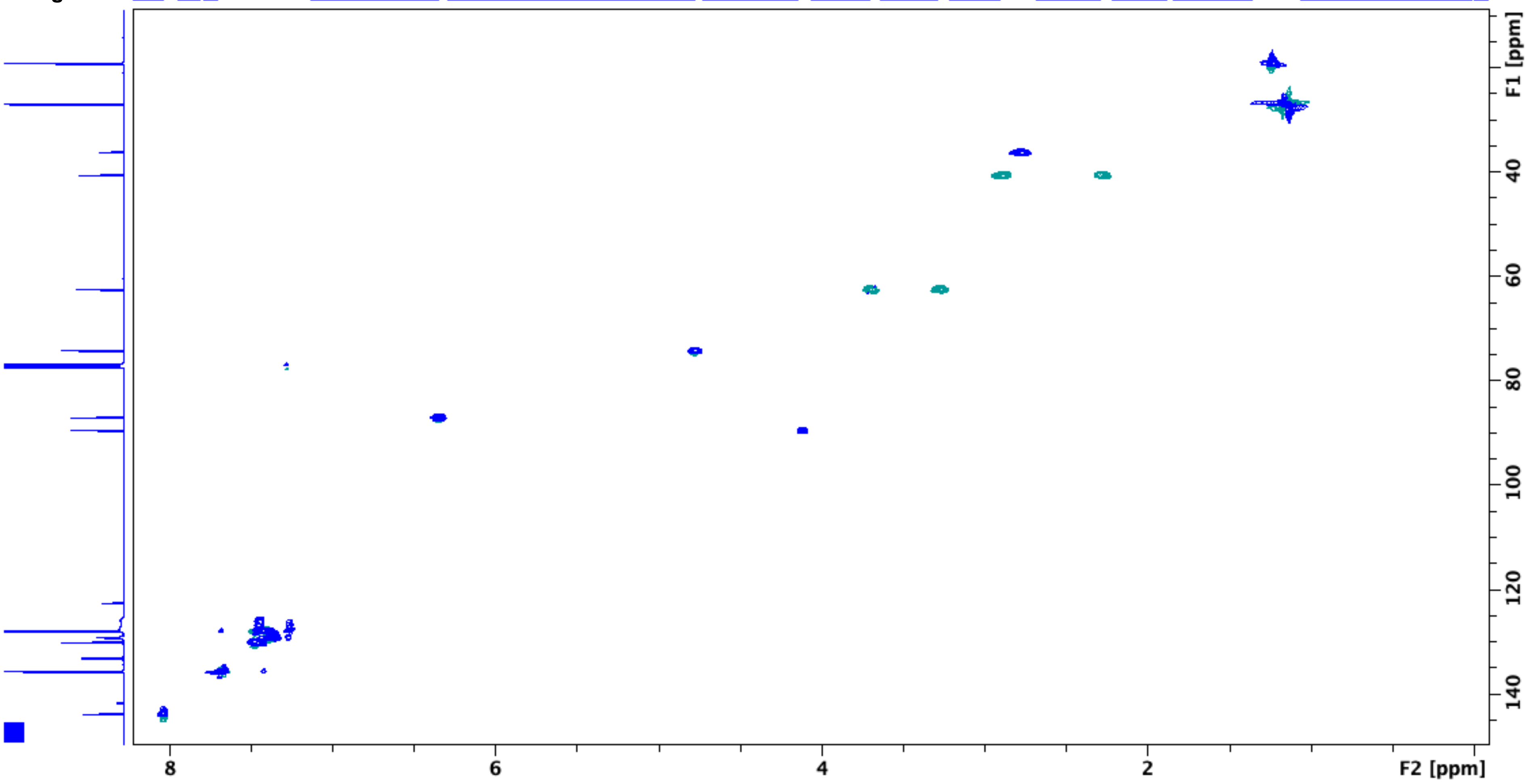

HMBC ($CDCl_3$)

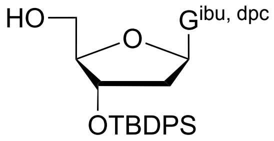

1g

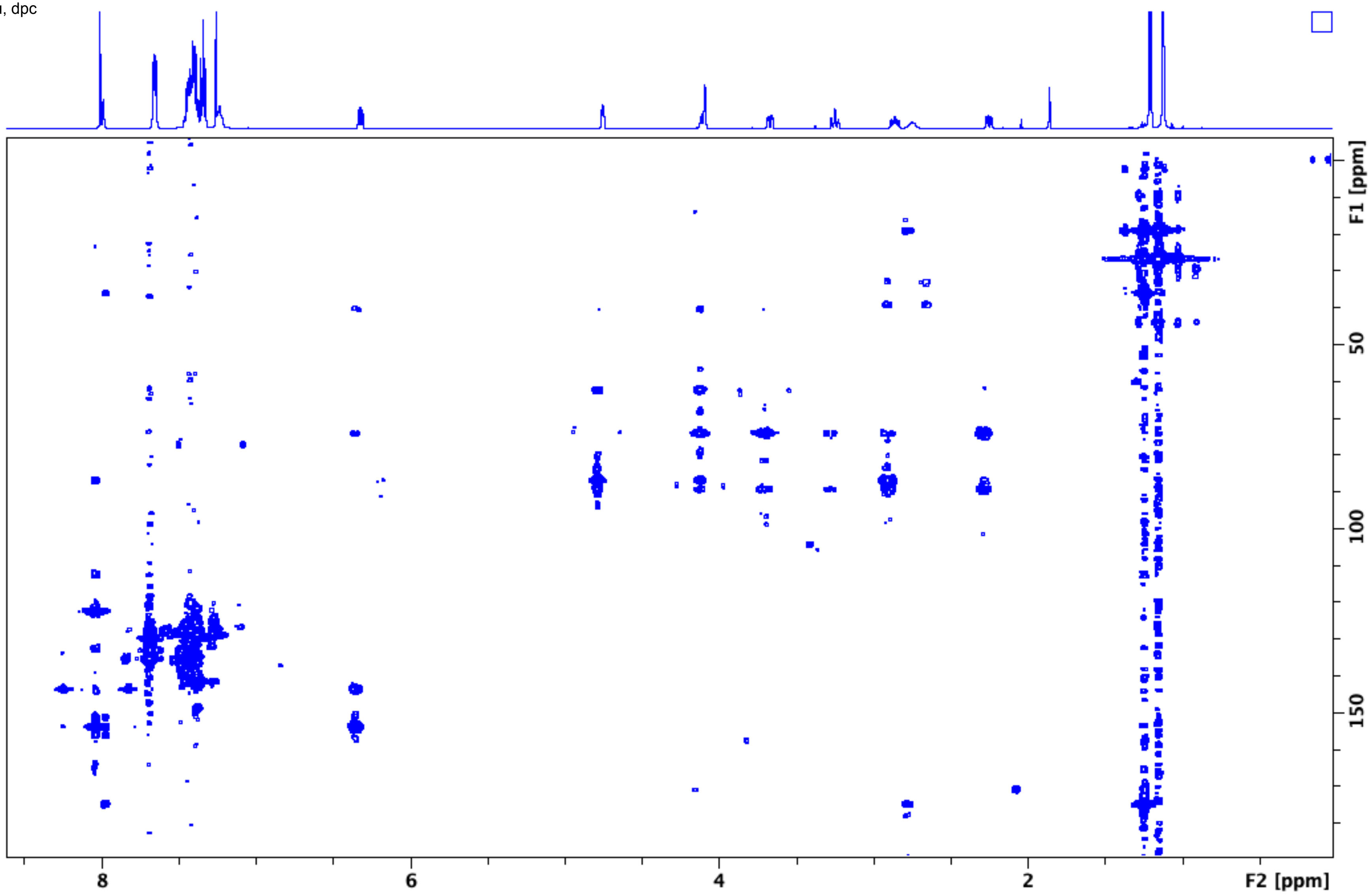

5'-*O*-DMTr-*N*^{2}-isobutyryl-*O*^{6}-diphenyl carbamoyl-deoxyguanosine 3'-*H*-boranophosphonate monomer (2g)

^{1}H NMR ($CDCl_3$, 500 MHz)

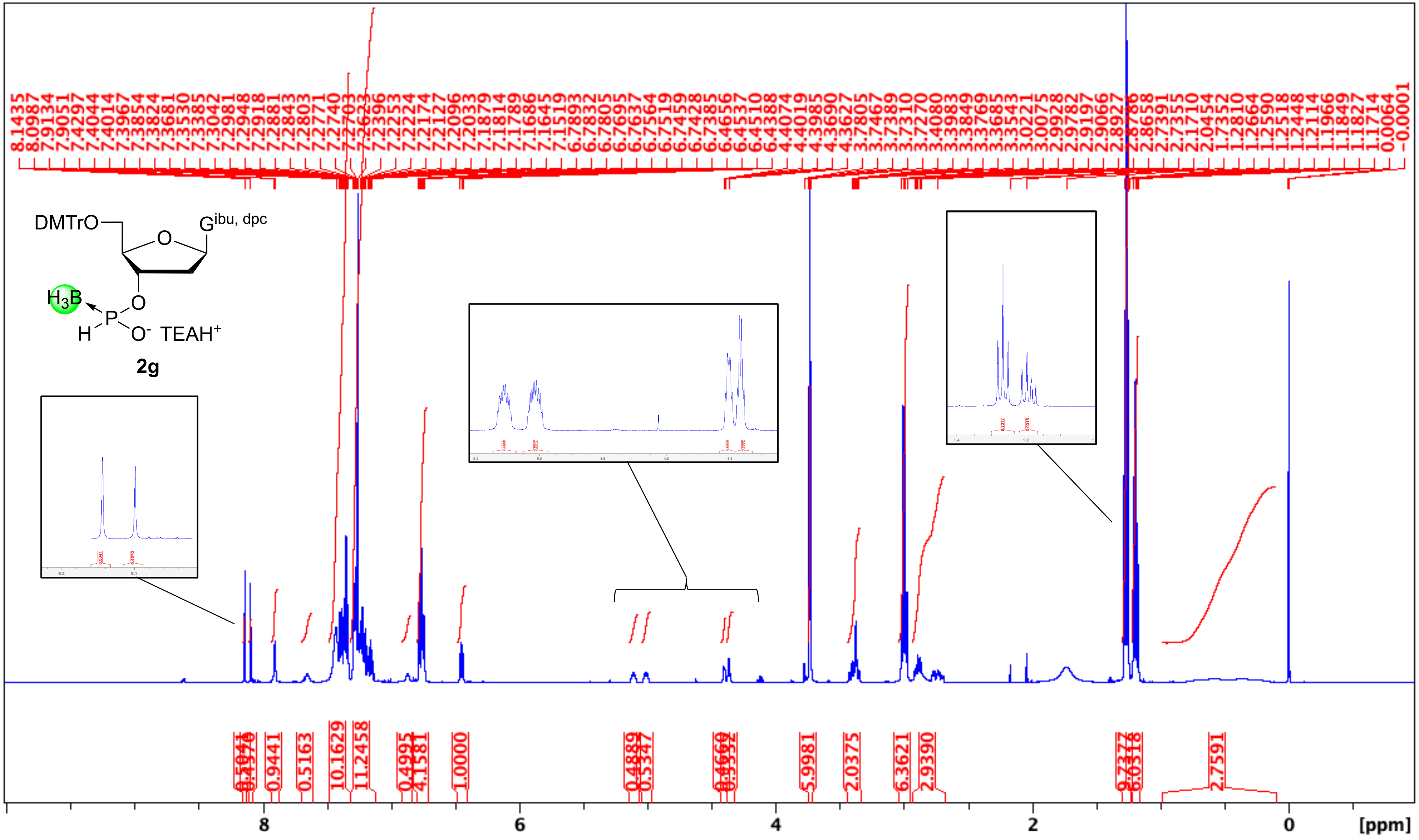

$^{13}C\{^1H\}$ NMR ($CDCl_3$, 126 MHz)

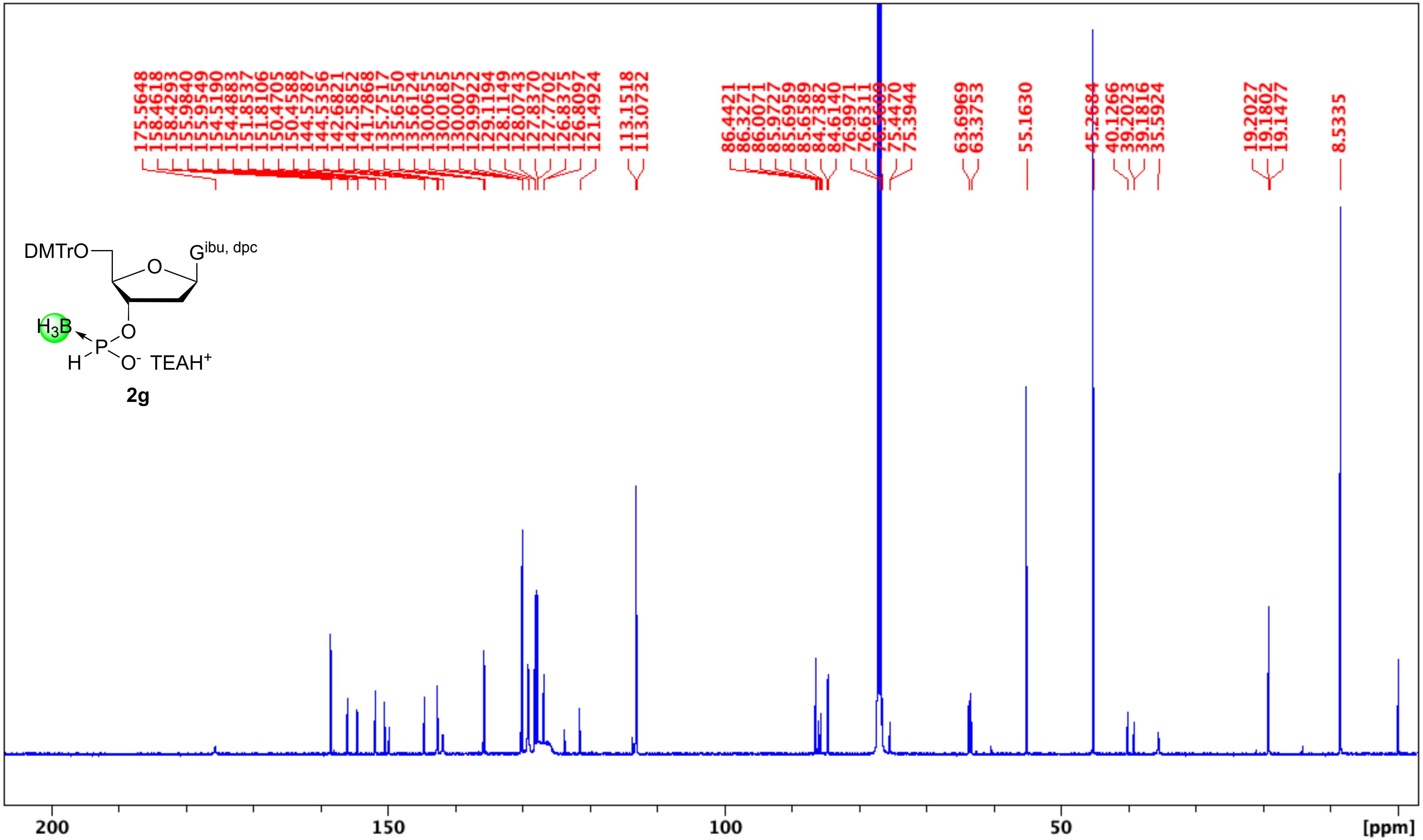

COSY ($CDCl_3$)

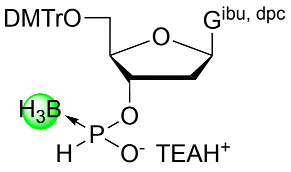

2g

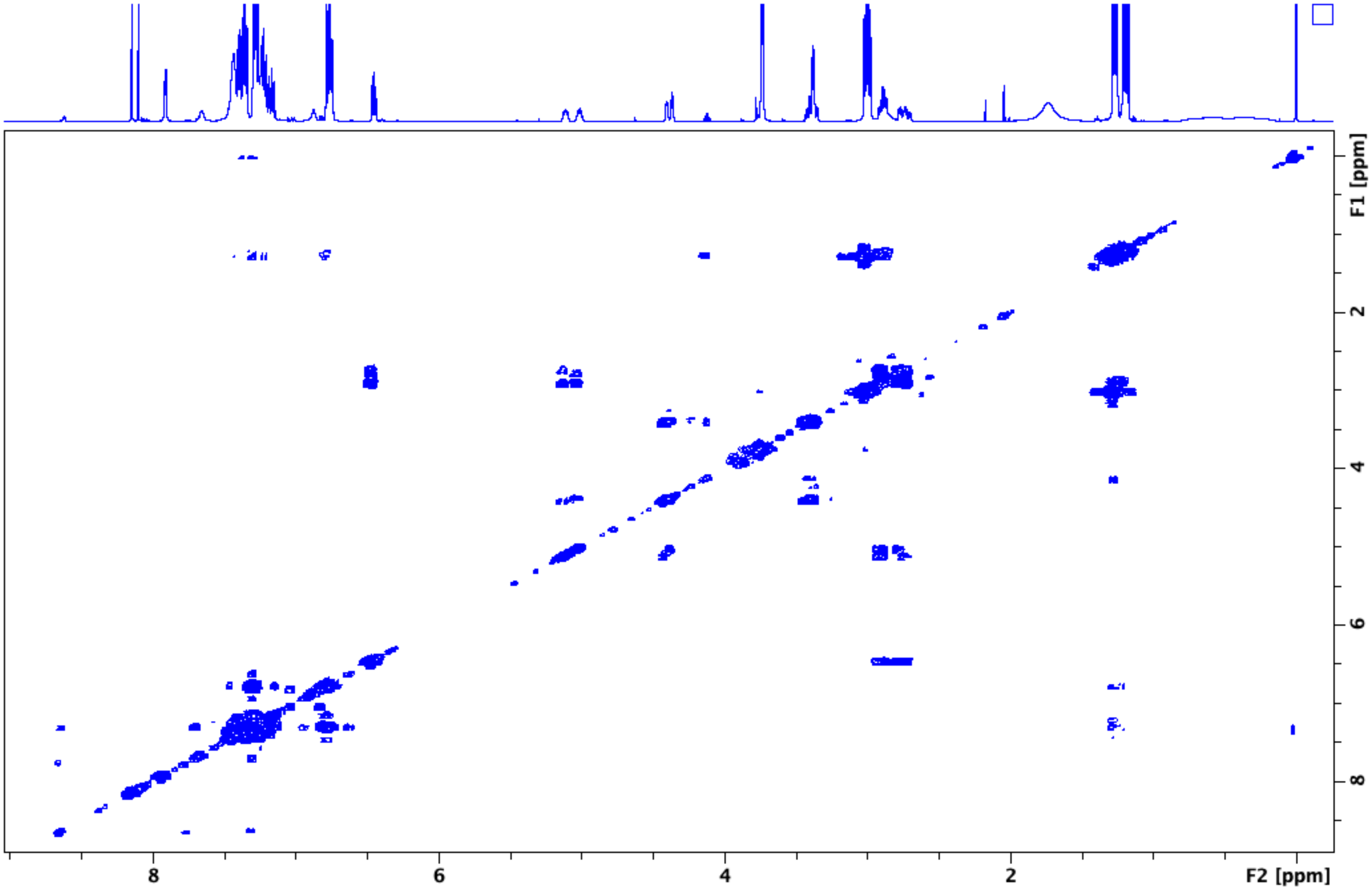

HSQC (CDCl3)

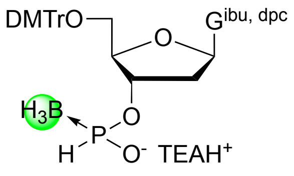

2g

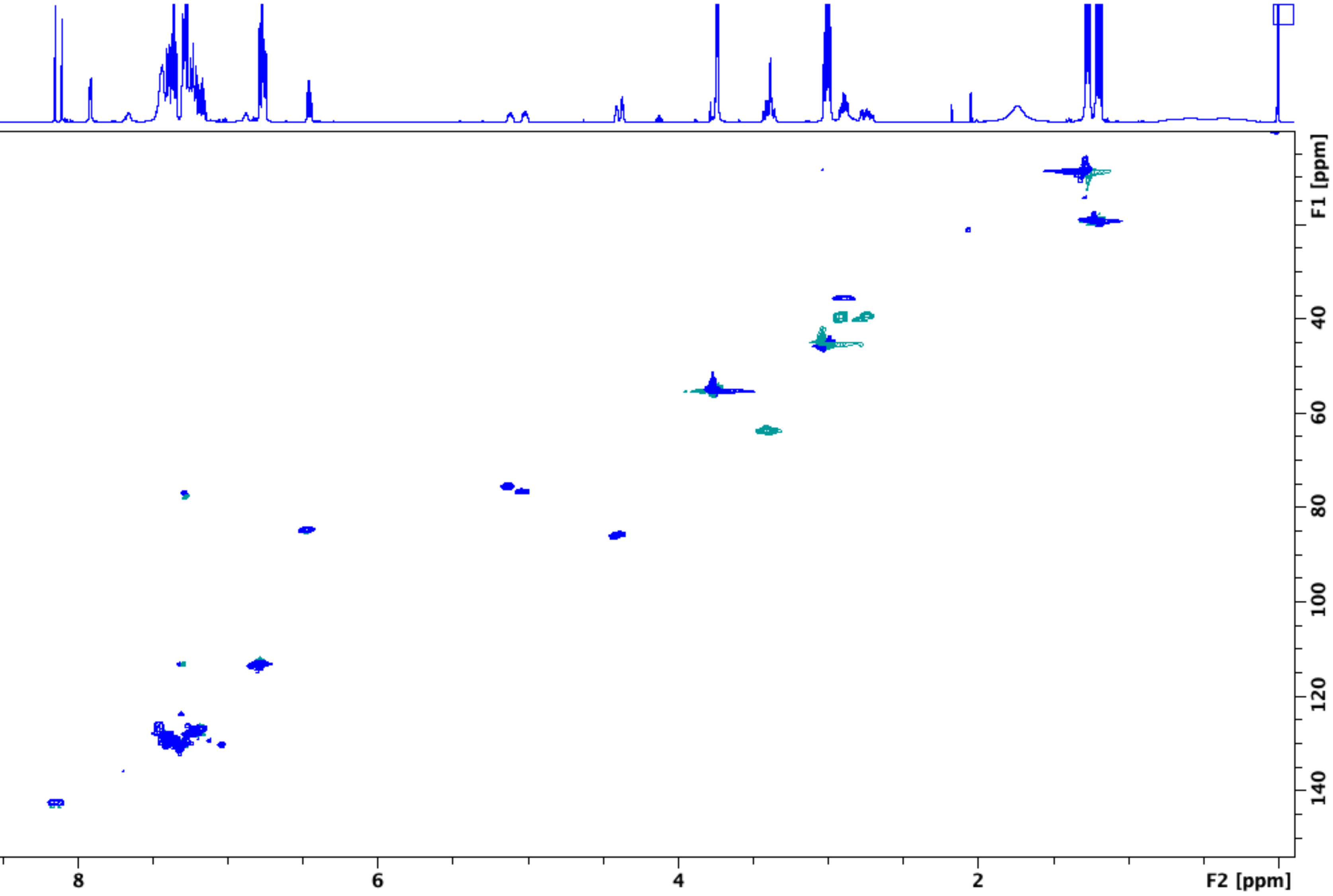

HMBC ($CDCl_3$)

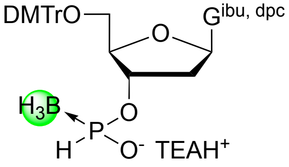

2g

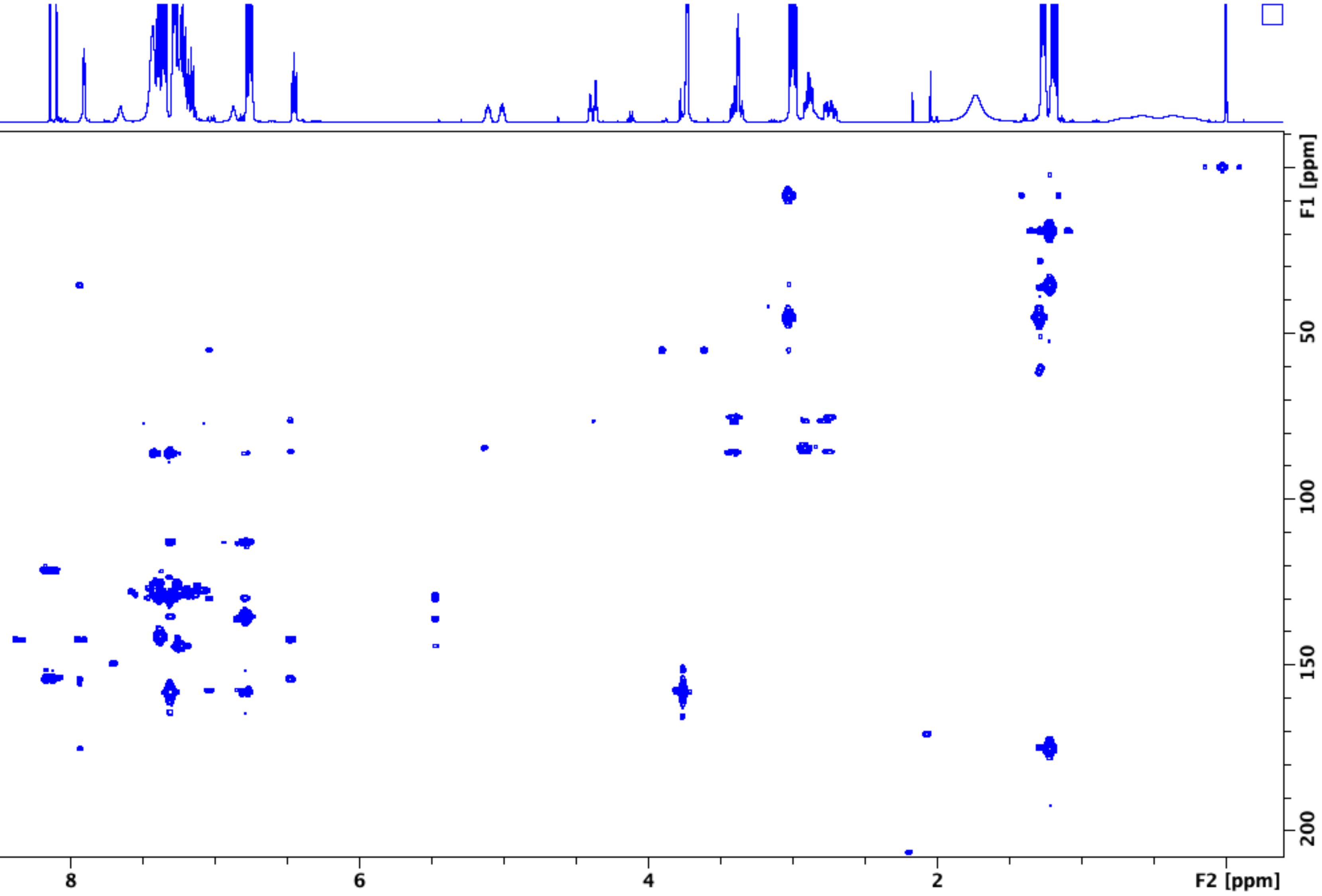

$^{31}P\{^1H\}$ NMR ($CDCl_3$, 202 MHz)

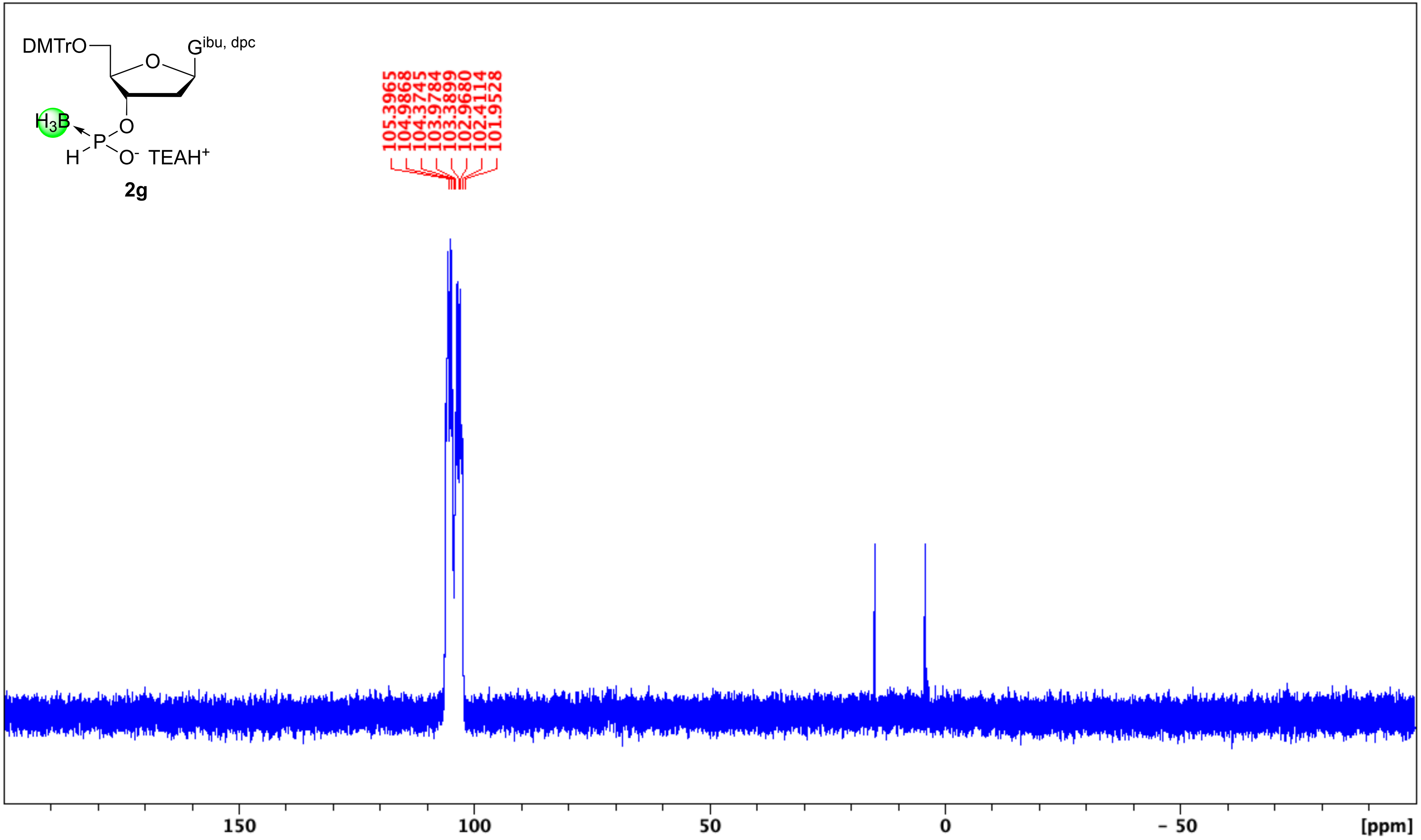

T-A 2-mer building block bearing *H*-boranophosphonate monoester on 3'-OH group (7ta)

^{1}H NMR ($CDCl_3$, 500 MHz)

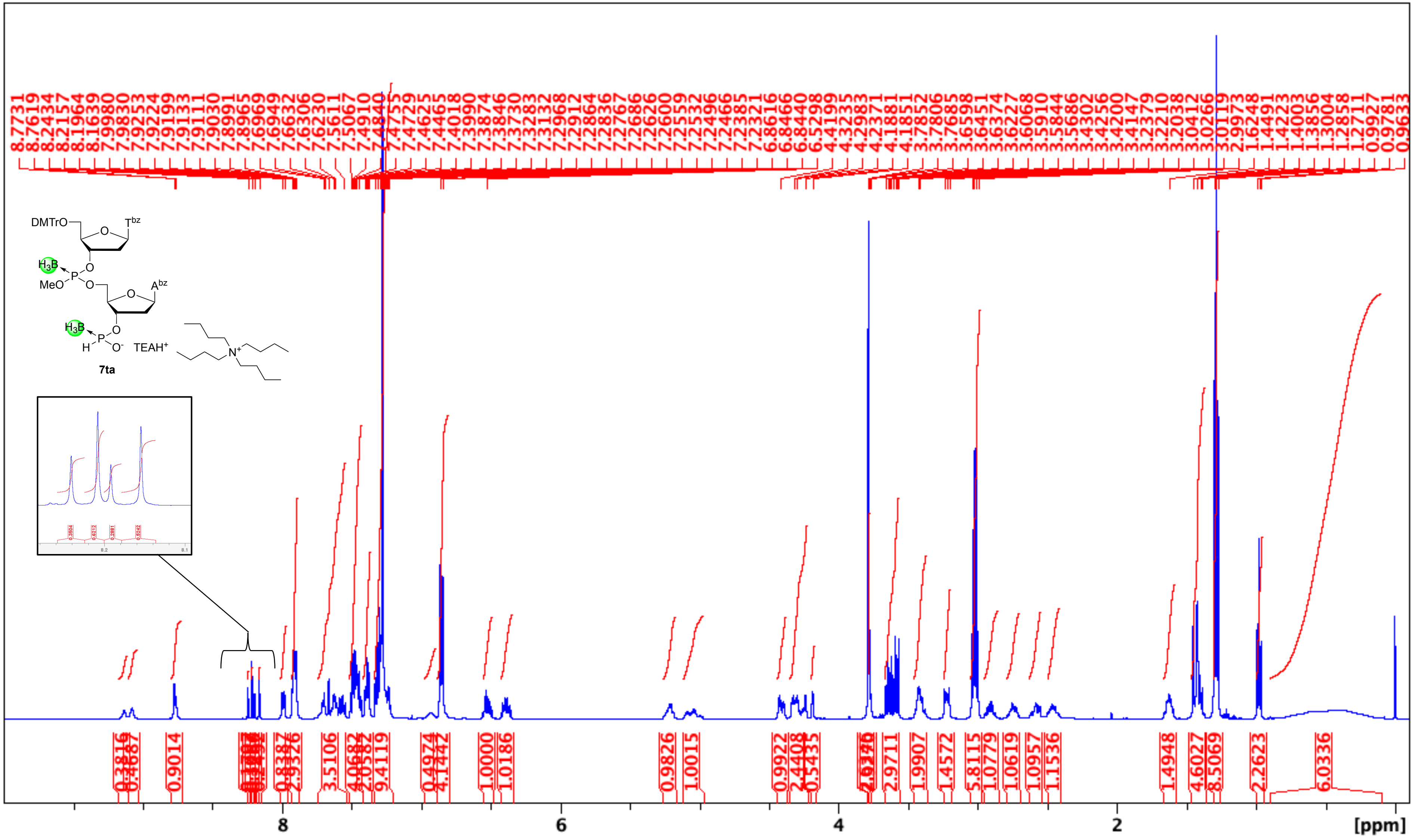

$^{13}C\{^{1}H\}$ NMR ($CDCl_3$, 126 MHz)

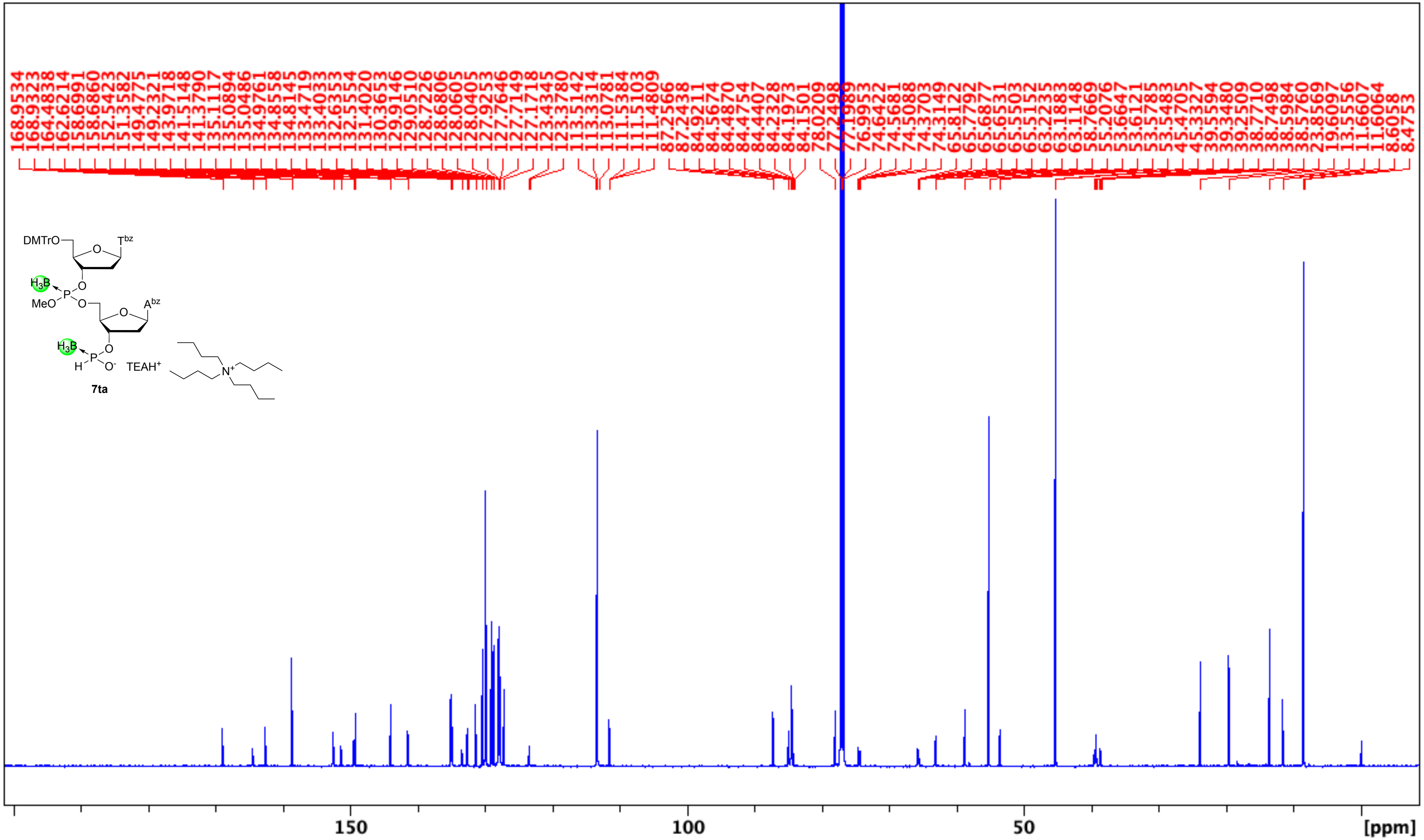

COSY ($CDCl_3$)

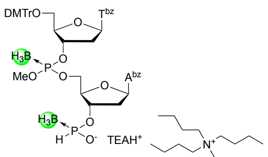

7ta

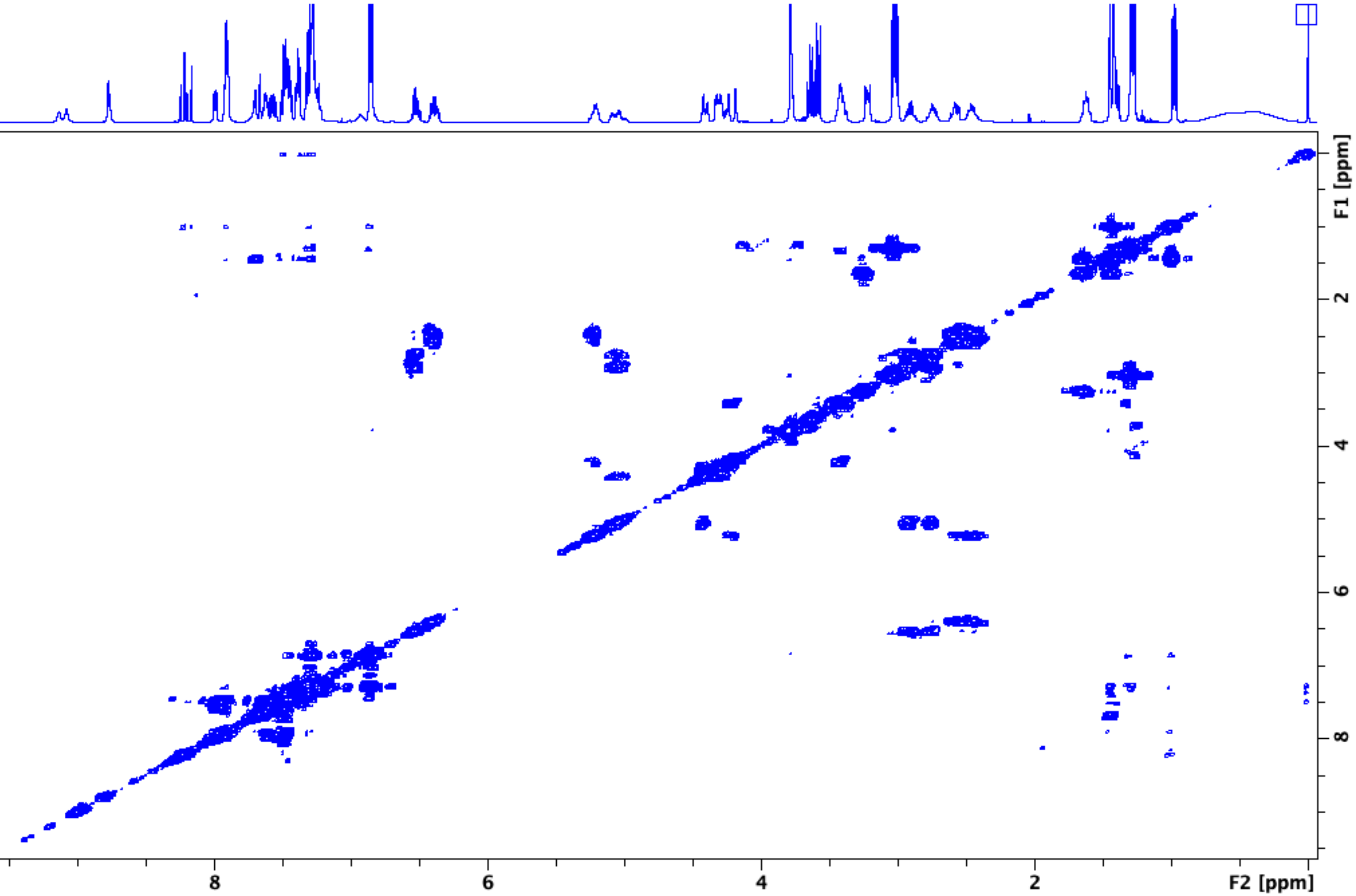

HSQC ($CDCl_3$)

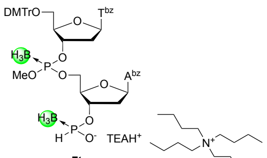

7ta

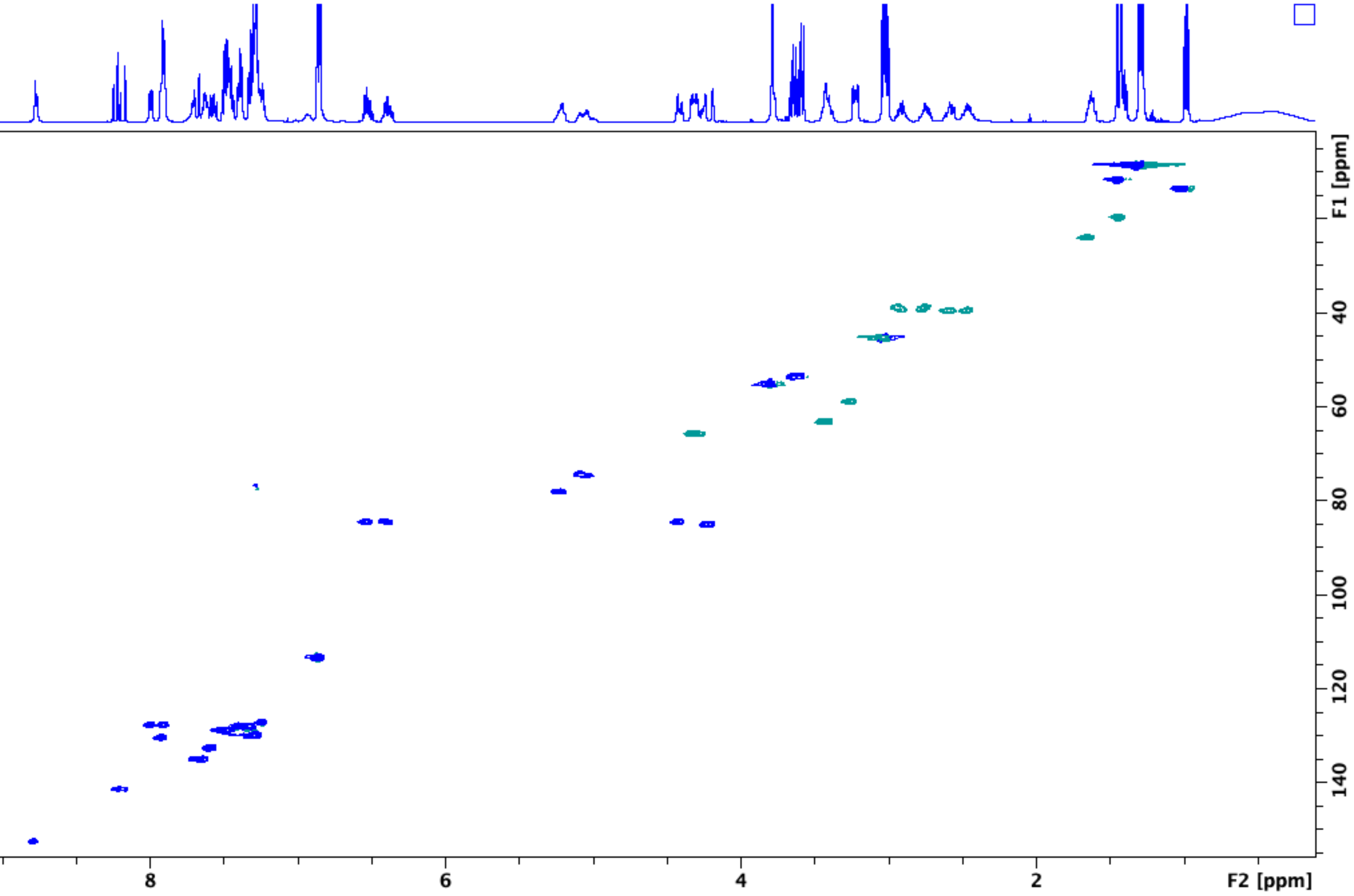

HMBC ($CDCl_3$)

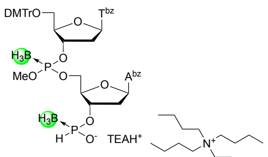

7ta

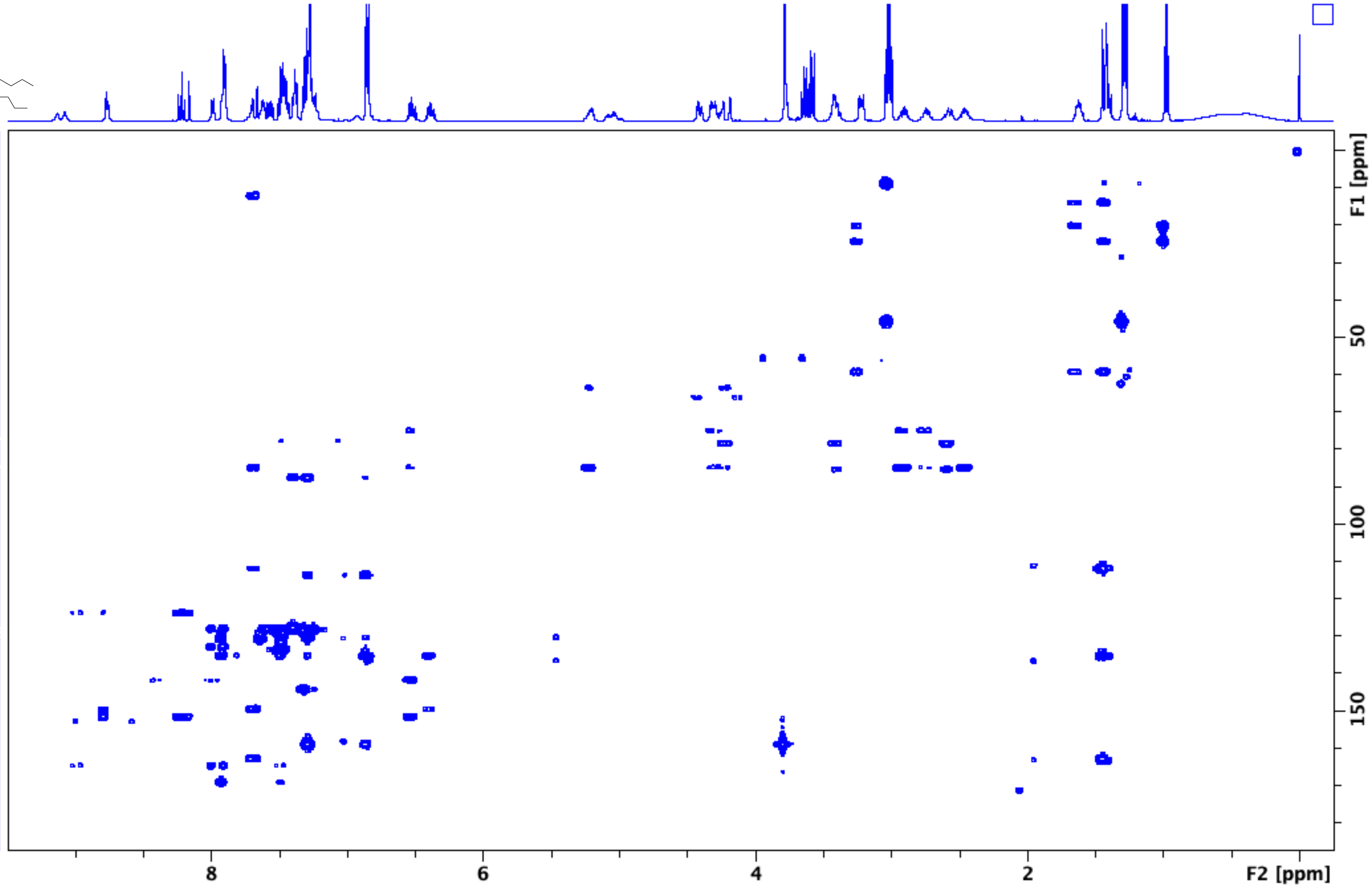

$^{31}P\{^{1}H\}$ NMR ($CDCl_3$, 202 MHz)

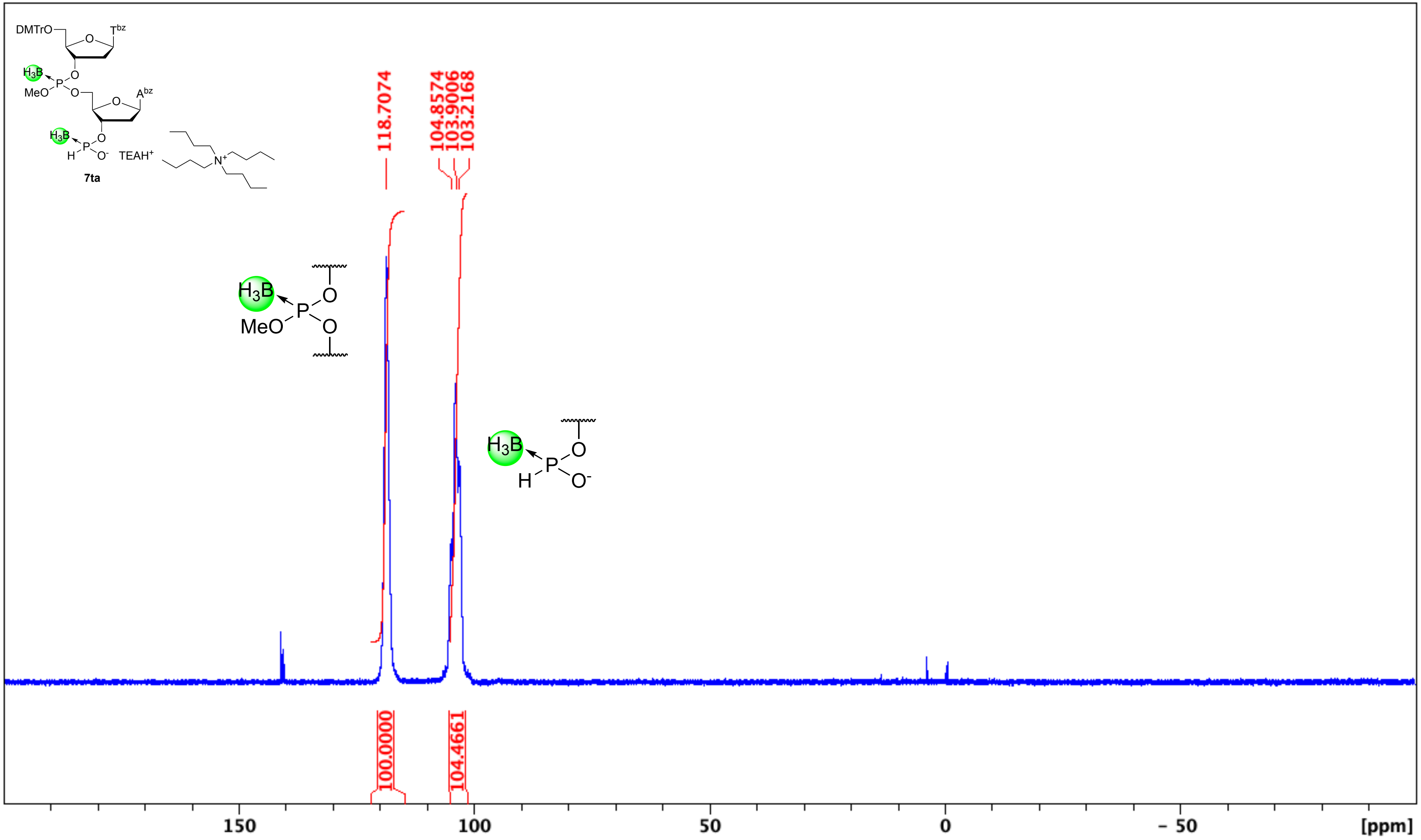

$^{11}B\ \{^{1}H\}$ NMR ($CDCl_3$, 160 MHz)

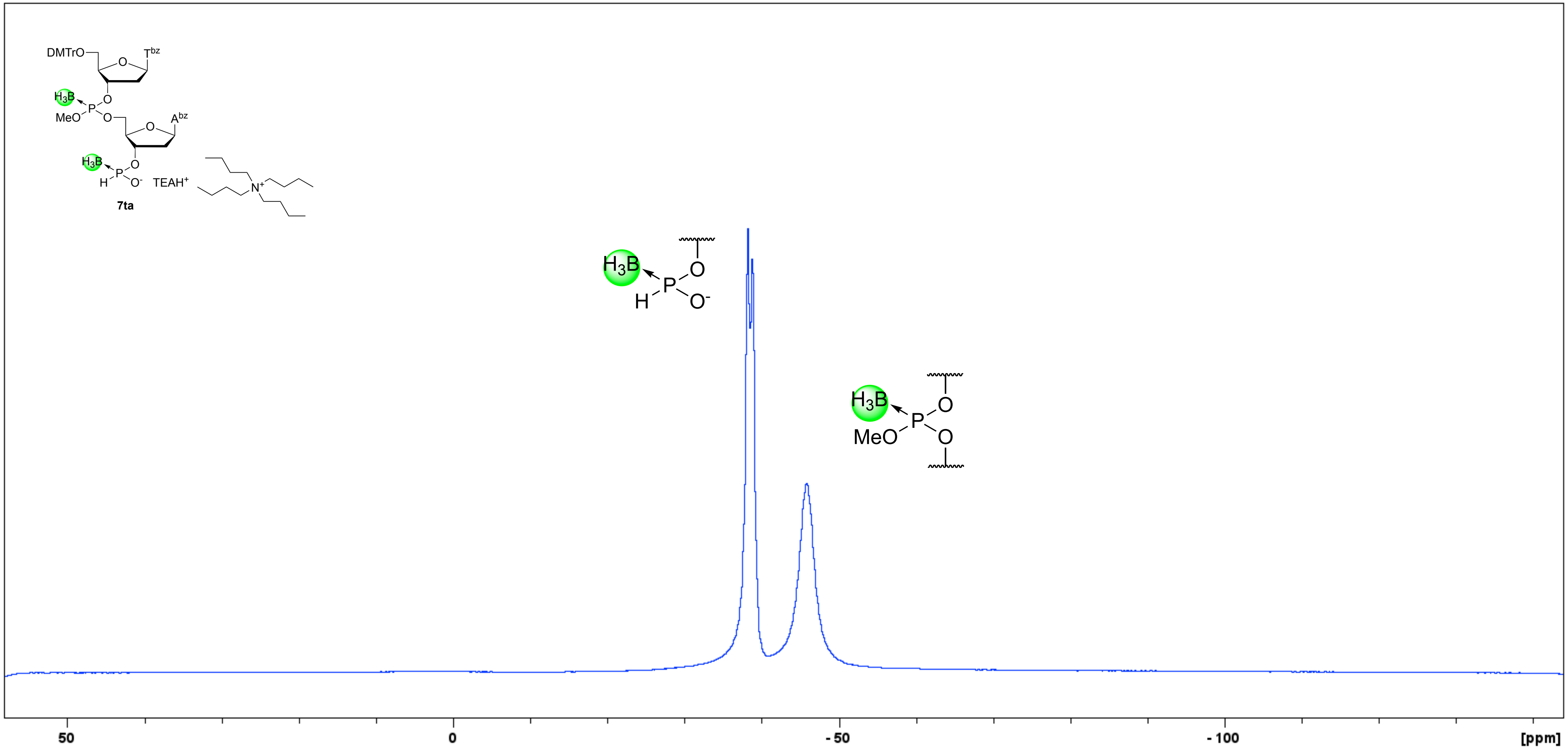

Potassium Fluorenylmethyl *H*-boranophosphonate monoester (10)

^{1}H NMR ($CDCl_3$, 500 MHz)

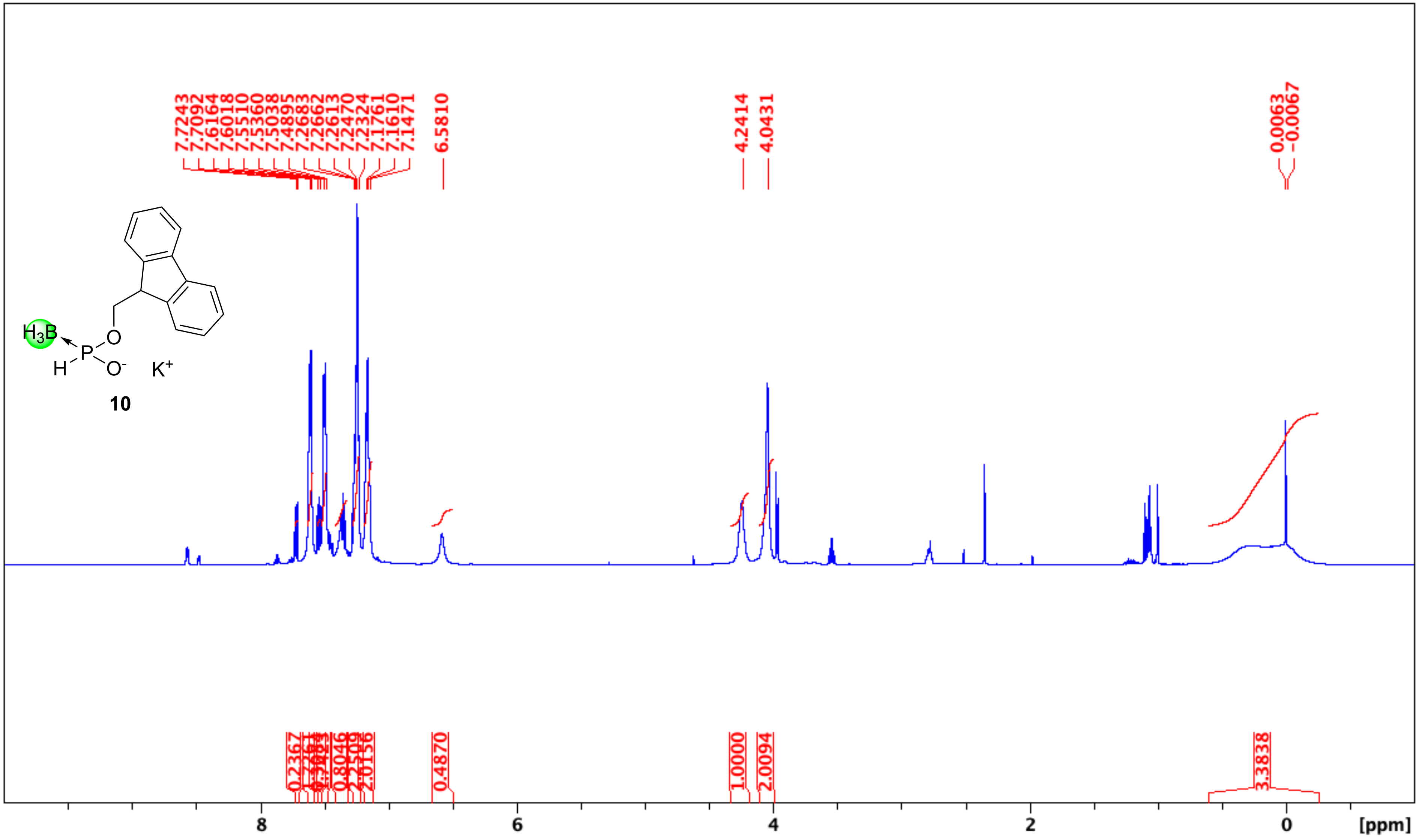

$^{13}C\{^{1}H\}$ NMR ($CDCl_3$, 126 MHz)

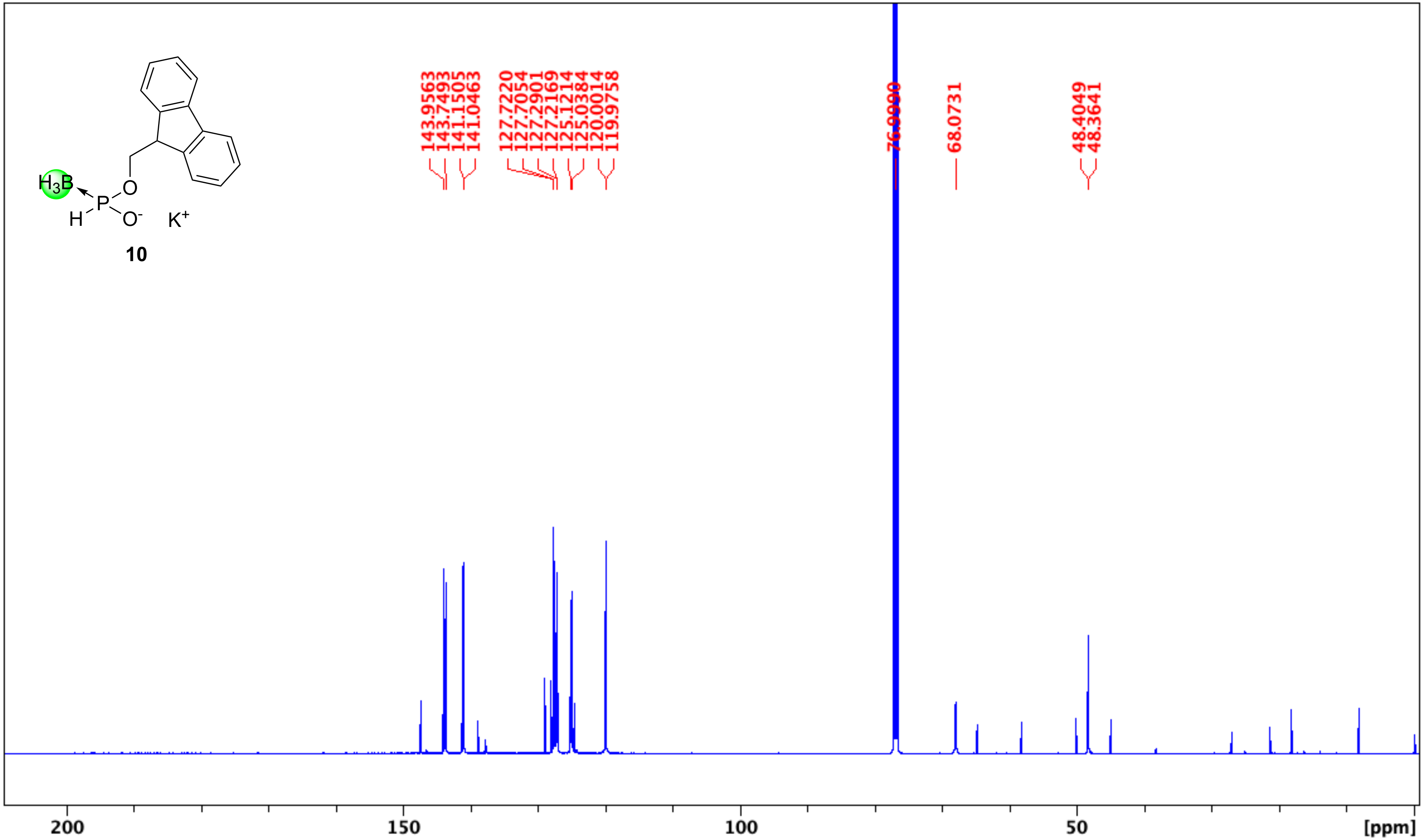

COSY ($CDCl_3$)

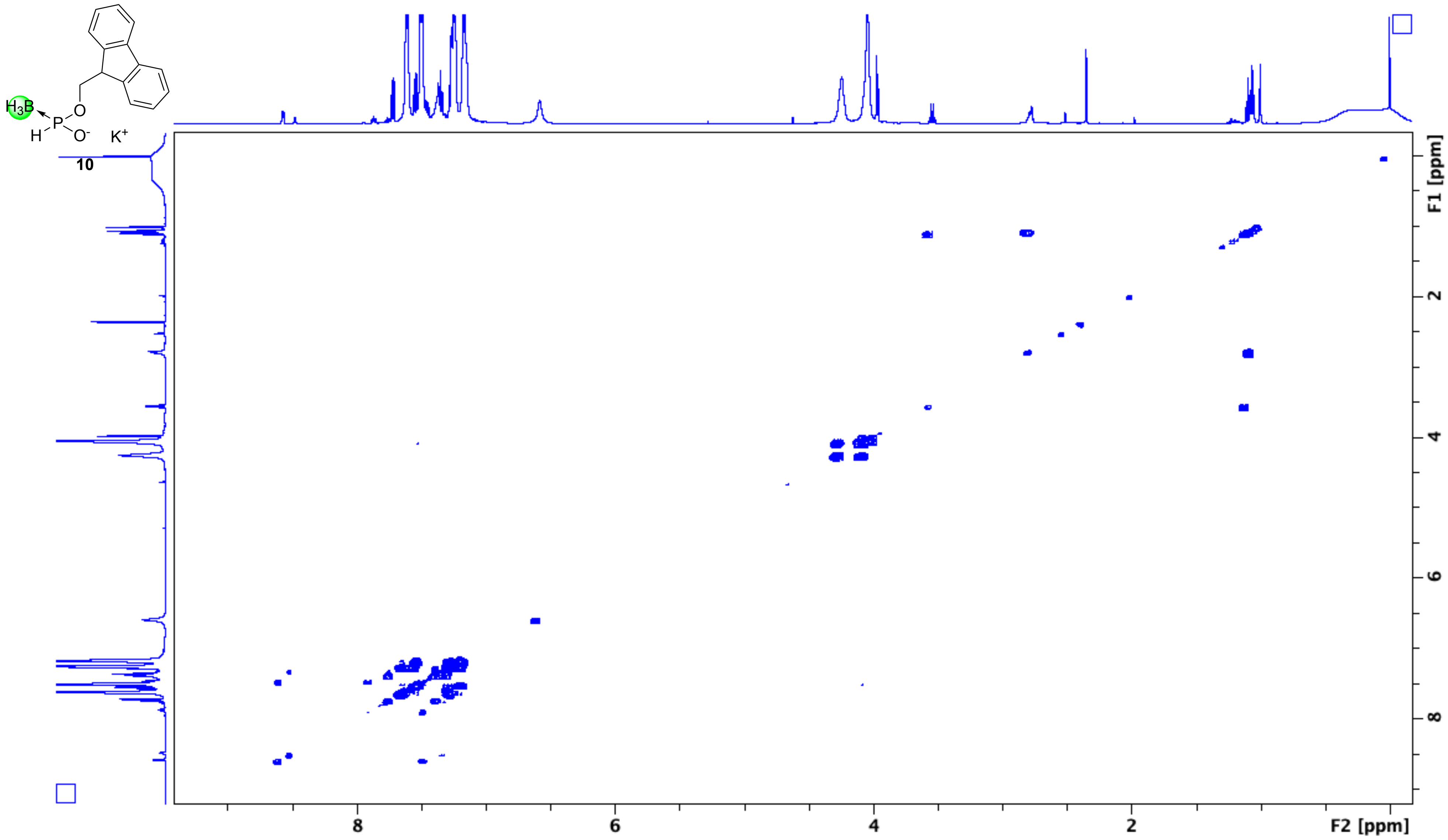

HSQC ($CDCl_3$)

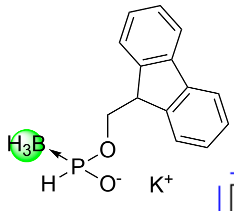

10

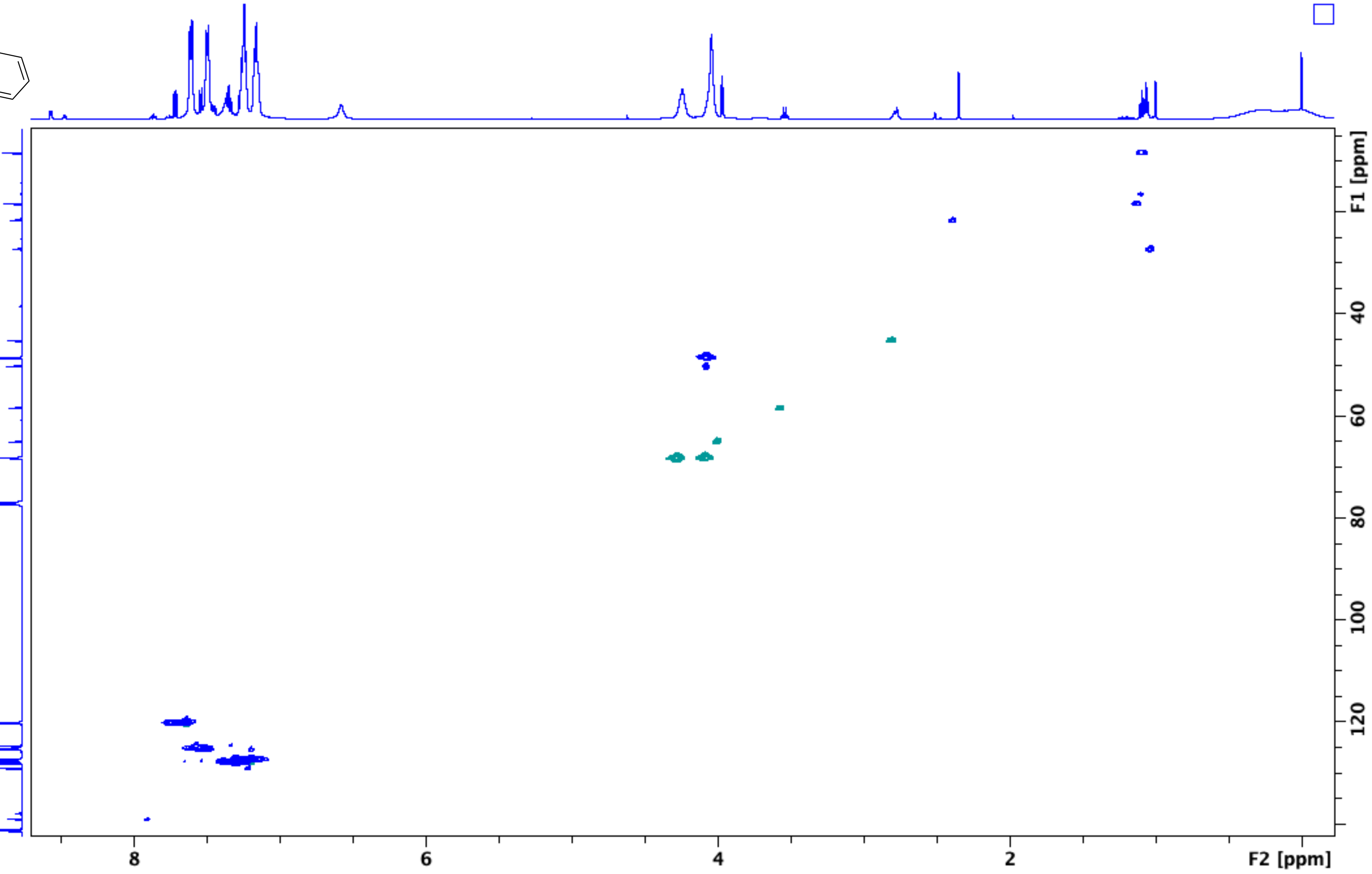

HMBC ($CDCl_3$)

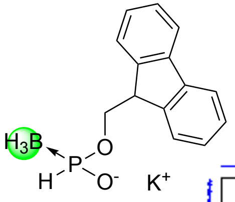

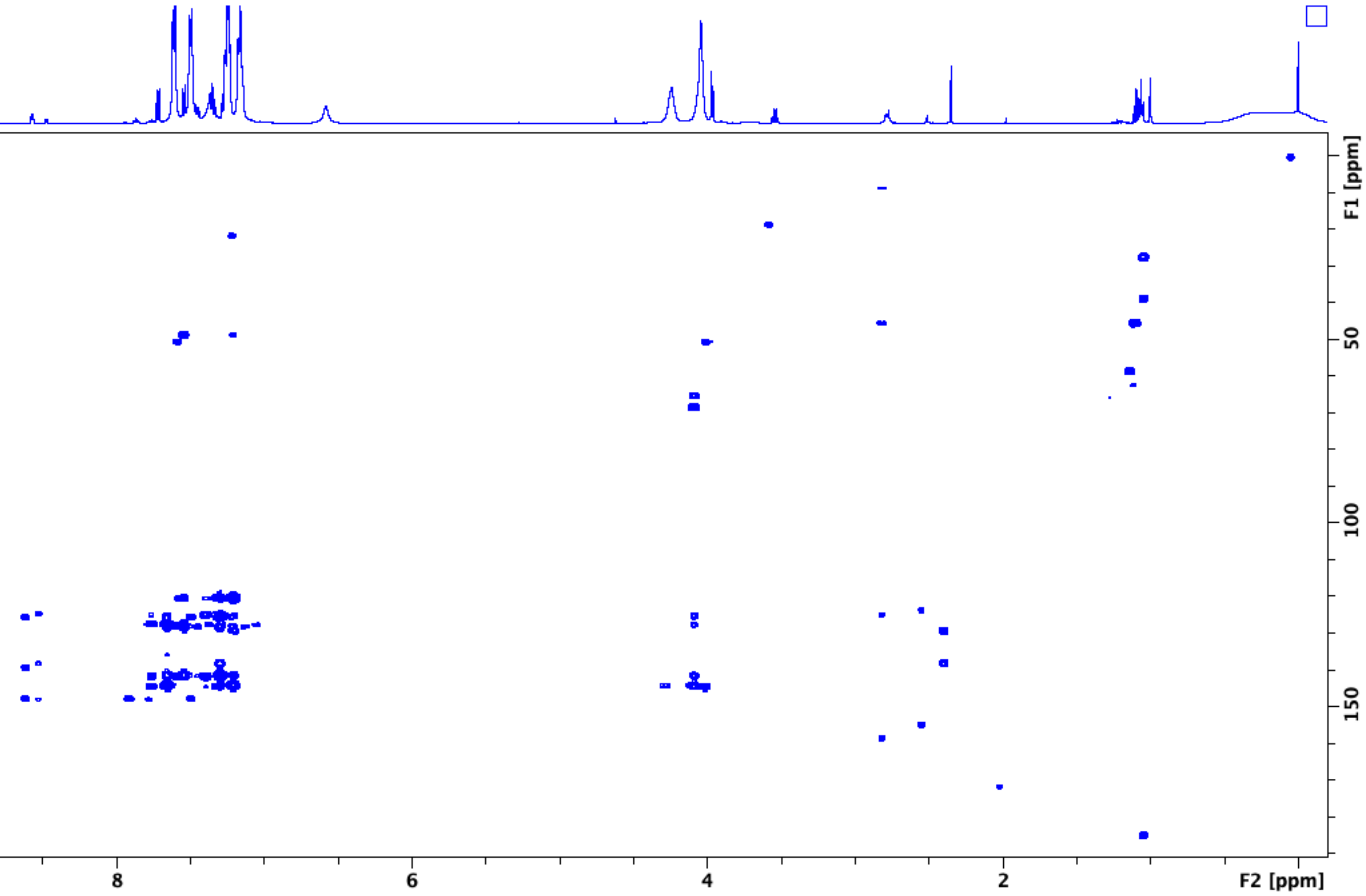

$^{31}P\{^1H\}$ NMR ($CDCl_3$, 202 MHz)

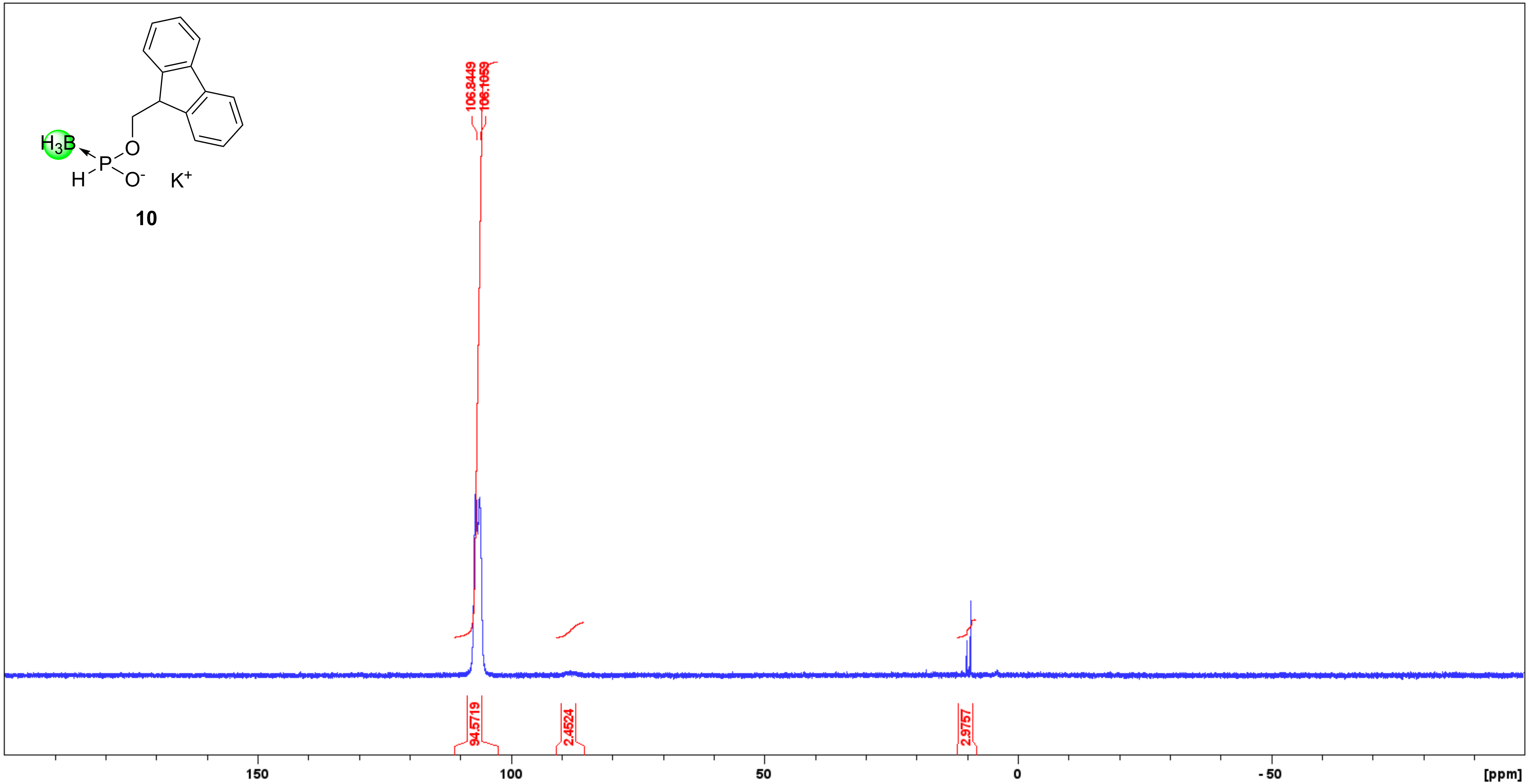

G-C 2-mer building block bearing 5'-OH group (12gc)

^{1}H NMR ($CDCl_3$, 500 MHz)

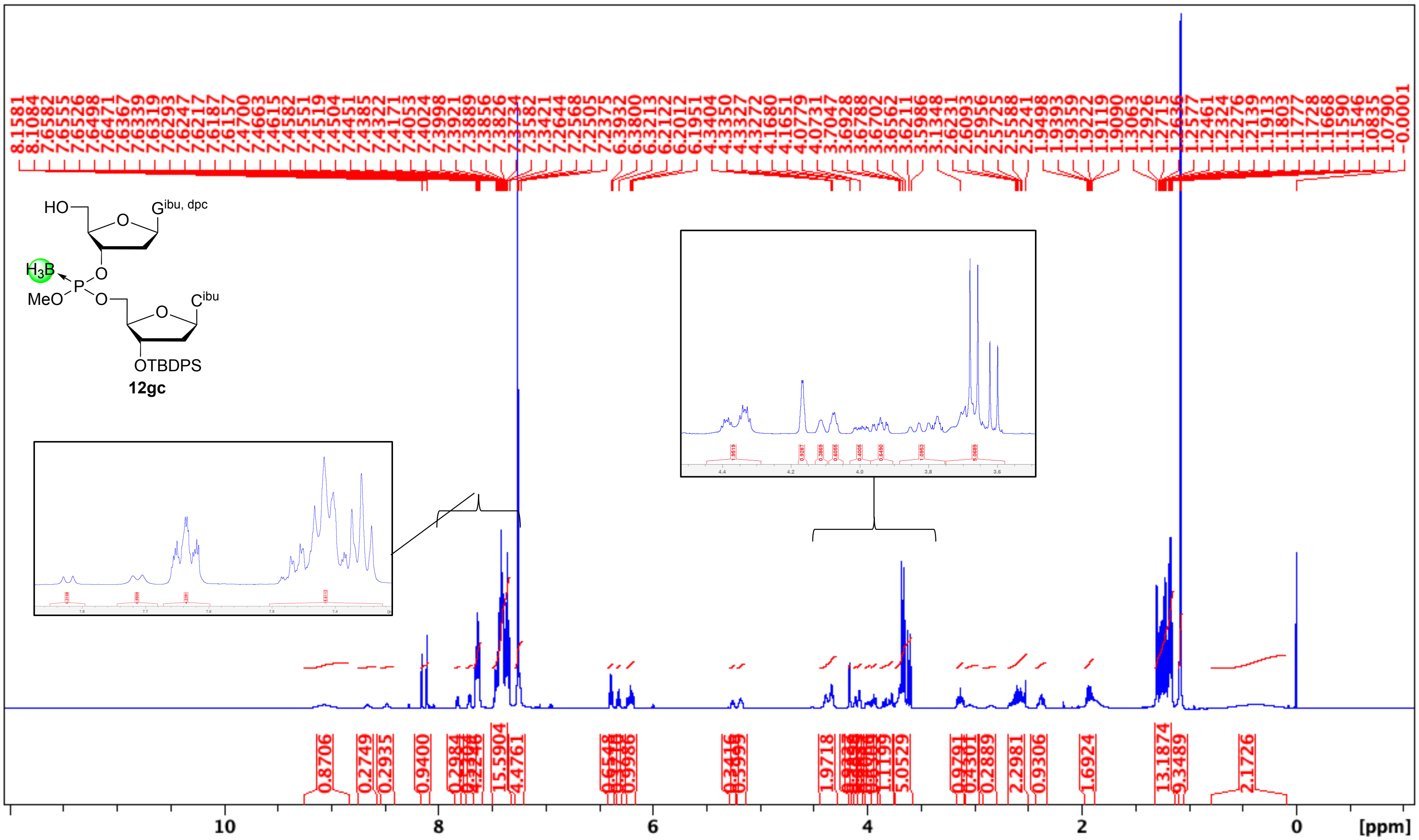

$^{13}C\{^{1}H\}$ NMR ($CDCl_3$, 126 MHz)

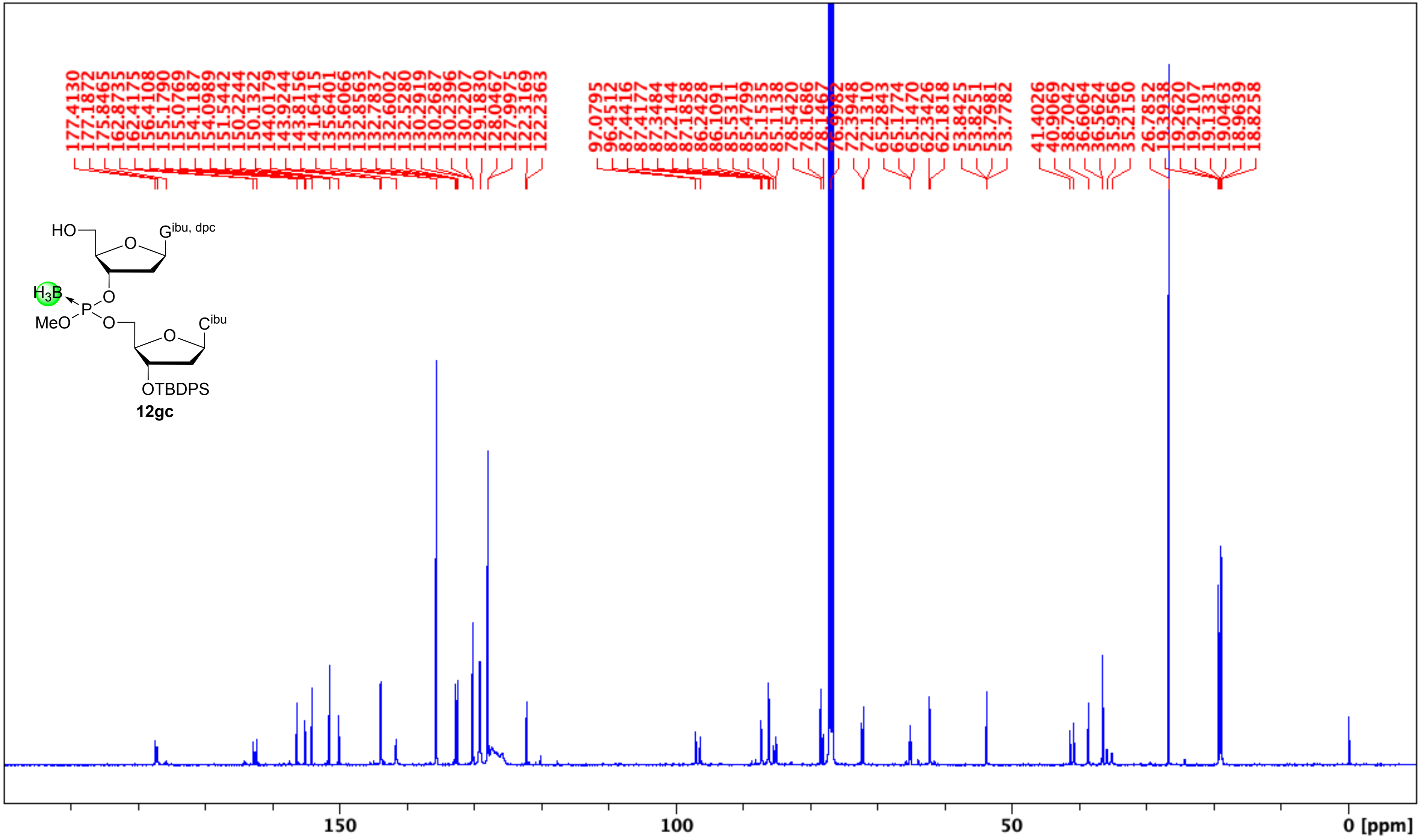

COSY ($CDCl_3$)

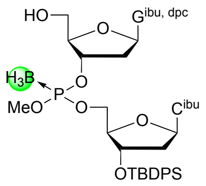

12gc

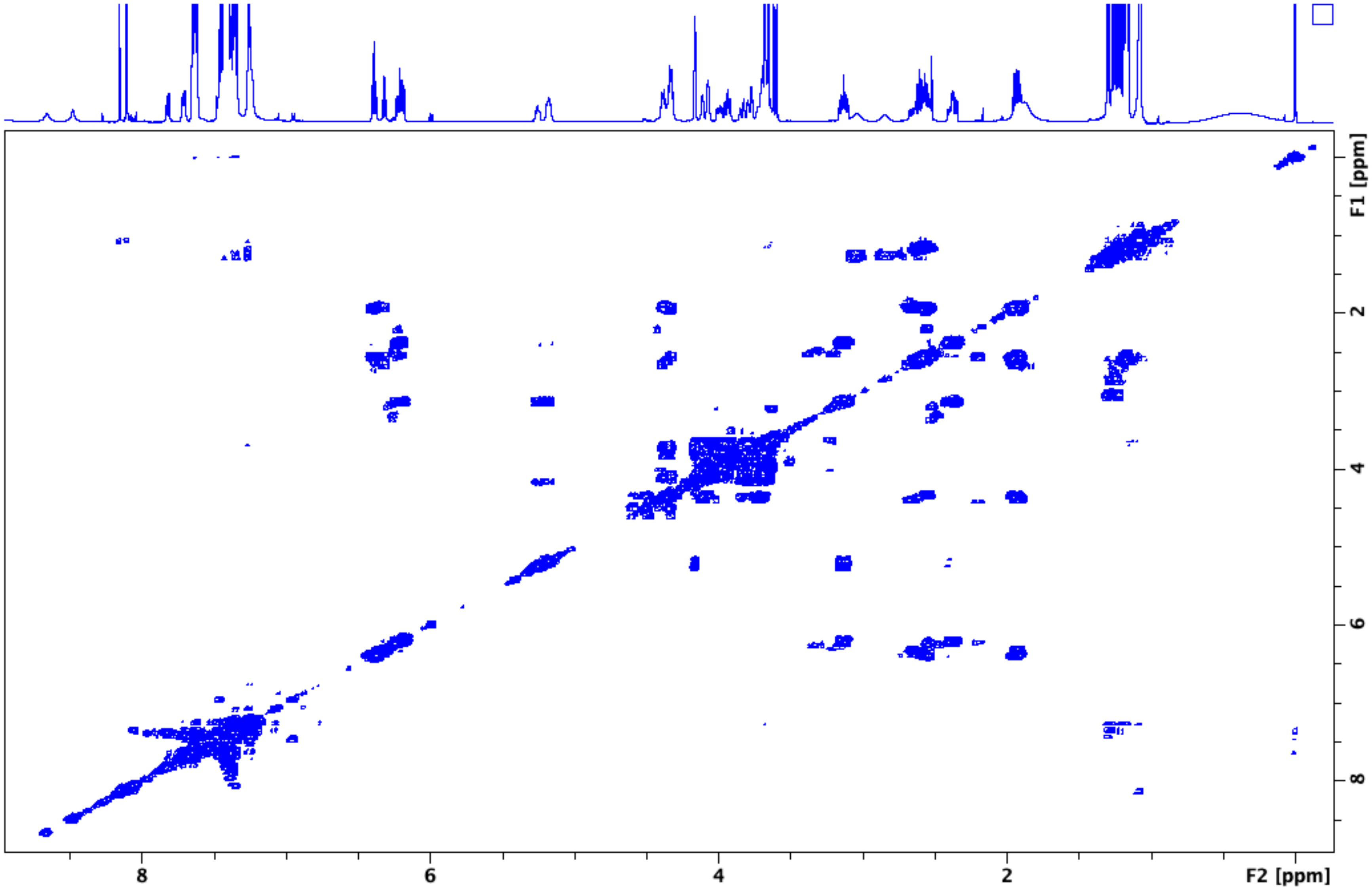

HSQC ($CDCl_3$)

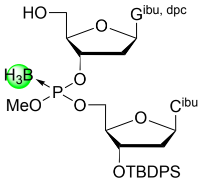

HO
O
G^ibu, dpc
H3B
O
P
MeO
O
O
C^ibu
OTBDPS
12gc

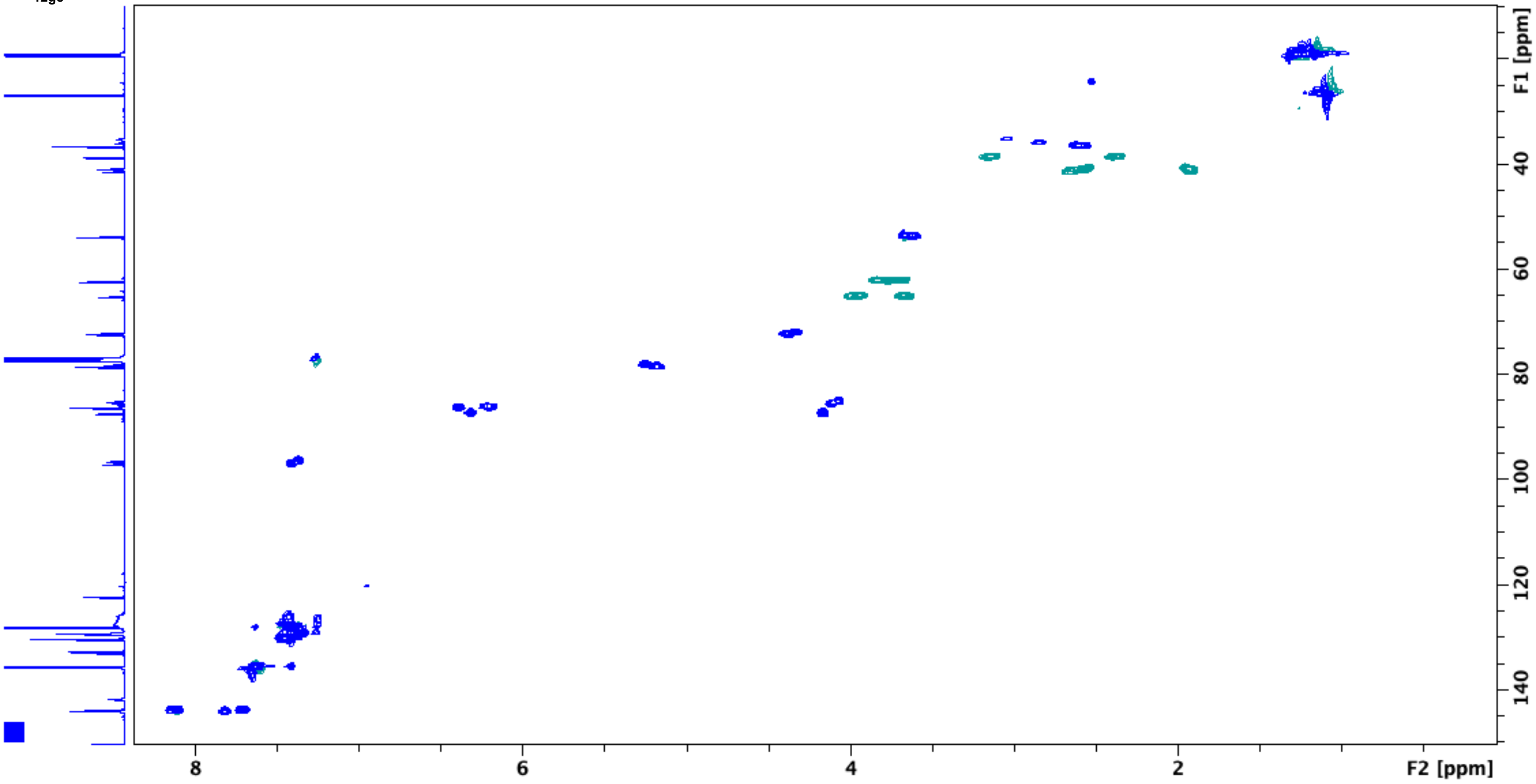

F1 [ppm]
40
60
80
100
120
140
8
6
4
2
F2 [ppm]

HMBC (CDCl3)

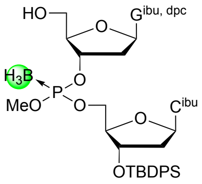

12gc

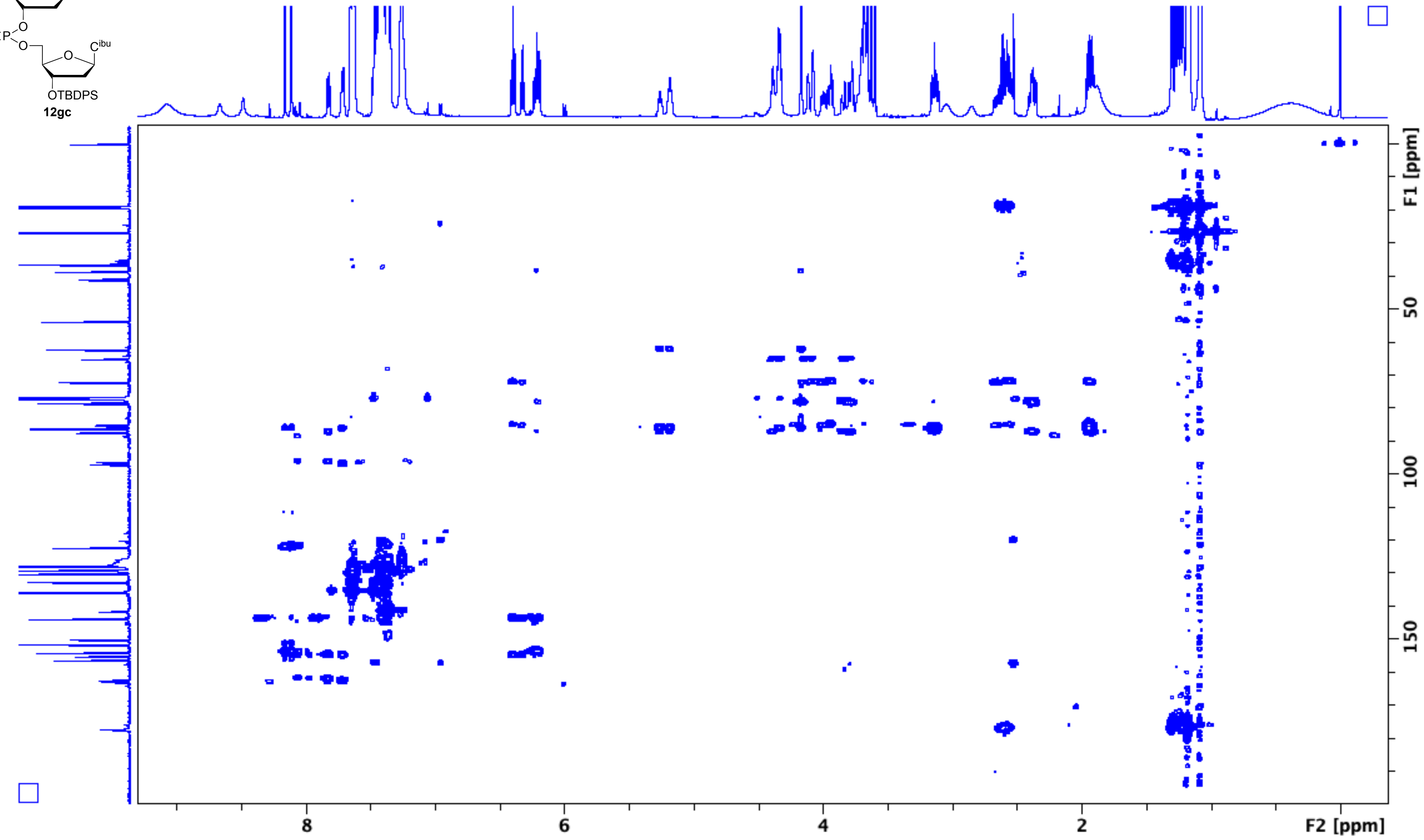

$^{31}P\{^1H\}$ NMR ($CDCl_3$, 202 MHz)

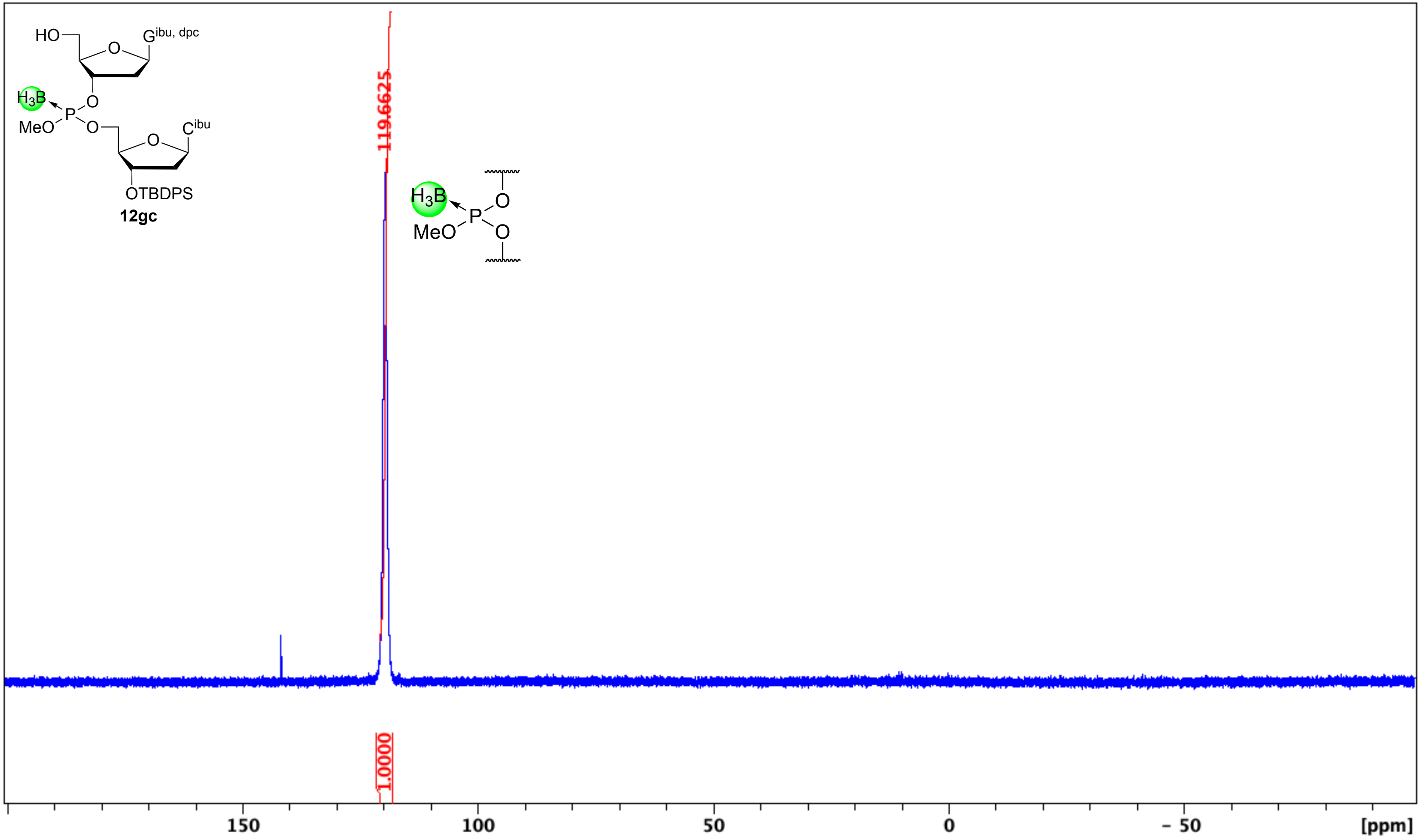

$^{11}B\ \{^{1}H\}$ NMR ($CDCl_3$, 160 MHz)

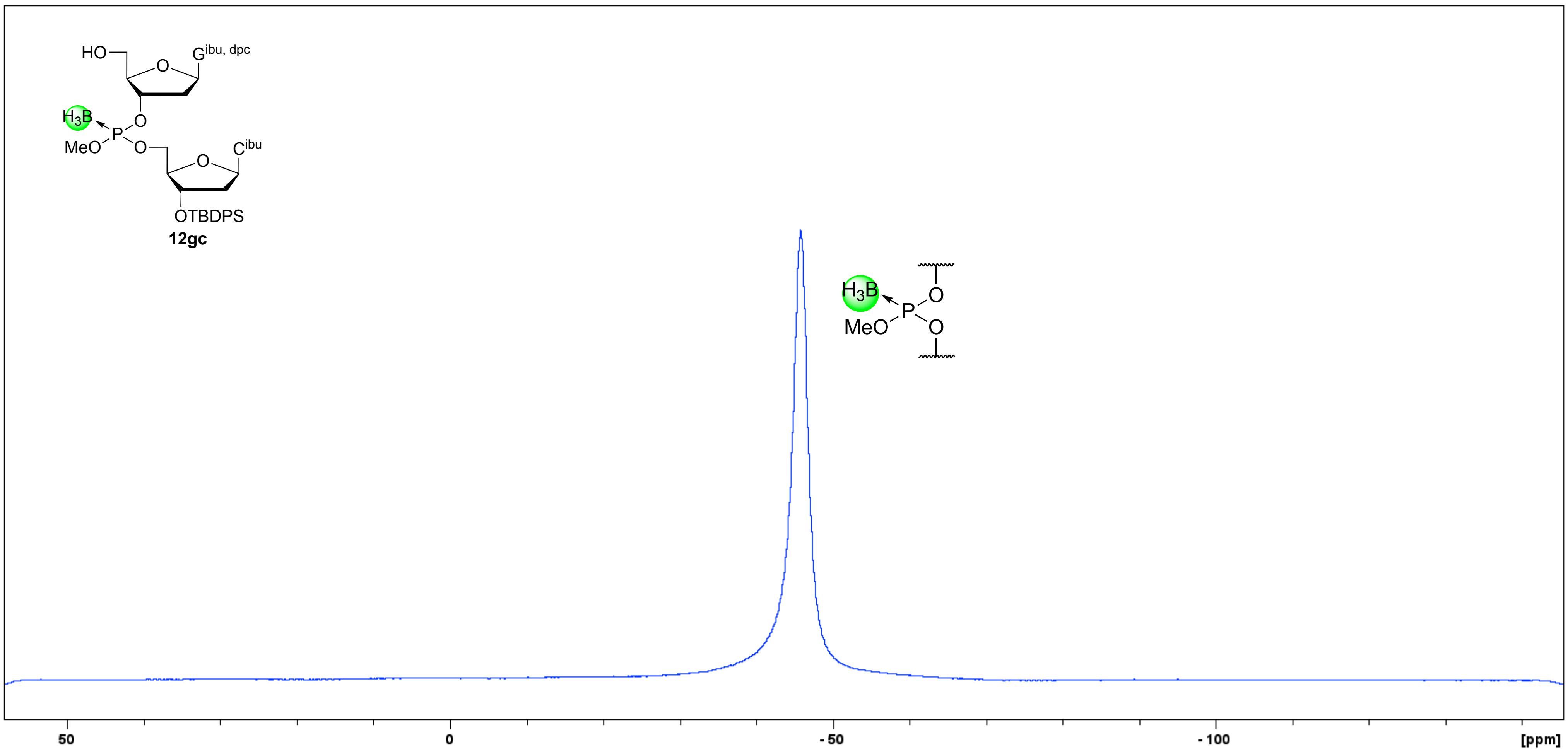

T-A-G-C 4-mer building block bearing 5′-OH group (14tagc)

^{1}H NMR ($CDCl_3$, 500 MHz)

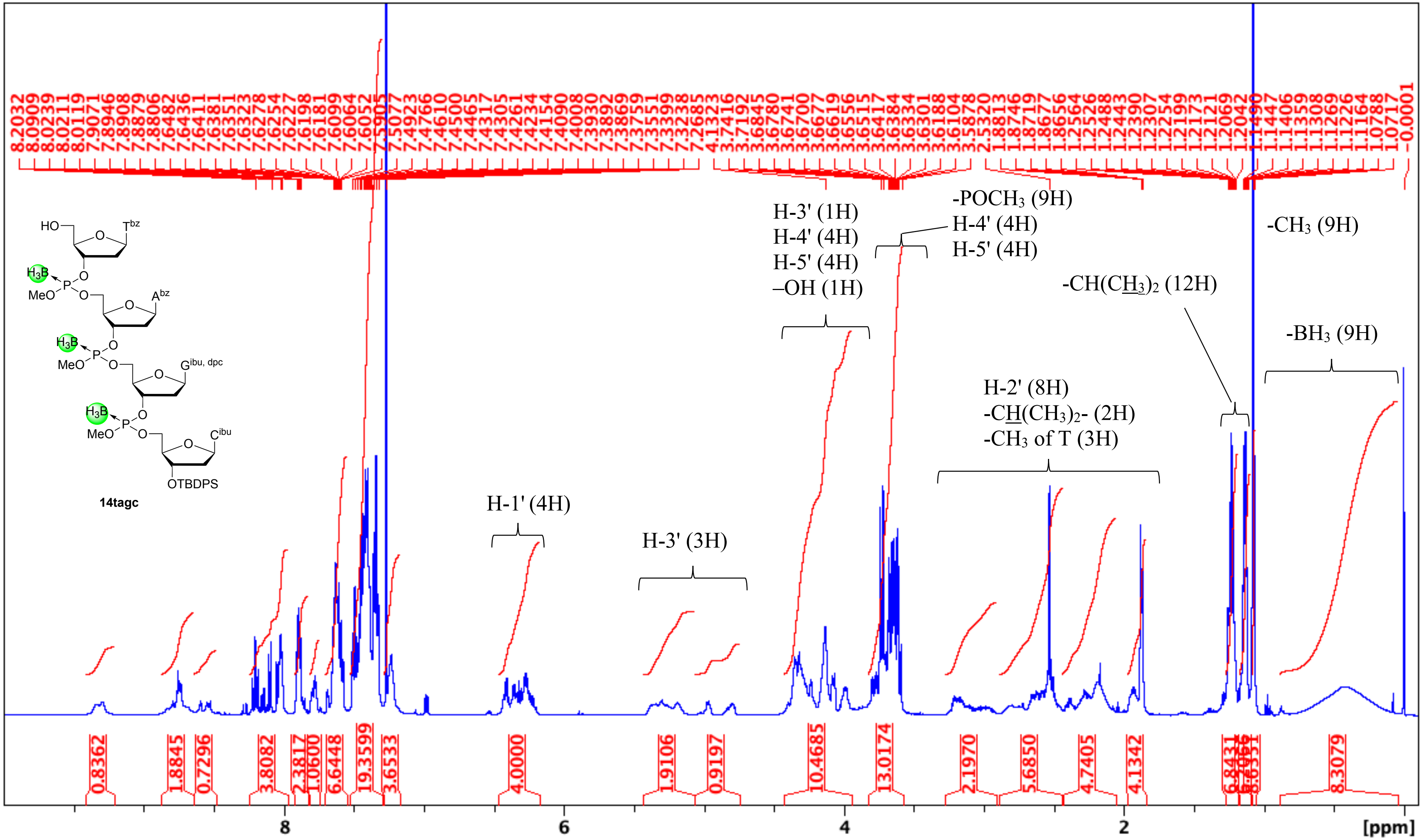

$^{13}C\{^{1}H\}$ NMR ($CDCl_3$, 126 MHz)

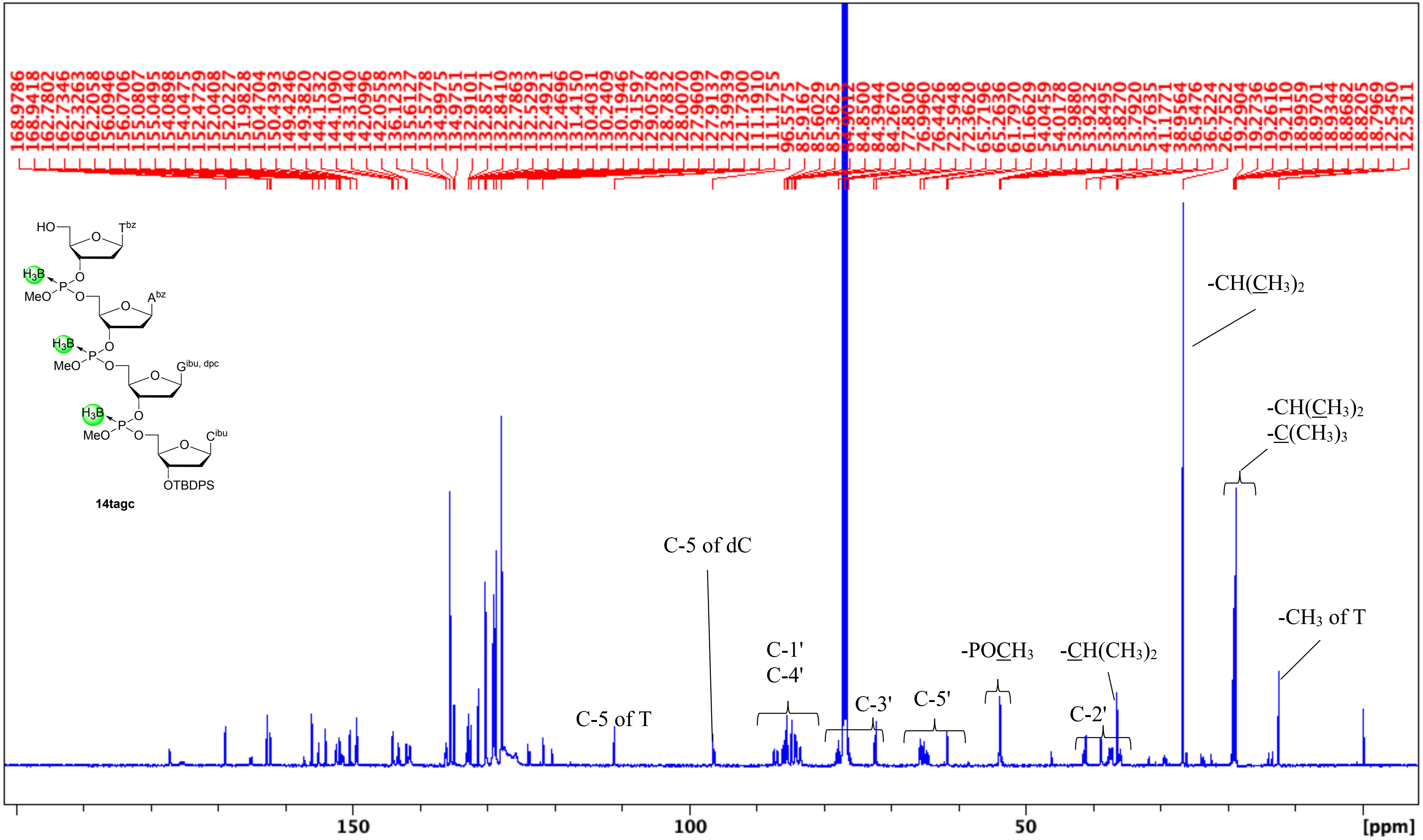

COSY ($CDCl_3$)

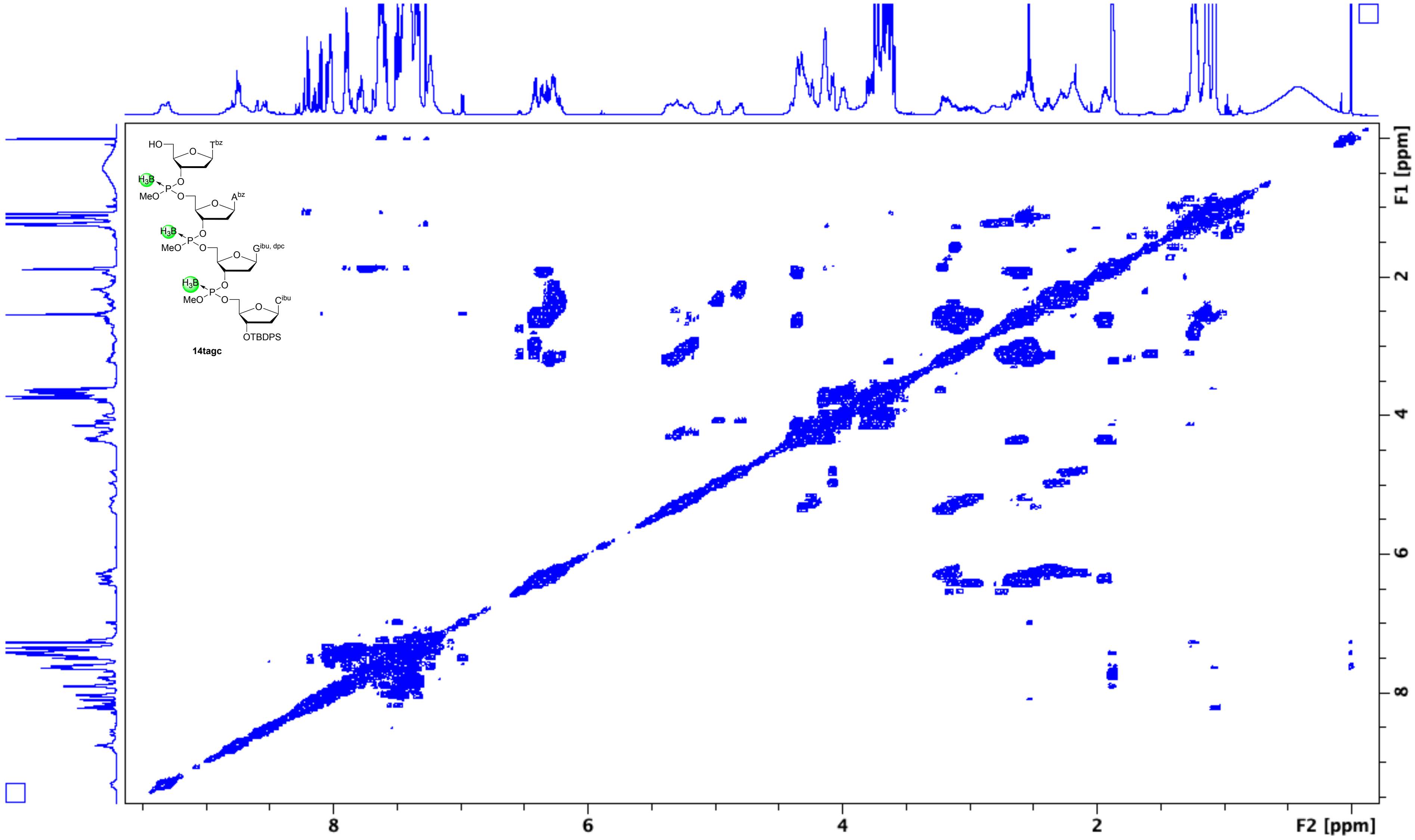

HO
O
Tbz
H3B
P
MeO
O
Abz
Gibu, dpc
Cibu
OTBDPS
14tagc
F1 [ppm]
F2 [ppm]
8
6
4
2

HSQC ($CDCl_3$)

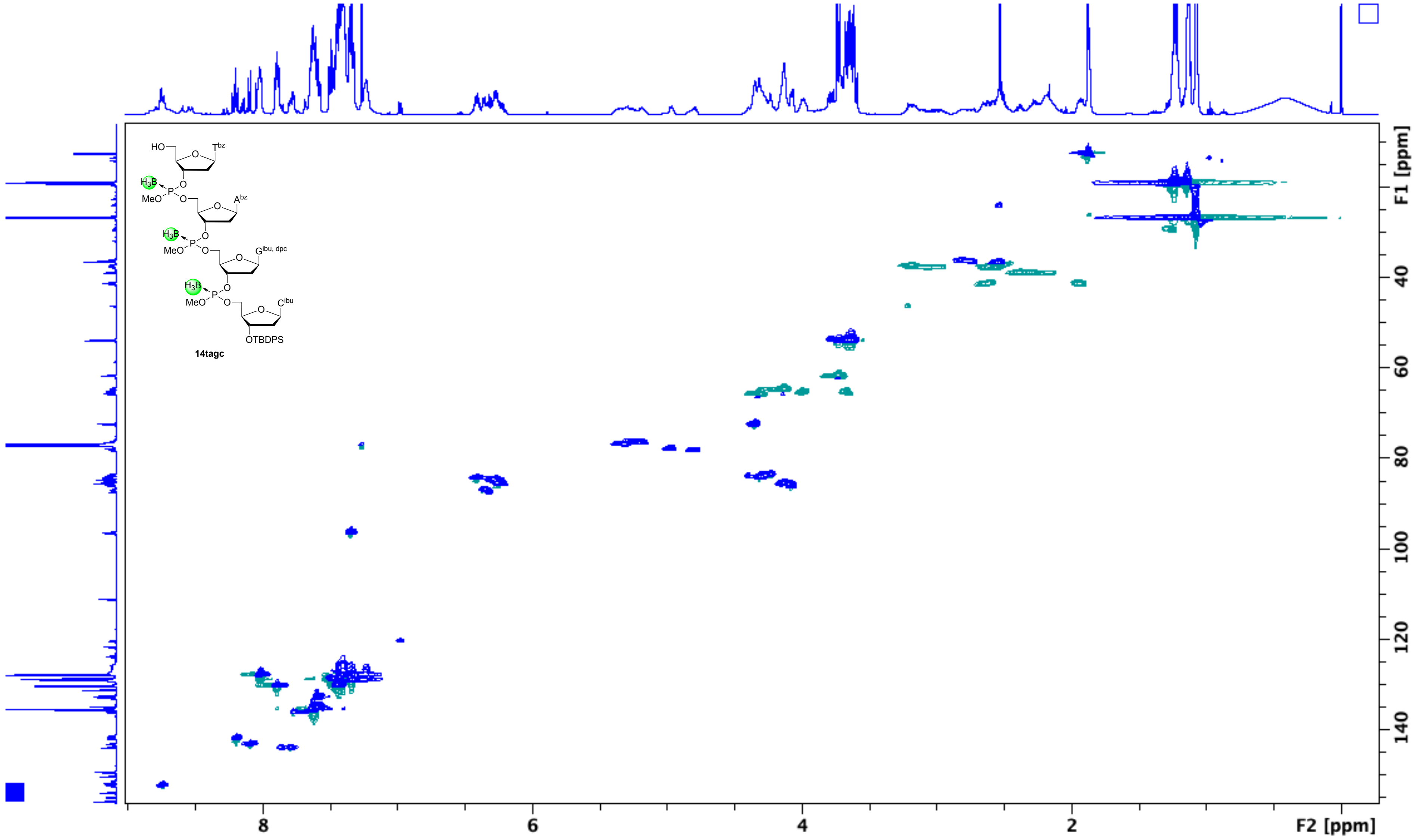

HO
T^bz
H3B
MeO
O
A^bz
G^ibu, dpc
C^ibu
OTBDPS
14tagc
F1 [ppm]
F2 [ppm]
8
6
4
2
40
60
80
100
120
140

HMBC ($CDCl_3$)

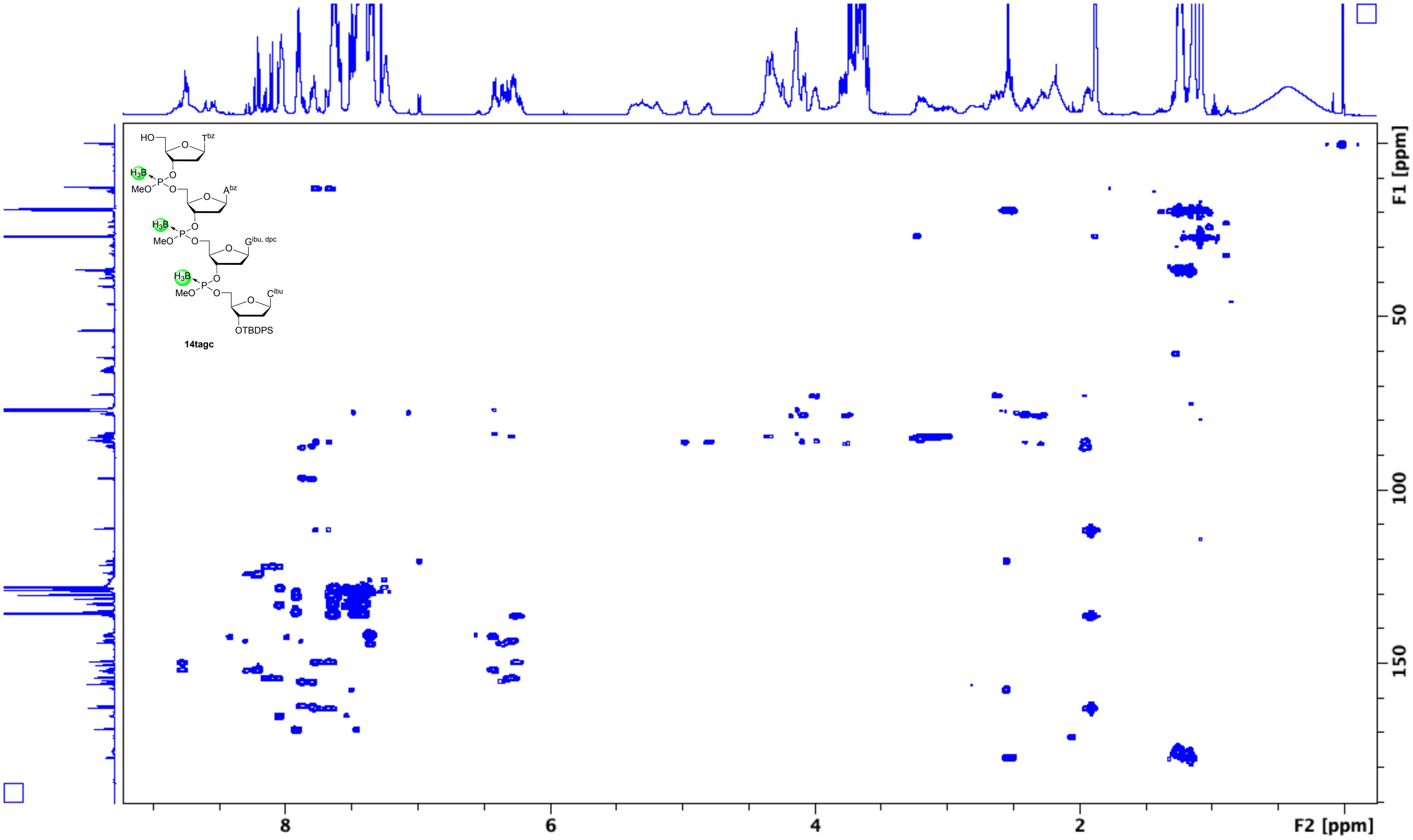

HO
O
T^bz
H3B
P
O
MeO
O
A^bz
H3B
P
O
MeO
O
G^ibu, dpc
H3B
P
O
MeO
O
C^ibu
OTBDPS
14tagc
F1 [ppm]
50
100
150
8
6
4
2
F2 [ppm]

$^{31}P\{^{1}H\}$ NMR ($CDCl_3$, 202 MHz)

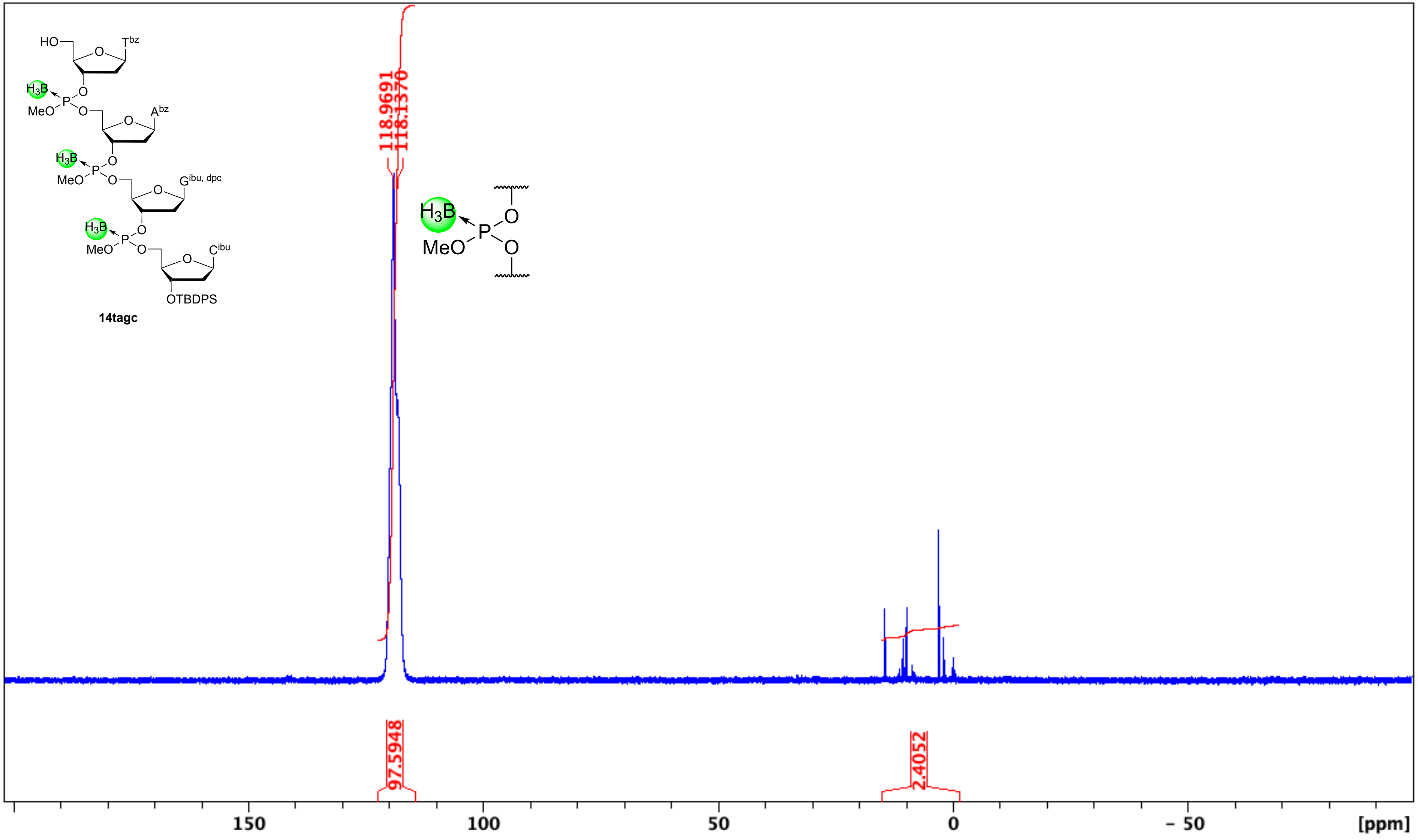

T-A-G-C 4-mer building block bearing *H*-boranophosphonate monoester on 3'-OH (15tagc)

^{1}H NMR ($CDCl_3$, 500 MHz)

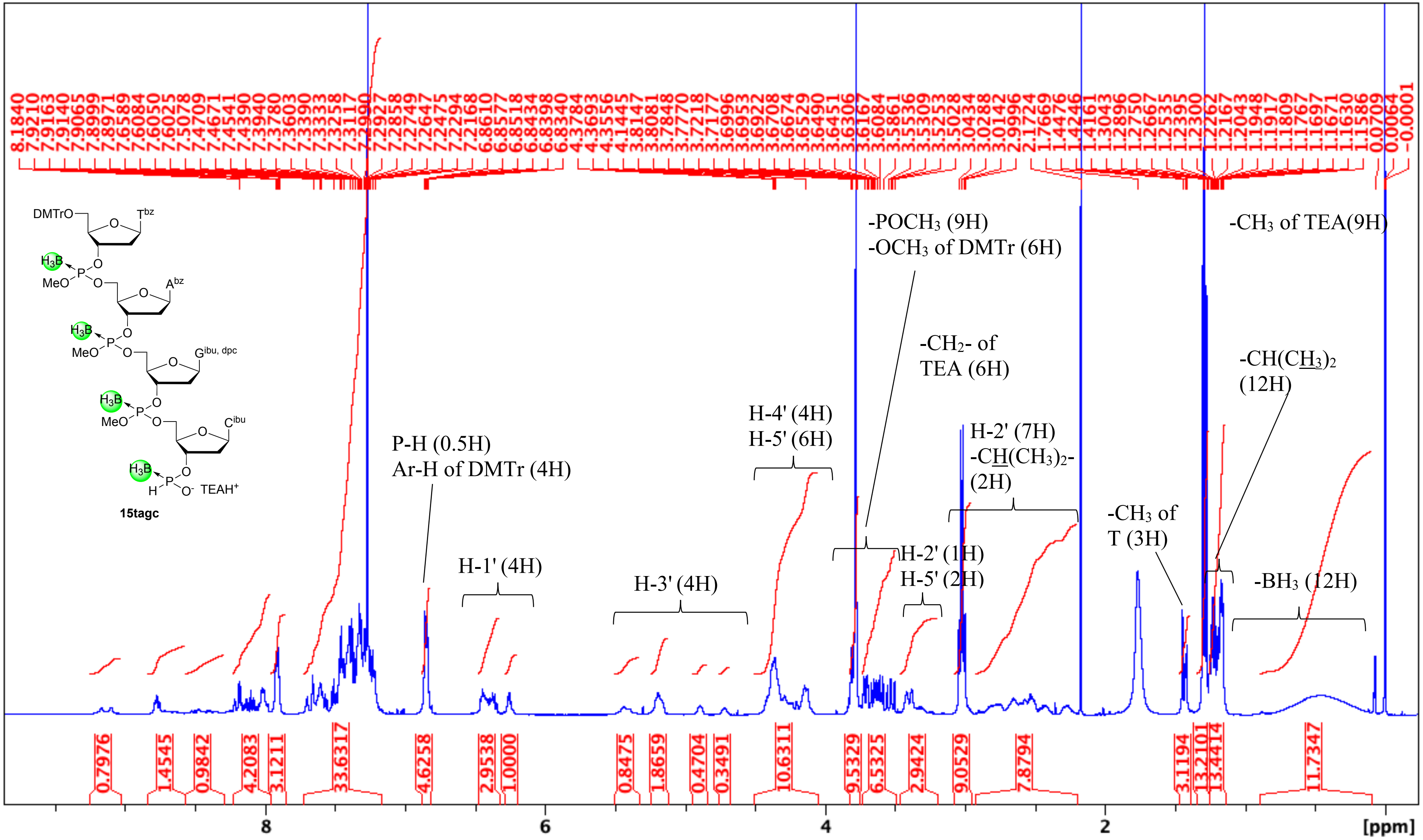

$^{13}C\{^{1}H\}$ NMR ($CDCl_3$, 126 MHz)

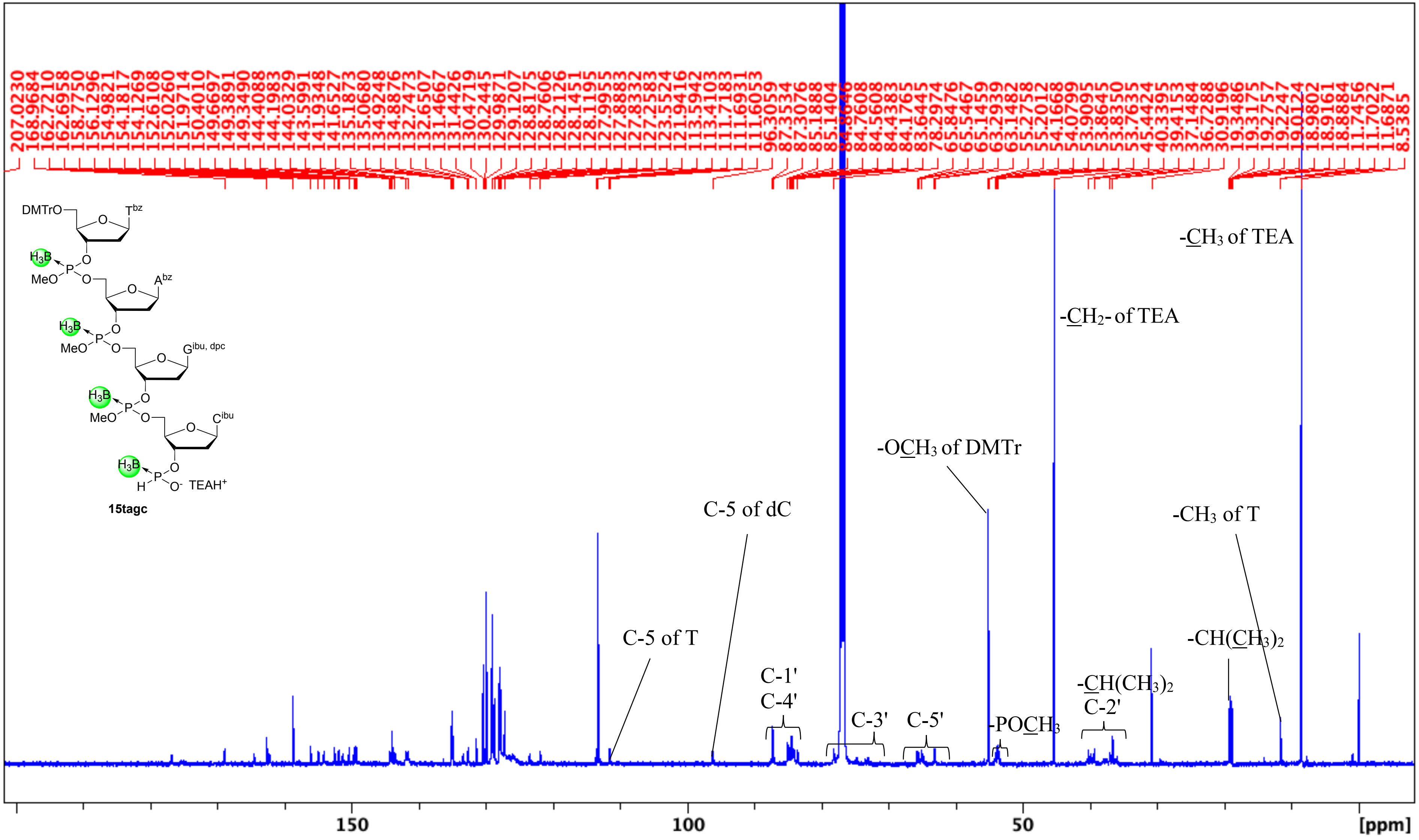

COSY ($CDCl_3$)

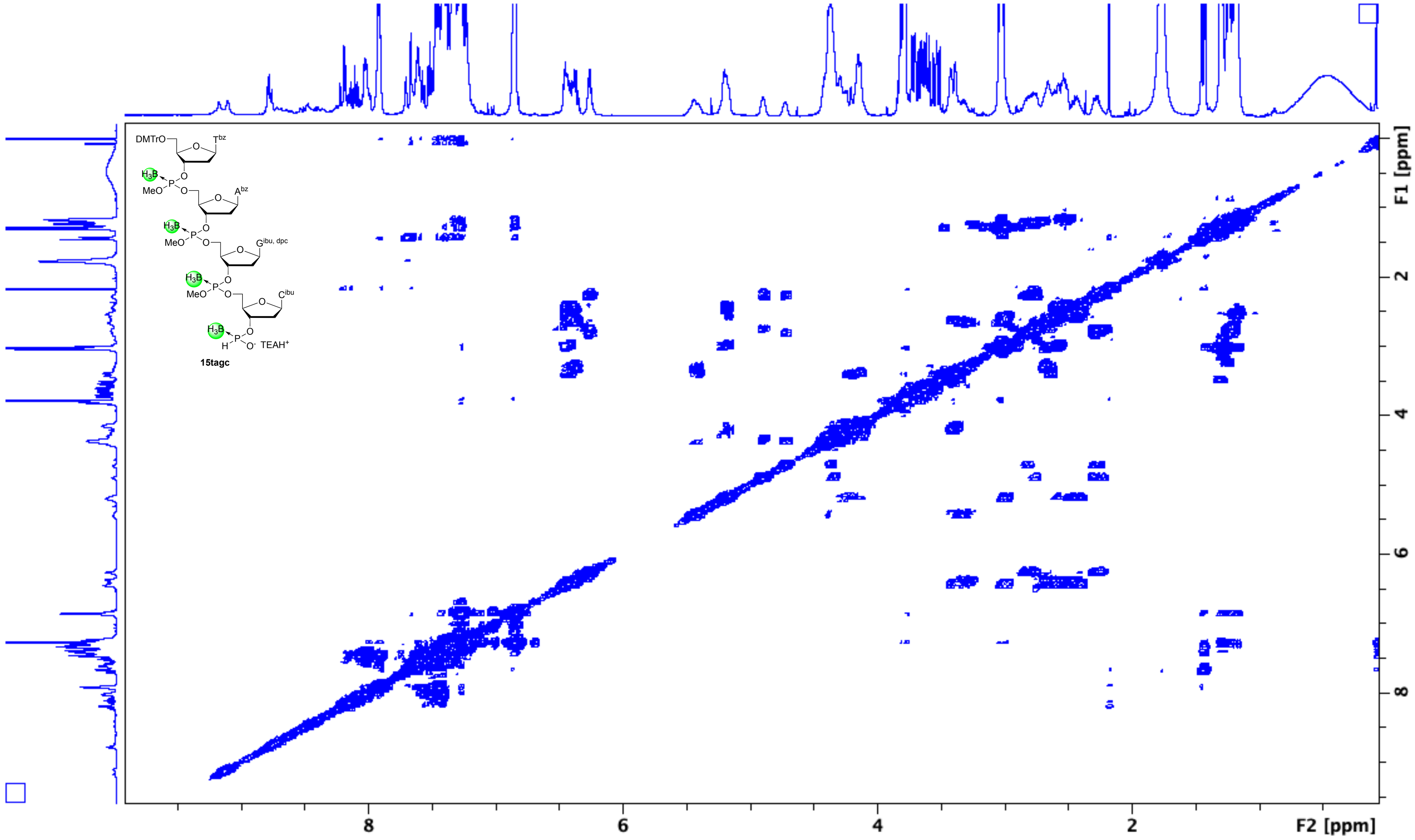

DMTrO
Tbz
H3B
MeO
Abz
Gibu, dpc
Cibu
TEAH+
15tagc
F1 [ppm]
F2 [ppm]
8
6
4
2

HSQC ($CDCl_3$)

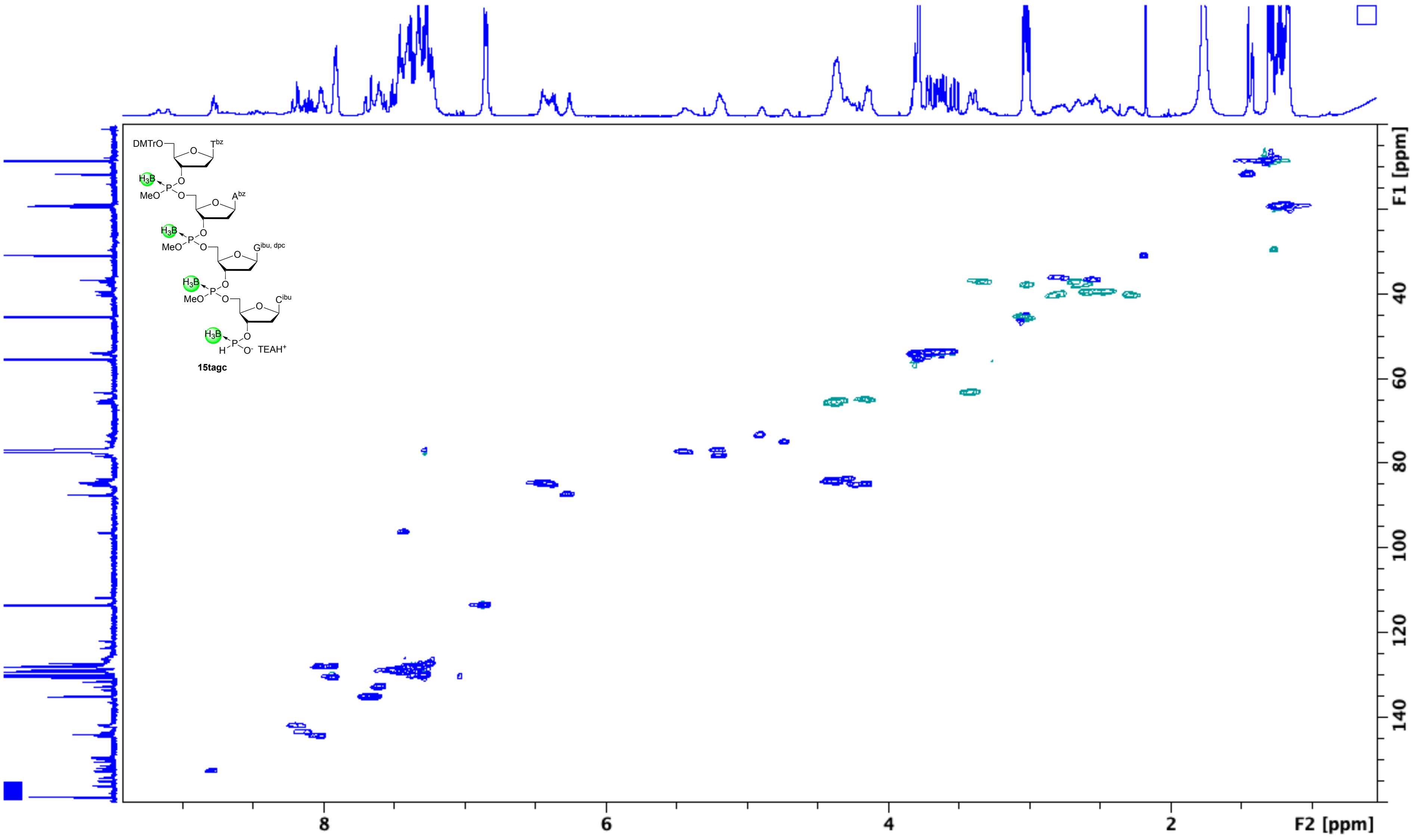

HMBC ($CDCl_3$)

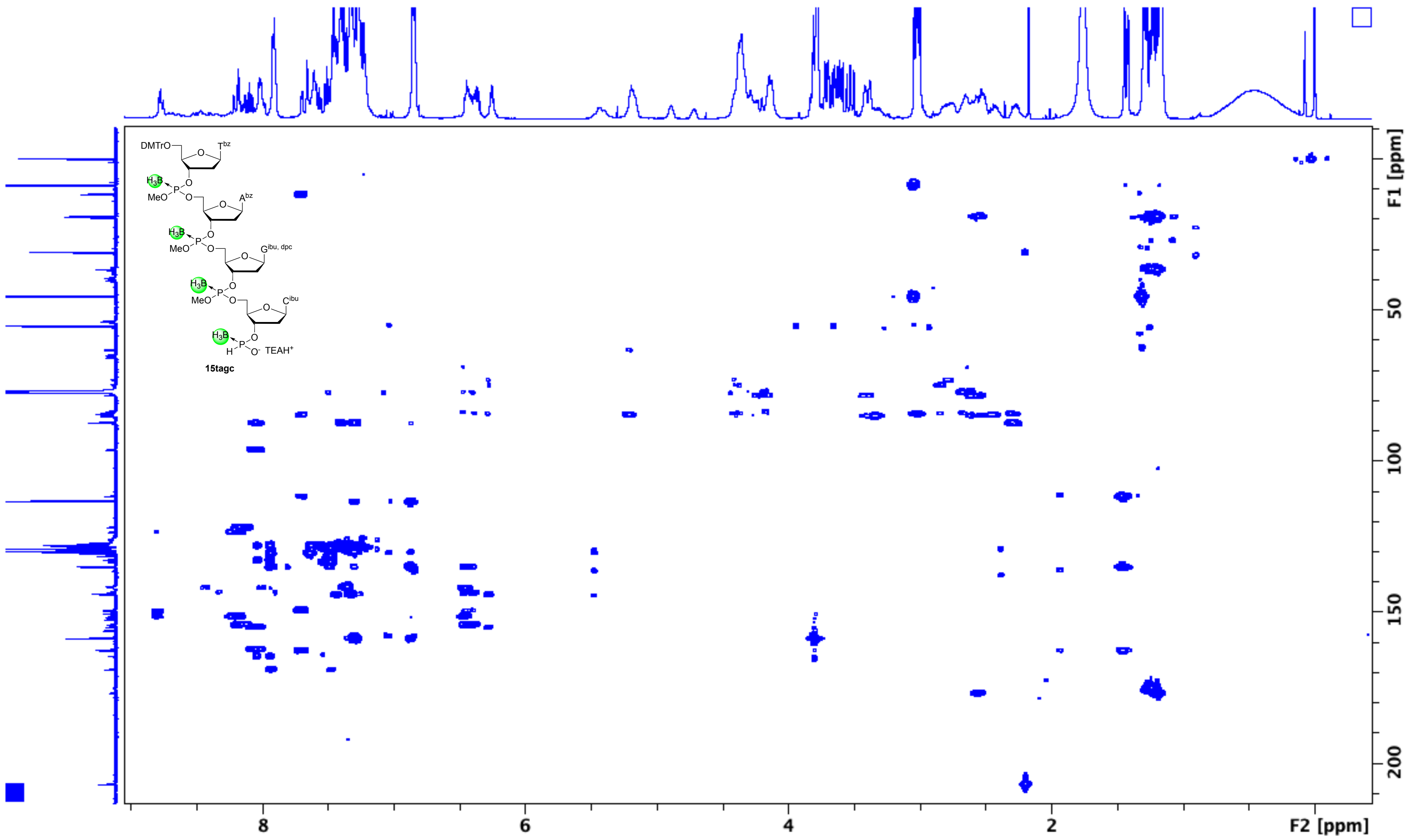

$^{31}P\{^{1}H\}$ NMR ($CDCl_3$, 202 MHz)

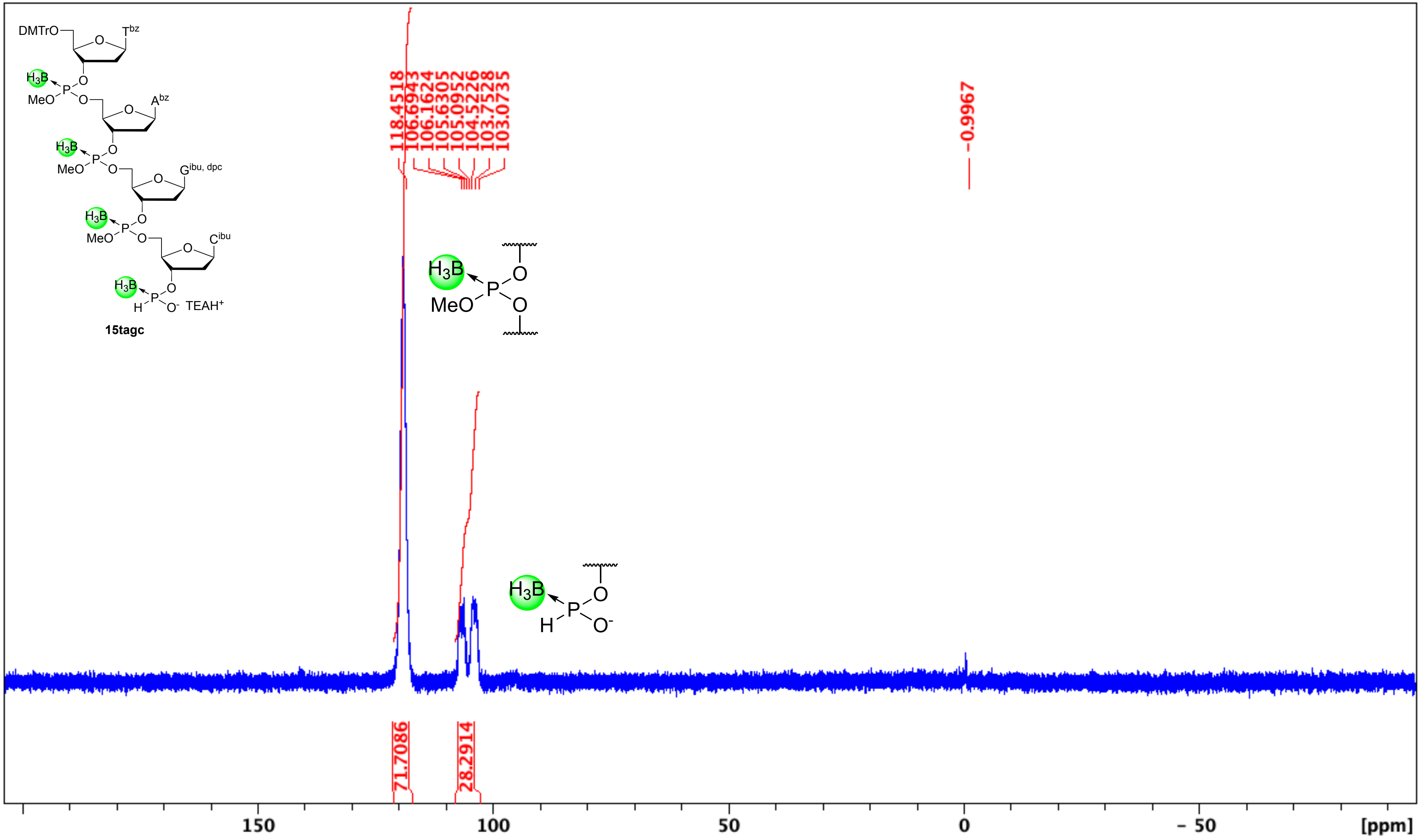

^{11}B {^{1}H} NMR ($CDCl_3$, 160 MHz)

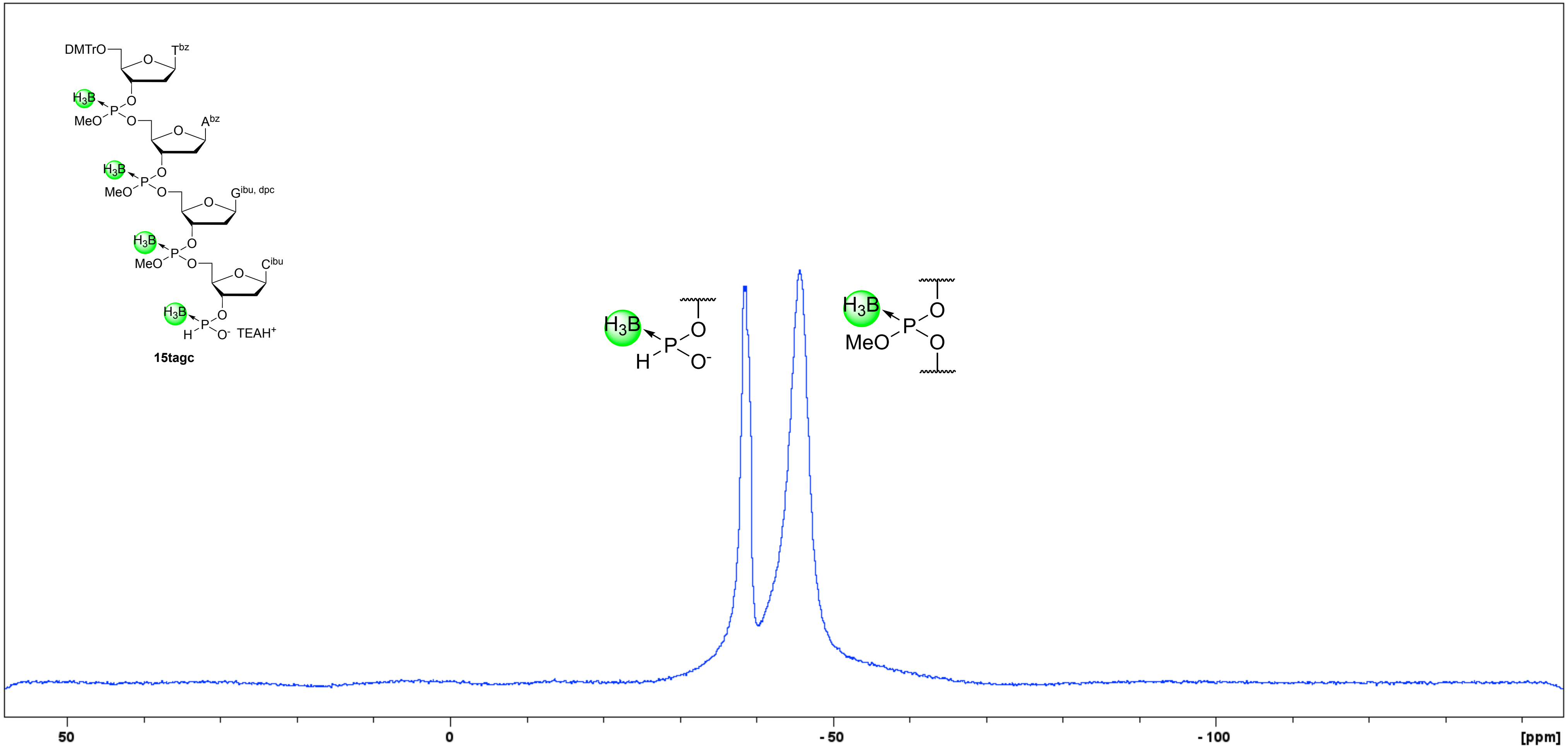

T-A-G-C-T-A-G-C-T-A-G-C 12-mer (18)

^{1}H NMR (500 MHz, D_2O)

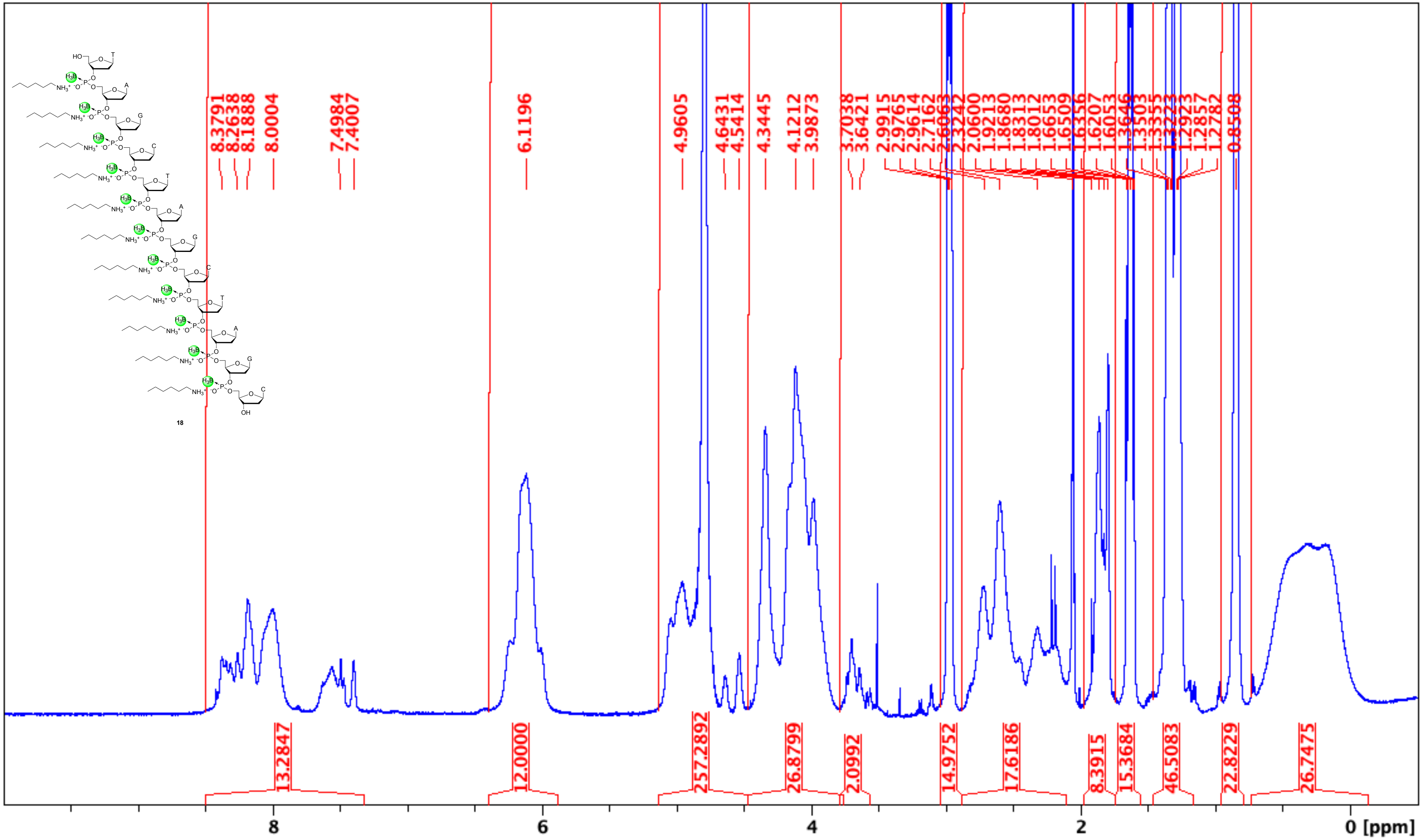

$^{13}C\{^1H\}$ NMR (126 MHz, D_2O)

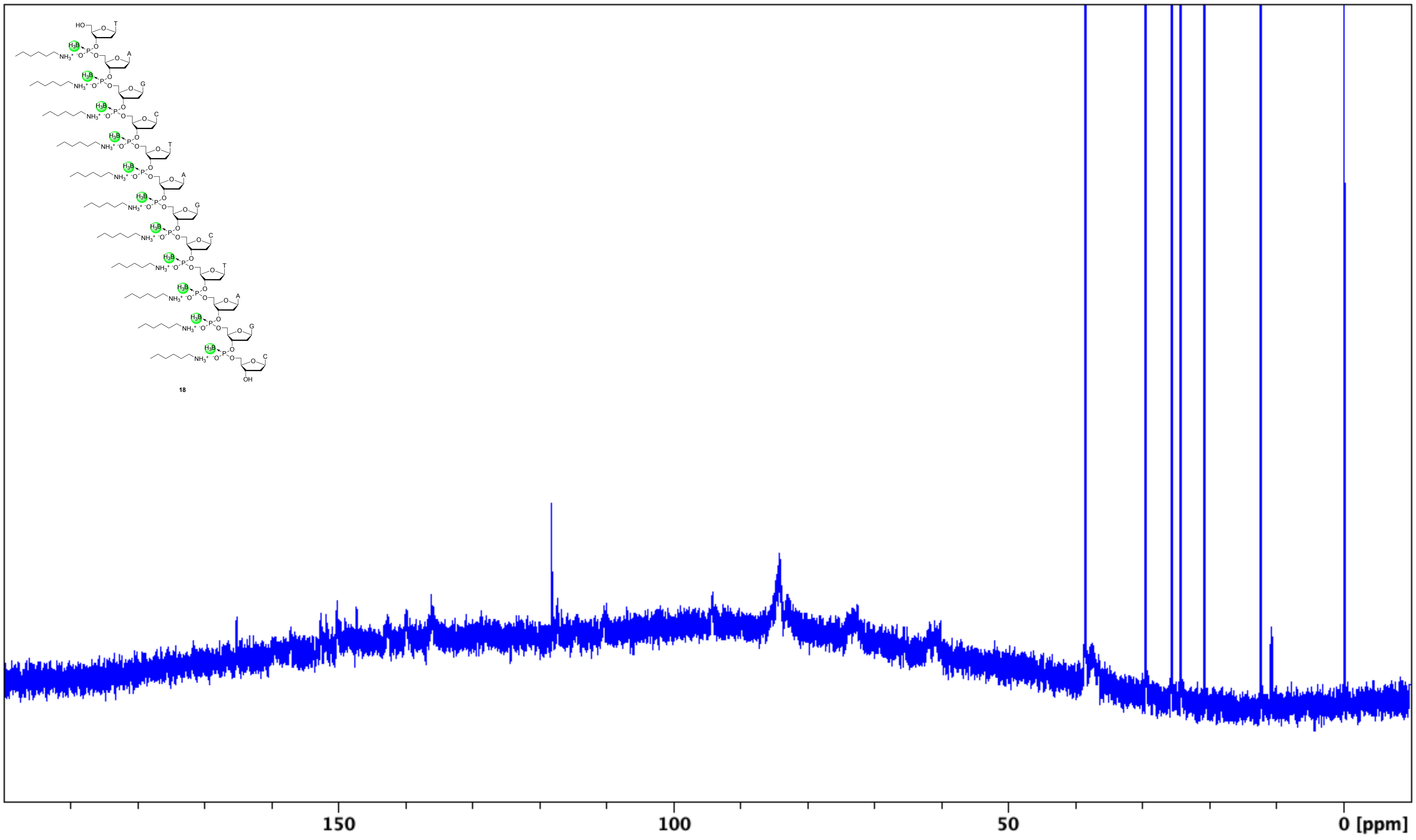

$^{31}P\{^1H\}$ NMR (202 MHz, D_2O)

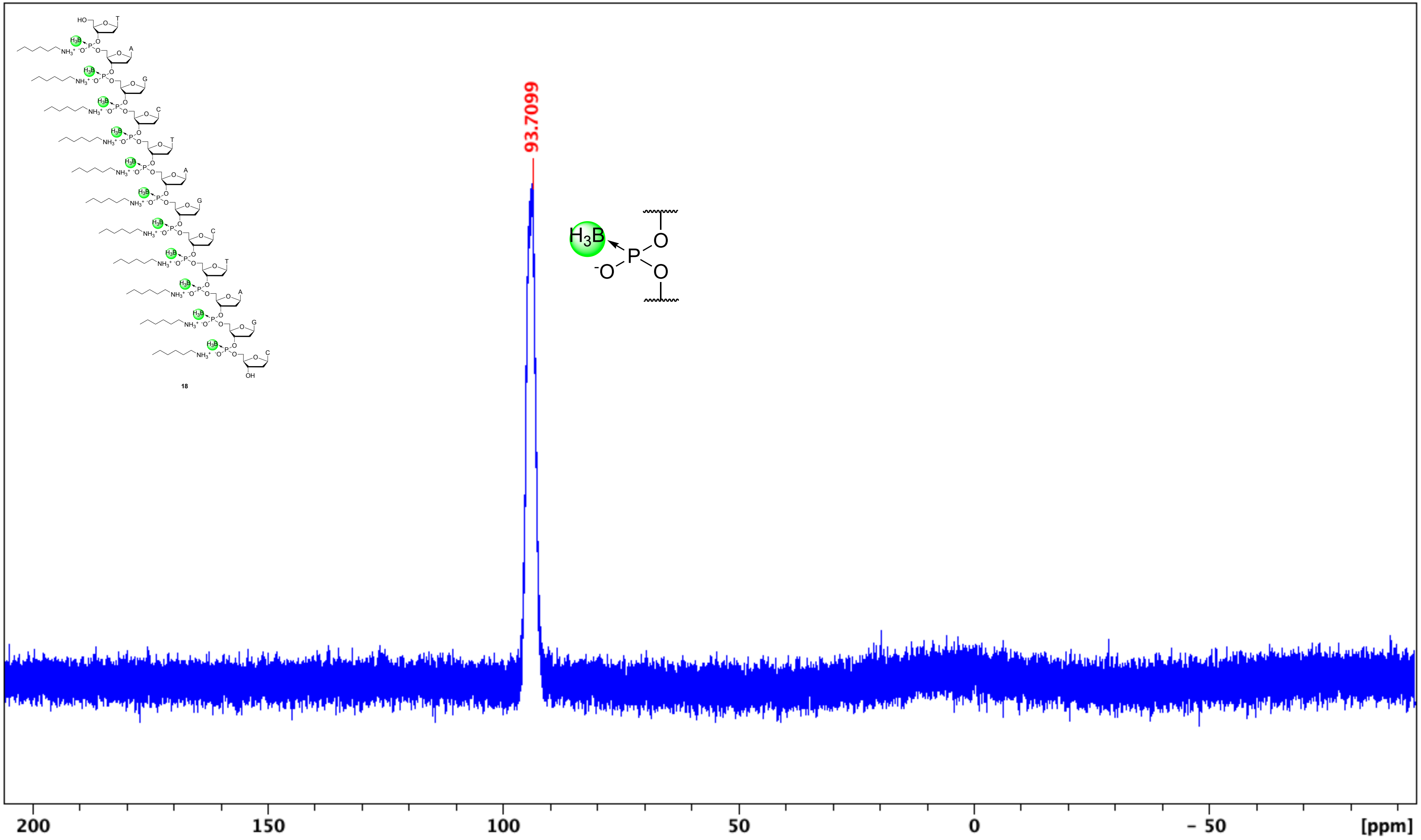

$^{11}B\{^{1}H\}$ NMR (160 MHz, D_2O)

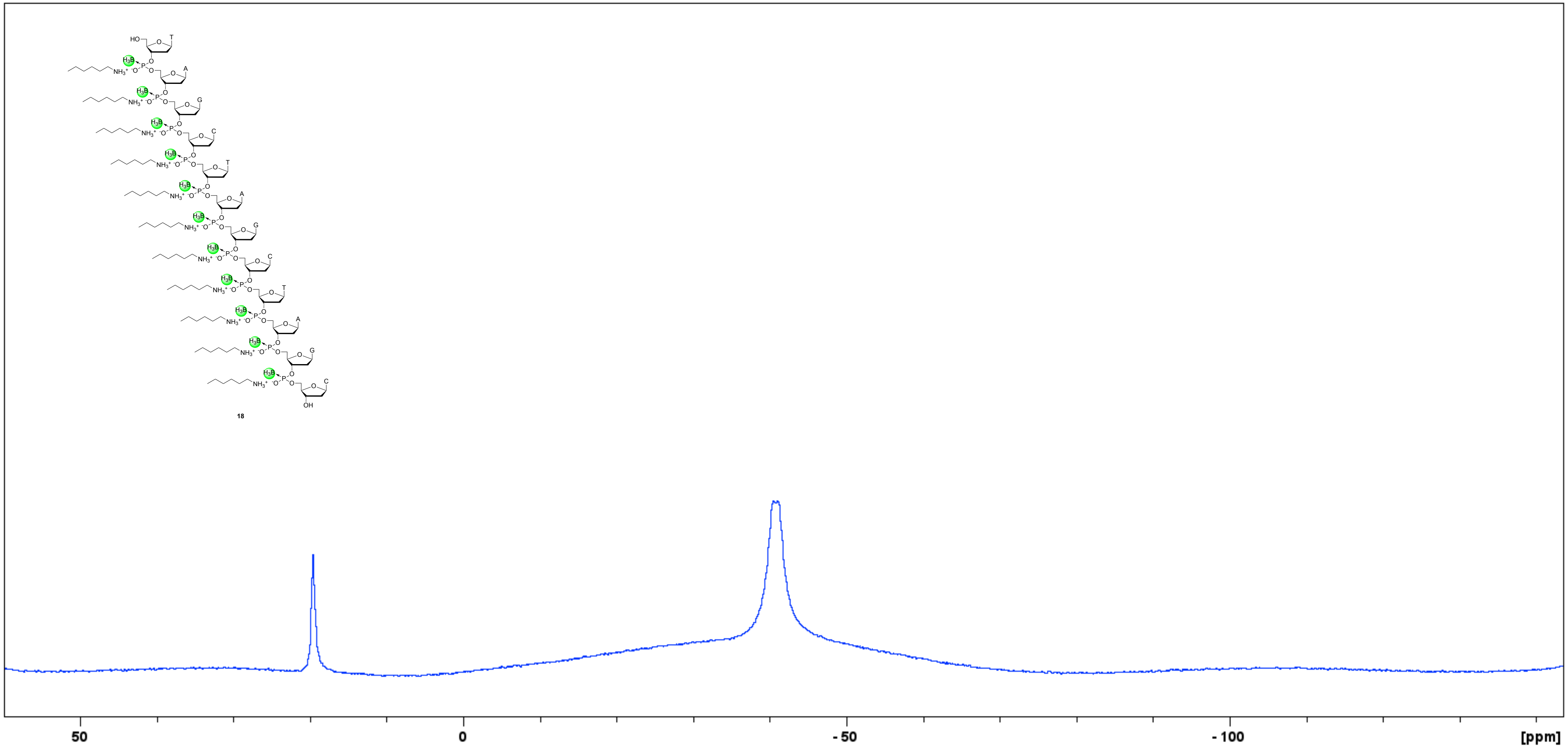

A-T 2-mer building block bearing H-boranophosphonate monoester on 3'-OH group (23at)

^{1}H NMR ($CDCl_3$, 500 MHz)

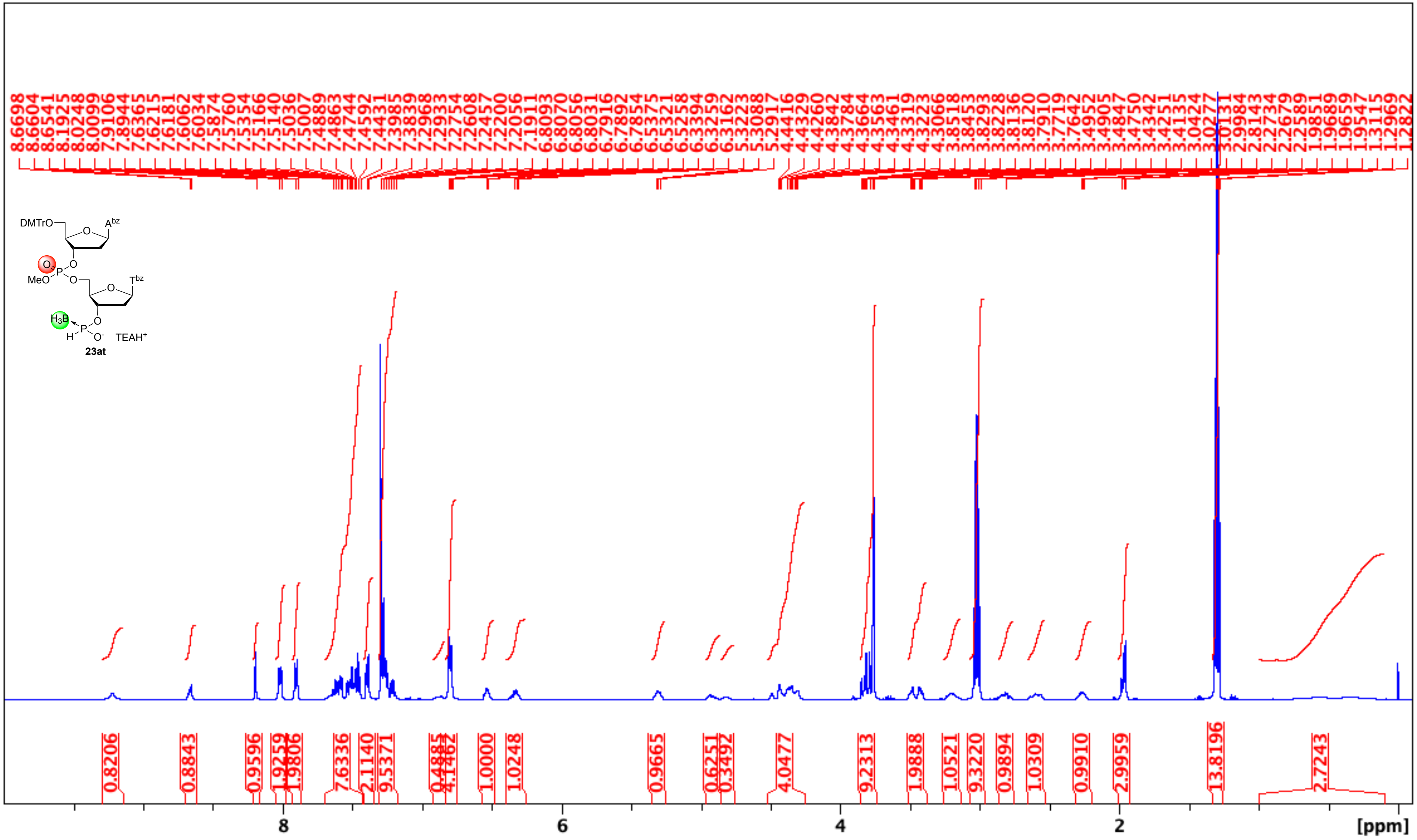

$^{13}C\{^1H\}$ NMR ($CDCl_3$, 126 MHz)

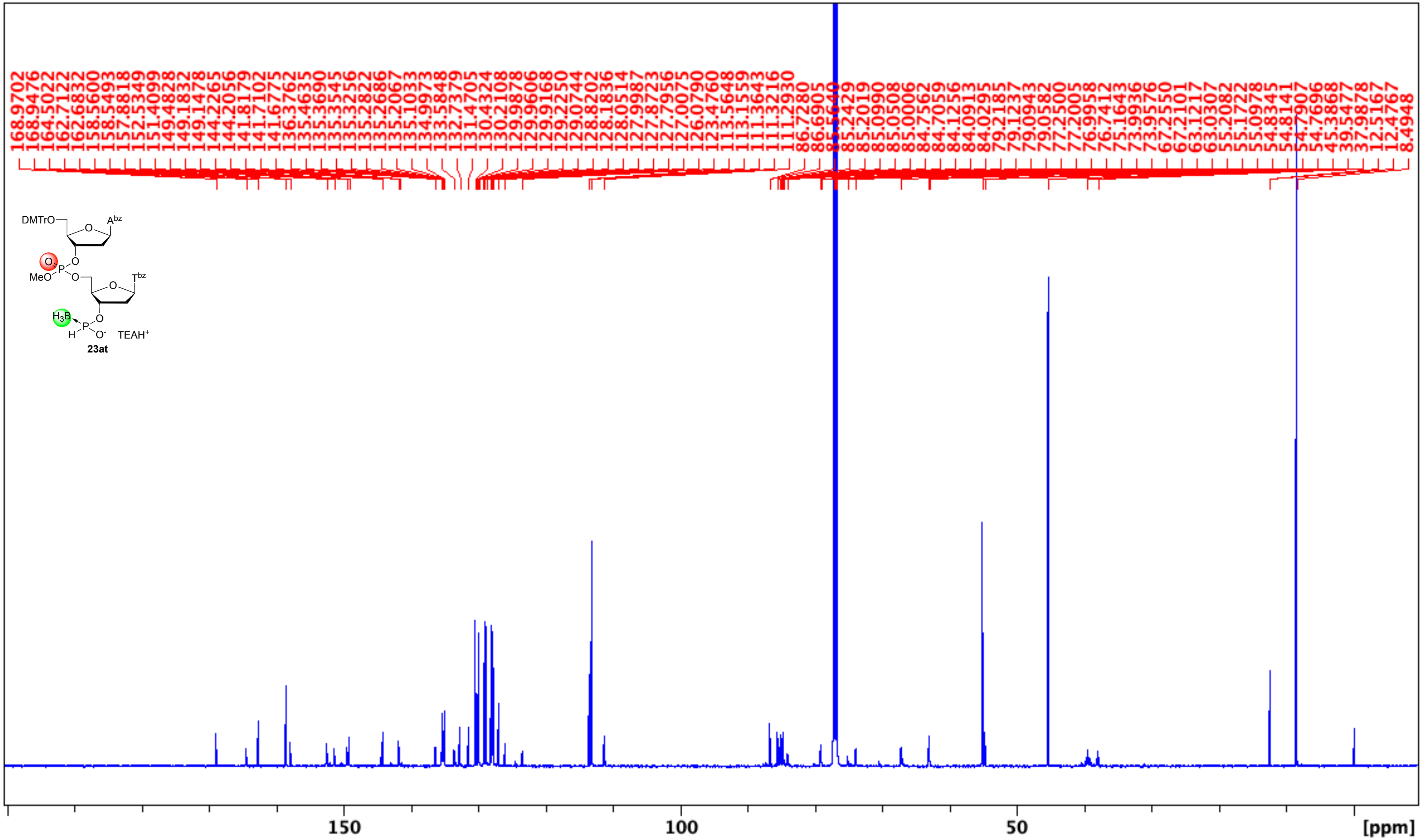

COSY ($CDCl_3$)

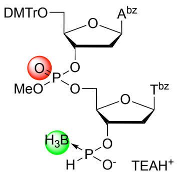

23at

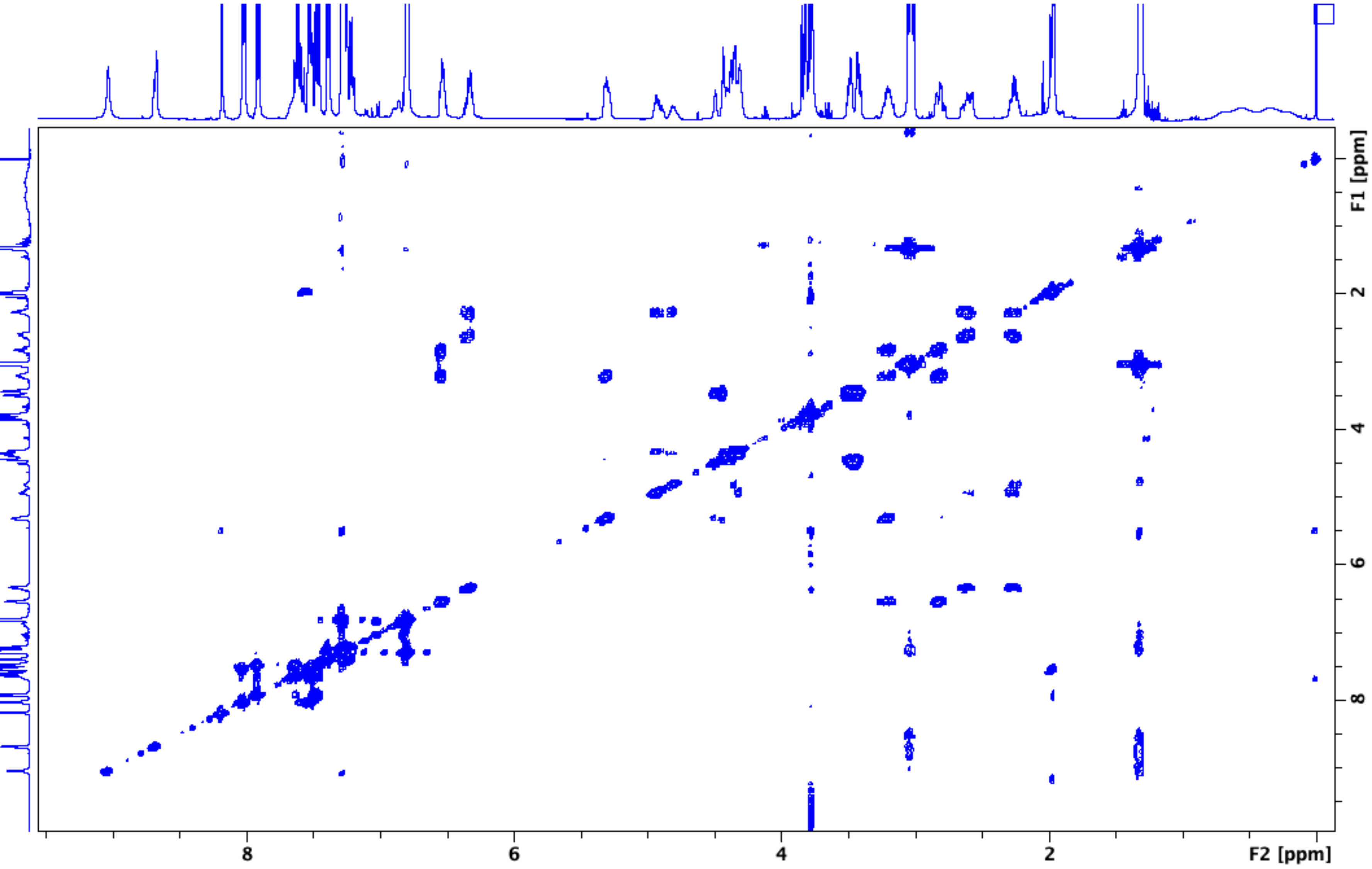

HSQC ($CDCl_3$)

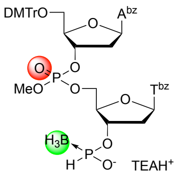

23at

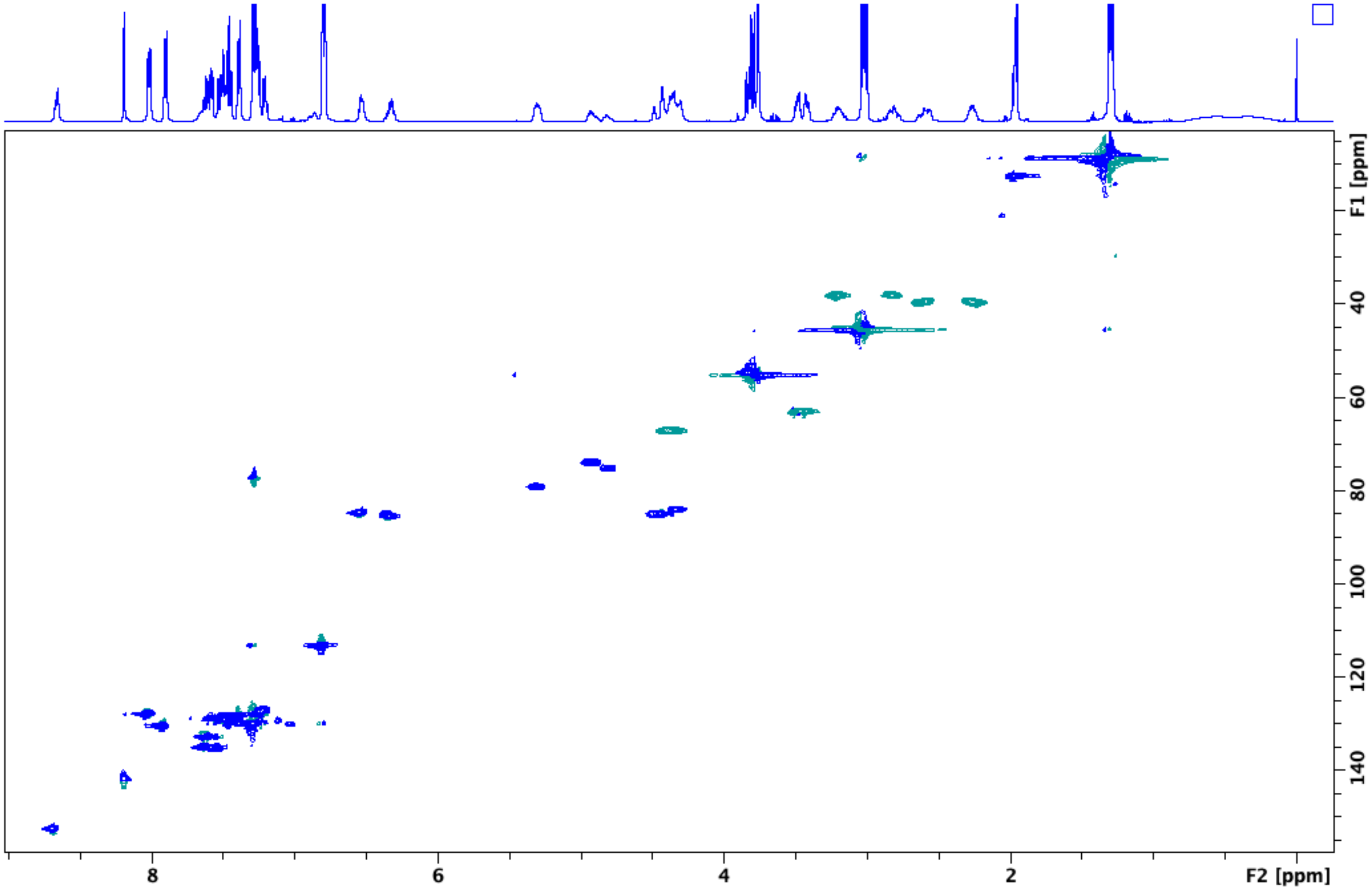

HMBC ($CDCl_3$)

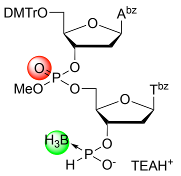

DMTrO
O
A^bz
O
O
P
MeO
O
O
T^bz
H3B
P
H
O
O-
TEAH+
23at

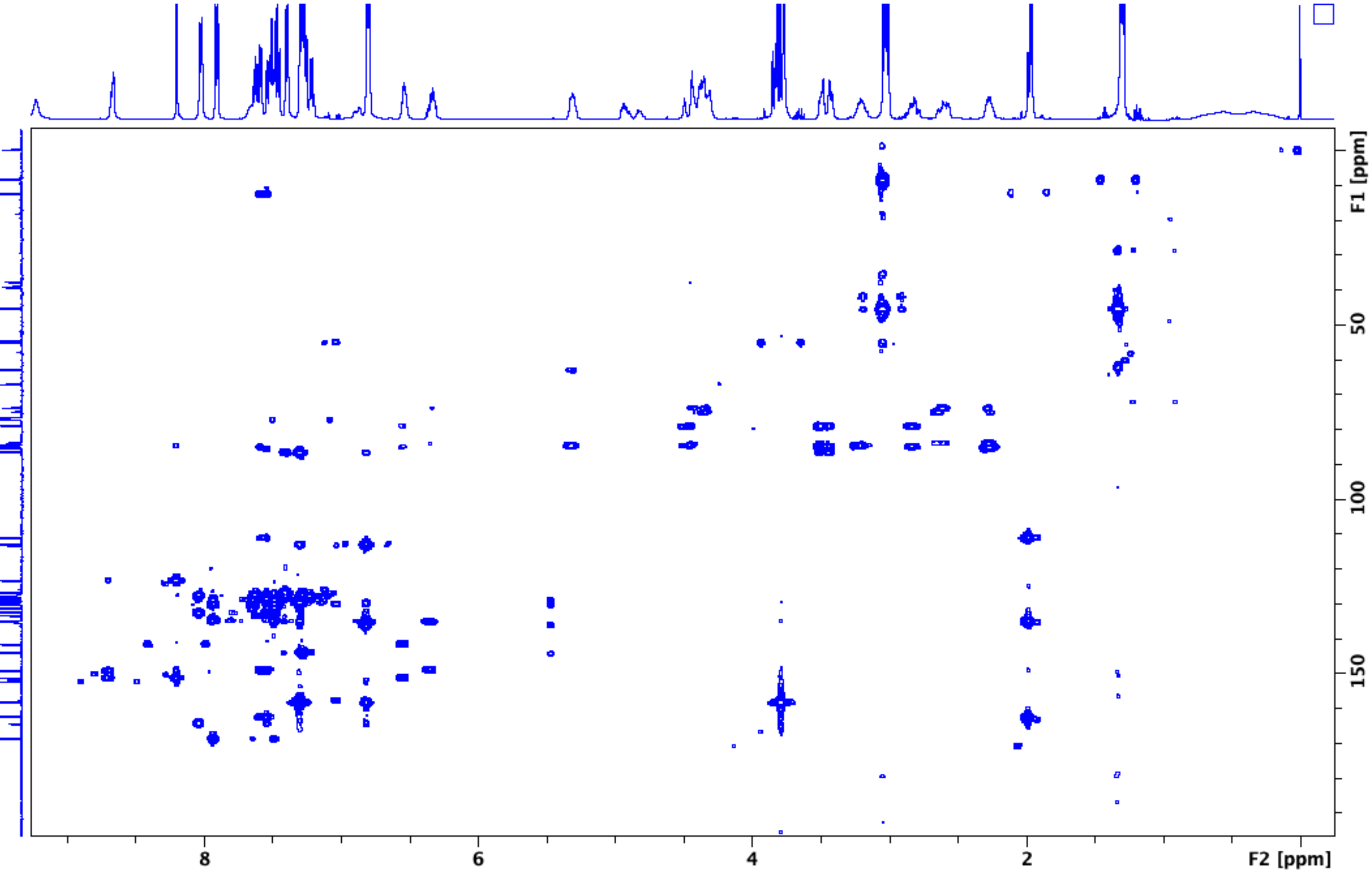

F1 [ppm]
50
100
150
8
6
4
2
F2 [ppm]

$^{31}P\{^1H\}$ NMR ($CDCl_3$, 202 MHz)

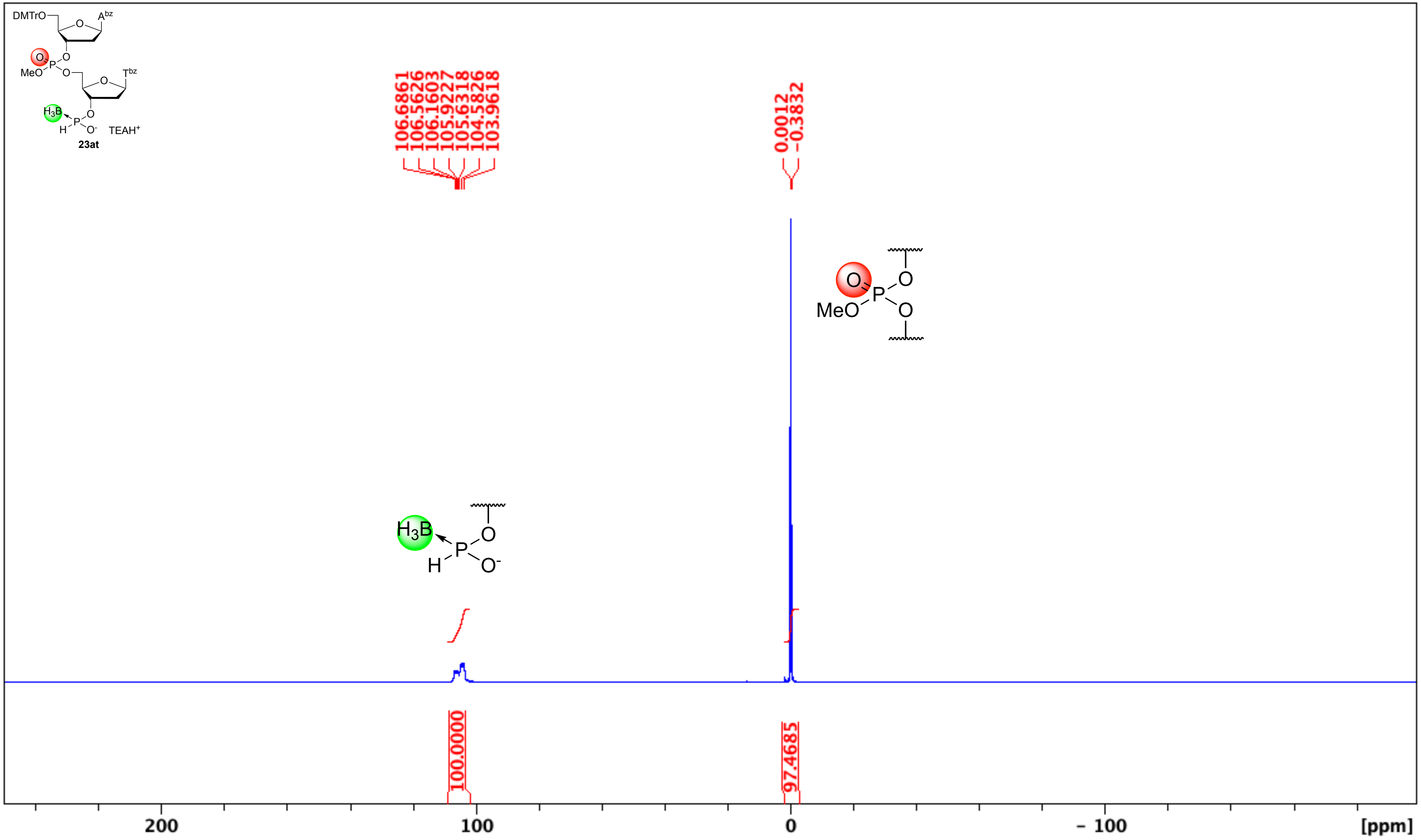

C-G 2-mer building block bearing 5'-OH group (27cg)

^{1}H NMR ($CDCl_3$, 500 MHz)

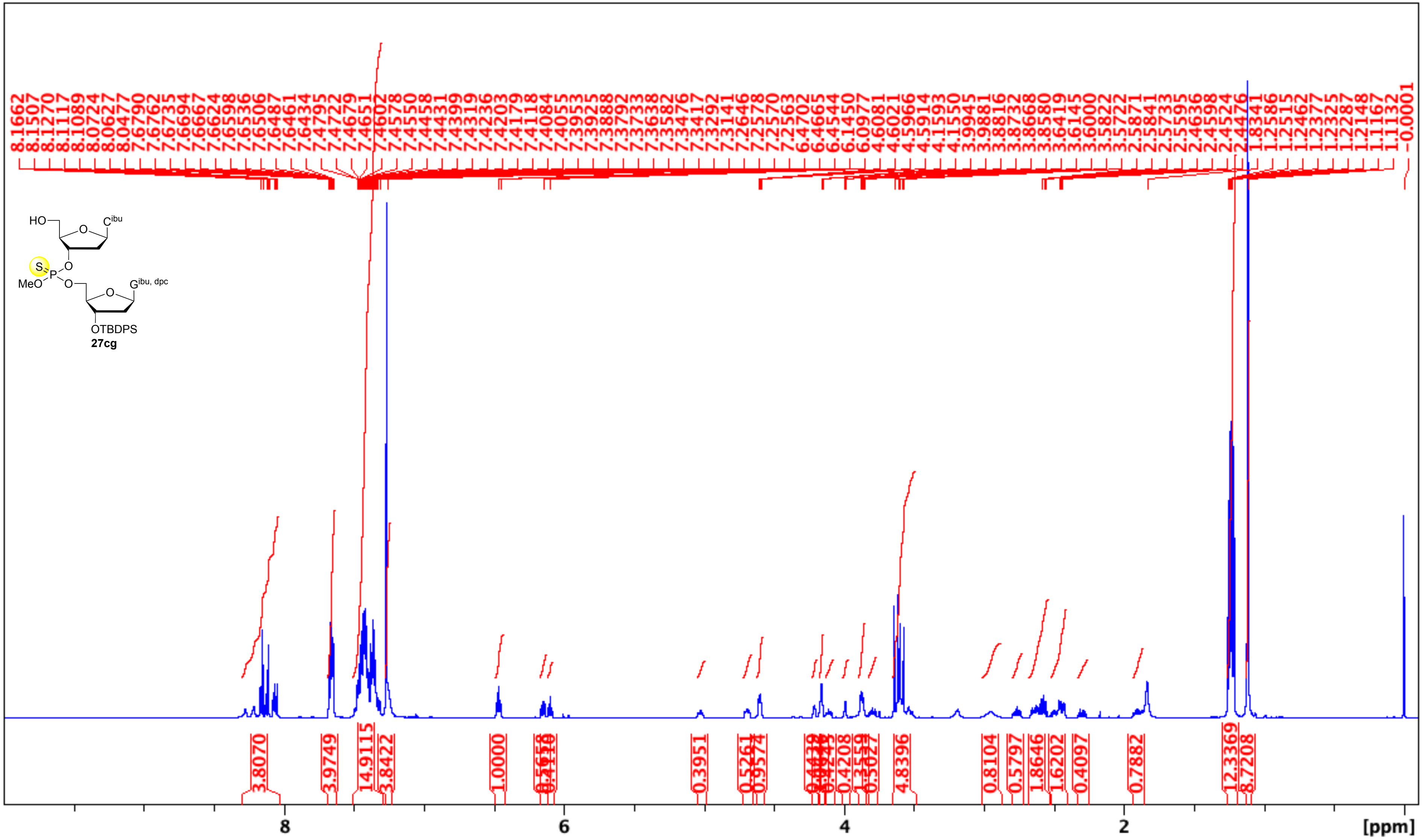

^{1}H NMR ($CDCl_3$, 500 MHz, zoom)

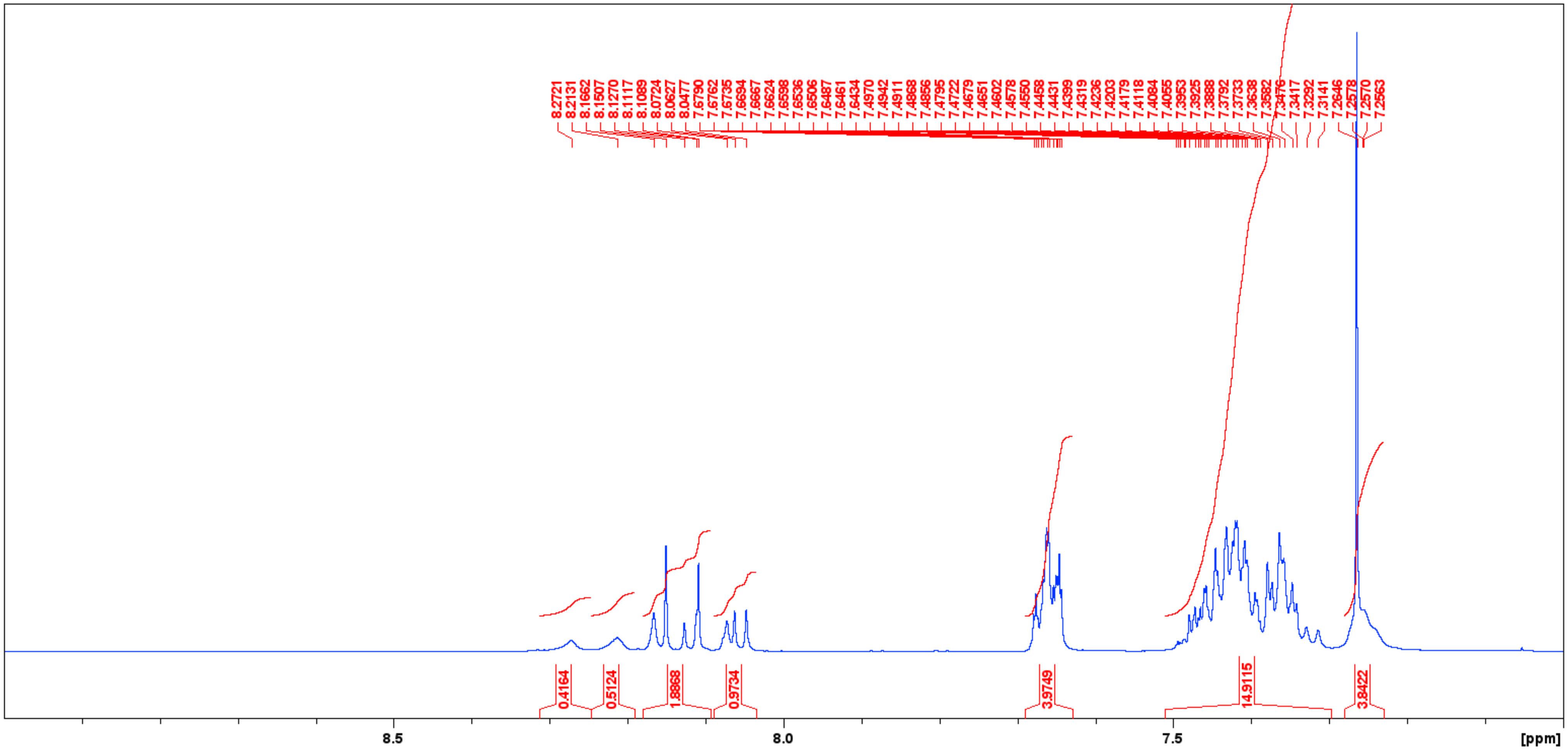

^{1}H NMR ($CDCl_3$, 500 MHz, zoom)

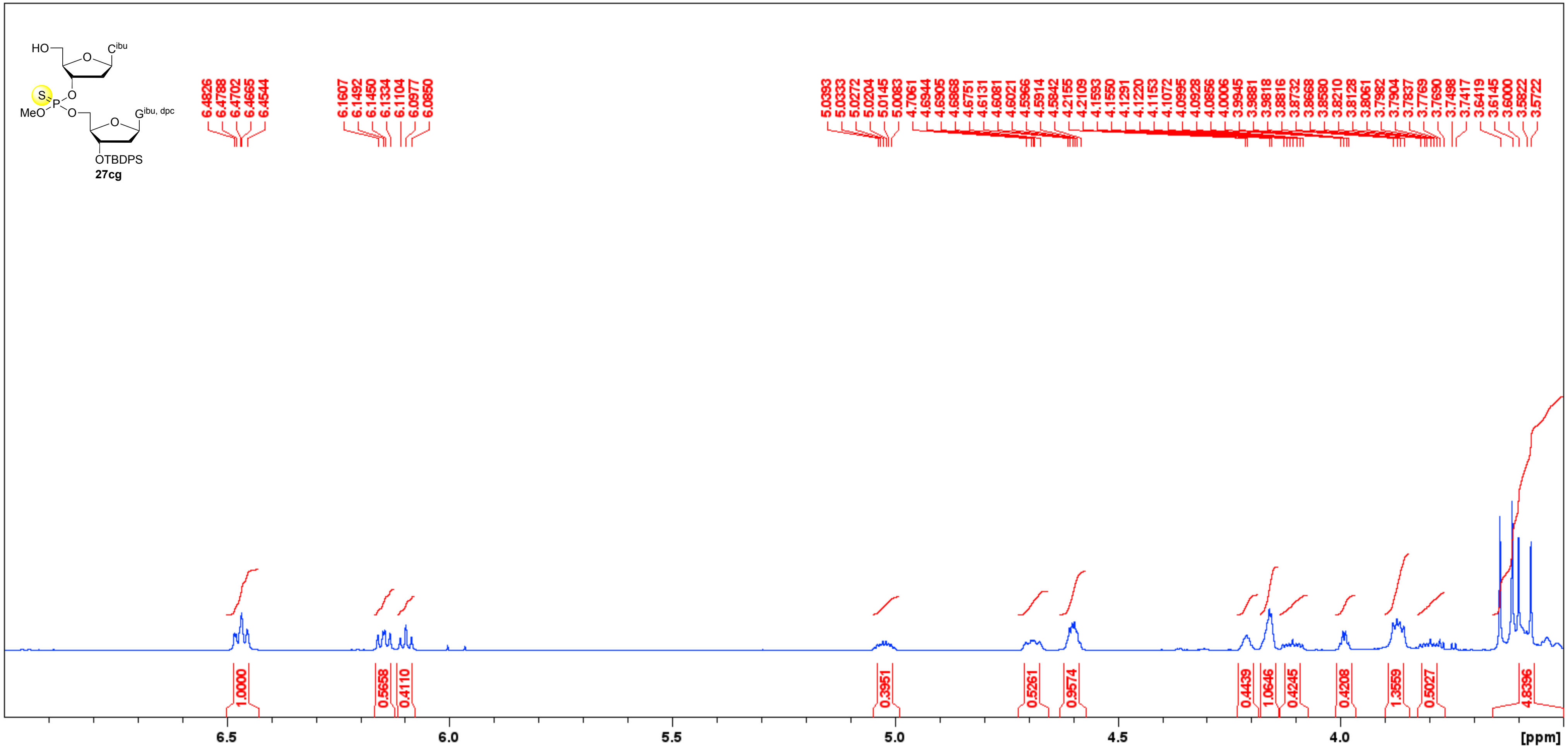

$^{13}C\{^{1}H\}$ NMR ($CDCl_3$, 126 MHz)

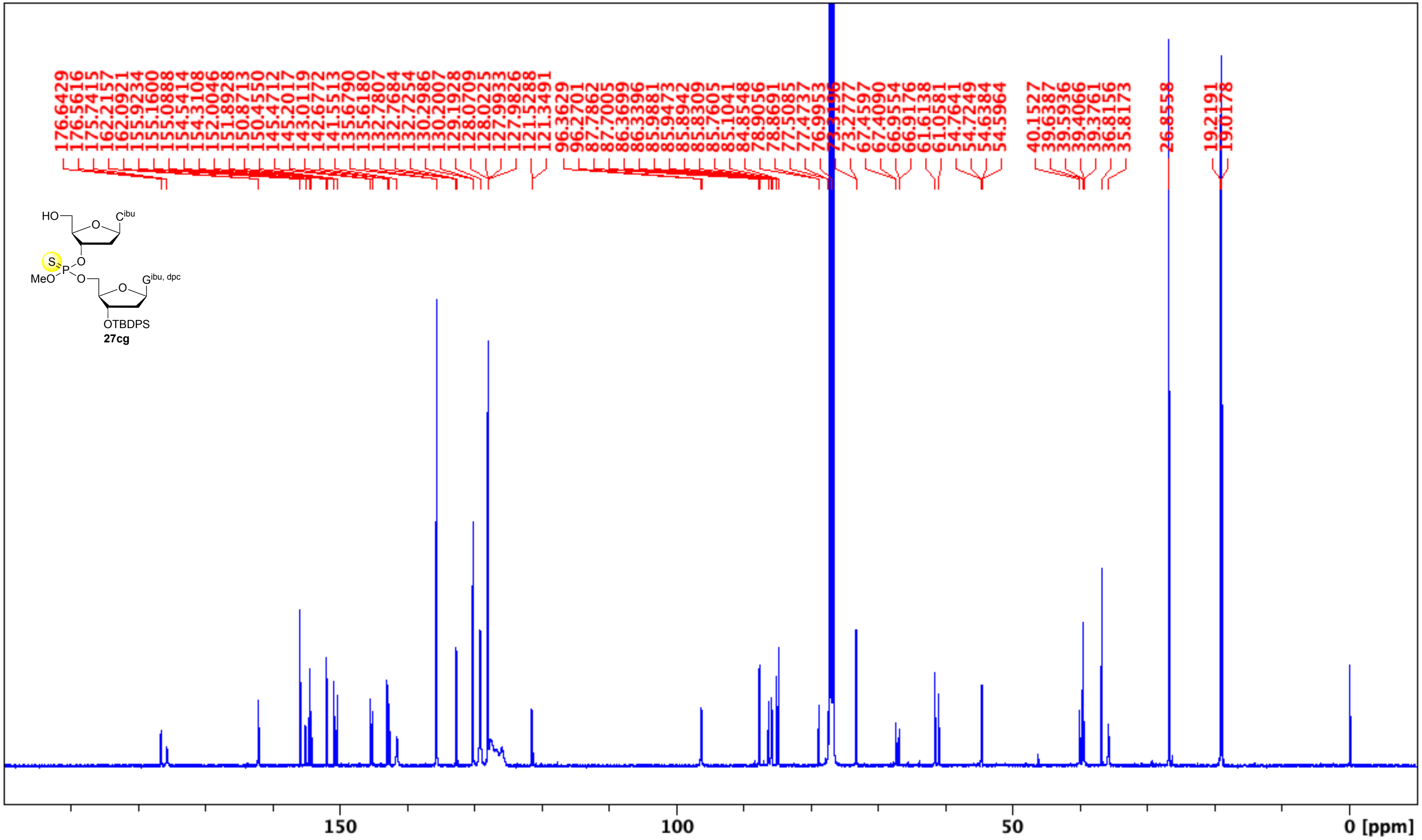

COSY ($CDCl_3$)

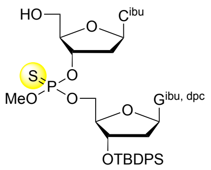

27cg

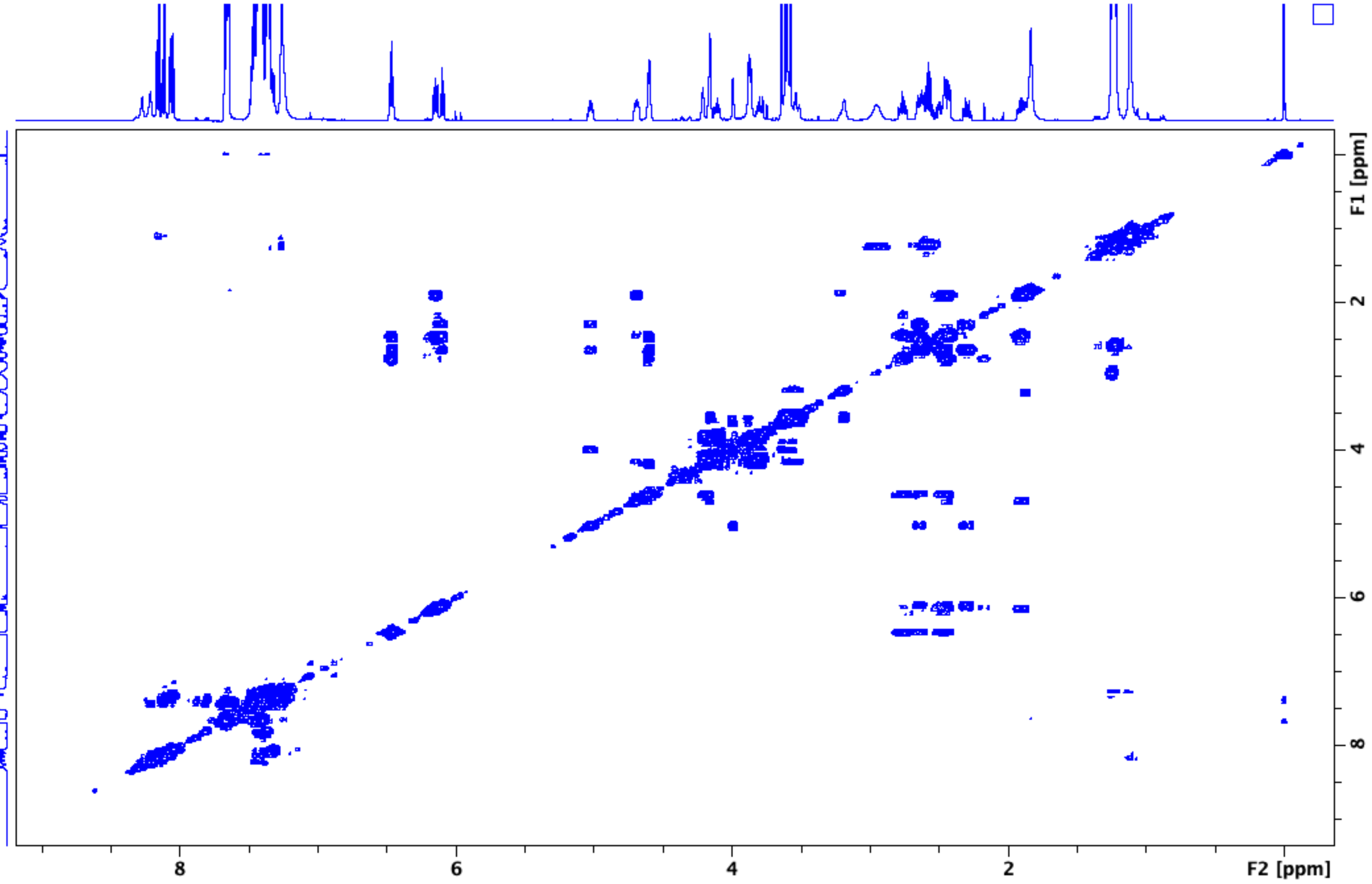

HSQC ($CDCl_3$)

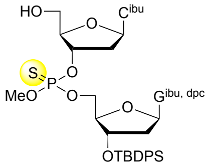

27cg

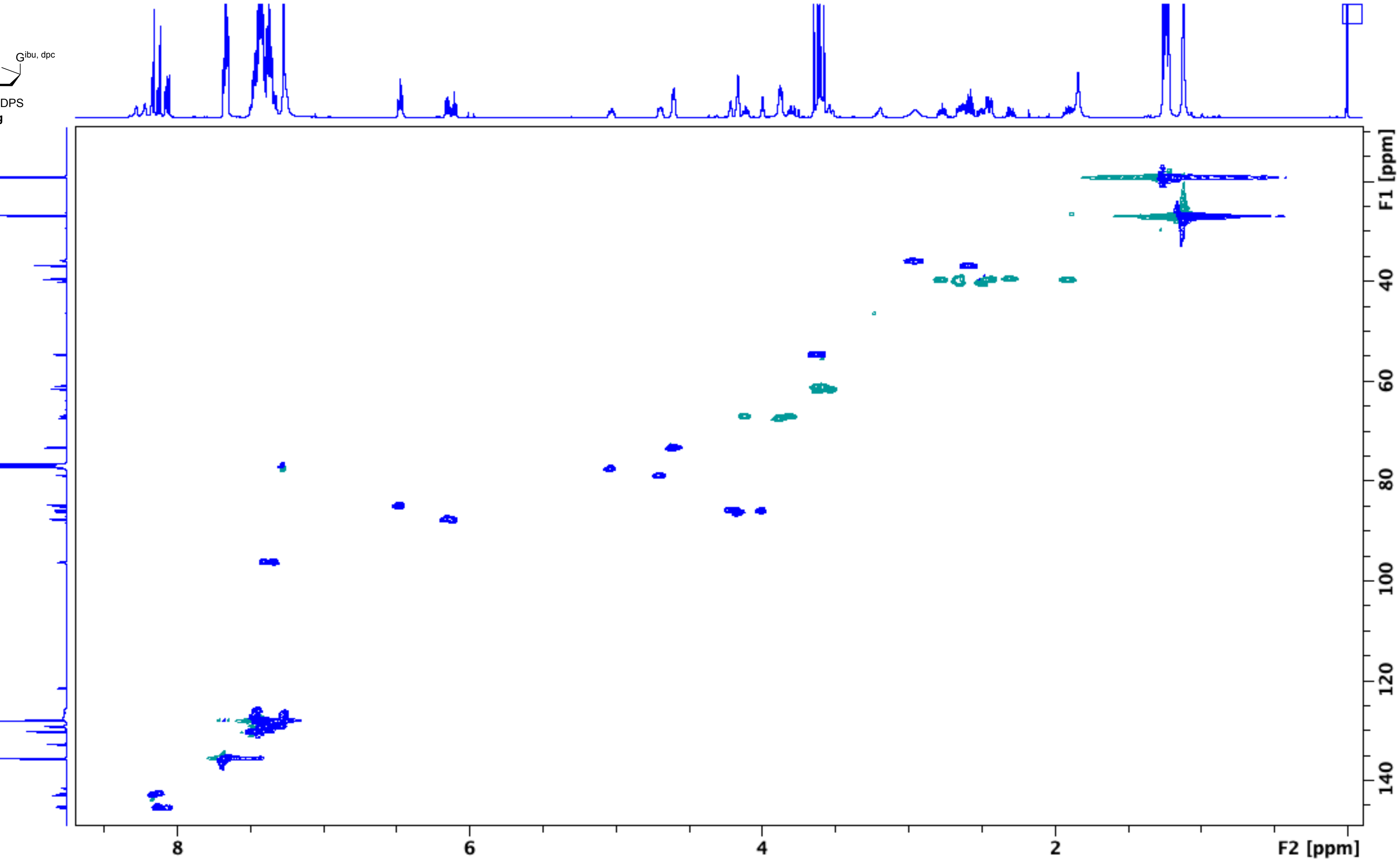

HMBC ($CDCl_3$)

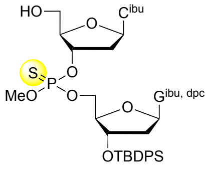

27cg

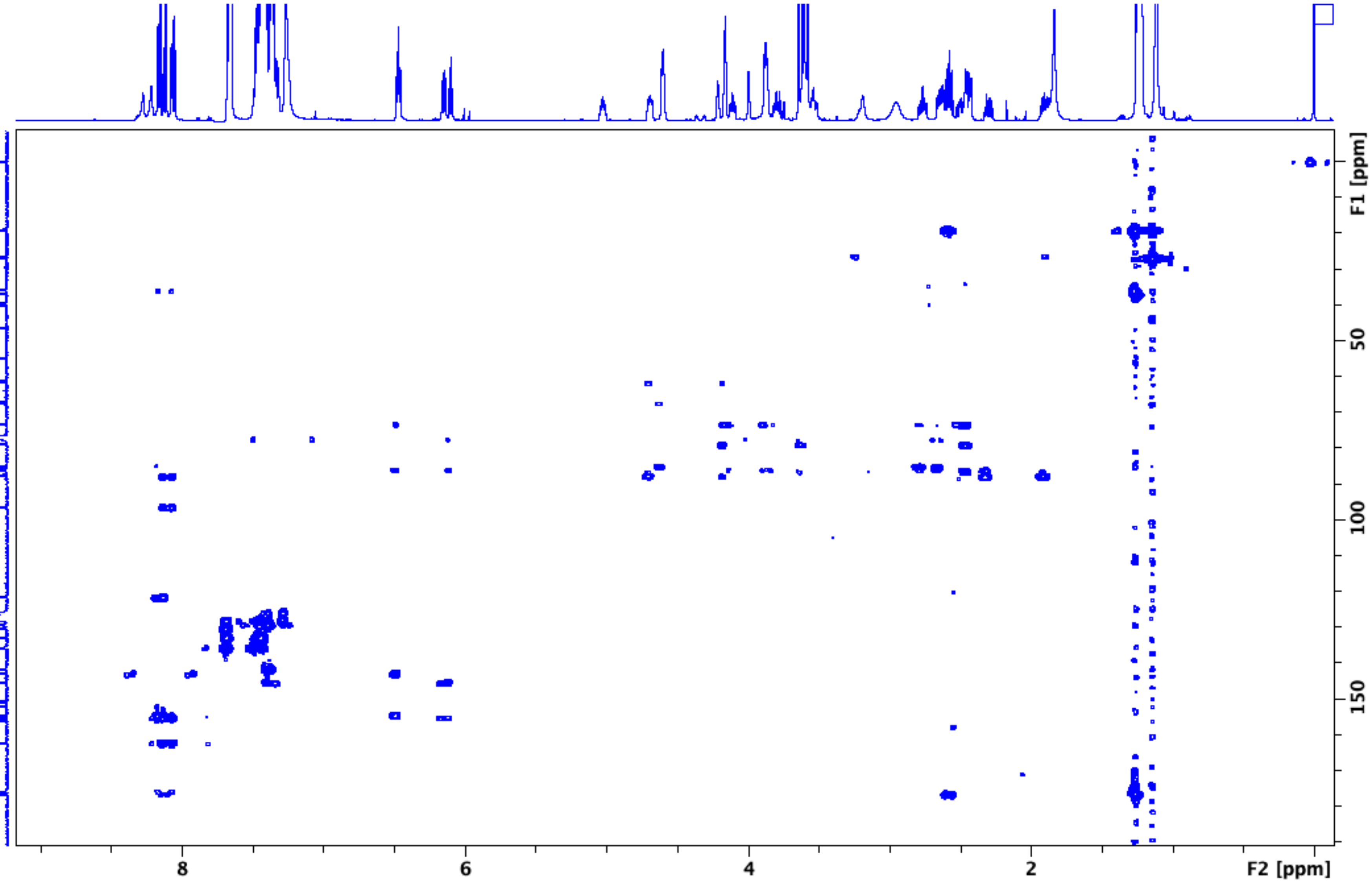

$^{31}P\{^1H\}$ NMR ($CDCl_3$, 202 MHz)

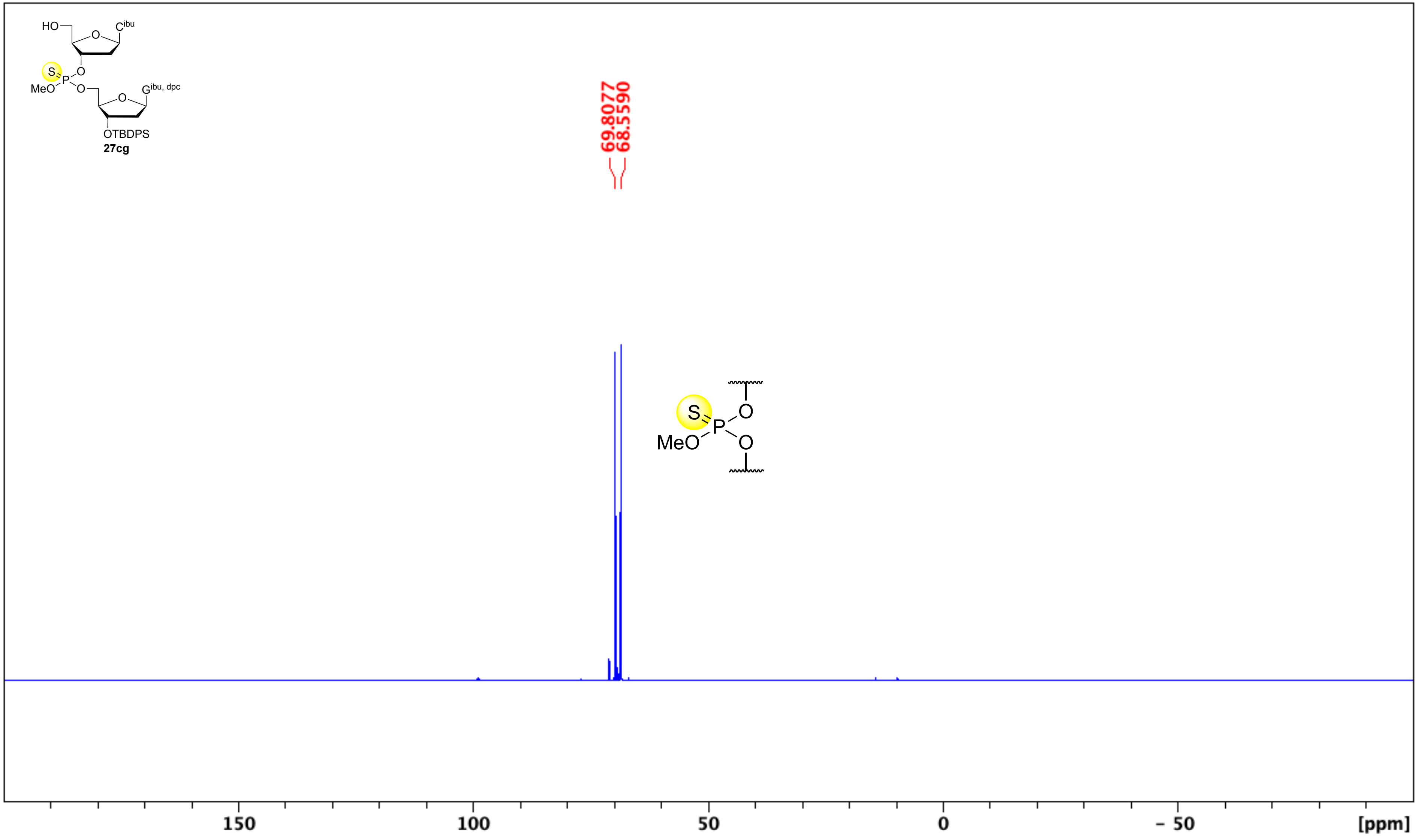

A-T-C-G 4-mer building block bearing 5'-OH group (29atcg)

^{1}H NMR ($CDCl_3$, 500 MHz)

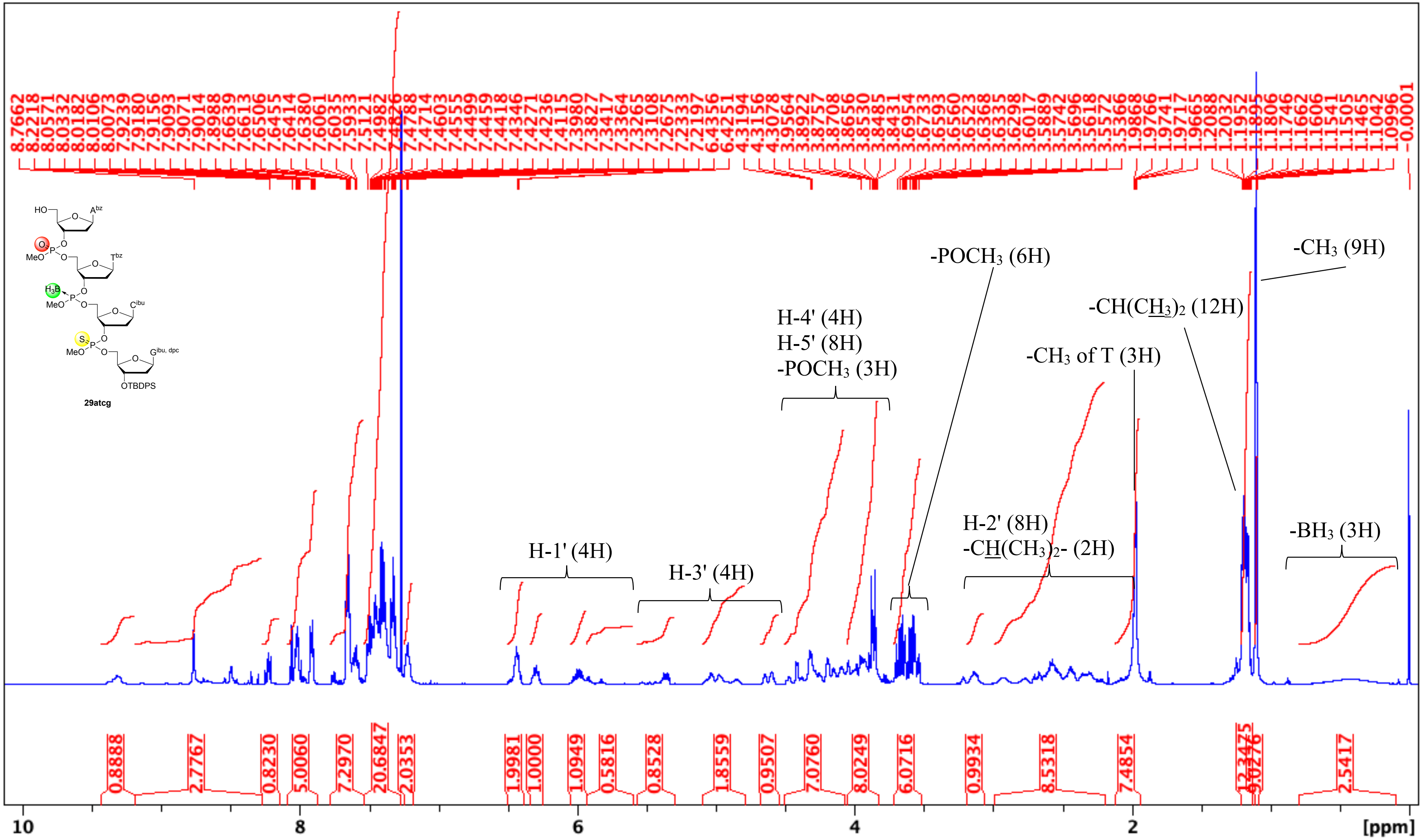

$^{13}C\{^{1}H\}$ NMR ($CDCl_3$, 126 MHz)

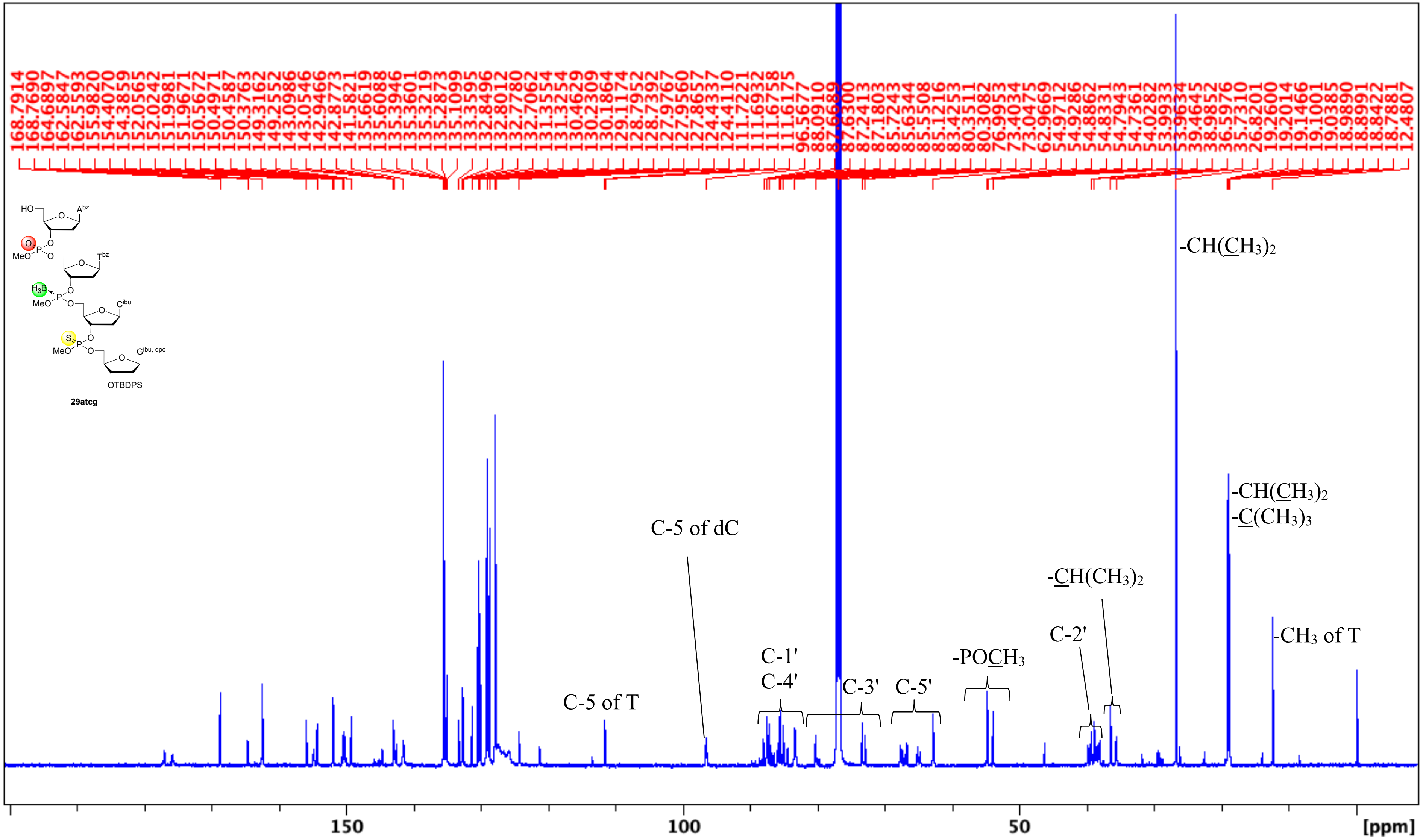

COSY ($CDCl_3$)

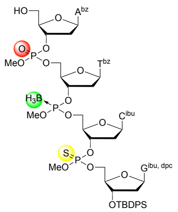

29atcg

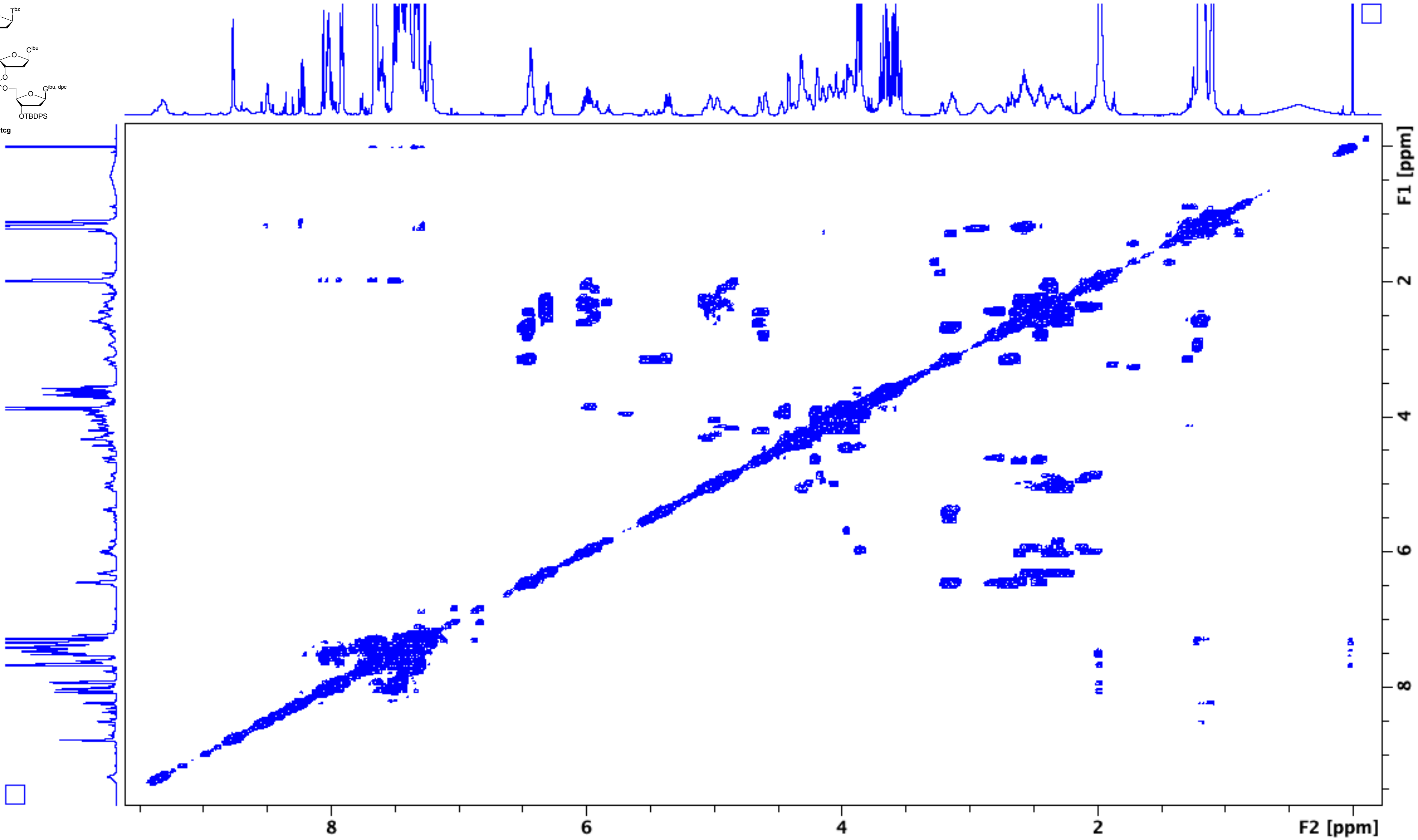

HSQC ($CDCl_3$)

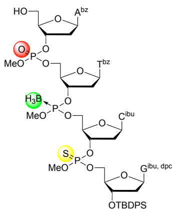

29atcg

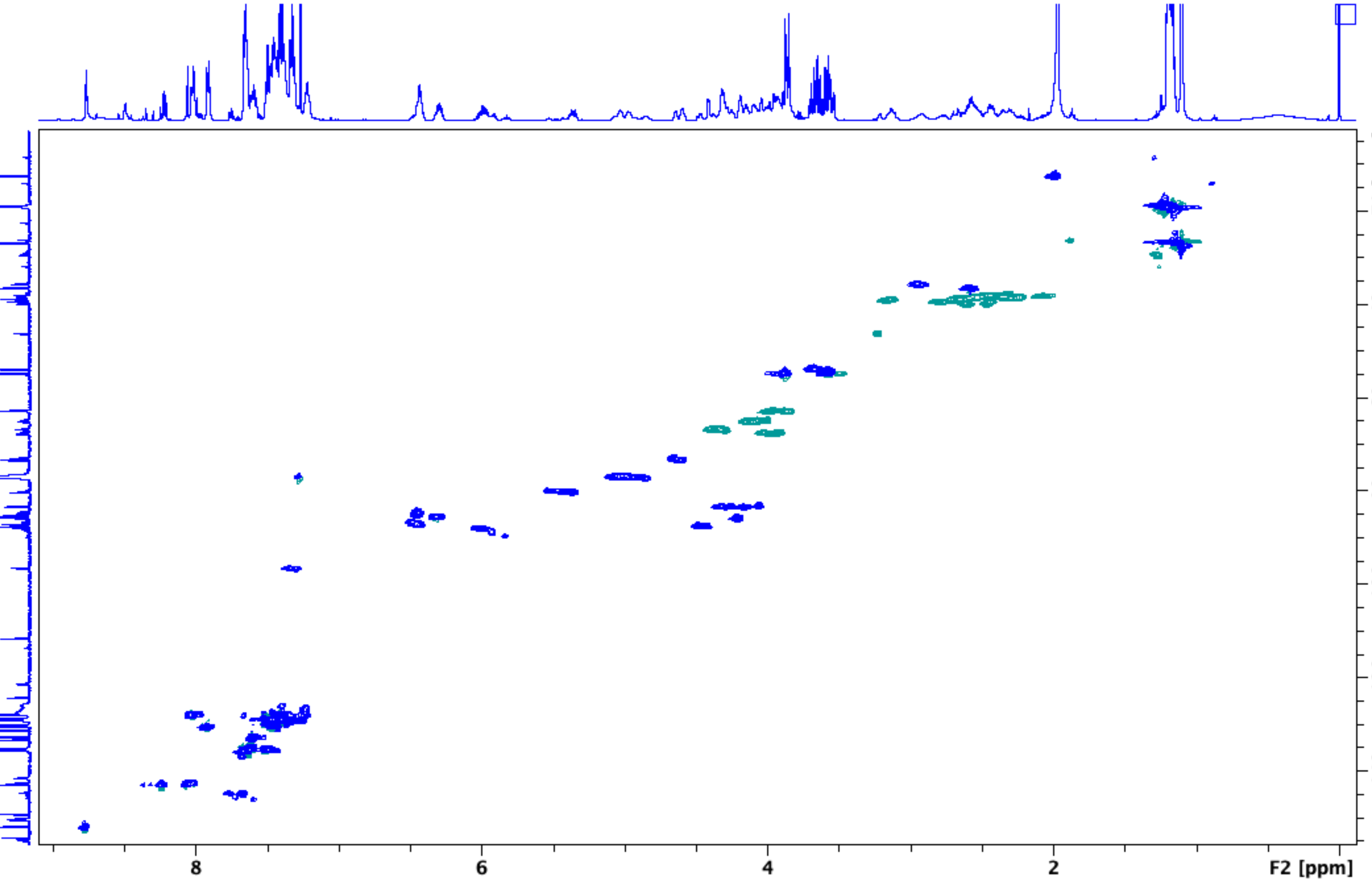

HMBC ($CDCl_3$)

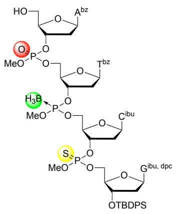

29atcg

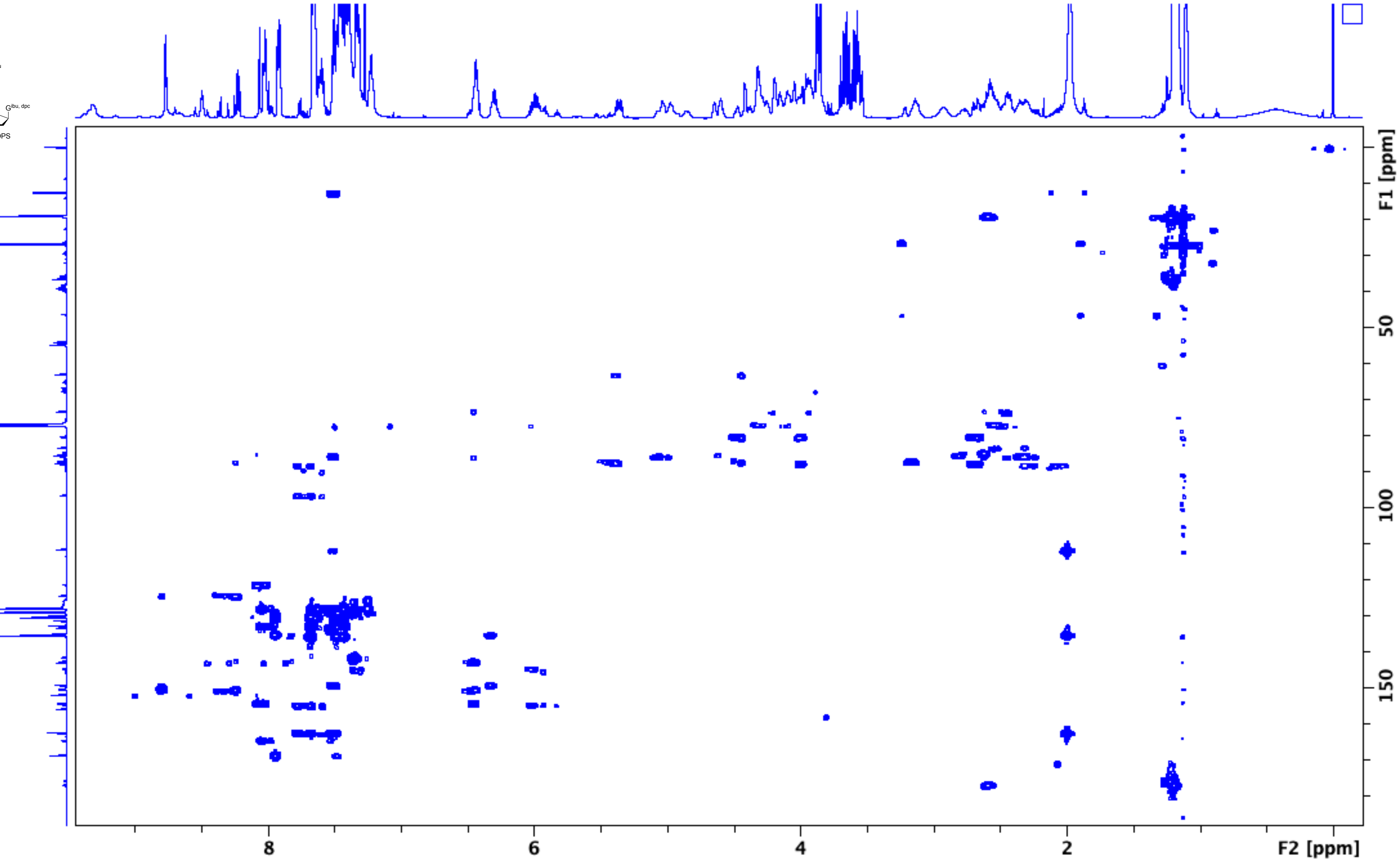

$^{31}P\{^{1}H\}$ NMR ($CDCl_3$, 202 MHz)

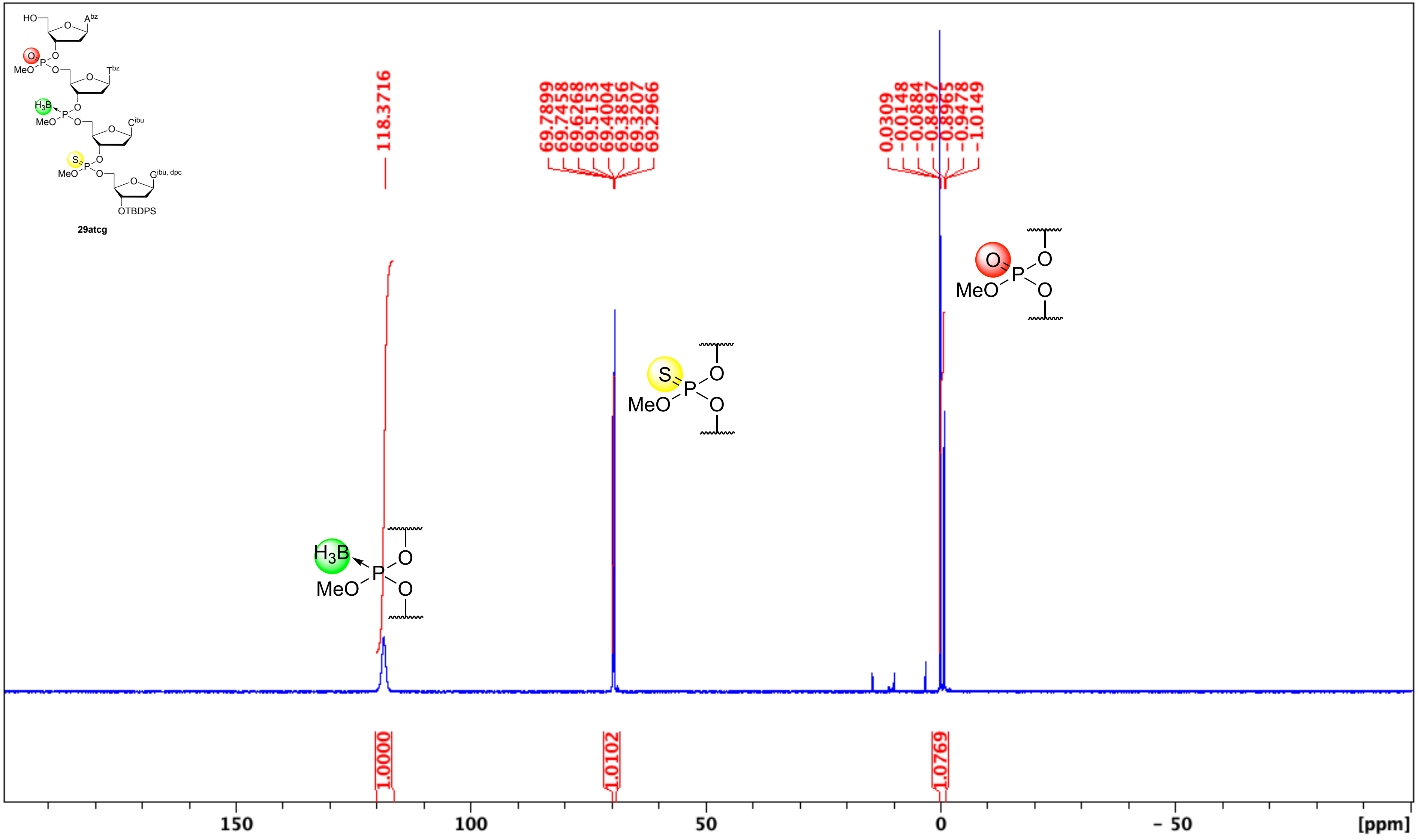

A-T-C-G 4-mer building block bearing *H*-boranophosphonate monoester on 3'-OH (30atcg)

^{1}H NMR ($CDCl_3$, 500 MHz)

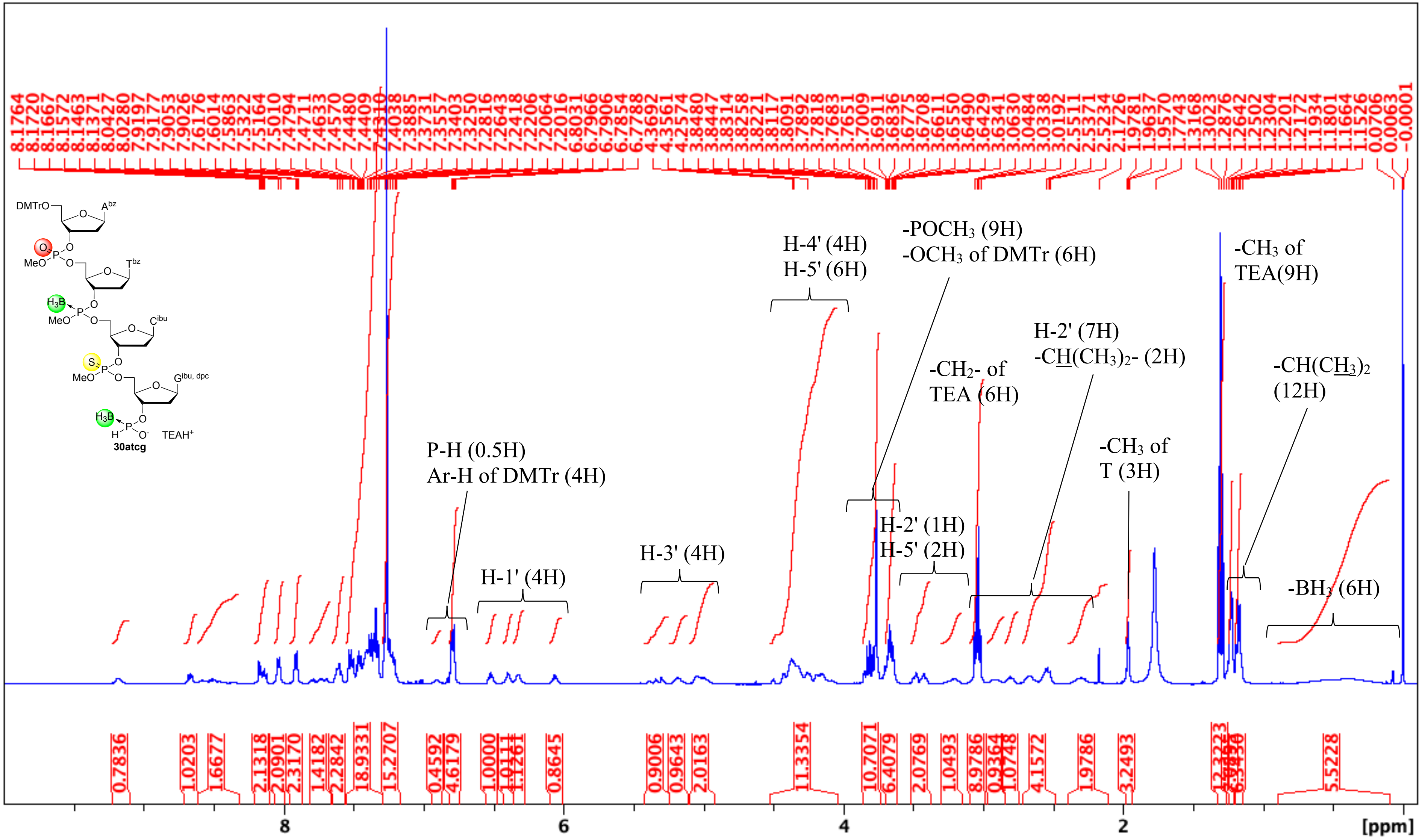

$^{13}C\{^1H\}$ NMR ($CDCl_3$, 126 MHz)

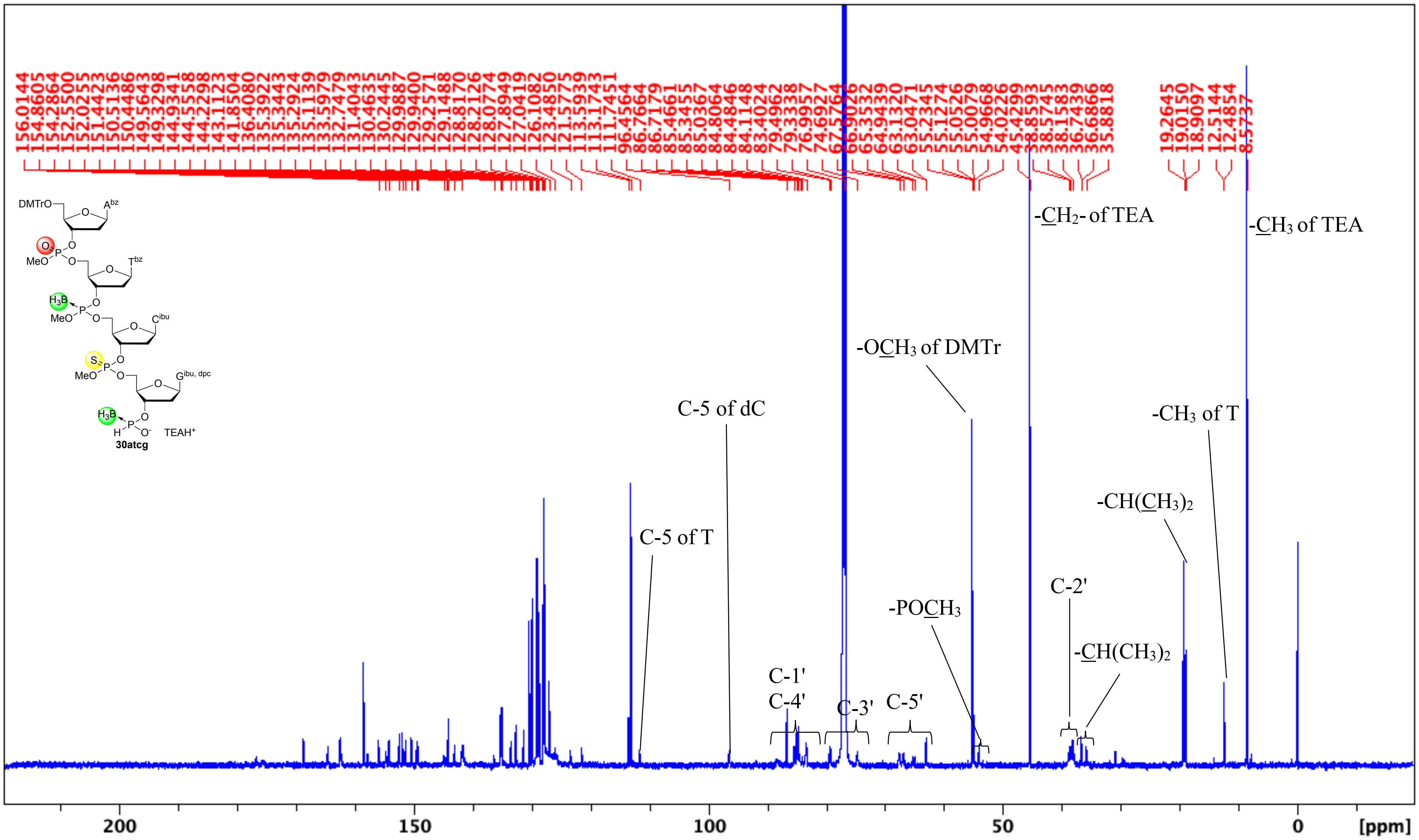

COSY ($CDCl_3$)

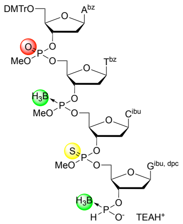

30atcg

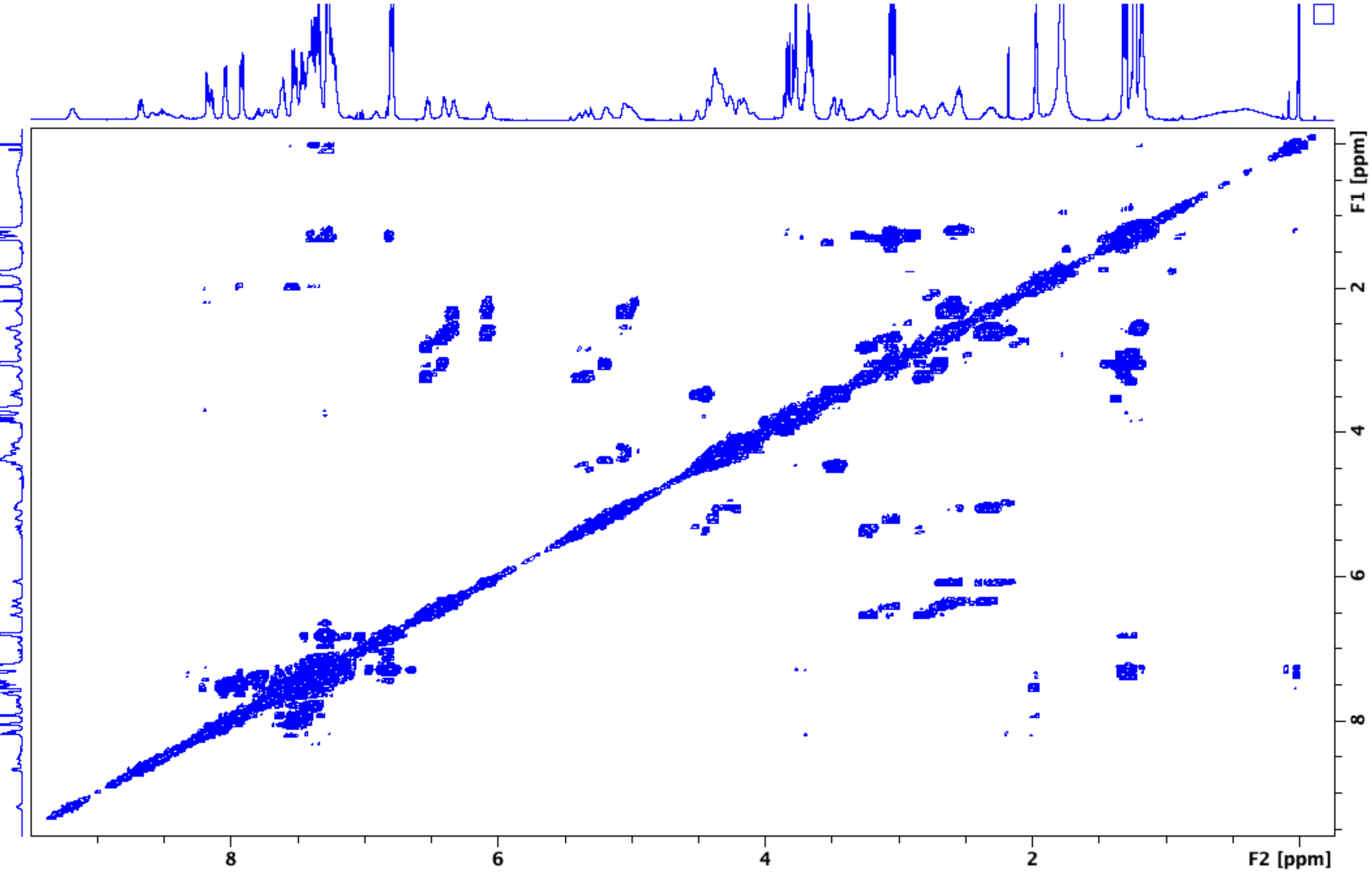

HSQC ($CDCl_3$)

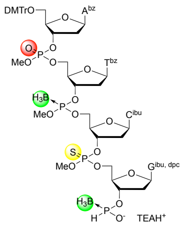

30atcg

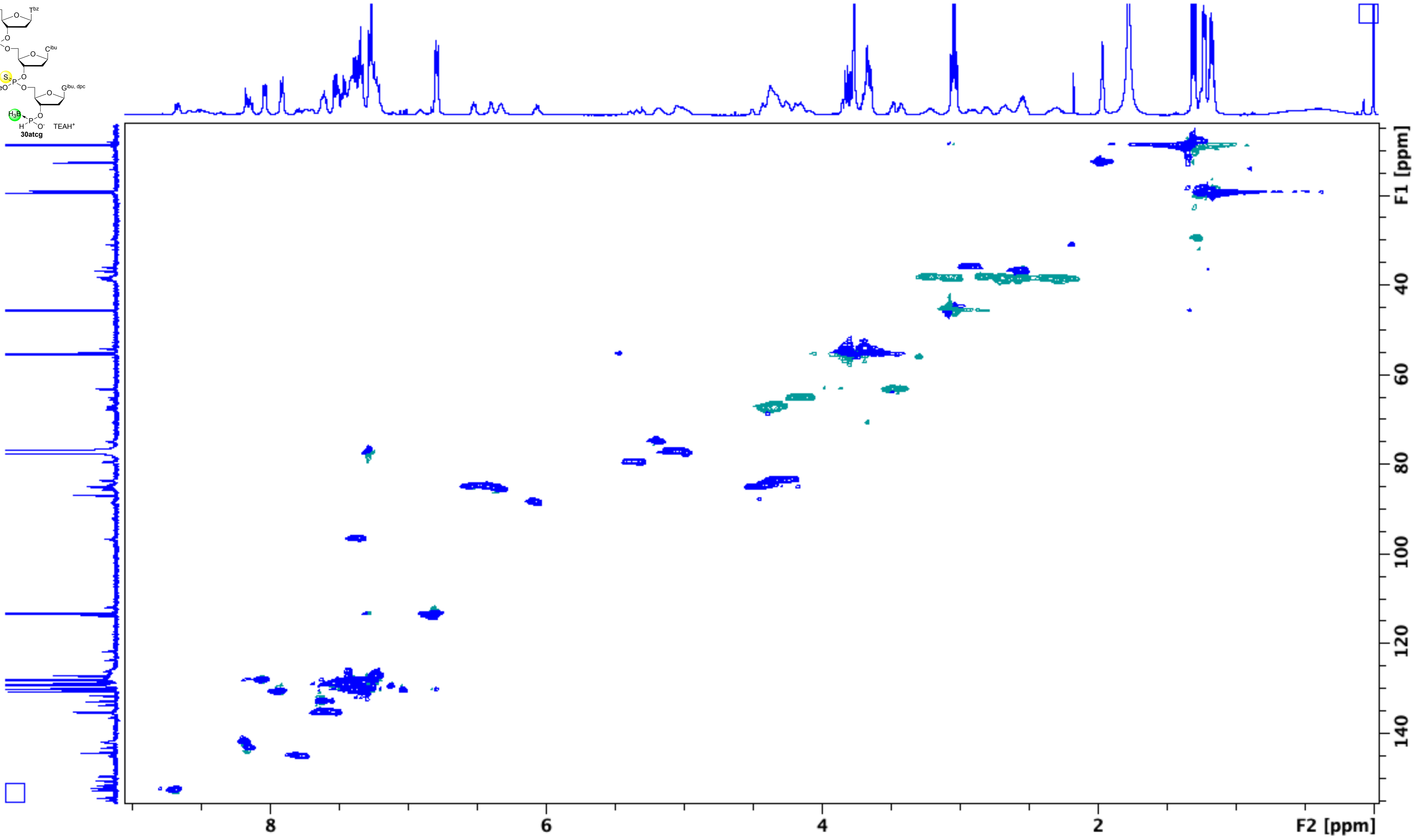

HMBC ($CDCl_3$)

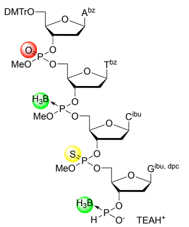

30atcg

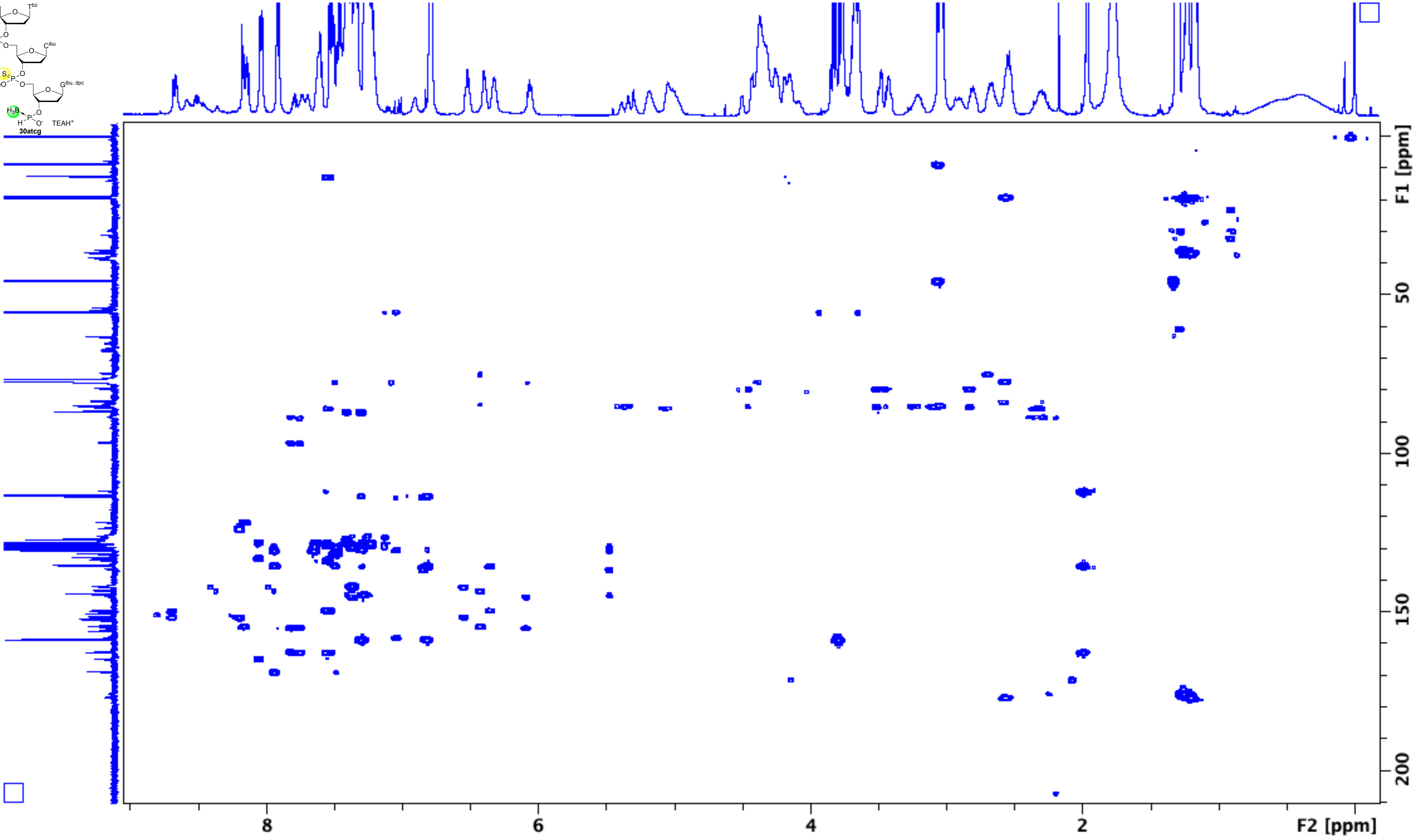

$^{31}P\{^1H\}$ NMR ($CDCl_3$, 202 MHz)

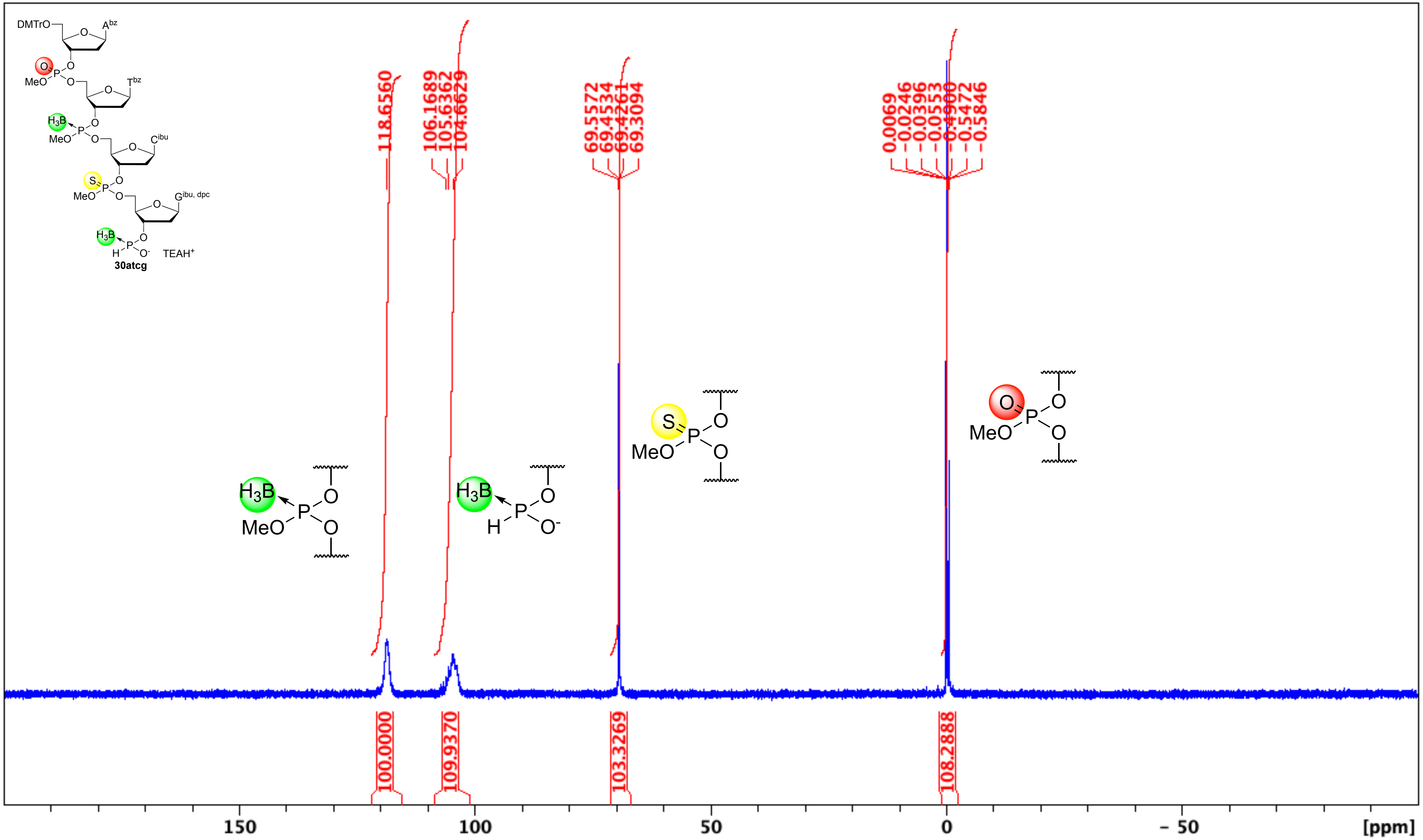

A-T-C-G-A-T-C-G-A-T-C-G 12-mer (33)

^{1}H NMR (500 MHz, D_2O)

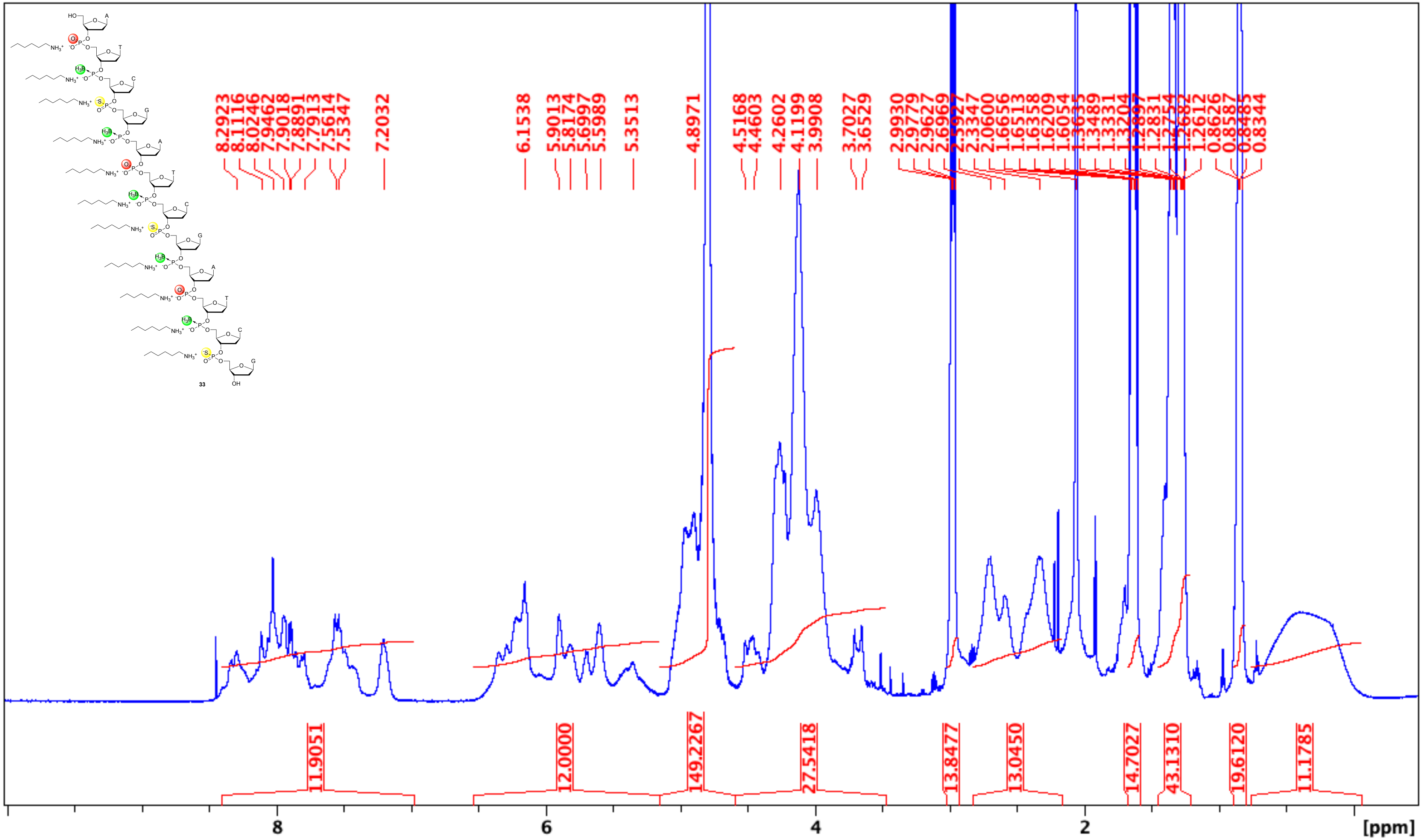

$^{13}C\{^1H\}$ NMR (126 MHz, D_2O)

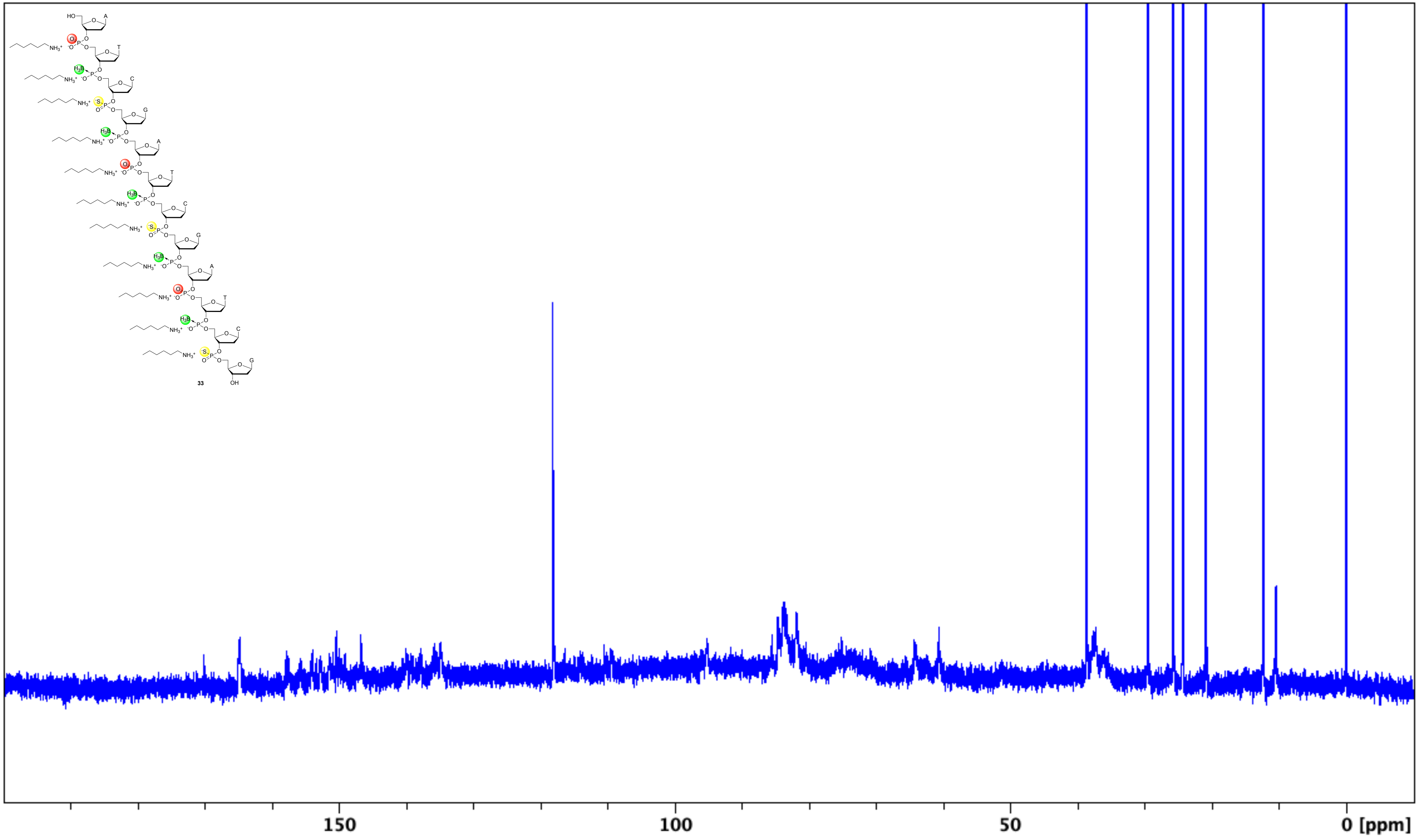

$^{31}P\{^1H\}$ NMR (202 MHz, D_2O)

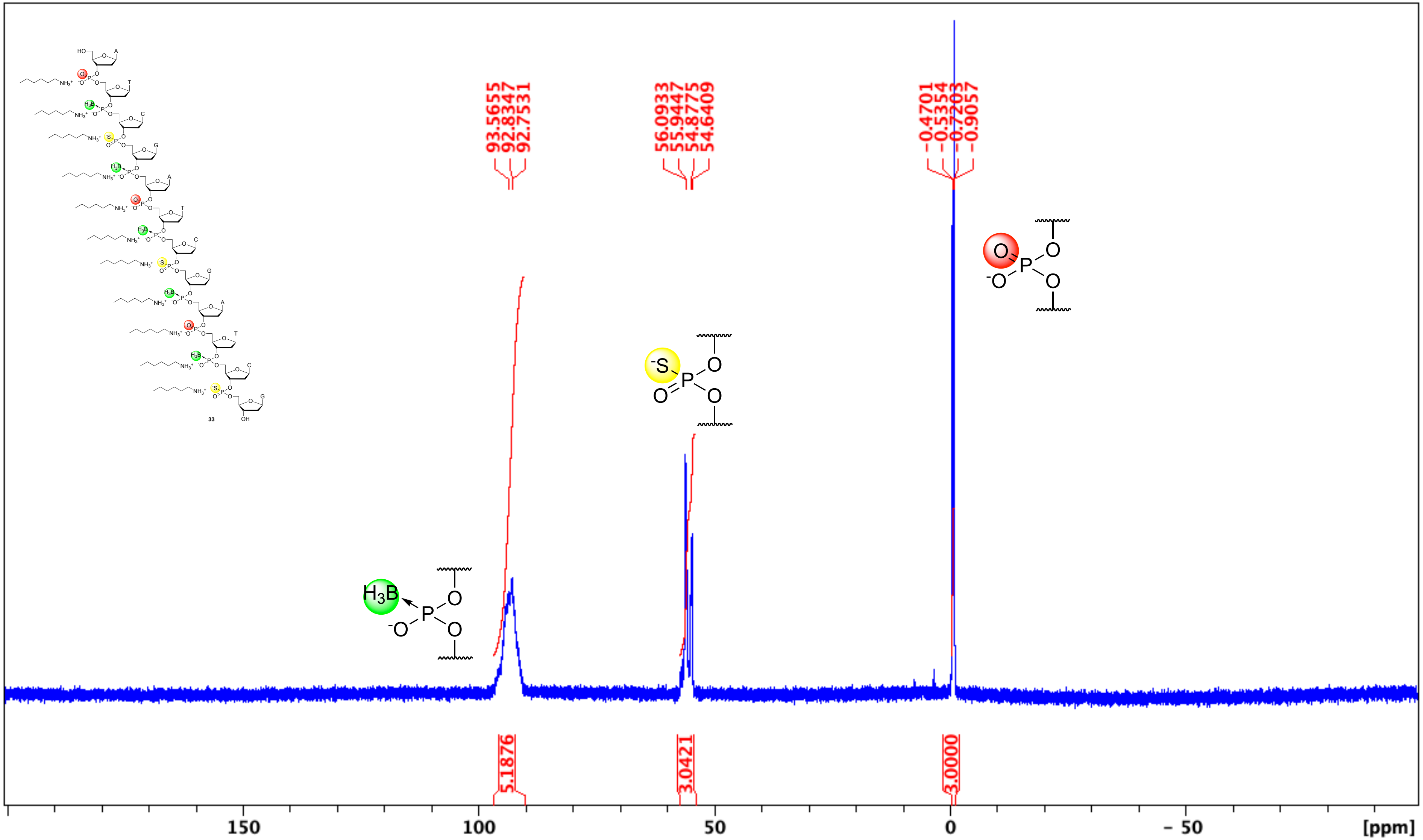

$^{11}B\{^1H\}$ NMR (160 MHz, D_2O)

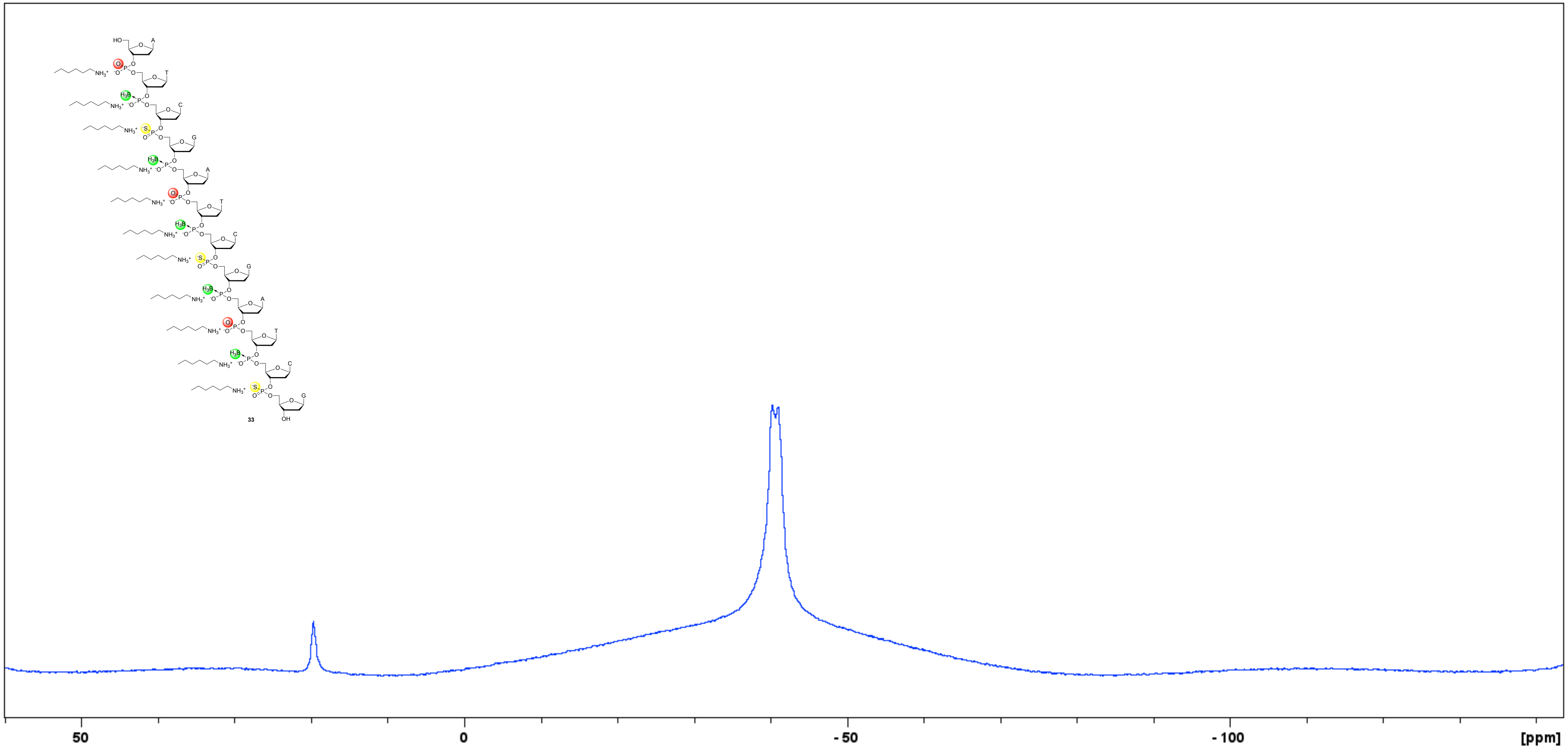

Supplement: Supplementary file 1 [file jo5c00583_si_001.pdf]
